# ΔNp63α promotes radioresistance in esophageal squamous cell carcinoma through the PLEC-KEAP1-NRF2 feedback loop

**DOI:** 10.1038/s41419-024-07194-4

**Published:** 2024-11-05

**Authors:** Jin Tao, Mian Mao, Yuhai Lu, Liyuan Deng, Shuhan Yu, Xiaofei Zeng, Weikun Jia, Zhiqiang Wu, Chenghua Li, Ruidong Ma, Hu Chen

**Affiliations:** 1 Department of Cardiothoracic Surgery, School of Clinical Medicine and The First Affiliated Hospital of Chengdu Medical College, Chengdu, China; 2 Department of Pharmacy, Sichuan Cancer Hospital & Institute, Affiliated Cancer Hospital of University of Electronic Science and Technology of China, Chengdu, China; 3 College of Life Sciences, Sichuan University, Chengdu, China

**Keywords:** Radiotherapy, Radiotherapy

## Abstract

Esophageal squamous cell carcinoma (ESCC) is one of the most aggressive cancers and is highly prevalent in China, exhibiting resistance to current treatments. ΔNP63α, the main isoform of p63, is frequently amplified in ESCC and contributes to therapeutic resistance, although the molecular mechanisms remain unknown. Here, we report that ΔNP63α is highly expressed in ESCC and is associated with radioresistance by reducing ROS level. Furthermore, ΔNP63α plays a critical role in radioresistance by directly transactivating the expression of PLEC. PLEC competitively interacts with KEAP1, resulting in the release of NRF2 from KEAP1 and its translocation from the cytosol to the nucleus, where it activates gene expression to facilitate ROS elimination. Additionally, radiotherapy-induced ROS also activates ΔNP63α expression via NRF2. Pharmacologic inhibition of NRF2 effectively improves radiosensitivity in nude mice. Collectively, our results strongly suggest that the ΔNp63α/PLEC/NRF2 axis plays a key role in radioresistance in ESCC, indicating that targeting NRF2 is a promising therapeutic approach for ESCC treatment.

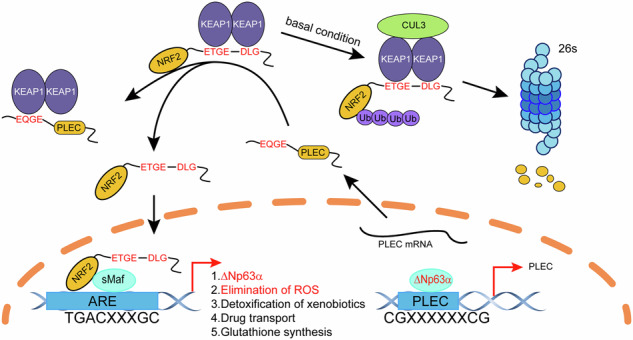

## Introduction

Esophageal cancer is the sixth leading cause of cancer-related death worldwide. Esophageal cancer can be divided into esophageal squamous cell carcinoma (ESCC) and adenocarcinoma (ESCA) based on histopathological characteristics. Almost 70% of global esophageal cancer cases occur in China, with ESCC accounting for 90% of these cases [1]. Over the past decade, clinical approaches for the early diagnosis and treatment of ESCC have evolved, with new paradigms replacing traditional methods at all stages [2]. However, more than 50% of patients with ESCC do not respond adequately to current treatments, and most succumb to recurrent cancer [3]. ESCC is known to exhibit resistance to anticancer therapies. Increasing evidence demonstrates that ESCC readily develops radioresistance, leading to the failure of radiation treatment [4]. Therefore, understanding the mechanism underlying radioresistance is crucial for developing new therapeutic strategies for ESCC.

The p63 protein, encoded by the TP63 gene and a member of the p53/p63/p73 family, is a master regulator that plays a critical role in epidermal differentiation [5]. p63 is also a lineage-dependent oncogene in squamous cell carcinoma (SCC). The TP63 gene produces two major isoforms, TAp63 and N-terminal truncated ΔNp63, using different promoters. Alternative splicing at the C terminus further generates different isoforms (α, β, γ, δ, ε) [6]. ΔNp63α is the prominent isoform of TP63 expressed in epidermal cells and SCC [6, 7], including ESCC [8]. ΔNp63α can trigger ESCC cell invasion and metastasis by activating the β-catenin/c-Myc signaling pathway [9]. ΔNp63α also promotes the growth of ESCC cells via the Akt signaling pathway or DKK3/CKAP4 [10, 11]. It has been reported that the expression of ΔNp63α is correlated with the survivorship in ESCC patients [12]. However, whether ΔNp63α is involved in the therapeutic resistance of ESCC remains largely unknown.

Radiotherapy and chemotherapy, the mainstay treatment for various cancers, kill cancer cells by generating of reactive oxygen species (ROS) with direct or indirect effects [13]. Generally, high levels of ROS are harmful to cells. However, ROS can also trigger tumor formation by inducing DNA mutations and activating oncogenic signaling pathways. Therefore, the regulation of ROS is critical factor in both tumor development and anticancer therapy. NRF2 (encoded by the NFE2L2 gene) is the master regulator of redox homeostasis [14]. Under normal conditions, NRF2 is bound to the KEAP1/CUL3 complex in the cytoplasm, which restricts NRF2 translocation from the cytosol to the nucleus and promotes its degradation via the ubiquitin-proteasome system (UPS). Under oxidative stress, KEAP1 undergoes oxidation and modification, preventing it from binding to NRF2. Consequently, NRF2 is stabilized and translocated into the nucleus [13]. In the nucleus, NRF2 activates the expression of numerous antioxidant and related genes by binding to antioxidant-responsive elements (AREs) (consensus sequence: 5′-TGACNNNGC-3′). The NRF2 pathway has been documented as a driver of cancer progression, metastasis, and resistance to therapy [15].

In this study, we found ΔNp63α is critical for radioresistance in ESCC. ΔNp63α transactivates the expression of PLEC. We describe a direct protein-protein interaction between PLEC and KEAP1, which results in the release of NRF2 from KEAP1 and translocation into the nucleus to activate the expression of antioxidant-related genes. Consequently, radiotherapy-induced ROS is cleared, leading to radioresistance in ESCC. Furthermore, NRF2 can activate the expression of ΔNp63α. Inhibition of NRF2 can reverse ΔNp63α-mediated radioresistance in ESCC cell-derived xenograft mouse model. Taken together, our findings suggest that targeting the ΔNp63α/PLEC/NRF2 signaling pathway could be a promising therapeutic approach for overcoming radioresistance in ESCC.

## Materials and methods

### Cell culture, reagents, and irradiation

Human embryonic kidney cell line HEK-293T, the human immortalized normal esophageal epithelial cell HET-1A, and human lung squamous cell carcinoma cell lines (NCI-H520 and HCC95) were purchased from the American Type Culture Collection. Human ESCC cell lines (TE1, KYSE30, KYSE150, KYSE180, and KYSE450) were generously provided by Professor Yinglan Zhao of Sichuan University. All cell lines were verified through short tandem repeat DNA profiling. Mycoplasma contamination tests were performed, and the results were negative. HEK-293T cells were cultured in DMEM (Gibco, Thermo Fisher Scientific, Shanghai, China) with 10% FBS (Gibco). TE1, KYSE30, KYSE150, KYSE180, KYSE450, NCI-H520 and HCC95 cells were cultured in RPMI-1640 (Gibco) with 10% FBS (Gibco). All cells were maintained at 37 °C in an incubator with 5% CO_2_. N-acetylcysteine (NAC), cycloheximide (CHX), MG-132, chloroquine (CQ), sulforaphane (SFN), and ML385 were purchased from Selleck (Selleck, Shanghai, China). The cells and mice were irradiated by x-rays using the MBR-1520R-3 system (Hitachi Medico Technology) with the indicated dosages.

### Plasmid constructs

Short hairpin RNAs (shRNAs) targeting human p63 or NRF2 were generated by inserting of specific oligonucleotides into a pLKO.1-puromycin lentiviral vector (10878, Addgene, Cambridge, MA, USA). The pLKO.1-scramble (#1864, Addgene) was used as a negative control vector containing scrambled shRNA (shC). The sgRNA targeting KEAP1 was generated by inserting specific oligonucleotides into a lentiCRISPR v2 (#52961, Addgene). The oligonucleotides used in this study are listed below. shp63 #1: GAGTGGAATGACTTCAACTTT, shp63 #2: CATCTGACCTGGCATCTAATT; shNRF2 #1: GCTCCTACTGTGATGTGAAAT, shNRF2 #2: CCGGCATTTCACTAAACACAA; sgKEAP1: TGGGCCGCCTGATCTACACCG.

The constructs encoding human ΔNp63α, ΔNp63α^R304W^, TAp63α, ΔNp63β or ΔNp63γ were described in the previous study [16]. The open reading frames (ORFs) of human RAD51, NRF2, KEAP1 and PLEC was purchased from Miaoling Plasmid Sharing Platform (miaolingbio.com, Wuhan, Hubei, China) and cloned into the lentiviral vector pLVX-puro (632164, Clontech, Mountain View, CA, USA), pLVX-Flag-puro or pLVX-HA-puro. The mutant of constructs was generated by KOD-Plus-Mutagenesis kit (SMK-101, Toyobo, Osaka, Japan). All the constructs were confirmed by DNA sequencing.

For promoter assays, a fragment of human PLEC promoter containing ΔNp63α putative binding sites (P1 and P2) was inserted into the Gluc-On promoter reporter vector (pEZX-PG04, GeneCopoeia, Guangzhou, China) and designated as PLEC-Gluc-WT. The putative binding site P1 or P2 was mutated and designated as PLEC-Gluc-P1 Mut, PLEC-Gluc-P2 Mut or PLEC-Gluc-P1&P2 Mut, respectively. A fragment of the human ΔNp63 promoter containing NRF2 putative binding sites (P1 and P2) was inserted into the Gluc-On promoter reporter vector and designated as ΔNp63-Gluc-WT. The putative binding site P1 or P2 was mutated and designated as ΔNp63-Gluc-P1 Mut, ΔNp63-Gluc-P2 Mut or ΔNp63-Gluc-P1&P2 Mut, respectively.

### Lentiviral packaging and infection

Recombinant lentivirus particles were generated as described previously [16]. Cells were infected with lentivirus at 50% cell confluence with 10 μg/mL polybrene for 24 h, then screened with 2 μg/mL puromycin (A1113803, Gibco) or 10 μg/mL blasticidin (A1113903, Gibco) at 48 h post-infection for stable cells.

### Cell viability assays

For cell viability assays, cells were plated in a 96-well plate at a density of 2 × 10^4^/well and treated with irradiation. CCK8 solution (C0039, Beyotime Biotechnology, Shanghai, China) was added to each well for 2 h and the absorbance was measured at 450 nm wavelength.

### Quantitative RT-PCR

Quantitative RT-PCR (QPCR) was used to detect the mRNA levels as described previously [16]. QPCR primer sequences are listed below. GAPDH-F: AAGGTGAAGGTCGGAGTCAA, GAPDH-R: AATGAAGGGGTCATTGATGG; PLEC-F: AGAGGCACATCAGTGACCTGTA, PLEC-R: ATCATTCCTGATGTTCACCAGCTTC; ΔNp63-F: GGAAAACAATGCCCAGACTC, ΔNp63-R: CTGCGCGTGGTCTGTGTTAT; NFE2L2-F: CGTTTGTAGATGACAATGAGGTTTC, NFE2L2-R: GCCTGATTAGTAGCAATGAAGACTG.

### Reactive oxygen species (ROS) measurement

ROS levels in cells were determined using a Reactive Oxygen Species Assay Kit (Beyotime Biotechnology) according to the manufacturer’s instructions. After treatment with irritation, cells were incubated with DCFH-DA at 37 °C, and ROS-mediated oxidation of the fluorescent compound DCF was measured. Fluorescence of oxidized DCF was measured at an excitation wavelength of 488 nm and an emission wavelength of 535 nm using a FACS Scan flow cytometer (BD Biosciences).

### Immunofluorescent analysis

For Immunofluorescent analyses, cells grown on coverslips were fixed with 4% polyformaldehyde in PBS, permeabilized in 0.1% Triton X-100, blocked with 4% bovine serum albumin in PBS, incubated with mouse-anti-KEAP1 (60027-1-Ig, Proteintech, 1:100) and rabbit-anti-PLEC (ab312312, Abcam, 1:100) overnight at 4 °C, and then treated with Rhodamine (TRITC)-conjugated donkey-anti-mouse IgG (715-025-151, Jackson ImmunoRsearch, PA, USA) or Fluorescein (FITC)-conjugated donkey-anti-rabbit IgG (711-095-152, Jackson ImmunoRsearch) for 1 h in the dark at room temperature. Nuclei were stained with DAPI for 5 min at room temperature. Coverslips were mounted with Antifade Mounting Medium (P0126, Beyotime Biotechnology). Images were acquired using Leica TCS SP5 II system.

### Cell fractionation

For separation of nuclear and cytoplasmic fractions, the extraction kit (Beyotime China, P0027) was used. Briefly, 2 × 10^6^ cells were collected, washed twice with cold PBS, and resuspended in 200 μL cytoplasm extraction buffer A and B for 10 min on ice, and centrifuged at 200 × *g* for 5 min at 4 °C. The supernatant was a cytoplasmic fraction. The nuclear pellets were washed with buffer A and then resuspended in 1 mL nuclear extraction buffer for 10 min on ice, and centrifuged at 1000 × *g* for 10 min at 4 °C.

### Immunoblot and immunoprecipitation analysis

Immunoblot analysis was used to detect protein level as described previously [16]. Antibodies for ATM (2873, 1:1000), p-ATM (5883, 1:100), CHK2 (2662, 1:1000), p-CHK2 (2661, 1:1000), RAD51 (8875, 1:1000), p21 (2947, 1:1000), LC3 (3868, 1:1000), HA-tag (3724, 1:1000), were purchased from Cell Signaling Technology (Danvers, MA, USA). Antibodies for NRF2 (ab62352, 1:1000), PLEC (ab312312, Abcam, 1:100) were purchased from Abcam (Cambridge, MA, USA). Antibody for Flag-tag (F1804, 1:1000) was purchased from Sigma (Shanghai, China). Antibody for ΔNp63 (619002, Biolegend, 1:500) was purchased from BioLegend. Antibody for p63 (381215, 1:1000) was purchased from ZEN-Bioscience (Chengdu, China). Antibodies for KEAP1 (60027-1-Ig, 1:1000), SP1(21962-1-AP, 1:1000) was purchased from Proteintech (Wuhan, China). Antibodies for NQO1 (CY6710, 1:1000) and GAPDH (AB0036, 1:5000) were purchased from Abways Technology (Shanghai, China).

Immunoprecipitation analysis was used to detect protein-protein interaction. Cells were lysed in lysis buffer (50 mM Tris HCl, pH 7.4, with 150 mM NaCl, 1 mM EDTA, and 1% Nonidet P-40). The lysate was then centrifuged at 15,000 × *g* at 4 °C to remove precipitation. The supernatants were incubated with mouse-anti-KEAP1 (60027-1-Ig, Proteintech, 1:100), rabbit-anti-PLEC (ab312312, Abcam, 1:100), rabbit-anti-NRF2 (ab62352, Abcam, 1:100), mouse-anti-Flag (F1804, Sigma, 1:100), rabbit-anti-HA (3724, CST 1:100), normal mouse IgG (sc-2025, Santa Cruz, 1:10) or normal rabbit IgG (2729, CST, 1:200) on a rotator overnight at 4 °C, followed by addition of protein A (sc-2001, Santa Cruz) or protein G (sc-2002, Santa Cruz) agarose beads and further incubation for 2 h at 4 °C. After washing with lysis buffer, the immunocomplexes were analyzed by immunoblotting.

### Mass spectrometry

Mass spectrometry (MS) analysis was used to detect associated protein and performed as described previously [17]. Briefly, the immunocomplexes were subject to dehydration, reduction, digestion, alkylation, and extraction. Peptides were separated and eluted. Data-dependent acquisition was performed in positive ion mode. Full MS was acquired in the Orbitrap mass analyzer. The raw files obtained were searched against the Swiss-Prot human protein sequence database (updated on 01/2017; 20, 413 protein sequences) using MaxQuant (version 1.6). The analyzed data of mass spectrometry was listed in Supplementary Table 1.

### Ubiquitination assay

To detect the ubiquitination levels of endogenous NRF2, cells were transfected with or without the indicated plasmids and treated with 10 μM MG132 (M8699, Sigma- Aldrich) for 4 h to block proteasomal degradation. The cells were lysed and immunoprecipitated for 4 h at 4 °C with Protein A Magnetic beads (P2102, Beyotime Biotechnology) loaded or bound with anti-NRF2 (ab62352, Abcam, 1:100) antibody according to the immunoprecipitation assay described above. The immunoprecipitated proteins were subjected to immunoblotting analysis with antibody against ubiquitin antibody (10201-2-AP, Proteintech, 1:1000).

### Publicly database analysis

The TCGA datasets (cBioPortal, www.cbioportal.org/) were used to analyze the correlation of mRNA between TP63 and PLEC, or between NFE2L2 and TP63 in esophageal carcinoma (TCGA, Firehose Legacy).

### Dual-luciferase reporter assay

Luciferase reporter assays were performed with Secrete-Pair^TM^ Dual Luminescence Assay Kit (GeneCopoeia, USA) according to the manufacturer’s instructions. For *PLEC* gene promoter analyses, cells were co-transfected with 500 ng of PLEC-Gluc reporters (PLEC-Gluc-WT, PLEC-Gluc-P1 Mut, PLEC-Gluc-P2 Mut, or PLEC-Gluc-P1&P2 Mut) and 750 ng of ΔNp63α expression plasmids (ΔNp63α^WT^ or ΔNp63α^R304W^) or empty vector (EV). For *ΔNp63* gene promoter analyses, cells were co-transfected with 500 ng of ΔNp63-Gluc reporters (ΔNp63-Gluc-WT, ΔNp63-Gluc-P1 Mut or ΔNp63-Gluc-P2 Mut) and 750 ng of NRF2 expression plasmids (NRF2^WT^, NRF2^ΔNhe2^) or empty vector (EV). Forty-eight hours post-transfection, cell culture media were collected and ΔNp63-Gluc and SEAP activities were measured. The PLEC-Gluc or ΔNp63-Gluc activity was normalized to SEAP activity.

### Chromatin immunoprecipitation (ChIP) assays

The JASPAR database (http://jaspar.genereg.net/) was used to predict the potential transcription factor binding site on the *PLEC* enhancer or *ΔNp63* promoter. Based on the predicted results from JASPAR, ChIP assays were used to confirm the binding regions of ΔNp63α on the *PLEC* enhancer or NRF2 on the *ΔNp63* promoter in KYSE450 cells with ChIP-IT Kit (53009, Active Motif, USA) using antibodies specific for ΔNp63 (619502, Biolegend, 1:50), NRF2 (ab62352, Abcam, 1:50) or normal rabbit IgG (2927, CST, 1:50), as described previously [18]. Immunoprecipitated DNA was subjected to PCR to amplify fragments of the ΔNp63 promoter elements using indicated primers listed in Supplementary Table 4.

### CDX tumors and drug sensitivity assay

Animal experiments were performed following Guidelines for Animal Experiments at Chengdu Medical College with the approval of the Institutional Animal Care and Use Committee. Male athymic nude mice (5 weeks old) were purchased from GemPharmatech (Nanjing, China) and fed a standard chow diet. KYSE150 stable cells were injected subcutaneously into the flank of mice, and tumor size was measured with a digital caliper at 7-day intervals. Tumor volumes were calculated using the following formula: length × width^2^ × 0.5. Once the tumor volumes reached about 100 mm^3^, mice bearing tumor expressing empty vector (EV) were randomly assigned 2 groups: blank, irradiation (5 Gy, once per week for 3 weeks); mice bearing tumor expressing ΔNp63α were randomly assigned 3 groups: blank, irradiation (5 Gy, once per week for 3 weeks), irradiation in combination with ML385 (30 mg/kg, 5 times per week for 3weeks). At the end of treatment, all mice were euthanized, and tumors were excised, weighed. fixed, embedded, and sectioned. Tumor sections were subjected to IHC staining for Ki67 and Cleaved Caspase 3.

### Immunohistochemistry (IHC)

IHC analyses were performed as previously described [19]. The antibodies used were: ΔNp63 (619002, biolegend, 1:100), NRF2 (ab62352, abcam, 1:100), Ki67 (27309-1-AP, Proteintech, 1:100) and Cleaved Caspase 3 (9664, CST, 1:100). Positively stained signals were scanned with a NanoZoomer (Hamamatsu, Japan). To evaluate the expression of Ki67 or Cleaved Caspase 3, positively stained cells were counted. The percentage of positive cells was determined independently by three authors (JT, MM, and YL). To compare the protein expression differences in the different specimens, the AOD (average optical density) score was calculated as previously described [19].

### Statistical analysis

Data from cell culture experiments were performed in triplicate and presented as means ± SD. GraphPad Prism 8 software was used for all statistical analysis. Unless otherwise indicated, differences between the two groups were tested using the two-tailed unpaired Student’s t-test. Homogeneity of clinical data were performed using Levene’s test in SPSS 16 software. The level of significance is indicated as * p < 0.05, ** p < 0.01.

## Results

### ΔNp63α promotes ESCC radioresistance by reducing radiotherapy-induced ROS

**Fig. 1:**
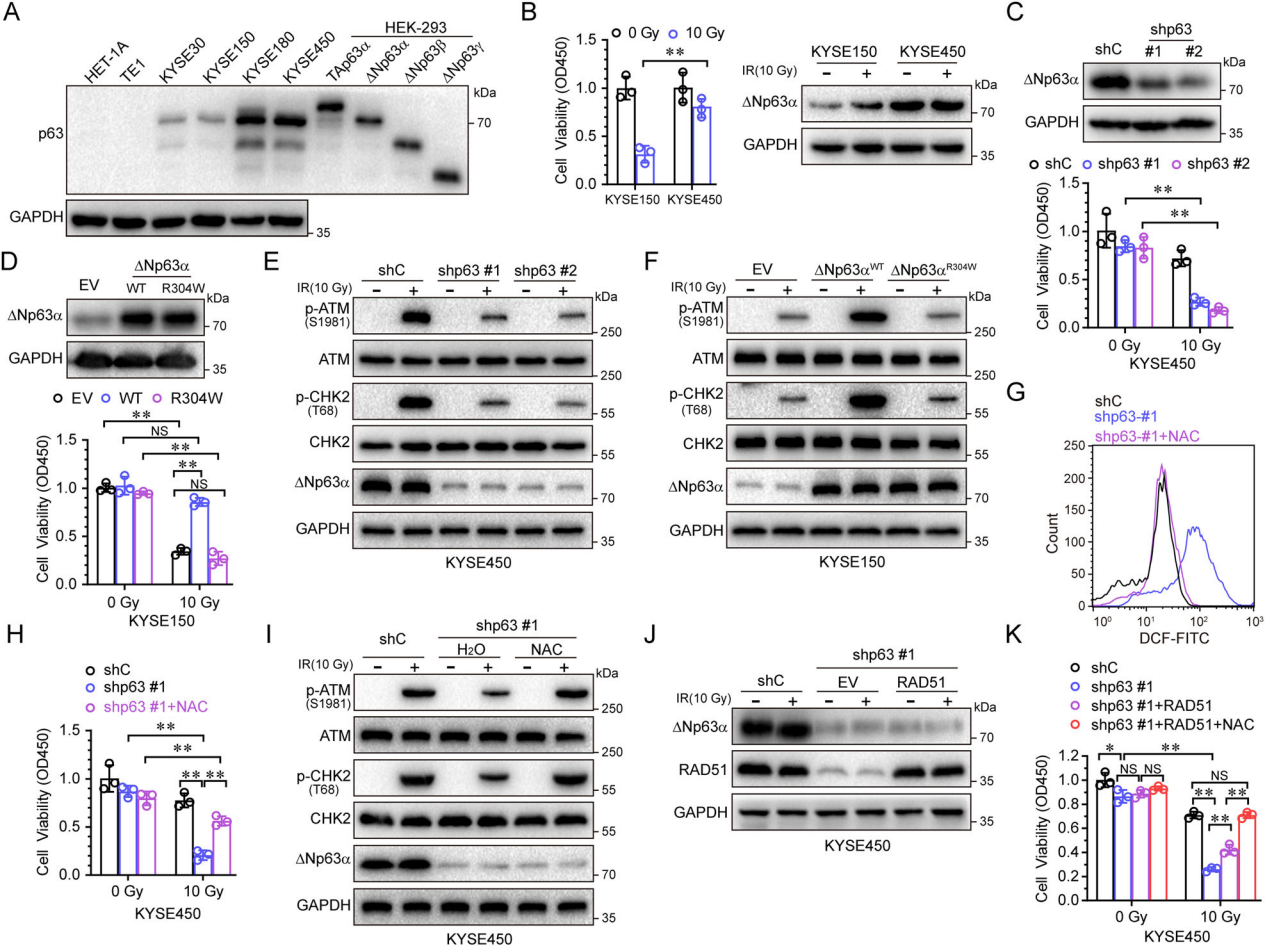
ΔNp63α promotes radioresistance by inhibiting radiotherapy-induced ROS. **A** Whole cell lysates derived from HET-1A, TE-1, KYSE30, KYSE150, KYSE180, KYSE450 cells, and HEK-293T cells transiently transfected with TAp63α, ΔNp63α, ΔNp63β or ΔNp63γ were subjected to immunoblot analyses. **B** KYSE150 or KYSE450 cells were treated with the indicated irradiation doses, then subjected to test cell viability testing using CCK8 (left panel) and immunoblot analyses (right panel). **C** KYSE450 cells stably expressing a control shRNA (shC) or two different shRNAs specific for p63 were treated with the indicated irradiation doses, then subjected to immunoblot analyses (left panel) and cell viability testing (right panel). **D** KYSE150 cells stably expressing ΔNp63α (WT and R304W) were treated with the indicated irradiation doses, then subjected to immunoblot analyses (left panel) and cell viability testing (right panel). **E** KYSE450 cells stably expressing a control shRNA (shC) or two different shRNAs specific for p63 were treated with the indicated irradiation dose, then subjected to immunoblot analysis for phosphorylated and total amounts of the checkpoint proteins ATM and CHK2. **F** KYSE150 cells stably expressing ΔNp63α (WT and R304W) were treated with the indicated irradiation doses, then subjected to immunoblot analyses. **G**–**I** KYSE450 cells stably expressing a control shRNA (shC) or shRNAs specific for p63 (shp63-#1) were treated with or without NAC (1 mM), then subjected to the indicated irradiation doses. The ROS (**G**), cell viability (**H**) and protein levels (**I**) were tested by FACS, CCK8 or immunoblot analyses, respectively. KYSE450 cells stably expressing a control shRNA (shC) or shRNA specific for p63 (shp63-#1) were infected with lentivirus expressing RAD51 or empty vector (EV), treated with the indicated irradiation dose, then subjected to immunoblot analyses (**J**) and cell viability testing (**K**). Results are presented as means ± SD from three independent experiments in triplicates. **P < 0.01, NS no significance.

*TP63* gene was amplified and overexpression in ESCC (Supplementary Fig. 1A, B). To evaluate whether ΔNp63α plays crucial role in radiotherapy, we first screened a panel of ESCC cell lines and selected two cell lines each with low (KYSE30 and KYSE150) and elevated (KYSE180 and KYSE450) endogenous levels of ΔNp63α expression (Fig. 1A). Next, we treated the cells with irradiation and measured the cell viability. As shown in Fig. 1B, KYSE450 cells with high ΔNp63α levels exhibited increased survival after irradiation compared with KYSE150 cells. Irradiation with 10 Gy resulted in ΔNp63α protein elevated slightly in KYSE150 cells, not affected ΔNp63α expression in KYSE450 cells (Fig. 1B, right). In contrast, ΔNp63α knockdown significantly decreased cell survival following irradiation treatment (Fig. 1C). Conversely, ectopic expression of wild-type ΔNp63α, but not the transactivation-defective mutant (R304W), markedly increased cell survival under irradiation (Fig. 1D). These results indicate that ΔNp63α plays critical role in ESCC radioresistance.

Radiotherapy exerts its therapeutic effect primarily through DNA damage induced by ionizing radiation and reactive oxygen species (ROS), which break DNA’s chemical bonds via free radicals [20]. Thus, DNA damage checkpoint responses are critical in cellular radiosensitivity. The activating phosphorylation of checkpoint proteins ATM and CHK2 induced by irradiation was significantly increased in ΔNp63α-overexpressing KYSE150 cells but decreased in ΔNp63α-knockdown KYSE450 cells (Fig. 1E, F), indicating that ΔNp63α promotes checkpoint activation in response to DNA damage. Furthermore, irradiation-induced ROS was significantly elevated in ΔNp63α-knockdown KYSE450 (Fig. 1G). Clearance of the ROS by N-Acetylcysteine (NAC) dramatically increased cell survival (Fig. 1G, H) and upregulated p-AMT and p-CHK2 (Fig. 1I) inhibited by irradiation. It has been reported ΔNp63α transactivates RAD51, a recombinase, to maintain genome integrity [21]. We restored RAD51 expression in ΔNp63α-knockdown KYSE450 cells (Fig. 1J) and found that RAD51 significantly, but not completely, reversed cell survival after irradiation treatment (Fig. 1K). However, RAD51 restoration combined with ROS clearance fully restored cell survival inhibited by irradiation (Fig. 1K), indicate that RAD51 is not solely sufficient without addressing ROS-induced damage. Taken together, these data demonstrate that ΔNp63α promotes radioresistance by reducing radiotherapy-induced ROS.

### ΔNp63α increases NRF2 protein stability to inhibit ROS

**Fig. 2:**
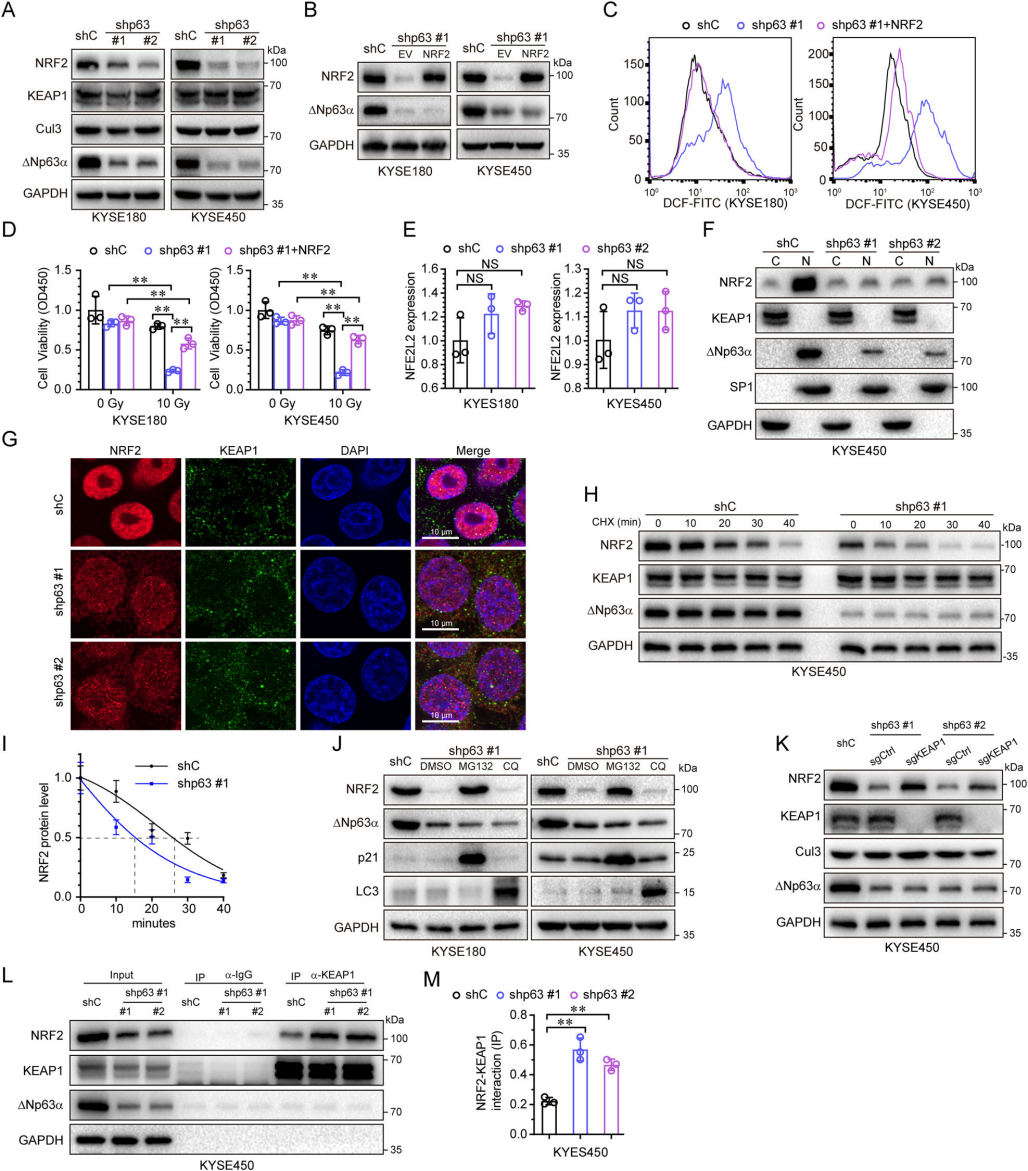
ΔNp63α inhibits ROS by maintaining the protein stability of NRF2. **A** KYSE180 or KYSE450 cells stably expressing a control shRNA (shC) or two different shRNAs specific for p63 were subjected to immunoblot analyses. KYSE180 or KYSE450 cells stably expressing a control shRNA (shC) or shRNA specific for p63 (shp63-#1) were infected with lentivirus expressing NRF2 or empty vector (EV), treated with the indicated irradiation dose, then subjected to immunoblot analyses (**B**) ROS level measurement (**C**) and cell viability testing (**D**). **E** KYSE180 or KYSE450 cells stably expressing a control shRNA (shC) or two different shRNAs specific for p63 were subjected to QPCR analyses. KYSE450 cells stably expressing a control shRNA (shC) or two different shRNAs specific for p63 were subjected to cell fractionation analyses (C: cytoplasm, N: nucleus) (**F**) and immunofluorescent analyses (**G**). KYSE450 cells stably expressing a control shRNA (shC) or shRNA specific for p63 (shp63 #1) were treated with CHX for indicated times, then subjected to immunoblot analyses (**H**) and quantification (**I**). **J** KYSE180 or KYSE450 cells stably expressing a control shRNA (shC) or shRNA specific for p63 (shp63 #1) were treated with MG132 (10 μM), CQ (20 μM) or DMSO for 24 h, then subjected to immunoblot analyses. **K** KYSE450 cells stably expressing a control shRNA (shC) or two different shRNAs specific for p63 were infected with lentivirus expression a control sgRNA (sgCtrl) or sgRNA specific for KEAP1 (sgKEAP1), then subjected to immunoblot analyses. **L** KYSE450 cells stably expressing a control shRNA (shC) or two different shRNAs specific for p63 were subjected to anti-KEAP1 (or anti normal mouse IgG1) immunoprecipitation (IP); the coprecipitating endogenous NRF2 was examined by immunoblot analyses (IB). **M** the interaction of NRF2 with KEAP1 were quantified on the base of KEAP1 immunoprecipitation. Results are presented as means ± SD from three independent experiments in triplicates. **P < 0.01, NS no significance.

The transcription factor NRF2 is known as the master regulator of the cellular antioxidant response [15]. To investigate whether NRF2 is involved in ΔNp63α-mediated radioresistance in ESCC, we knocked down ΔNp63α and measured the expression of NRF2. Knockdown of ΔNp63α resulted in a significant downregulation of NRF2 protein (Fig. 2A). We then restored NRF2 expression in ΔNp63α-knockdown KYSE180 or KYSE450 cells (Fig. 2B), and found that NRF2 markedly reduced the ROS level induced by irradiation (Fig. 2C), and increased cell survival (Fig. 2D).

To understand how ΔNp63α regulated NRF2 expression, we measured the mRNA level in ΔNp63α-knockdown KYSE180 or KYSE450 cells, and found that knockdown of ΔNp63α had a little effect on NRF2 (NFE2L2) mRNA level (Fig. 2E), suggesting that ΔNp63α affects the protein level of NRF2. Cell fractionation and immunofluorescence analyses showed that knockdown of ΔNp63α in KYSE450 cells led to a decrease of NRF2 in the nuclei (Fig. 2F, G). Next, we measured the protein half-life of NRF2 in ΔNp63α-knockdown KYSE450 cells by using cycloheximide (CHX) treatment and found that ΔNp63α knockdown reduced the protein half-life of NRF2 (Fig. 2H, I), indicating that ΔNp63α regulates the protein stability of NRF2.

To investigate how ΔNp63α affects NRF2 protein stability, we treated ΔNp63α-knockdown KYSE180 or KYSE450 cells with a proteasome inhibitor (MG132) or a lysosome inhibitor (chloroquine, CQ), and found that only MG132 blocked the degradation of NRF2 (Fig. 2J). It is well-documented that E3 ubiquitin ligase complex, KEAP1-Cul3, controls the ubiquitylation and proteasomal degradation of NRF2 in response to redox stress [15]. KEAP1 is an adapter that brings NRF2 into the E3 ligase complex. However, we found the knockdown of ΔNp63α had little effect on the expression of KEAP1 or Cul3 (Fig. 2A, K and Supplementary Fig. 2A, B), suggesting that ΔNp63α does not affect the complex of KEAP1-Cul3 to regulate NRF2 protein stability. To further examine whether KEAP1 was involved in ΔNp63α-knockdown-mediated NRF2 protein degradation, we depleted KEAP1 using CRISPR in ΔNp63α-knockdown KYSE450 cells. We found that KEAP1 knockdown significantly upregulated NRF2 protein expression which was downregulated in ΔNp63α-knockdown KYSE450 cells (Fig. 2K). Our immunoprecipitation assay clearly showed that ΔNp63α knockdown promoted the interaction of KEAP1 with NRF2 (Fig. 2L, M). These results suggest that ΔNp63α inhibits the KEAP1-NRF2 interaction, stabilizes NRF2 protein, suppresses radiotherapy-induced ROS, and subsequently promotes cell survival.

### ΔNp63α inhibits the interaction of KEAP1 with NRF2 via PLEC

**Fig. 3:**
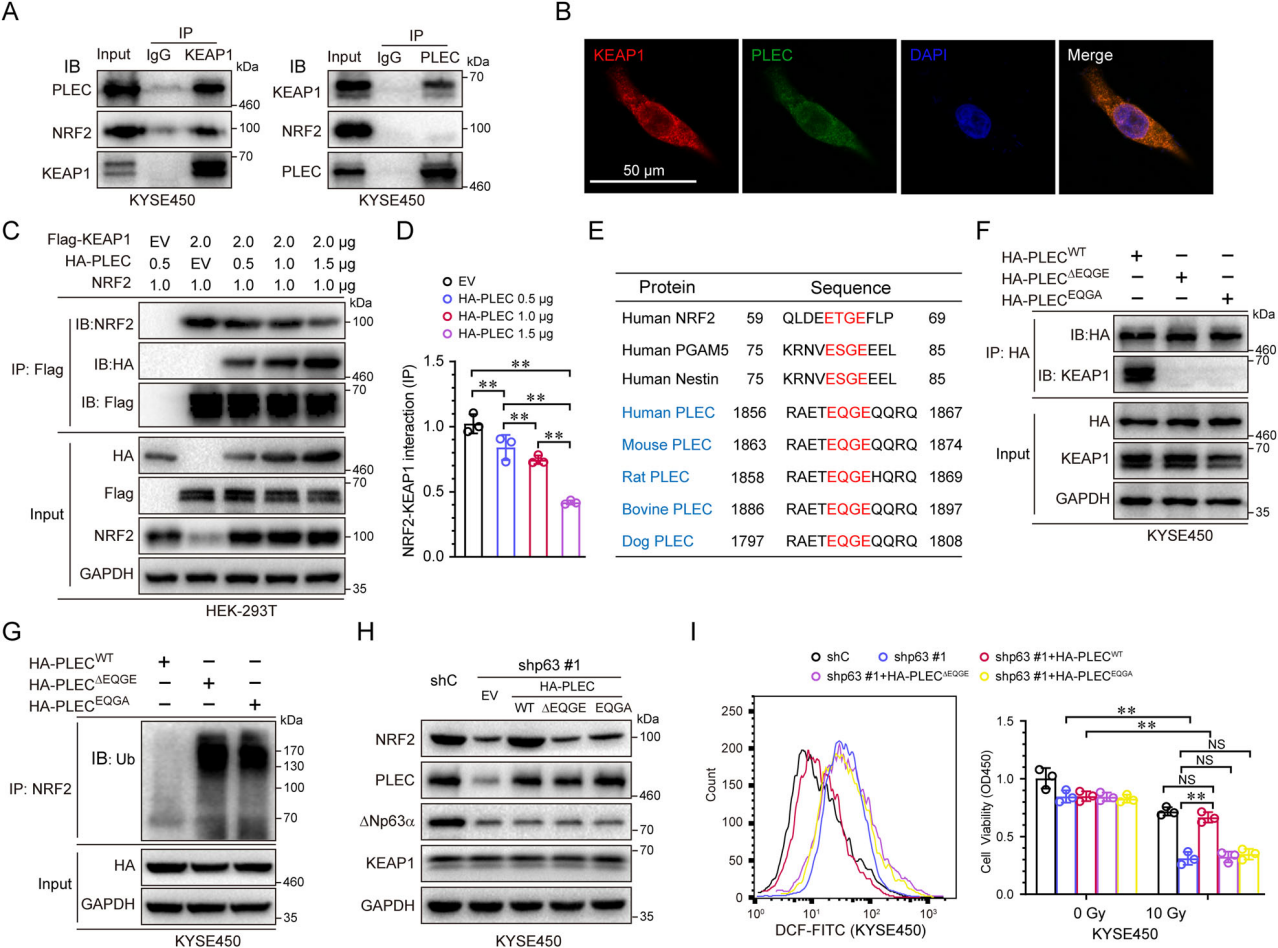
ΔNp63α protects NRF2 from ubiquitin-proteasome degradation by PLEC. **A** KYES450 cells were subjected to anti-KEAP1 (or anti-PLEC) immunoprecipitation (IP); the coprecipitating endogenous PLEC (or KEAP1) were examined by immunoblot analyses (IB). **B** KYSE450 cells were subjected to immunofluorescence (IF). **C** HEK-293T cells, expressing NRF2, Flag-tagged KEAP1 and/or HA-tagged PLEC, were subjected to anti-Flag immunoprecipitation (IP); the co-precipitating NRF2, HA-PLEC, Flag-KEAP1 were examined by immunoblot analyses (IB). **D** the interaction of NRF2 with KEAP1 were quantified on the base of KEAP1 immunoprecipitation. **E** Sequence alignment highlighting the putative Keap1-binding motif in PLEC from different species and those previously reported in NRF2 and PGAM5. **F** KYSE450 cells expressing HA-tagged PLEC^WT^, PLEC^ΔEQGE^, or PLEC^EQGA^, were subjected to anti-HA immunoprecipitation (IP); the co-precipitating KEAP1 was examined by immunoblot analyses (IB). **G** KYSE450 cells expressing HA-tagged PLEC^WT^, PLEC^ΔEQGE^, or PLEC^EQGA^, were subjected to anti-NRF2 immunoprecipitation (IP); the co-precipitating ubiquitin was examined by immunoblot analyses (IB). KYSE450 cells stably expressing a control shRNA (shC) or shRNA specific for p63 (shp63 #1) were infected with lentivirus expressing PLECs (wild-type and two mutants) or empty vector (EV), treated with irradiation, then subjected to immunoblot analyses (**H**), FACS assays (**I**, left panel), or cell viability testing (**I**, right panel). Results are presented as means ± SD from three independent experiments in triplicates. **P < 0.01.

The regulation of NRF2 protein relies on the transcription activity of ΔNp63α. Given that knockdown of ΔNp63α does not affect the expression of KEAP1 and Cul3, we speculated that ΔNp63α transactivates a gene that regulates the KEAP1-NRF2 interaction. To test this hypothesis, we performed an immunoprecipitation assay using anti-KEAP1 antibody in ΔNp63α-knockdown KYSE450 cells. The immunoprecipitates were identified by mass spectrometry (MS) analysis. Combined MS analysis with ChIP-seq analysis, we found showed PLEC is a KEAP1-associated protein and may be transactivated by ΔNp63α (Supplementary Table 1). This interaction was validated in KYSE450 cells by co-immunoprecipitation analyses using anti-KEAP1 antibody or anti-PLEC antibody (Fig. 3A). Immunofluorescence assays showed that KEAP1 and PLEC mainly co-localized in cytoplasm (Fig. 3B). To determine whether an increase in PLEC levels might decrease the interaction between KEAP1 and NRF2, Flag-KEAP1, HA-PLEC and NRF2 were co-transfected into HEK-293T cells. Coimmunoprecipitation assays confirmed that an increase in PLEC competitively inhibited the KEAP1-NRF2 interaction and stabilized the protein level of NRF2 (Fig. 3C, D). Previous studies have demonstrated that KEAP1-associated proteins (e.g., NRF2, PGAM5 and Nestin) contain a consensus E (S/T) GE motif for binding to the Kelch domains of KEAP1 [22, 23]. Interestingly, the PLEC protein in different species share a highly conserved EQGE motif (Fig. 3E). We then constructed an EQGE-deletion vector (PLEC^ΔEQGE^) and a missense mutant vector (PLEC^EQGA^) in which EQGE^1863^ was changed to ESGA^1863^. As shown in Fig. 3F, both PLEC mutants were unable to associate with KEAP1. Immunoprecipitation assays showed that the levels of ubiquitin-conjugated NRF2 were increased in PLEC^ΔEQGE^ and PLEC^EQGA^ cells (Fig. 3G), suggesting that the EQGE motif of PLEC is essential for its competitive binding to KEAP1. Furthermore, only wild-type PLEC, not its mutants, in ΔNp63α-knockdown KYSE450 cells was able to rescue the expression of NRF2 (Fig. 3H), the antioxidant capacity and cell survival (Fig. 3I). Taken together, these results demonstrate that ΔNp63α inhibits the interaction of KEAP1 with NRF2 via PLEC, thereby protecting protects NRF2 from degradation.

### ΔNp63α directly transactivates PLEC expression

**Fig. 4:**
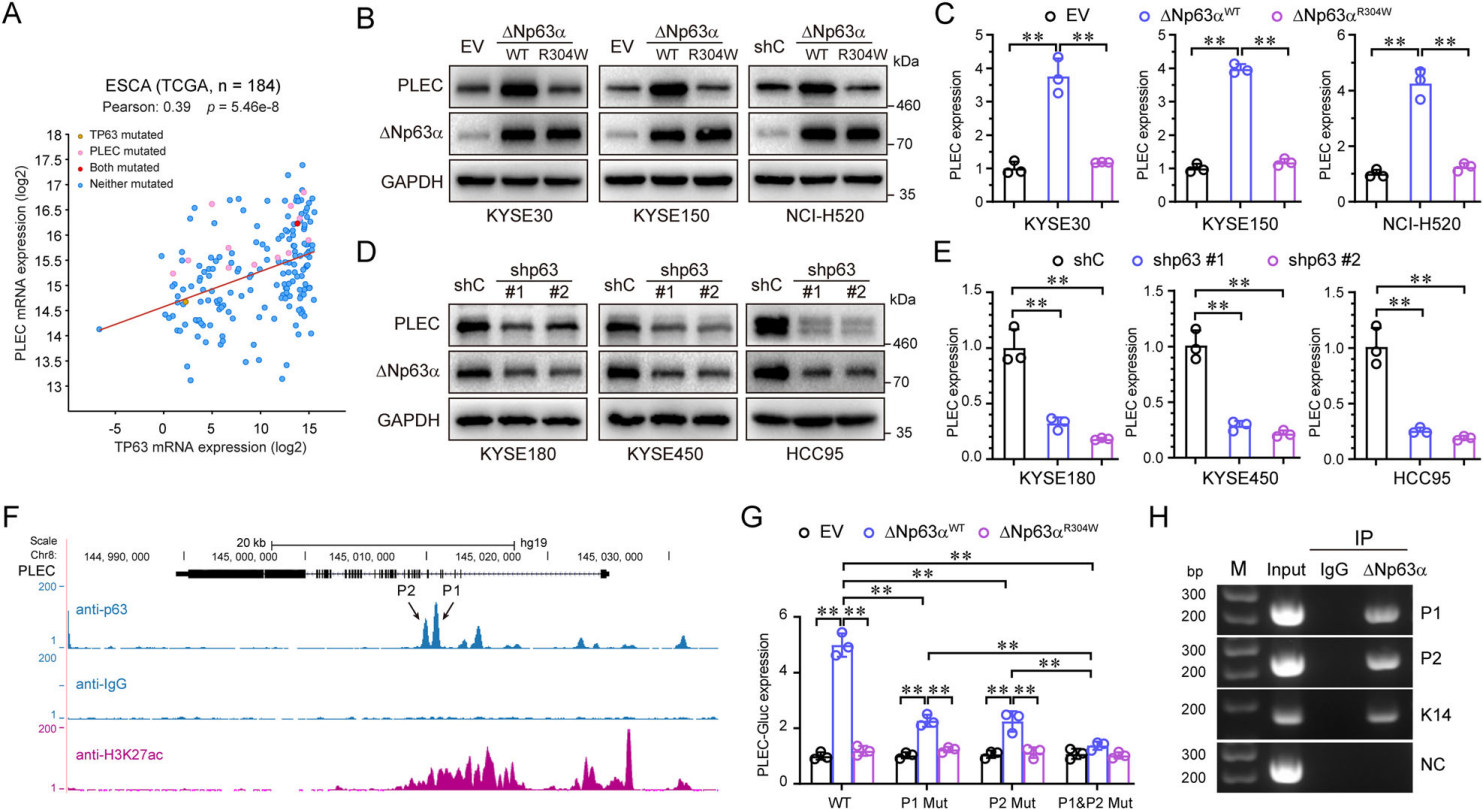
ΔNp63α directly transactivates the expression of PLEC. **A** The Pearson correlation coefficient (R value) and a two-tail probability test (P value) between TP63 and PLEC were analyzed based on TCGA database. KYSE30, KYSE150 or NCI-H520 cells stably expressing ΔNp63α (WT or R304W) were subjected to immunoblot analyses (**B**) and QPCR assays (**C**). KYSE180, KYSE450 or HCC95 cells stably expressing a control shRNA (shC) or two different shRNAs specific for p63 were subjected to immunoblot analyses (**D**) and QPCR assays (**E**). **F** TP63 chromatin immunoprecipitation (ChIP) sequencing data from human keratinocytes (GEO accession number GSE32061) identifies the putative TP63-binding sites within PLEC enhancer. **G** HEK-293T cells were co-transfected with PLEC-Gluc-SEAP reporter (WT, P1 Mut, P2 Mut or P1&P2 Mut) and ΔNp63α (WT or R304W) expression plasmid. PLEC-Gluc and SEAP activities in media were measured at 36 h post-transfection. **H** ChIP assays using indicated antibodies or a normal rabbit IgG were performed in KYSE450 cells. Primers specific for P1, P2, or NC (negative control) were used. The K14 was used as a positive control. Results are presented as means ± SD from three independent experiments in triplicates. **P < 0.01.

**Fig. 5:**
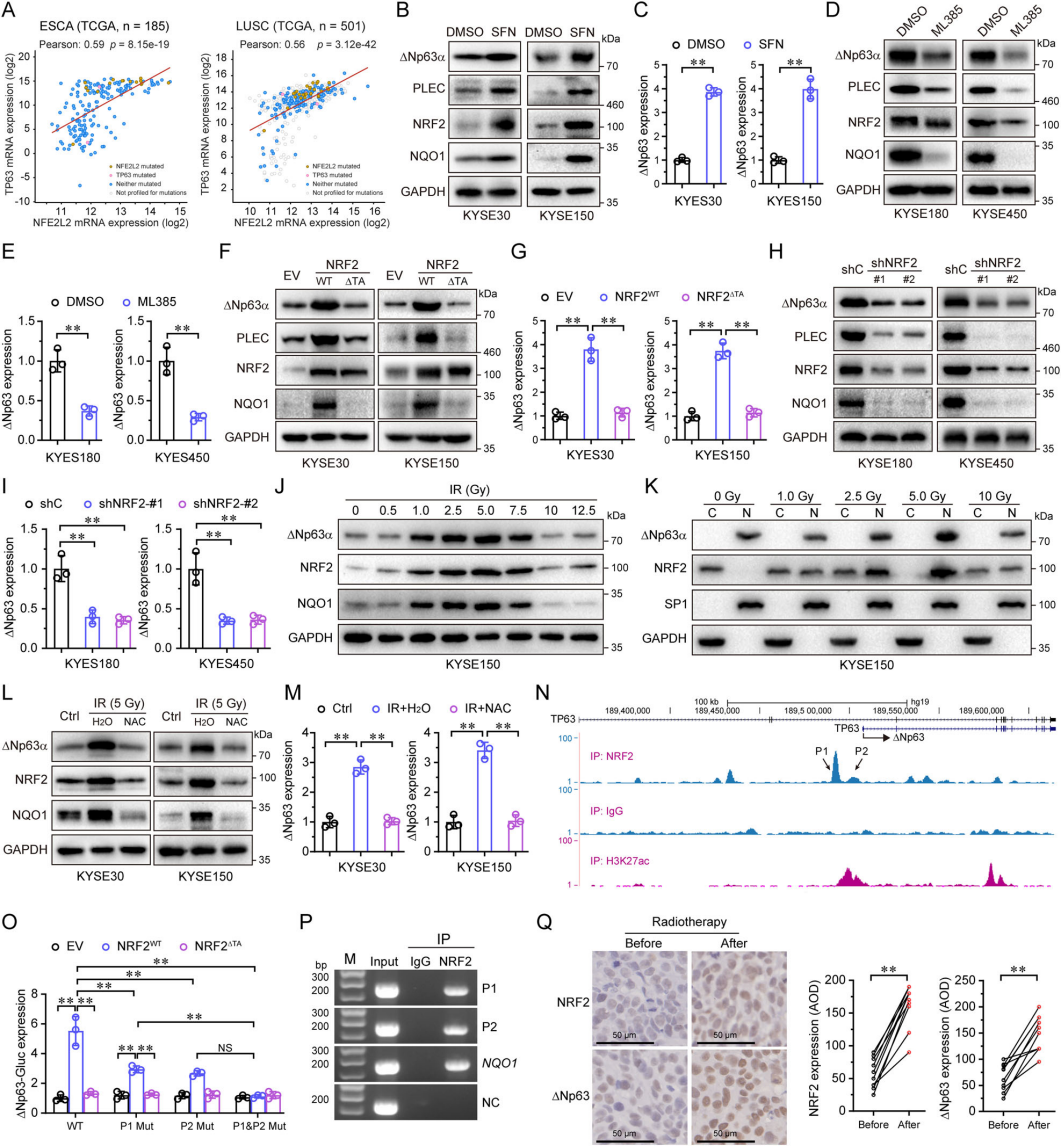
NRF2 directly transactivates the expression of ΔNp63α. **A** The Pearson correlation coefficient (R value) and a two-tail probability test (P value) between NRF2 (NFE2L2) and TP63 were analyzed based on TCGA database. KYSE30 or KYSE150 cells were treated with SFN (5 μM) or DMSO for 48 h, then subjected to immunoblot analyses (**B**) and QPCR assays (**C**). KYSE180 or KYSE450 cells were treated with ML385 (5 μM) or DMSO for 48 h, then subjected to immunoblot analyses (**D**) and QPCR assays (**E**). KYSE30 or KYSE150 cells stably expressing NRF2 (WT or ΔTA) were subjected to immunoblot analyses (**F**) and QPCR assays (**G**). KYSE180, or KYSE450 cells stably expressing a control shRNA (shC) or two different shRNAs specific for NRF2 were subjected to immunoblot analyses (**H**) and QPCR assays (**I**). KYSE150 cells were treated by irradiation with indicated doses, then subjected to immunoblot analyses (**J**) and cell fractionation analyses (C: cytoplasm, N: nucleus) (**K**). KYSE30, or KYSE150 cells were treated with irradiation, then treated with NAC for 24 h, followed by immunoblot analyses (**L**) and QPCR analyses (**M**). **N** NRF2 chromatin immunoprecipitation (ChIP) sequencing data from A549 cells (GEO accession number GSM2423706) identifies two putative TP63-binding sites within ΔNp63 promoter. **O** HEK-293T cells were co-transfected with ΔNp63-Gluc-SEAP reporter (WT, P1 Mut, P2 Mut or P1&P2 Mut) and NRF2 (WT or ΔNhe2) expression plasmid. ΔNp63-Gluc and SEAP activities in the media were measured at 36 h post-transfection. **P** ChIP assays using indicated antibodies or a normal rabbit IgG was performed in KYSE450 cells. Primers specific for P1, P2, or NC (negative control) were used. The NQO1 was used as a positive control. **Q** Representative IHC images and protein expression quantification of NRF2 and ΔNp63 in 10 patients with ESCC before and after receiving radiotherapy. Results are presented as means ± SD from three independent experiments in triplicates. **P < 0.01.

To elucidate the molecular mechanism by which ΔNp63α regulates PLEC expression, we analyzed the correlation between ΔNp63α mRNA levels and PLEC mRNA expression by using TCGA database. As shown in Fig. 4A, ΔNp63α (TP63) mRNA levels positively correlated with the PLEC mRNA level in ESCA. Ectopic expression of wild-type ΔNp63α, but not the transaction defective mutant (R304W), significantly upregulated both mRNA and protein levels of PLEC (Fig. 4B, C). Conversely, knockdown of ΔNp63α resulted in a significant decrease in both mRNA and protein levels of PLEC (Fig. 4D, E). Chromatin immunoprecipitation of ΔNp63α combined with massive parallel sequencing (ChIP-seq) in human keratinocytes has revealed more than 7, 500 putative ΔNp63α-binding sites [24]. To determine if ΔNp63α had putative binding sites within the gene regulatory sequences of *PLEC*, we examined enriched sequences near the *PLEC* genomic locus using the UCSD genome browser (Fig. 4F). We next analyzed the human *PLEC* genomic sequence for transcription factor-binding sequences and found two conserved putative ΔNp63α-binding sites, termed P1 and P2 (Supplementary Table 2). Luciferase reporter assays showed that ectopic expression of ΔNp63α, but not the transactivation defective mutant (R304W), promoted the reporter activity of ΔNp63α-Gluc-WT (Fig. 5G). The reporter maintained the activity when P1 or P2 site was mutated (P1 Mut or P2 Mut) alone, but mutating both sites (P1 & P2 mut) abolished reporter activity, suggesting that both P1 and P2 sites are important for *PLEC* gene transcription. Furthermore, ChIP assays demonstrated that ΔNp63αdirectly binds to the P1 and P2 sites, with K14 as the positive control (Fig. 4H). Taken together, these results demonstrate that PLEC is a target gene of ΔNp63α, and that ΔNp63α directly transactivates PLEC expression.

### ROS-activated NRF2 promotes the transcription of ΔNp63α

Our data suggest that ΔNp63α help maintain the redox balance by regulating the antioxidant capacity in ESCC cells. We previously found that oxidative stress could enhance ΔNp63α expression [25]. However, it is unclear how intracellular oxidative status influenced ΔNp63α expression. To explore factors mediating ΔNp63α transcription, we analyzed the correlation between ΔNp63α mRNA levels and other genes expression using TCGA database. As shown in Fig. 5A, ΔNp63α (TP63) mRNA levels positively correlated with NRF2 (NFE2L2) mRNA levels in ESCA and lung squamous cell carcinoma (LUSC). Thus, we speculated oxidative stress activates ΔNp63α expression via NRF2. Activation of NRF2 by sulforaphane (SFN) significantly upregulated the protein levels of NQO1, a well-established target of NRF2, as well as ΔNp63α and PLEC in KYSE30 or KYSE150 cells (Fig. 5B). SFN also dramatically increased ΔNp63α mRNA levels (Fig. 5C). Conversely, inhibition of NRF2 by ML385 significantly downregulated both mRNA and protein levels of ΔNp63α (Fig. 5D, E). Furthermore, ectopic expression of wildtype NRF2, but not the transactivation defective mutant (ΔTA), significantly upregulated ΔNp63α mRNA and protein levels (Fig. 5F, G). Knockdown of NRF2 resulted in a significant decrease in ΔNp63α mRNA and protein levels (Fig. 5H, I). ΔNp63α protein levels increased with higher irradiation doses from 0 Gy to 5 Gy (Fig. 5J), accompanied with NRF2 protein translocated from the cytoplasm to the nucleus (Fig. 5K). Clearance of ROS by NAC significantly decreased ΔNp63α expression, which was upregulated by irradiation (Fig. 5L, M). ChIP-seq analysis in A549 cells has revealed numerous putative NRF2-binding sites [26]. To determine if NRF2 had putative binding sites within ΔNp63 gene regulatory sequences of *ΔNp63*, we examined enriched sequences near the *ΔNp63* genomic locus using the UCSD genome browser (Fig. 5N). We found two conserved AREs (5’-RTG AYnnnGCR-3’) in the human *ΔNp63* gene promoter, termed P1 and P2 (Supplementary Table 3). Luciferase reporter assays showed that ectopic expression of NRF2, but not the transactivation defective mutant (ΔTA), promoted the reporter activity of ΔNp63 -Gluc-WT (Fig. 5O). The reporter-maintained activity when either the P1 or P2 site was mutated alone (P1 Mut or P2 Mut), but mutating both sites (P1 & P2 mut) abolished the reporter activity, suggesting that both P1 and P2 sites are important for ΔNp63α transcription. ChIP assays confirmed that NRF2 binds to both the P1 and P2 sites, with NQO1 as a positive control (Fig. 5P). Furthermore, in 10 patients with advanced ESCC treated with radiotherapy, we found that the expression of ΔNp63 and NRF2 were significantly higher after radiotherapy than that before treatment (Fig. 5Q). Taken together, these results demonstrate that ΔNp63α is a target gene of NRF2 and that NRF2 promotes the transcription and expression of ΔNp63α via a positive feedback loop.

### Targeting NRF2 inhibits ΔNp63α-mediated radioresistance

**Fig. 6:**
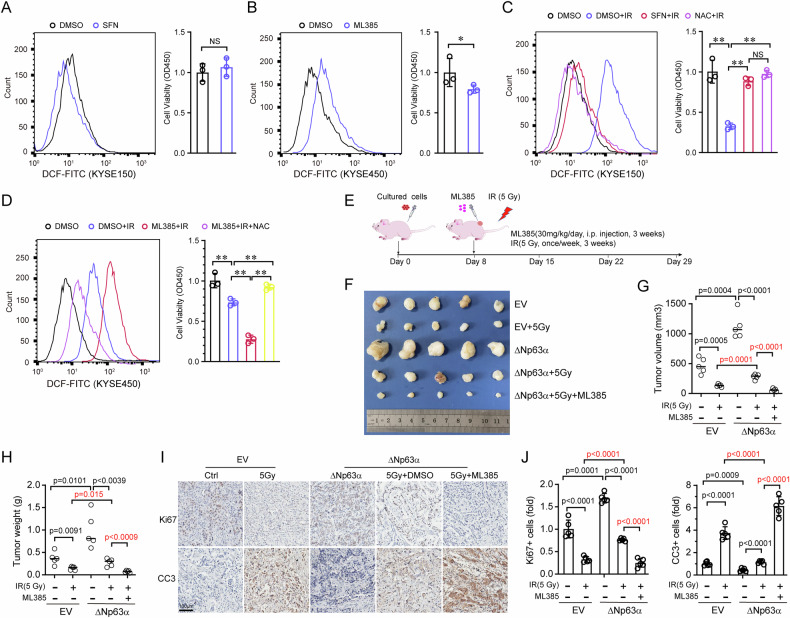
Inhibition of NRF2 overcomes ΔNp63α-mediated radioresistance. **A** KYSE150 cells were treated with SFN (5 μM) or DMSO for 48 h and then analyzed by FACS (left panel) to assess ROS and cell viability assays (right panel). **B** KYSE450 cells were treated with ML385 (5 μM) or DMSO for 48 h and subjected to FACS (left panel) and cell viability assays (right panel). **C** KYSE150 cells were treated with or without IR (10 Gy), then combined with SFN (5 μM) or NAC (1 mM) as indicated for 48 h, followed by FACS (left panel) and cell viability assays (right panel). **D** KYSE450 cells were treated with or without IR (10 Gy), then combined with ML385 (5 μM) or/and NAC (1 mM) as indicated for 48 h, followed by FACS assays (left panel) and cell viability assays (right panel). **E** Schematic description of the animal experimental design. **F** Representative images of dissected xenografts from the indicated groups at the end of the experiments. **G**, **H** Tumor weight of the subcutaneous xenografts and tumor volume in the indicated groups. **I** Representative immunohistochemistry images of Ki67 and Cleaved caspase 3 (CC3) in the indicated group. **J** The protein levels of Ki67 and CC3 were quantified by average optical density (AOD).

To investigate effect of activation or inhibition NRF2 on ROS levels and cell viability, KYSE150 or KYSE450 cells were treated with SFN or ML385, respectively. As shown in Fig. 6A, activation of NRF2 by SFN obviously reduced ROS level, although this did not lead to a marked improvement in cell viability. In contrast, inhibition of NRF2 by ML385 caused a substantial increase in ROS levels and a significant reduction in cell viability (Fig. 6B). These results suggest that low endogenous ROS levels may not significantly impact cell viability. However, following irradiation treatment, cell viability was markedly reduced as ROS levels increased in KYSE150 cells (Fig. 6C). Treatment with either SFN or NAC effectively neutralized irradiation-induced ROS and significantly improved cell viability. Conversely, inhibition of NRF2 by ML385 further elevated irradiation-induced ROS, enhancing the irradiation-mediated reduction in cell viability (Fig. 6D). Notably, NAC treatment almost completely restored cell viability following irradiation. These findings underscore the critical role of NRF2 in maintaining ROS levels and supporting cell viability.

To examine the effects of ΔNp63α and NRF2 on ESCC progression, we generated xenograft models by subcutaneously injecting transfected ESCC cells into nude mice, followed by treatment with irradiation and ML385, an inhibitor of NRF2 (Fig. 6E). As shown in Fig. 6F-H, ectopic expression of ΔNp63α could enhance the tumor growth and weight. Irradiation significantly reduced tumor growth and weight, whereas overexpression of ΔNp63α promoted tumor growth after irradiation. Irradiation combined with NRF2 inhibition significantly suppressed ΔNp63α-mediated tumor growth, indicating that NRF2 inhibition can counteract ΔNp63α-mediated radioresistance. Furthermore, immunohistochemistry assays showed that ΔNp63α increased Ki67+ cells and decreased Cleaved Caspase-3+ (CC3) apoptotic cells compared with the empty vector (EV) group after irradiation treatment (Fig. 6I, J). However, inhibition of NRF2 combined with irradiation significantly reduced Ki67+ cells and increased CC3+ apoptotic cells. These results indicate that NRF2 inhibition of NRF2 can counteract ΔNp63α-mediated radioresistance in ESCC.

## Discussion

Radiotherapy is a major treatment option for unresectable or locally advanced ESCC. However, it has been reported that the overall survival of patients with advanced ESCC has not improve significantly [2, 27]. Notably, overexpression of ΔNp63α is associated with radiation resistance in oral squamous cell carcinoma [28]. In this study, we revealed ΔNp63α promotes radioresistance in ESCC by regulating the protein stability of NRF2. We systematically identified that ΔNp63α directly activates the transcription of PLEC, which binds to KEAP1, resulting in the dissociation of NRF2 from KEAP1. Consequently, NRF2 is stabilized and translocated to the nucleus, and activates target genes responsible for cytoprotection, conferring resistance to chemotherapy, radiotherapy, and immunotherapy [29]. Therefore, targeting NRF2 is critical for overcoming the therapy resistance. Most importantly, pharmacologic inhibition of NRF2 using ML385 markedly attenuated ΔNp63α-mediated radioresistance and enhanced the antitumor activity of radiotherapy in nude mice.

ΔNp63α, the predominant p63 isoform, is critical for squamous epithelial development, and is frequently amplified and overexpressed in ESCC. ΔNp63α is at the heart of cancer stem cells (CSCs)-related signaling [30]. It is believed that CSCs are the principal causes of mortality and therapy failure due to radioresistance [31], suggesting that elimination of CSCs is essential for overcoming radioresistance and improving ESCC treatment. Recently, it has been demonstrated that ΔNp63α transactivates RSK4 to promote CSCs properties and radioresistance in ESCC through direct phosphorylation of GSK-3β at Ser9, consequently activating of the β-catenin signaling pathway [32]. One study has shown that breast and liver cancer stem cells tend to have low ROS levels owing to the expression of ROS-scavenging systems [33], implying that ΔNp63α might be involved in ROS-scavenging. CSCs are a small population of cells of solid tumors [34]; therefore, it is unlikely that all ΔNp63α positive ESCC cells are CSCs. It has been reported that ΔNp63α plays an important role in drug resistance by increasing the transcription of AKT1, EGFR, 14-3-3δ, WIP1, or by downregulating CD95, BAX [30]. Here, we found ΔNp63α promotes radioresistance in ESCC through the regulation of NRF2-ROS signaling. We identified that ΔNp63α stabilizes the protein level of NRF2, rather than its transcription level, which is consistent with the previous report [35].

**Fig. 7:**
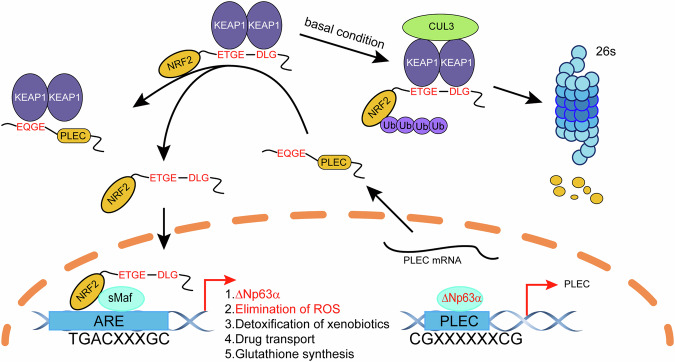
A model illustrates how the ΔNp63α-PLEC-NRF2 axis promotes radioresistance by reducing radiotherapy-induced ROS. Our study demonstrates that ΔNp63α, which is overexpressed in ESCC, drives the transcriptional activation of PLEC. PLEC, in turn, binds competitively to KEAP1, preventing NRF2 from undergoing ubiquitin–proteasome degradation. This stabilization facilitates the nuclear translocation of NRF2, which enhances antioxidant capacity and promotes radioresistance. Additionally, low-dose irradiation activates NRF2, which subsequently upregulates ΔNp63α expression. Collectively, these findings suggest that ΔNp63α and NRF2 participate in a positive feedback loop, amplifying antioxidant responses and helping maintain cellular redox homeostasis, which ultimately contributes to enhanced radioresistance in ESCC.

The expression of ΔNp63α is tightly regulated at transcriptional, post-transcriptional, translational, and post-translational levels. Our previous work demonstrated that ΔNp63α is transcriptionally inhibited by several oncogenic signals [18]. More recently, we revealed that hypoxia-activated XBP1s suppressed ΔNp63α expression at the epigenetic level [16]. ΔNp63α is an oncogene and plays a crucial role in ESCC development. ROS also plays an important role in the initiation and progression at low to moderate levels. NRF2 is the key transcription factor that responds to ROS and activates several oncogenes, such as KLF9, p62/SQSTM1, ATF3, and Nestin. Therefore, we hypothesized that the oncogene ΔNp63αcould also be regulated by NRF2 activation. Indeed, we identified that ROS-activated NRF2 directly binds to the promoter of *ΔNp63* and triggers the expression of ΔNp63α. These findings expand our understanding of the mechanism through which NRF2 activation modulates ΔNp63α. Notably, we observed that ΔNp63α levels rose progressively from 0.5 Gy to 5.0 Gy, then decreased from 7.5 Gy to 12.5 Gy, returning to baseline levels after 10 Gy (Fig. 5J), indicate that low-does irradiation or ROS can elevate the expression of ΔNp63α. Collectively, we demonstrate a positive feedback loop between ΔNp63α and NRF2, which is responsible for mediating antioxidant responses and maintaining cellular redox homeostasis in ESCC (Fig. 7).

NRF2, the master regulator of cellular antioxidant responses and a cytoprotective protein, is regulated by a finely tuned control system. The homodimer of KEAP1, the adapter protein for the E3-ubiquitin ligase complex, binds NRF2 and promotes its ubiquitination and degradation in the absence of oxidative stress. Therefore, the NRF2 protein level might be affected by either disruption of KEAP1-NRF2 complex stability or reduced expression of KEAP1. It has been reported that p62/SQSTM1 and Gankyrin can bind to KEAP1 and protect NRF2 from degradation. Additionally, p21 and CDK20 reportedly contribute to the upstream regulation of the NRF2-KEAP1 pathway. Moreover, USP15 stabilizes the KEAP1-NRF2 complex and promotes the degradation of NRF2. Consistent with these findings, we show that ΔNp63α can facilitate the stabilization of NRF2 by activating the transcription of PLEC, which competitively binds with KEAP1 and consequently releases NRF2.

Increasing evidence indicates that two separate sequences, the E (S/T) GE and DLG motifs, are responsible for the interaction with KEAP1. Recent reports have shown that some antioxidant proteins, including PGAM5 and Nestin, can compete with NRF2 for binding to KEAP1 by suppressing the binding of the DLG or E (S/T) GE motif. For instance, the p62/SQSTM1 protein binds to KEAP1 through the sequence STGE, which resembles the ETGE sequence used by NRF2 to interact with KEAP1. Interestingly, we identified EQGE motifs in the PLEC protein through sequence analysis. In this study, we found that this high-affinity motif was responsible for the interaction with KEAP1, and that deletion or missense mutation of the EQGE motif in PLEC reversed its ability to release NRF2 from the inactivating complex, thereby increasing the proteasomal degradation of NRF2. Additionally, other research has found that somatic mutations and gene variations in KEAP1 frequently occur in lung cancers and cancer-derived cell lines [36], suggesting that mutation KEAP1 might disrupt its ability to bind NRF2.

Plectin (PLEC), a ubiquitously present cytolinker protein in several cell types (epithelia, muscles, and fibroblasts), plays an essential role in the hemidesmosome by connecting keratin filaments to the underlying integrin α6β4 [37]. PLEC deficiency in the skin results in intraepidermal skin cleavage in basal keratinocytes. Mutations in the *PLEC* gene cause basal epidermolysis bullosa simplex (EBS) in 8% of cases. Interestingly, ΔNp63α is the master regulator that plays an essential role in epidermal differentiation [5]. Therefore, there might be a connection between ΔNp63α and PLEC. In this study, we identified that ΔNp63α directly activates the transcription of PLEC. PLEC comprises N- and C-terminal domains, which include multiple protein-protein interaction sites, linked by an elongated central rod domain. We found that PLEC competitively binds to KEAP1, resulted in the release of NRF2 from the NRF2-KEAP1 complex, and consequently stabilizing NRF2 to maintain cellular redox homeostasis. These findings expand our understanding of the mechanism of NRF2 activation.

Taken together, our findings suggest that the ΔNp63α/PLEC/NRF2 signaling pathway and antioxidant defenses could be targeted as promising therapeutic approaches for cancer treatment.

## Supplementary information

Supplementary Figures

Supplementary Table 1

Supplementary Table 2

Supplementary Table 3

Supplementary Table 4

Original Western blot

## Data Availability

The data analyzed during this study are included in this published article and the supplemental data files.
