# Supplementary material for: ΔNp63α promotes radioresistance in esophageal squamous cell carcinoma through the PLEC-KEAP1-NRF2 feedback loop
Source: Cell Death Dis. 2024 Nov 5;15(11):793. doi: 10.1038/s41419-024-07194-4 (PMC11538512; doi:10.1038/s41419-024-07194-4)
Supplement: Supplementary file 1 — Supplementary Figures [file 41419_2024_7194_MOESM1_ESM.docx]

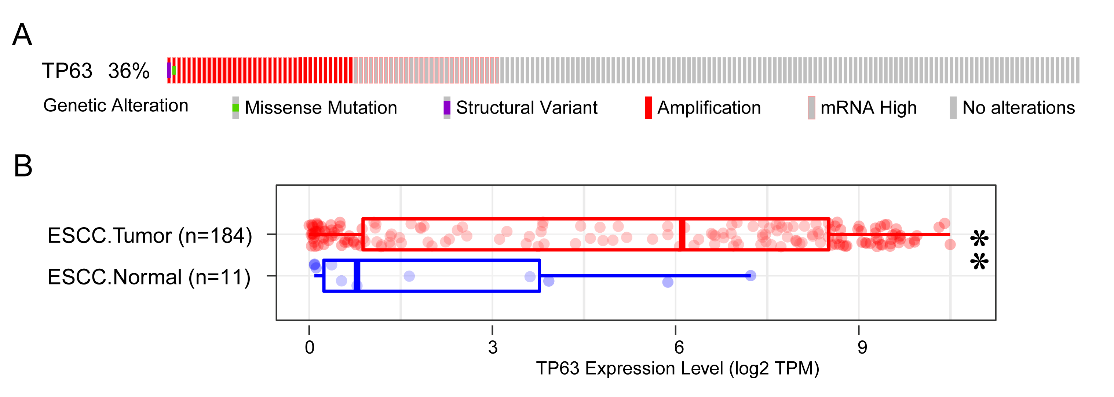


**Supplementary Figure 1**

(A) Genomic alteration frequency of *TP63* gene in ESCC. (B) mRNA expression of *TP63* gene in ESCC tumor and normal tissue.


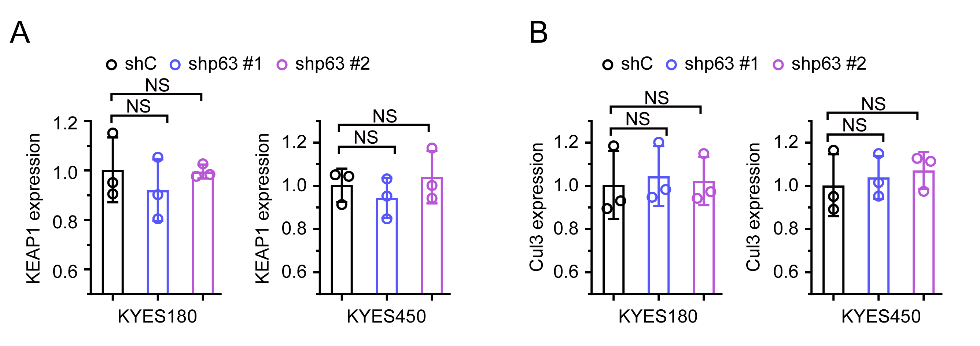


**Supplementary Figure 2** (Related to Figure 2A)

(A-B) KYSE180 or KYSE450 cells stably expressing a control shRNA (shC) or two different shRNAs specific for p63 were subjected to immunoblot analyses, then quantified the expression of KEAP1 (A) and Cul3 (B). Results are presented as means ± SD from three independent experiments in triplicates. **P < 0.01.
