# Supplementary material for: ΔNp63α promotes radioresistance in esophageal squamous cell carcinoma through the PLEC-KEAP1-NRF2 feedback loop
Source: Cell Death Dis. 2024 Nov 5;15(11):793. doi: 10.1038/s41419-024-07194-4 (PMC11538512; doi:10.1038/s41419-024-07194-4)
Supplement: Supplementary file 3 — Supplementary Table 2 [file 41419_2024_7194_MOESM3_ESM.rtf]

Supplementary Table 2 The consensus p63-binding sites in the PLEC promoter.
TTTGCCATGTTGGTCAGGCTGGTCTCGAACTCCTGACCTCAAGTGATCCACCCACCTTGGCCTCCCAAAGTGCCAGGATTACAGGCGTGAGCCACTGCACCCGGCCGACTTTTAAATTTATATTGCCCTTTACAATTAGAGGTATTTTGCAACTGTAATTTGATGTTGGTATGAACTTCCATGTTCTCGTGTGTCCTAATATGCCCTTATTTGTCCTCATTCTTCATTACTAGTTTGGCTGGATATAGTGTTCAGGTTCAAAATATGTTTTTCCTCAGATATTTGAAGACATTGCTGTTTTTCTTCATTCTTAAAAGAATATTTTTCTTGGAAAGTTAAAAAATATCCCCCCATGGTTTCTTAGCATCTGGTGTCGGTAATGAGAGACCCAATGTGAGAAGGTGAGACTTCTTCCGGTTTGCATATTGACCTTGTTGTGTGTGTGTGTAAACTTGCAGGGTTGGCCAGCTGCGGTGGCTCACGCCTGTAATCCCAGCACTTTGGGAGGCTGAGGCGGGCGGATCACGAGGTCAGGAGATCGAGACCATCCTGGCTAACACAGTGAAACCCCTTCTCTACTAAAAATACAAAAAAGATAGCCGGGCGCGGTGGCAGGTGCCTGTAGTCCCAGCTATTCGGGAGGCTGAGGCAGGAGAATGGCGTGAACCCGGGAGGCGGAGCTTGCAGTGAGCCGAGATCGTGCCACTGCACTCCAGCCTGGGCGACACAGCCAGACTCCCTCTCAAAGAAAAAAAAAAAAAAAAACTTGCAGGGTTTTCTTTCACTCTGAAGGTTGAGGTTTCCTGGCACGTGGGTGCCAGGGGCGTTGTCTTTCCTCATTGTCCTTCTTGGCATTTCACGGATCTTGTGTATCTGAAGATGTTCCTTCAGCTCAGGGAAGCGTCTTCCCTGTGTGTGCTGGAGCTCCGGCCGGTGGAGCTCCTGCTATGCCGGCTCAGACCTCAGGCCCCCTCACCTGCTCCCTCGGAGTTCCTTTCTCTTCCGTTAGCTTCTCACTCTGCATCAGTTCCTGAACTCCATCTAGAACGCTAGCTGGGTCTTCAGTTTGAACCACTTCCTCATTTGGCTCACCCCCTCTAGATTTAGCTCAGCGTCCCTTTCTGAATGCAGGAGCACTCTGGTTTTCAGACTAGTTCCTTTTCATAGCAGCTAGTTTTGTTTAATAGACATGGTGTTCTTTTGCAACTCTCTGAAGAAACTATTTAAAATTTCTTTAAAAGTGTTTCCCAGGGCTGTCAGGTTGGTCACCTGAGGCCTTCTCTCCCACCTGGGTCTTTCAGCACTGAGAGGTCTGTGGCATCTCCTCGTGTTGGAGTCAAGTCCTGTAGGCAGCATAGGGCCCTGGCTCAGCTTTTCTCTGCAGAGGCCTCGCTTGAGTGGGTGGGGTTTGCCCGCCCGCAGACCTCCACGGGAGGGGGAGGGGTCAGGCCTCCCCAGCGGCCCTCTGAAGTCACTTGCTTCACGGAGGTGTTACTGTCTGCTGCTGGACAGAGCATGATGGGGGCTGCAAGGGCTCCCTCAAACCCTGGACTCCTCCAACAGAGGGCTCCTGGTTGCCAGGCTCAGCTCTGCCCTGCGTCGGCCCCAGGGCGTAGGGAGGGTGTTTAATCCTGGCCCGGGCCTTCCCCGCAGGTGGAGCGCGTGTCGCACCCGCTGCTGCAGCAGCAGTATGAGCTGTACCGGGAGCGCCTGCTGCAGCGATGCGAGCGGCGCCCGGTGGAGCAGGTGCTGTACCACGGCACGACGGCACCGGCAGTGCCTGACATCTGCGCCCACGGCTTCAACCGCAGCTTCTGCGGCCGCAACGGTGAGGCCTGAGCCGGGCAGGGGCGGGGGCTCTGCGCACCCCGACCACCCTGACCTTGCCCCGTCTCGCCCTGCAGCCACGGTCTACGGGAAGGGCGTGTATTTCGCCAGGCGCGCCTCCCTGTCGGTGCAGGACCGCTACTCGCCCCCCAACGCCGATGGCCATAAGGCGGTGTTCGTGGCACGGGTGCTGACTGGCGACTACGGGCAGGGCCGCCGCGGTCTGCGGGCGCCCCCTCTGCGGGGTCCTGGCCACGTGCTCCTGCGCTACGACAGCGCCGTGGACTGCATCTGCCAGCCCAGCATCTTCGTCATCTTCCACGACACCCAGGCGCTGCCCACCCACCTCATCACCTGCGAGCACGTGCCCCGCGCTTCCCCCGACGACCCCTCTGGGCTCCCGGGCCGCTCCCCAGACACTTAACCGAAGGGGCCACCCTCTGGCCTCCTGCTTCCCAGGCTCCCAGCTCCGCACAGGCTGATGCTCCCCGCCCCCAACTGTGGCCGCCTGAGCTGTCCCCGGGGACGCCCCTGCCTCCCTCTGCGGGCTCCAGAAGGCGGTGTGGGGGATGGCGGTCAGCAGCGGCCGAGGGGGGCCGGGCTAGGTCCCAGCCTGGGCCGACCCCACCACCAGGGGTCAGCAGAGCCCAGGAGGCGACACCGCCCGCCCGCCGCTCCCAGACCTCGCCCGAGTCGGCTCTGTTGTTTGAATAAACGTGAACGTGAACCCAGGCGGAAGGGACCCGGGAACTCTGACTGCGACCTCTGTTTGGGGAGTGGGGATCTCCGCGGCCCAGGGGCGGGATCTGCTGCTTCCGAGGAGGCGTCGGGGTGCGGGGCGGGGCCCGGAGCTCACCGGGGCGAGCGCACGACGTTCCCGTGTCGCGCACGCCGGCGCAGACAGCGTCTCCTTGACTACCGGCCCGCCCCGTGCGCCGGGGGCTCAGGTGTGCGCCGCTGCCCTGGCCGAATCGCCCGCCCGGGCCCCAGCCCCGCCGCTCCCGAGATCTCGGCGGCCCCGCGCAGACACGGGCGCCCGATCTCGCTGGCAGCGTTCGCTCGCTCTCGGCCGGGCCCGCGCGGCGCAGCTCTGGGTGGGGGCCGGGGCGGGGCTCGGGGCTCGCCCCGCCTGCGCCGCCTCCGCCGCTCTCCCGGCCCGAGCAGTCTGGCTGCGGCGGCAGAGCTGAAGTGAGCGGAGCCACCAGGCATGTATGACCGCGGCCAGGATGGAGAGGCGCGCCGCGTGCGGGCGCTGCGGGGTGGGGGTCGGGGGGCGGTCTCCGGCCGCAGGCCACACGGAACTAGGGGCTGCGCCGCGCCCCCTCCTGCCCTCGCTCGAGAGGACAGCACCTCCCTGCACGCCCGAGACTGCGGAACTGGACGGGCGGTGCCCGGGGTGTGGGCAGGAAAGGCCGCTGGGGAGGCGCGGCTGACCGCAGGGCTCCGGGCGGGGGGCTGCAACCGGGGCGGCCCGTGGAGAAGCCAGTGGGGGCGGGGCGCGGCGGCCCCCTCCTCGGTGTCCGCTGCCTCCTCCTTAGCCCTTGGCCTGAGGGCGGAGGCACAGAGCCCACATTGTGCCCCCGCGCCAGCCCACCCCCTCCGAGACAGAGCCCCATTCACCTCCCGCACGCCAGTGCTCTTGGGGACGGGGGCTGGGGAGTGTGCGCGCGTGGGTATGCGTGCGTCCTGGTTGCGTCCGCGGTGCCGGTGTACGCGCCCCCACTGTGGGCCGCTGGAGCTGCGGCCCGGGCTCCCTCCCGGCCTCGCTGCAGCAGAGCTCCCGCGGCCCACGCCGCTGCGGCTGCAGCTATTTATAGGCCTTCGCTGCGGAGATCGAGGTGGGACTGCGGGCTGAGGTGGGGTGGCGTGATGGGGAGTCCTAGAGGTCGAGAGAGCTTGGAGGTGGACTGCCGGTGTCCAGGGGAAGGGCTTGGGTGTCCTTCCCTGATGACCCGGTGAGTGGAGGGGGCGGTGGTCAGCTTTCCTTGATGGCCTCGCCTGCCTGCGATTAGAGGGTTAGAGGCGGGTCCAGTTTGGGATGGAGGCAGTGGAGGCCGGCATCTGAGTGAAGGTAGTCACCCCTTCCCCTTCCATTTGACAGAGTGGAAGGTGGTCTGTCGTGTGCCGGTTGAGCCCCTGGGTGACAGAGTAGGTGGGTGGGGTGTGGGTGACTAAGAGAGGAGAGTGGCAGTCAGTGTGTGGGAGTGGGGGTGGCCAGGGTGGTCAGTGTCCTGGTGATGGACTGGAGGGTAAGGCCAGTGGCTGGGGGGTCACTGTGTAGGTATCTGAGGAGTGCGCGGGAGGGGGCAAGGCAGTCATGGATGGAGAGAGGGTAAGTCAGTGCCTGGGGCCAGAGCATGGGGGTCAATATGTGGGTACAGCAGAGTGCAGGAGGGGTGTGGGTGCCAGTGTCCAGGCGAGAGGCTGGGTGGGGAGAGTTCAGTGTCTAGGTTAAGAAGGTGGTGGGTGGGTCGGCGTGCAGAGTCCACTCCTGAACAGGCAGAGATGTGACCCCTCCTGCTCCCCTTCCAGGAGGCCATGTCGGGTGAGGACGCTGAGGTCCGGGCAGTCTCTGAAGATGTCTCCAATGGAAGCAGTGGCTCGCCCAGCCCTGGGGACACACTGCCCTGGAACCTTGGGAAAACGCAGCGGAGCCGGCGCAGCGGGGGTGGCGCTGGGAGCAACGGGAGTGTCCTGGACCCAGCTGAGCGGGCGGTCATTCGCATCGCAGGTAACTGCGCAGGGCCTTGCCTGTGCACCTCCTTGGCACGCGAGGTTGACTGAGTTCCCGCTGCGTGCTAGGCGCTGGAGCGGGGACTGGGTGGGGGAGGCCTAGGGGGATCTGCGGGTCACGGAGGGCCTCGCGCGTCCCAGGTGAGTGCAGGCGGCACAGCGCCACCTGCCGGCCGCGCCCAGGCGGTTCATGAGGCCTGGGGTTGGGCTTCTTGCCGCGGCGTCCATCCTTGATTTTAACACGGTCCAGTGCCCAGATGCAACAAGGATAATTTTTTGGGTTCCCCACCAGGCCCTCGGTCTTGGCTGCCACAATGGCGGCCAGGTGGGAATGTACAGGAGCAGTGTATAGGGAGGGGGTACCTGGTTGTCTGGAGCCCCAGCCTCTTCCTGAGCCAGGATCTGGCCTTGCTTTCCAGTAGCTCCCCATCCAGGGAGAACGATGGGAAGCATCCAGCGAGATGCTTGGGGAAGGGGGAGGGCAGGAGGGAAGTGGGTGACCTAGAGCTGTGCTAGGAGAGGGAGGAGCAGTAGCTCTCTAGGCTTTGGCTTGAGAGTCCTGACCTGTTCCAGCCGGAGGGCAGGGAGGCCCCTGCGGGAGGCCAGGAGAGGGCTGCAAGGCCAGGTGCTGAGCCCTGGGGGGATGTTGCAGACGACGGGAAGCGGGGCTGGTAGGAGCAGGAAAGAGGGAAGGAGAGGGTCACTGCTGTGCACCCAGCTCCAGCCCCAGGGTCTGGTTCCATCTGCATGACCTTCAGGGCTTTTCACAGCACACACGGACTTTAAAGAAAGAAGTCACTTTCTTCCCACTTGAAAGGGAATCACTTGTGTCGTTCCCACGCCTGGGCTTTCTTCAGCATCACCTTTACAGTTGTGGAGGAGTGTGGGGGGTCCCGAGGCCCCGGCTGACCGTGGTTGCCTTCGGTACTCACTGCAAAGGCTTTGCCCCCAGCCAGCACCTTCCGCGCCGGGGCTCTGCGTCCTCTGACGGTTTCCTTAGGACCGATTCCTGGAAGCGGGACTTGCAGTTACAGGACATATGGCATTAAGCTTGGAGAAAACACGGCCAGGGGCATCCCAGACAGGGTGGCCGACATTGGTGAAGGCGGGGAGAGCGGGGGCTTTGCCAATCTGATGGGTGGAAAGAGTTGTTTTAATTATCACCAAGCATTTTTTCCTTCTGTGAATGGGCTATTCCAGTCCTCGTATGTGTACTTAATTTTTCTTGTGTGTAGTGTTTTAAGGGTTCTTTATACCGACTCAGTCGTCTCCAATGTTGCAATGGGAAAGTTCTCAGCTGCACGCAGGAAGCAAGGTGGGGGGCGGGCAGGGGAGCGTCTCGGAGGCAGGTCGCCGGCCCGGCGGGGAACAGACAGGTCGCGGCCGCCCCTGGGCGGGGGCCACCCGAGGACCCCCGGGCGACCGCGGTGTCGGCGGCCGCGACCCGGGCGAGAACGGGAAAGGGCGTGCGTGCGGGACTCGGAGCCCGCGGAACCGGCCCGCCGGGAGGGGCCCAGACGCGGCTCCCTCTGCCCGGCCGCGGCGGCCCCGGCCCCGCCCCGCCCGTGGGGCATCCTGGGTGCGGGGGCGGGGCGGGGGCGGGGCGGGGGCGGCCTCTTTAAGCGGCGCGCGCGGCCGCGGGGACAGAGTGGCCGCCGGGTGCTGGAGGCTCCGCTGCACCCCGCGCCCCGCGCCCGCCGGCATGGCCGGCCCGCTGCCCGACGAGCAGGACTTCATCCAGGCCTACGAGGAGGTGCGCGAGAAGTACAAAGGTACGGCCCCCCGTCCCGCTGCCTGTCGGGCCGCTCCTGACAGCCCAGCCGCCGAGCACCTTGTCGTCCACGGTGGCGGTCCCGATCGCCGGCCCGAGCGCGGCCACTCCGCCGGCCTCGTCCGCCTGGATCGCCCCCAACGGCTGCCAGTGCCTAGGGAAGGGGAGGACTCGCTCCGCTGAGCCCCCGAGGCCATGCCCTTAATAGGGCATCGCGCGGCTCGGGTGGGTGGGGTGGGCCGCGGGCGGGTGGGGGCGCTCAGGGCTTGTCGGGGTCGGCTGTCTGAGCCAGGATCCGGTGTCTGTGGCCAGCAGACACATGGGTCAAGGCTGAGCCATCCCCTGCCCCACCTCTGGGGCGCGTCTGCATGGAACAGGGGCAGCTCCGCCGCCTGGCAATGGGTCACATAGTTGCCGCCTCTCCTCACCTCCCTAAACTGGCGAGGGCCTGGGCTGCCACCCTTCCTTGGCAGGGCACCCCCCCCACACCCCTTCTCTGTTCACTAGGGGACAGCTGTGGGCTAGGGGTCAGGGTGGCCCCTCCTTCCTCTCTAGGGGAGCATCAGAGTGGCCTGCCCACCTGCTTGATGCCTCGGCAGACCCCCTTGCCCTAGTCCACGCCCACGGGGAGCCCCTGGACCCTCCCAGGTCCTGGTCCCACCGGCCATTCTTGCAGCCCAGCTGCCTGGCTCAGCCTGGGTCGCCCGGCTCCAGGCGGTTGTGGACACAGCATTGGCTGCCGGCCTGCAGAGGCTTAGGAGCAAGGGACTGAGCTGCTGGGGAGGGTCGCGGTGGGGGCCCCCAGGCAGAGCCGATCAGAGCAGACTCCAGCTCCACGTGGCTGGTTTTGCTGGCTCTGCCCCCAAGGGCAGCTCAGGGAGAGAGGTCCTGCTGGGGCCCCGTGGTGCCGCTCTCCACCACAACCTTGCCCAGCTGGGTAGGTTTCATGGTGCCCCGAACTCATCTCCTTCAGGCGTCCTCCAGCGACGGCTCTGGGGCAGGGTGGATGTACTGCTGTGGCTCTCTTTGACAGTGCTGTGCCCAGCCGCTTTGCCTGTGTGACTAGTTCATTCGGGCCAGGGGAGACACTCCTGCACCCTTGGGGAGGCCTGGAGCGGGCAGCCTGTTGCTCATGCTCTACATGGCCGGCCCTGCCTTAGAGGGAGCACCCGGAGGGGTGCTGGGGTATAGGGCAGGGAAGCAGCCGCCCACCTGGGCTCCAGCCCTCCTGGCAAGCCCCAGGACAGAGCTGTTTCTGAGCTGACCACTCTGTTCAAAGTGCGGGAACTGTGCAGGATCGTTTCCTCCTGGAGTTCTTCATCTGGTCAGGTTGCTGGGCTGCCTGGGGTGCCCCAGCCTGTTCTGGGCATTGTGCTGAGGCTTTCCTGGCCCCAGGGCTGTCTTTCCTCTACCTCCTGGGGTGGGAACTACTTCTGATTCATCCAGGGGTGAGCCAGACAGATCCCTGGAGGTTGCTGGGACCCTCTGTCAGGGTCCTCCCTCCCAAGCGCAGCTCCTCTGGAGGCCAGGATTGGTGTTGGGCACACTCGCAGTCTTGGCCCATTGTCTGCCTTCTGGGCTTGGTGGTGGAGGCTGGTGAGGGGGCAGGGGGGCCGGGGGGCTTGCCTGAAGTGGAGTTGTCACGTGATGGTGCAGCTGCCCGCATGTGTGTGGCACTGGCCTGAGAGTGGCTAAACAGCAAGGCAGGTGCTGAGGCTCCGTGAGCACACCAGGTACCCCAAGTCTCCTTAGCTCCTGGCTTGAGACAAGGGGACGGGAGGGGCTGGGCTGGTACCTGTGTCTGGGAGCCACGGACCAAGGCTGAGGAGGGCCTGCAGGAGGTCAGAGGTGGCCCCTGTGCCCTACACCTTTCCAGGCCTGACCTCTGTGAGCCCCTTGGACCTGCTAGAGGGCTGGAGCCCCGGCCCTTCCTGTCCCTGGGGTGGAGGGACTGTGGGAGGGCATGGCACCCCTTGCCAGCCAGCCAGTGACGGCAAGGCAGCCCCAAATCCCTCCATGGTAAGCAGCTGCGGGCCCCTGTCCTGGGCCCAGCTCAGCATTTGGTGGTTCTGAGGAGGGCAGGGAGGCCAGGCCTGTGCCCCCTGCCCAGCTGATCCCTGCACTGAGGAGGTGCTCTGGCTTAGGCCTCCTGGCCTCCTCCCCTCTGTGGCATTGCCTCGGTTCCACCTCACTGTGCCCTCTCACAGACAACTCCCTGTCTGACCCTCACTCCGGTGACCTTCACCCCCCACCCCACAACGTCTCAGACCCTCCCCTGGACACGTCATTCCCTGGCTCTCCCCTGGAAATCCCCGGTCTTCCCTGTACAGTCAGGCGGCCACTGCTCCGCTTGGAACATCGCCAACTTCCTGTCTGCCTGAGCCCCTCAGCCCCCCCATTCTCCCTGTCCCCCATCCAGGGCAGATTCCTCTGGGGCTGAGGCTACTGCAGCCTGTGGCGGGAGAGCTCTGGGCACCCTGTGGGAGAGGCCTCCGTGCTGAGGGCTGTGGGCTGGTGACTTAGACAGTCGGCCCAGCTGCCAAGAGGTGTCCAGTCGCCCAGCGCCCCAAGCCTGGCCTGTGACAGCTCATAGAGGCTGGGCCGTTCTGCCGCACCATGGGAGCCTGGAGAGAGACCTGGGGTGGGTGTGGCAGGGCGGGGACAGTTCTAGGAGAGAGTTGTGGCAATGCAGGTGAGTGGAGGTGAGGCGGCCAGTCCATAACTGAAAAGAGAGCGGTGTTGCCAGGGGCTGGGTGAGAAAATATCCTGGAATTGACAGCTACACAACTCCGAATCTACTGAAACCACTGAATTGGACGCTTTGAATGGGTGAATGGTGTGGCATGTGAATTACCTCTCAATAAAGCTATTAAAAAATACTAATAATCAGCATGAGGTCAACCTAATTGGTACTGACATGAAAAGCCAGTGGCTCCAGAGGAAATCTCAGTCTGAGCTCCCTGTAAAGGGGCGGCCAAATCCGTGTGCTCTCTCCCGCCCTCTCTCATGCACAGCGATCGCCCCAGCCGGGAGCCTGAACCCAGAGGTGTGACTCTCCCCGTTTTTCTTTGTATACTTCCACCCTGCTTGATTTTTTTTTTTTTTGGAGCGTTTTACTTTTGACAGTAAACACCGACCAAAATTCCTCTCCAGTGAGCCCCAAATAACCTCCAGCCTGCCCTCTGCCAGCTGAGCACCAACCACTGCAGATGACGCCCTACGCCATCACCCACTCACCCGTGCAAACACCCTGCAGTGCTGGCTGAGGGCCTGGGGCCAGTGCCAGGGACACAGATAACCAAGCTCAGAGTCCAGGACAGGGGAGAGTAGGGGACACATGAGGAGGGGGCCAGGCACAGTGGCACTGGGCAGGCCCAGGGCTGGCAAGGGGTCCTGAAGAGAGTGCAGGCCACAGGCTGGGCACACGCCCTGTGGAGACCCAACACACCCAGCCCCTCTCCAGGCCCGCTTTTCCCCCAGACCAATGCAGGCCTCCCACCCCTGGCCCCTGCCCCACACCAACGCCACCCACTCACCAACGGTACTCACCCCGGCCTCATTCTCACCCCTGCTGCCCTCTCCAGGACCACCTCACAGCACCCCTAAGCCCCACTCCCATCCCTAACATGACAACCTAAAGTCTCTATCCCCAGCTGCCCAGGCCCCCGGCTGGCCCCACTCCCCACTCCCCGGCCTGCACTCATGCCCTGACCCCCAGGACAGGCCCCTGCCCACAACGCTGGACTGCTGCTGGGCCGCTCTTTCTTTCTTCACTGACCGCCTCACCTCCACTCACCTGCATTGCCACAACTCTCTCCTAGAACTGTCCCCGCCCTGCCACACCCACCCCAGGTCTCTCTCCAGGCTCCCATGGTGTGGCAGGACGGCCCAGCCTCTATGAGCTGTCACAGGCCAGGCTTGGGGGGCTGGGCGACTGGACACCTCTTGGCAGCTGGGCCGACTGTCTAAGTCACCAGCCCACAGCCCTCAGCACGGAGGCCTCTCCCATAGGGTGCCCAGAGCTCTCCTGCCACAGGCTGCAGTAGCCTCAGCCCCAGAGGAATCTGCCCTGGATGGGGGACAGGGAGAATGGGGGGGCTGAGGGGCTCAGGCAGACAGGAATGGTTATGGATTGGCCTGTACTGGCCCTGGGTCTGGCCTCTTCGACTTGCCGGGACGCTGTCAGGGCTGCTCACATCGCCGCGCCATCAGCACCTCACCCTCCCCCCGCTGGAGTCGGTTCCGTGGTGTGGAAGTGCCCACTTTTGCTTATGCGTTTATCAGTTGACGGATAGTGGGTTGTTTCCACCTTTTGGCTGTTATGAGTGAGGCTGCTATGGACGTTTGCATACACGTTTTTGTGTGGTCGTATGTTTTTTATTTCTGCTGGGTAGATACCTAGGAGTGGAATTGCTCAGCTGTGTGGTAATTCTATATTTAACCATTTTGAAGAACTGCCAGATTGCTTCCTAAAGCGGGCGTGCCATTCTGCATGCCCAGTAACAGCCGGGTGCGAGGTTTCCAATTTCCTCACATCCTCACCAACACTTGTTATTATCTGACTCTTTGATTGCAGACATCCAATCCTAGTGGGTGTCGAGTGGAATCTCATTGTGGTTTGATTTGCATTTCCTGATGGCTAATGACGTTGAACACTTTGGTGTGATTGTTGGCCATTTTTTATATCTTTTGAGAAATGTGTATTTCAATCCTTTGTCCATTTTAGAATTTACCTTTTGGTATTTTTGAGTTGTAAGTGTTCTTTATATATTCTAGATGCCAGTCCTTTATCAGAAATATGATTTGCAAACATTTTCTGTTTCTGTGGTCTGCCTTTTTATTTGCTTGCTGGTGTCTTTTTTTTTTTTTTTTTTGAGATGGAGTTTTGCTCTGTCACCCAGGCTGGAGCAGGTTCAAGTGACTCTTGTACCTCAGGCTCCCGAGTAGCTGGGACTACAGGCGTCTGCCACCACACCTGGCTAATTTTTGTATTTTTAGTAGAGATGGGGCTTCGCCATATTGGCCAGGCTGGTCTCAAACTCCTGGCCTCAGCCTCCACAGTGCTGGGATTACAGCGTGAGCCACCTCACCTGGCCGGTGGTGTCCTTTGAAGCACGTACATTTTAATTTTAATTATTATGACTCCAGTTTATCTATCTTTTTCCTTGGTTGCTTGTGCTTTTGGTGCCATATTTAAGAAGCCATTGTCTAATCCAAGTCATGAAGCTTTACACCAATGTTTTCTTTTCTTCCTTTTTTTTTTTTTGAGACGAAGTCTCGCTCTGTCTCGCTCTGTCACCCAGGCTGGAGTGCAGTGGCATGATCTCAGCTCATTGCAACCTCTGCCTCCTGGGTTCAAGCAATTCTCCTGCCTCTGTCCGCCGAGTAGCTGGGATTACATGTGTGCGCCACCACACCTGGCTAATTTTTGTATTTTTAGTAGAGATAGGGTTTCCCCATGTTGGCCAGGCTGGTCTTGAACTCCTGACCTCAAGTAAGCCACCTGCCTCAGCCTCAGTGCTGGGGTTACAGGTGTGAGCCACCGCGCCTGGCCTGAATTCTTTGATTATTAATTAAGCTGAGCACATCTCCTAGAGTTGTTGACTATTTGCACCTTTTCTTTTGTGAACTGCCTATTCACAAGCATTGTTGGCCAGGCTGGTCTTAAACTCCTGTGCTCACATGGAAATGGAATCAAGGGATCCTCCTGCCTTGGCCTCCCAAAGTACTGGGATTACAGGTGTGAGCTGCTGCCATGCCTGGCTACTACACCTTTTTACATCAGGGACTTGAGCATCTGTGGATTCTGGTATCCATGGGGAGTCCAGAACCCATCTCCCGCTGATACCGAGGGACACTTGTATTCGGCGTAAACTTCGAAGTTGCCTGTGGGCAGCACCAGCTCACGGAACAGAACTTTTCAGCTCCTGGAAAGTTCCTGCTGAGTCCCCACTGGGCACACCATGGATGGGTCTCACACCACGCATCTATTTTTGTTTAATTCTGTCTGTTTCTTTTTTTTTTTTTTTTTTTTTTTTTTTGAGATGGAGTCTCGCTCTGTTGCCCAGGCTGGAATGCAGTGGCGCGATCTCGGCTCACTGCAACCTCCGCCTCCCGGGTTCAAGCCATTCTCCTGCCTCAGGCTCCCGAGTAGCTGGGACTACAGGCGCCCGCCACCATGCCCAGCTAATTTTTTGTATTTTTAGTAGAGACGGGGTTTCACCGTGTTAGCCAGGACAGTCTCGATCTCCTGACCTCGTGATCTGCCCACCTCGGCCTCCCAAAGTGCTGGGATTACAGGCGTGAGCCACCGCGCCTGGCCTAATTCTGTCTGTGGTTTCTTTAGCCATATCAATTTTTAACACTCACCAGTCTTTTATGATTTCTGGATTTTATGCTATGCTTAAAATGGCCTTCTTTTAGAAAAACATCTGCTGAGAGTTGCTTCCAGCACTGCTGCATTTTTTTAAACATCTCTGTCGTTAATCTTTGTGGAATTTGCTTGGGGCTGAGGTCGAGATGTTCAAGGAGGCTCGATGTCATGCTTGGTGTCACGCTCATAGGGTCTGGCCGTGGGCTTGTGGGTTACAGGGATGATTGTAAACATCTCCCCTTCCACCAATCCGGCTTCCTGACGCAGGGGCTGTGTATTGCTTCACTTCTGTGCCCCCGAGCCGAGCCCTGCCCTGTCCTGTGGCAGAATCCAGCTTCCTGACGCGGGGACTATGTGTTGCTTCACTTTTGTACCCCCCCAGCCGAGCCCCGCCCTGTCCTGTGGCAGGTGTGGCTTGAAACAAGCGTTGGGTGGGAGGGATAGGTGGACTTGGAGCTGGCTGGACCCCTGCAAGATCCTGGCCTGGTGCCTCCCAAGGAGCGGCTGAGGAGTCCCCCGTGGTGGCTGTGGAGGTAGAGAGCAGCCTGGGGTGGAGAGGATGTGCCTGCGGCAGAGAGGGCGGAAGAGCCAGCGCTTAGCCTTGGGTGCATAAAATCAGAGATGCCTGTGAGATAGGTATGCGTGTGCAGACACTGAGGTGCAGGAAATGCTGGGCTGAGGTGGGGAGGCCAGGCTGGGAGGCCATCACTAGGGCAGGCCTCGGGCCAGAGCTCTGAGCCTGGGGTCCTCCAGAGCTTAGGTGCCTATGAGAAGAGGCAGCCGGGAAGCAGAGCAGTGGGCAGCTGTGCAGCCATGGGGGCCTGTTGGGTCTGCGAAGGAGGAAGGGGCGTGAGGAAGCAGGGCCACGTTGGGAGCAGGGTGAGGCCTGGATGGTGCTGGGACCAGGTGATGAGGGGTGTTGGGACCTCAGCGGGACTGCTTGGGGTGTCGTCGGATTGGAGGTGCCAGGGGGCATGTGGGAAGTGCTCAGGAGGCCCTGCTGGTGCCAGGCTATGGTGGGTCCAGCAGGGTGGCAGGGAGGGAGAACCCTCTCCAGGACAGAGCGGTGACATGGAGGCTAGGCCGCAGCAGGTCACAGCCTCAGCTGGATGCTGGCCCGAGTTCCCTGGGGAAGGAGTTCCCTGGGCCTTGGCCTGAGGGACCTGCATGGTCACAGGGGGCATGGGCATCACCTGGCCCAGGGTGAGTACTGAGTGGTCGCTCACATTTACCCAGCCACACATCATCAGTGGCCAGTCCAGACTGCTGCTCACCACTGAGGGCCCTGGAGATTTTCCCAGGGTTGGGCTGTTGGGAGGGTGTGGGCAGTGGTGCCCTGTGGCCTCAGTTTCCCCAGTGAGGCCCAAGAGGAGCTGGCAGCAGAGGGGCCAGGGGCCAGGTACCTCAAGAAGGGACTGGAGGAGGCTGGGAAGGGCGATGCTAGTGGGACCAGGTGCCCAGATCTCCCACCGCTGGGGTCATGGGTGATGCCCACAGACCCACAGTGGGTAGGGAGATGCGGGGCTGGGAGAGCAGGGGCCGTGGGTCATGAGGTCAGGGACCCAAAATGGGGCATGGGCCGGGTCATCTGTAGGCCCAGTGGAAGGGGAGTTGGGAACACGCGTTCAGACGGTGGAAGGAGGGTGCCAGCTGGGAAGACAGGATCCTGTAGGACTTGGGACATCAGCCTGTGGGGCCTGCTCTCCAGGATTTGGGTCCTGGTTAGCTGCAGAAGAGTCCTCCCAGCTGGTGGTCAGCTCTTGGCAGGACTGTTGCTGCTTCTTCCTGGAGGCAGAGGAGACCCAGGACCAGGAATGGGATGGGGATCACTTTGTGTCCCTGTGTGGCTCCAGGAGTAGCGGCAGACCCAGGGCCAGTGCTGCAGGAAAGGAAGGCCCCAGGACGGCATCAGGGTGGCTGGTGTGAGTCTGGGTGCTCTTGGGCAGGCTGGTGTGTCAGGTGGGCTGGGGCCAAGTGGGACGGTGAGGGTGGGGAAGGCCATCCACAGGGGCACACCAGGGTGGGCGGGGGGGTGGGGGTTGGTTGGGGGAGTGGGGCTGGTCAGGGCTGGAAGTGTGGGTCTGCAGGAGCAGAAGGAGGTAGCTGCAGTAAAGGTCTCTGGTTGTGGCTTTTATGATTTTTATTCTGCTCTGTTTTTCAAATGAATGTGTCTTTTGACCTTTTGAAAGTGCATCTCTTTCTCTTCTTTCTTGCTAAACGAGTGGCTACCTGGGAAGCTAACAGAGCAGGCACAGGATAGGCCTAAGAGACACAGCTGGACGCCACTGTCATGACTGCCACAAGAATGCATGGTTGTTTACAGAATACAGATACCCCCAGAGAATGTGCCCTGTTTTAGGTTCCTGTGGCTGCTGTCACACGTACCCACAAACTGGGTGGCCTTAAACAACAGAAATGTATTCTCTTACGGTTCTGGAGGCCACATGTCCAAAATCAAGATGTTGACAGGGCCACGCTCCTTCGGAGGCTCCAGGGCGGACTCTGTTCCTGGCCTCTCCAGCTCCTGGTGGCCATCGGTGCCTGGGCTCGTGGCCGCATCACTCCAGTGTCGGCCTCCATCCTCCCGTGGCCCTCTCCCCTGTGGCTTCTCTTCCATCTCTTGTAAGACACTTGGCATTGGTTCTAGGCCCCCTACTGGGTAATCTAGGATGATCTCATCTCAAGATCCTTGTGTTAATTACACCTGCAAAGACCCTTTTTCCAAATAAGATACCAATTATGGGTTCCAGGGAATTGTTCATAGACATATCTTTTGGGGACCTCCATTCAACCCACTACAGTCTGTCCTCTGGTCCCTCAAAACTTACATCTGCCCCACTTCCAAAATAAATGTTTCTTATGCCAACATGCCCCAAAGTCTCAACCTATTGTAGCATCAGCTCTAAGTCTAAAATTCCATCCGCTTAGCCAGGCACAGTGGCTCACGCCTGTAATCCCAGCACTTTGGGAGGCCTGTGGGTGGATCACCTGAGATCAGGAGACCAGCCTGGCCAACATGGTGAAGCCTTGTCTCTACTAAAAATACAAAAATCAGCCAGTCATGGTAGTACACACCAATAATCCCAGCTACTCAGGTGTATGAGGCACGAGACTTGCTTGAACCCGAGAGGCAGAGGTTTCAGTGGGCCGAGACTGCACCAATGCACTCCAGCCTGGGTGACAGAGCAAGACTCCATCTCAAAAAAAAAAAAAAAAAAAAATTAGCCAGGTGTGGTGGCACATGGCTGTAGTCCCAGCTGCTCAGGAGGTGGAGGTAGGAGGATTGCTTAACCCTGGAGGCTGAACCCAGGTGACAGAATGAGACCCCATCTTGAAAAAAACAAGAACAACATTTAATCAGCTCAGAAGTCCCAAATCTGATCATCGAAGTTGTGTCTAAATCAGGTGTTGGTGAGGCTCTGGGTAGGATCCAGATGGGGGCAAAATTTTTCTCCATCTTTTTACTTGTAAAACTAGAAAGCAAGTTTTATGCCTCCAAACTGCAATGTTGGGCTAGGCATAGACTAGACATCACCATTCCCAAAGGGAGAAATATAAAGGAATAAAGGGGTTGCCAGTCCCAAGCAAGTCTGAAATCCAGCAGGGCAAATTCCGTGGGGTTTCAAGGCCCGTGAATGCTCTTCTGTGGCTCAGTGCCCCACCCTCTGCACCTGCACCTCTGCCAGCCTCTGCCTCTAGGCCTAAGGCCCTGACCTTGGAGTCATTCTTCCTTTTTCTCAAAGGGTAGCACATGTTTGCAGCTGCCTGGGTCTGTCATCCTGTTTCTTGCCTGTAGACTCTCAGAAGCCCAATACCTTCCTTCATTTCATCCTGTCTCTGTTCCCATCAATCCAAGCAGGCAGTGTTTCTGCTGGTAAAACATTCTCATAAACCTTCTGGGTCTCCCCTGTATGTCACAGGGAGCCAGGCCATTAGAGAGAAGGGTTCACTCGGATCCTCCCTGGATAATCTTGTCTCTATTCCTGGCTTCTGTTGAGATGAGTGAATGGACCCATGAGTCACATACCTCATCTCTGCAGAGATGGCTTTCTCTGCAGAACACACTTTCCTAACAGTGAACCTCCCAACATGAGCATCTTTTGCAATCTAAATAGGCTGCAGATATCCCAAATCACCCATGCTGTTTCCTTTTTGCTTAATAGTTCCTTCCTCAATTTATCTCTTTCTTCCTGTACTTTACCATAAGCAGCAAGAAGCAGCCAAGCCACACCTTCCACACTTTGCTTGGAAATCTTTTCAGCTAAATATCCAAGTTCATTGCTTACAAGTTCTGTTGAGCACCCGACAGTGAACACAGTTCGGCGAAGTTTCGCTTCAATGTGAGAAGCACCTTTCCTCCACTTTCCTCGTTGCCTTCTAAGGCTTCACCACAAGCACCTTTAATACCCATATTTCTACCAACCGTCTGGTCAGACAGGCTAGGCTTTTTCTACTGTGTGCCTCAAGATGTCCCAGCTTCTGTTTATTACCCAATTGCAAAGCCACATCCACATTTTCAGATATTCGTCACAGAGGCACCCTGCTCCTGCTCGCTGTCTGTATTGATTTCCTTTGATTCCCACAGGGAAGAGCCACAGGCAGGGCGCCCTAAAACAATAGAAATTTATCTTTTGATTGTTCCGGAGGCCAGAAGTTAAAAATCCGGTGTCGGCAGGTCCACGCTTTCTCCGAAGGCACCCGGGAAGAGCCTAGGAATAGCCGCCTGCCTCTTCCAACTCCACTGCCCCTTGGCTTGTGGCTGCATCATGCCAATCCCTGCCTCTGTGTCTCTCCTCGTCTAAGGACACTTGCTTTTGGATCTAGGGCCCATCCAGGTAATCCAAGATGATCTCATCTCAAGATCCTTTATTTAATTATATCTGCAGAGACCCTGTTTACAAATAAGGCCACATTTATGAGCTCCAGGGACATGGACATAGATTTTTTTGTGGCCACCATTGGACCTACCCAGCCATCCTGCAGTAGTTGGTTAATGTAGAACTTACACATTTTTGTTCCATAGTCATTGTTTTAAAAAGCGTGCACAGTGGGCTGGGCACGTGATTACAGGTGGCACATGCCTGTAATCCCAGCACTTTGGGAGCCTGAGGTGGGAGGATCTCTTGAGCCCAGAATGTCAACACTGCAGTGAACCTGGGCTACAAGAGCAAGACCCTGTCTCAAAAAGAATAAAAAAATAAAAAAGCATGCACAGTGTTCTTTAGTGTGTGTTAATTTATCTGTAATTTCATGATTTAATGTTATGAAGTATTTTAGACATAAGTAACTTCTTTTACTTAGTGATACAGCTTGGGCATCTCTCTGTGACATTAATTTACAATACAGCATTCCCCTGTATGGAAACCATATCATATTTAACCAATCAATCCTTTCTCCTAGGACACGGAGTTTAATTTTGGATTTTTGTATTAGAAATGACTGCAGTGAACAGTGTTGTAGCTGGGTCATTGTGCATCTCTATGACTGTTGTTAAAAAGGGAATTTCTGGCCAGGTGCGGTGGCTCACGCCTGTAATCCCAACACTTTGGGGGGCCGAGTTGGGCAGATCACGAGGTCAGGAGTACGAGACCAGCCTAACCAACATGGTGAAACGCTGTCTCTACTAAAAATACAAAAATTAGCAGGGTGTGGTGGTGCACGCCTGTAATCCCCGCTACGCAGGAGGCTGAGGCAGGAGAATCACTTGAACCTGGGAGGCGGAGGTTGCAGTGAGCTGAGATCGCGCCATTGCACTCCAGCCTGGGCGACAGGGCAAGACTCTGTCTCAAAAAAAAAAAAAGGAATTTCTGGGTCAAGTGGTTTTCAAAATTTAAGCTTTAATTTACATCAAGATACTGTTTTTTACCTATCGTATTGGCAAAAATCAAAAATCTGGATTTCTAGGTTCTGTTGCTGAAGCTGTGGGAACCAGGCCAGAGAAAGCGTCTGTCAGAGCCACAAACGCGCAGTGTGTGTCCGAGCAGACGATGGTCACTGGCCTGAGTGCGCTCAGAGGCTGGGCCCACACGTGCCGCTCTGCATGGCCAGACGTGGGTCACGGTCCCCGTGCAGAGCGGGAGGCCTCCAGCTGTGGGGTGGGTGGGAAAGCAGGGGCAGAATGGTGCCCGGAAGATTTCACCGTTTGTGTTAAAAGGAAGAAAACCAAGAATCACATGGTATTTGCTTGATTTTCTGTAAAGAAATTCTGGAGAAACAAACAGGAAGCCACTAACAGTGATTTTCACACTGTGTGTGTGGGTGGGGGGAGACAGGGAGAAGGGAGGGGAGCAAGACTTCTCTGTCTACTCTCAGTGTTTGAGTCACGTGAATATTTTCTATTCCAACACGATTTTGATTTTTAAAGTTTAGTGAAGGGAAGAATGTAAACTGTTATAAGTTCTTTTTTTTTTTCTTGAGACAGAGTCTTGCTTTATCACCCAGGCTGGAGTGCAGAGATGCAGTCTCAGCTCACTGCAACCTCCGCCTCCCGGGTTAGAGGGATTCTCCTGCCTCAGCCTCCTGAGTAGCTGGGATTACAGGCACCCGCCACCACACCCGGCTAATTTTTGTATTTTTAGTAGAGACGGGGTTTCACCTTGTTGGCCAGGCTGGTCTCGAACTTCTGACCTCAGGTGATCCGCCCGCCTTGACCTCCCAAAGTGCTGAATTACAGGCATGAGCCACTGGGCTTGGCCTAAGTTTTTATTTTATTTTATTGTTTATTTTATTATTATTATTATTATTATTTGAGACGGAGTTTCGCTCTTGTTGCCCAGGCTGGAGTACAATGGCATGATCTCGGCTCACTGCAACCTCCGCCCTCCGAGTTCAAGCGATTCTTCTGCTTCAGCCTCACAAGTAGCTGGGATTACAGGCATGCACCACCATGCCCAGCTAATTTTGTATTTTTAGTAGAGACAGGGTTTCTCCATGTTGGTCAGGCTGGTCTCGAACTCCCAACTTCAGGTGATCTGCCTGCCTCGGCCTCCCAAAGTGCTGGGATTACAGGCGTGAGCCACCGCGTGCCTGGCCAGTTCTTTTTTATTTTTTTTGAGACGGAGTCTCACTCTGTCGCCCAAGCTGGAGTGTAGTGGCATGATCTCAGCTCAATGCAACCTCCACTTCTCCTGCCTCAGCCTCCTGAGTAGCTGGGACTACAGGCGCCCGCCACCACACCCGGCTAATATTTTGTATTTTTAGTAGAGATGGGGTTTCACTGTGTTAGCCAGGATGGTCTCAATCTCCTGACCTCGTGATCTGCCCGCCTTGGCCTCCCACAGTGCTGGGATTACAGGCGTGAGCCACCACACCAGGTTTCAGACACAATTTTTAAACAATTTCTAAGTTGCTGGCTAGGTACTGCAGTCGTCGTCCCTCCAGCAGTGCCCAAGTGGGCCGACTCCTCCCACCTGCCCAGCACCCGGTGTTGGAGCTTCAGACAAGTCACATGGCCTCGCTGTCTGACGGGAACTTGTGTAAGGAGTCTCCTGGCAGAGCCAGCGGGAGGGTGAGCAGAACTGCGGCCGCGTCAGGTTAGCGCGGCATCTGCCACCTGGGCCTCTGCATCCCGGACCCCGAGCCTGGTCCTGGAGCCATCCTGGAGCCGGGGCGTTCCCTGCATCAGCTCTGACCGATCGTCAGTTATCTTATTCCAGCACGTTTTGTGCTGATGAAGAGTTTTTCCTGCTGTATTTTCCTAACTTGTTATTACTGGCCTAAAGAAATCTCTCGGGTCTTTTAACTGCGTTTGAAGCCAGCCTCTCAATGAACTTTCTCTGAGCTCCAGTAAATGGTGAGTTGAGTTGTTTCAGGTGCCAAGAGGAGCAGTGCTGACTTGGGTAAATGCTGTCCCATGTCCTGGCGCTGGACCGAGAGCTGCGCCTGCACCGTCCGCATTCACCCTCACACGCCCTCCCCTCAGCAGCCTCGCCTCACACTGGGAGGGTGCCCCGCTGTGCTACAAGGGTCATTTATTTGCATGTTAACGATGTAAGCTCATTCCTTGTTATAAATACTGGTTTTTATCAAATGAGCTGTCGAGATTTTCTAGATCAATATACCGGTTTTCTCCTTTAATCTGCTAGTGAAGTCAACGATATTAACAGAGTTCTGGGAAAGTGCATTATCCCTGAACTCCAGGAATTCATTTCTTCTCTTGCTCCATGTTTCCTGTGGTGACACCTTCGTGTCTGGTGCTGGGTGAGGCAGCCGCCCCAGGGGCCCACAGGTGTCTCGGTGTTGGAGGGCTGAGAGGGAGGGAAGGGGGAGCCACCATGGATCAGGTGAGAAGCCTCTCCACAGAGGCCTGGCGAGGTCCACCCTGCTGCAGCAGAGCAGGTGCCTGCGTGAGGAGGGCCTCCAGAGGGCAGCGGCAGGGCTGTGGTGGGACCTGCTTGTGCTCGGAGAGCACTGGCTGCTGTTGTCCAGTGCTTGGAGGTGAGGGGCAACGAGGGAGGCTACCACTGTCTTTGAGGTGAAAGGGTGGTGGCCAGGCCAGGGTGTGGTGGAGACAGTGGGCACAGGACAGGTTTGAGAGATATTTGGGAACGATGGGCCCCCTAATGGATGGTCATGTGGGTGGGACCCAGGACCTTCCCCGCAAATTGGTCTCTAGCAGACACCTGGTATTGAGATACTCTTGTACTGGGCTGCAGGGGAGAGAGGAGAAGGATGTGGCTCGGAGAGATGACCTCTGTGCCTCCTGGGCCCTCACTGGGACTCCCAGCAGCCCTGGTGGGCATCCAGTCCCGCAGTGGTGGCCCTCCACCCCAGAAATCCTGCAGGCTGGCTGGCCCGACTGAGCTCGCACTGAGCCCCACCCTGCGCAGGGGTGGCCTCCAGAGCATGGAAGGCTCTCCTCCCACCTGTCCCGCAGCATCCGCAGAGGCCGGGCTGTTGTTGGGCCAAGGCTGCGAGGCAGAAGCCTGGGGCAGGTGAGGTGGCTCCTGTGGACCTGCCGCAGGCTCAGGCGGGGAGCAGAGCAGGGGTCTGTGCCTCCGGAGGGCTGAGTGTGTGCTGGATGTATCTGGCAGGATTTCACTGGGCCTGGTCTCCCTGGGGCTATTGTCGGGGGCCCCAGGTGGAGGTATCTGCCAGCAGGGTGTCTGCCTGTCGCAGGTCGCCTCTGCATGGGGTCACTGGGTCACAGCCCACTGGCCTGCTCCACCACCACCAGAGAGCCCCAGCAGGGCAGGCTGCCAGGGGGTTAGGGGCTGGGTGGGTGGGTGGGCGTGAAGATGGGGCAGGGAGCAAAGCTGAGGGCAGGCAGGGTGGTGGTGGGCAGGGCCTCAGGGGAGGGCCCATCCCATTCGCAGGTGTGGTGTGGGGGACAGGGTTGGAGCCCCTTCTTTGGCCTGGGCCTGTGGCCAGGGCAGCCAGGGCCTGGGGACTGGTGAAGAGGAGGTTGCTCTGTGCCCAGACAGAGGGGTTGGGAAGGAGGGAGGGCTTATCTGTGGTGCCTGGGCTCAACCTTGGTTCTGGGGTCCCCAGTGGTGTGGGGAATTTGCAGAGAGCTTCATGAGGATTTGGCTTTGCCCAGGGTGTGAGACCTGGCCAGGAGACCTGATGGCACCCCAGCTGGGCATGGGGGCCATGCTGGGTTCGACCAGCAAAGCAGAAGCTAGAGGTAGGGGTTTTCCGGAGGCACTGGTGCTGGTGAGCCCGACTGCAGCCTGGGGGTCCCTGCAGTTCACTGGCCTGGACACACAGACCCGCGTGCTTCTTCGGCAAGACTCCTTTGTGATCCATGAATTGGGGCCAGAGCGATGAGATCTCCTGGGCAGTGGCCGGGGCTTTGCTTCCCACGCTCCAGAGCTGCGTCACCAGGGCGCCCAGCTTCCTCTACCCCAGCTCCCTTTCCTCCCCGCTGCCTAAATCTCTTGCTCGTGGGCCCTTCGCTGCCAGCCTGGGACCTGCCTGTCCTGTTGAAATCCCCGGCTGTAGCCCCGTCTCCAGGCGCCCCCCTCCCGGCTGCAGCCACCTCCACCTTCTTGGGGGTTGCCTGGACTTGTTTCCTCTACGGCAAGGCCTTGGTGCCTGTGGCCTCTCTTCCTGTGCTGGCTGCCTGCGGCCCGTCCCCACATAGCTGTCCCTTCCTTTGGGAAGCCTGTGCCGGGAAGGAACCACAGAGCTGACTGACGTGCAGCTGGCTGAGCCCTTCCCAGGGTCATCGTGGCCGCCCCCACTTCCAAGGCTGCACCATGTGGCCACCGTTCCAGGCAGGCTGGGCTGGGCAGTCTCAGGCTCCTTCATGGGAACTTGCTGCTGCTGCAGCTTGTCGATAAGCCTCTGGCTGCGGGAGACCACAGGTGACCATGGTGGCCACTCACAGGCGGGCAGCTTGACTTCAGGGGGGAAGTTAAAGGCGGAGTCGGTGCATGAAAAGCTGTGTGTTCCTTCTCTGAACAGCTGTGTGCCCTCACCTTCTCATTAGCTTGAAACCATGATCCTATCTTTTTGTTTGTTTTCGAGTCAGTCTTGCTCTCTCCCAGACAGGAATGCAGTGGCGCAATCACGGCTCACTGCAGCCTTGGGTGGGCTGGTTCAAGCCGTCCTTCTGCCTCAGCATCCCAAAGAGCTGGGACCACAGGCCCAGGCCACCGTGTCAGGATAGTTTTTTAGTTTTTTGCAGAGACGGTGTCTTGCTTTGTTGCCCAGACTGGTCCTGAATTCCCAGGATCAAGTGATCCTCCTGTCTCAGCCTCGGAAAGTGCTCAGCCACCACGCCTGGCCAATCTTATAACATTCCATTTTATGCGTTTTAATCCTTTAAAAAAATCTCCCTGGCCGGGCGTGGTGGCTTATGCCTGTAATCCTAGCACTTTGGGAGCCCAAGACGGGTGGATCACCTGAGGTCAGGAGTTCGAGATCAGCCTGACCAACATGGTGAAACCCCATCTCAACTGAAAATACAAAAATTAGCCCGGTGCGGTGGCACACATCCGTAGTCCCAGCTGCAGAGGCTGAGATAGGAGAATTGCTTGAACCCAGGAGGCAGAGGTTGCAGTGAGCCAAGATCACACCATTGCACTCCAGCCTGGGTGACAGAGCGAGATTCTGCCTCAGTTAAAAAAAAAAAAAAAAAAAAGTCTCCCTAGGCTGAGGAAAATTTTTATGCATTAAATTAAAAAAATTGATCTGGACCAGGCAGTGTGGCTCACACTTGTAATTCCAGCACTGGGAGGTGGGAGGATTGCTTGAGCCCAGGAGTTTGAGACCAGCCTGGGCAACACAGAGACAGACTGTCTGTATAAAAAATTAAAAGATGAACTGGGAGTGGTGGTGCACGCCTGTAGTCCCAACTACTCAGAAAGCTGAGGTGGGGGCATCGCTTGAGCCCAGGAGGTCGAGGCTGCTGTGAGCCGTGATTGAGCCACTGCACTCTAGCCTGGGTGACAGAGTGAGACCTTGTCTCAAAAAAACAAAACAAAAAAAAAACAGCATTTTTTAAAAATGTATGAACTTTTTAGATCTCTTTATAAATATCTGTCACTTTCACTTGAGGTTTACTAAGGAAGTATGATTCACTTGCAGGCCAAATTTAGGTTTATCCAGATACAGCTCAGTAAAACCTAGCTGGGAATTCAGCAGTGGCGCGATCTCGGCTCACTGCAACCTCAGCCTCCTGGATTCCAGTGATTCTCGTGCCTCAGCCTCCTGAGTAGCTGGGGCTACAGGCGTGTGACACCATGCCTGGCTGATTTTTTTGTGTATTTTTAGTGGAGATGGGGTTTTGCCATGTTGGCCAGGCTGGTCTCAAACTCCTGACCTCAAGTGGTCCACCTGCCTCGGCCTCCCAAAGTGCTGGGATTCCAGGTATGAGTCCCCGTGCCCGGCCTGAAATTCTGTTTTAATTCCTCACTTCACCATTTTCCTTAAGTTTGGCAACTGGTGTTTCTTGAGACTCCCCACACCCCAACTGGCTGAGTCCCAGGGAGGGCAGGGACGGGCAGGGGGACGAGAGACTCCAGAACTGCTGGGTCTACGGTGGAGGTGGGGCTGGCTTCTGGCCCTTTGCCTGTTAGCTCGTACCCGTGGGAGCTGCCGGCCTCGCTCCTGGATCATGGTCACGTCCTCCATGCCAGCCCCAGCCTGGCGCACAGTGGGTCTGTTTACAGGAGGGGAAGGACTTGCGGGTGAAGGCAGAGGAAAGGCCTTGCTGGAGACTGAGGCAGGCTAGTCACTTCTATCTTGTGGAGGCTGTCCTGCCCCACACTACCAGCACGGACTGGGGTGGAGCACAGTCCCGGGGCCAGTGTTGAAGGGGTGCCCTGGTGTGGGCAGGGCCCAGCATTCCTCCGTGCCCCCTGCAGCGCAGTGAGTGAGGCAGAGCTGACCTCACCCTGCCAGTGCTACCTGCTCCCCCAGGCGTGCGCAGTGACACACAGCTACTCCTGCGGGCTGAGGAGGCTGACCCCATTAGCCATAGGGGTGGTTGGATCAGAGCAGCTGCGAAGGGTCCCTGGTGGTCGTGGGGGGTGGGTGAGGGATGGAGCTGCAGTGTCATCTGGCGGCAGTGGAGATGCTGGCGAGTGGGCAGACTGGGGGGCGCAGCCAGGAGGACCTGTGGGGCTGCCTGTGGGGCATCCTGAGGCTGGGTGGGAGAGGCAGAGACCAGGGTGCAGGTCGGACCTGTCATCTTTGGAGCCAGTGTACAAGGTGCCTGACCCAGGGCTGCGCAGAGGCGAGACGCCCGGGGAAGGGGCACACTGCTGGGGCCGGGGAGGTGGGAGGTGGCCGACCAGGGTCTGGGGGCTTGATCTCAGCAGCCTGCCAGCCCAGGCCCTGGAGTTGGGCCGGCTGAGGAAGTTACTGGGAGATCTGGATGGGTGGGTATCTCCTGCAGGGCGCGGGTAACGGCTTCAAGGCCCTCGCACGTGTGTCCCACAGGCTGTTCTAAGCATGGCTCGTTGCAGTGGACTCGGGGGGCTGTGGGGTGGGCCGGGGGCTGCGGAGCTGGGGAGTGGGCCGTCCGAGCGCTCCTGGATCCGGCCTGGTGACCTGGCAGTCTGGCTGGGGTTAGCGGGGTCGCGTGCCCCAGAACCACAGGCTGGGGGGGAGGGGTGCGCGGTGCGGCCGGGAGCATGCGCCCGGGGCGGCCCTGTCCGGCGCATTGATCAGCTGAAGCCCCGCCCCTGGCCGGCAAGCCCTGCCCCGGACCTCCGGGCTCCTCCCCCGGCTGCGGCGCTGGCCTGGGCAGTGCGGTGCGGGGCTGGACTCTGCCTGGTGGTGGCACAGCCGCCCTCAGCCCCGGCGCAGACATGGACCCCTCGCGAGCCATCCAGAACGAGATCAGCTCCCTCAAAGGTGCGGTCCTGGGGCCGGGCCCGAGCCGGGGGGCGCGGACCACACAGTGACCTCTGGCTGGGTGCAGTCCACGCAGGGTGGCGGGCAGGGTCTGCCATCCCTGGGGTGACACTGTGGGTCGCTGAGCCAGAGCCGCAGGAGACCCCAGACTCGTCCCCCTCCGGGCTCCACCTGGTTCGGGGTCCTACTGGGCTGGCTTCAGCGGCTCCGCAGCCCTGCAGGAGCGTCGGGGGCGGGGTGGCTGCTGCAGCGGCGGCGGCGGCGGCGGCGGCGGCGGGGCGGGGACAGGGACACAGTGGGAGCTCAGCCTTCACAGCAGGGTCAGCCCCTTCCACCCGGCCCGAGTTTGCCGCCCACAACCTGGCGTCCAAGGGGCAGGGCAGGGCTGGGGGACCACTGTGCAGAGGCGCCGGGCCGGCTGGCCCTGAGCAAGTCCACACTGCGGCTCTTTGTCTTGGGGTGGGGTGGGGGGGCTGGGGGCTGCCCAGGAGCCCTGACTGGTTGCTCTGGAAACCCAAAGGGGTCTGGAGTGGGGGGAGAAGGGGCCGCCCAGTCGCACACCCTGGCCCTCCCAGTGCTGAATCAGCTCCCGCTTCGGTTCCCACAGGGTGGGGGCAGGGGGCGGTAGCTTCCGCCCGGGGGTCTTGGGGGACGCCTGGACCCTGGCGCAGGGGTGTGTGTGCTTCGGGGGGGGTGTGTGCTGCAGGAGGTGTGTGCTGCGAGGCGGGGGGGGGTGGCGCATGCCGCAGGGGGGCGTGTGCGGAGCTGGCAGGGTGGGCCAGGAACATTCTGCTCCGGGCTCCTCCCTGCGGCTTCCCTTCCCGCCTGCAGGCCCCTGGCTGGTGGGAGGGAGGGGACTCCCGCCGAGCCCCGCCCCATCTCTGCTGGAGGAAGCTACCGCCCTTTCCTGCCCGGAGCCGGCTTGGGGGAGGCGGGGCTGGTTGGGTGGCGGCCCAGCGGGGGTCAGTCCTGGCACCCCTGCAGGCCTGTGTAGGAGAGAACCTAGGTCCACGTGCTTTTAAGAGGCCTTGGGATCATTTTGGGACAGTGTGGGTATCGTGGGTTAGTTTGGTGGGGCCCTTGGAGATCTGGGGGGGATTAGGTTAGGGAATCAGGCCAGGAAGATGCGGTCGGCCTGCCGGGAGTGCAGGAGGTCAGTGTGGGGGGGTGCGCTGAGGGACTGGCCAAGAGGGTAGGGCCTTCTTTGGGCAC
P1
AGGGCTGGGCAGGCTTGGACCCAGCTGGTGTTGTTCCCAAAGCCACAACCCTGGTGGGTGGAGGGTCCGCAGGTGCTGGTGGTCCGAGGGGCGTGGCCACTGTGGTCCCTACATCTTCCTGGAGCCTCACCTCCTGAGGCTGGCCTTTTCCTGCCTGGGGCTGGCTTCAGAGGGAGGGAGAGCCATGCCGCAGCGAGCTGGCCCTGCCAGAGGGCTGTGGGAACCGTGGCTATGCCTTCGGAGTTTCCCCGCCACCACACCTCTGTGTACGCAGGCGTGCGCGTGCGCGTGCGTGTGTGCGCGCGTGTGTGTGTGTGTGTGTGTGTGTGTGTTTGGAGCCTGCACCGTGGCGCCATGGCGACGGCCCCATAAACCCGCCCCCACAAGCTGCTTGTTGGCCGCCTGCCGGCTGCTGGCCAGCGCCTCCCACCTCGGGATCCCCCCTCTCTCCCACTCCCCCCCACCGCCCTATTTATAATCAGTTCCCGGGTTTGCCGGGCTTGGTGGGAAGGGGTGTGTCGGCTTGGGGCGAGCAGCGATACGGGTGGGGGTCCATGGAGCACAGCGTGGGACAGTGGGAGTGTTGGGTGCGGTTCTCATGCCTGGACCCTCAGTGGGGGCGGCAGCAGCGGGGCGGGGAGGGGGGGGTGGATGATGGGTGTGGCTGGGCCAGGCCTGCAGGGCCTCACAGGGCAGTTTCCTCTTCCCTTCCCTTGGGCCCTGGCCGTCTTCTGGCCTGGCATTCTTCCACCCTGCAGCCATCCATCTGGCCTGCAGACGGGCACTTGGGCTGCCCAGGGTGGGCATCTGTGGGCGGTAGGGACTCGGTGACTTTCTCTGTGGCAGCGGCTGGGAATCTCTGGCCTCCAACAGCTGGGTTGGAGGTGGGGGTGAATTGAGGCCCCTCTGAGCCCCCTAGCCGGTGCTCTGCTCAGAGGGCTGTGGGCTTGGAGAGGCCACTCCTAATCTGGCCTGGAATGGGAAGTGTTAACTTGAGTGGCCACTGCCTCTAAGCCTGGTGTGTGGAGGTGGGGGCTCTGTCCGTTTGCCCGCCTGATGGACAGCAGCAGCGGTGCCTGCAGCCCTCCTCATGTGCTGTGGCAGCTGCCCCCAGCCGCTCCCCTCGGACTTCCTCCCGCAAGACCCCAGCTTCTTCCCCAGGCCCTAGTCCTCTCCGCTCAAGCCTCGCATCCCTCATCCCTCCAGGCCTGGCAGCCCAGCACCCCCCTTCAGATCCTTGTGAAGCCGGGACACCAGAGCCCACATCCCTGCCGAGTACCCCTCCTGCCCTCAGCCCCCCTGCCTGCCCTGCCACCCTCTTCTTTAGACTCTTGGTCTATATCCGCCCCCACTTGCCCCAGCACAGTCTTAGGGACCTTGAAGGATGTGTTTAGAAACTGATGTCATTTCTGACGTTGGAGCCGTGGGGCCTGGTGGGGGGAAAGGGGCAGCTGTGGGGGTGGCCGCTGCCTCTTGAGGTGCCCCCCACTCTACAAGGGCCCCGTCGGCTGTGGGTGGGAGGGGCCGCCCATGCTGCTGAGTCAGAGGGGGCCGGCCCGGCGTCCTGGAGGAAGGCGGGCAGCCGGCAGTGCCGGCAGGGATTGCACGGAGCCAGGCGCCACGAGCCACACAGCCGGGAGCCCGCGCCCTCACGCTCAGCACCCCAGCCTGCCTGCCCGCCCGCCTGCCCTGCCGCTGCCCGCTGTCACACCCTGTAGCGCGGGAGCTTCTCGCAGCGCCCCGGGCCTTCTCGTGGTACCCTGCCTGCTGCCTTTGCCCCCGCACTGACTGCCCGGCTCCAGCAGCCATGGTGGCCGGCATGCTCATGCCACGGGACCAGCTGCGGGCCATCTATGAGGTGCTCTTCCGCGAGGGCGTGATGGTGGCCAAGAAGGACCGGCGGCCCCGCAGCTTGCACCCCCATGTGCCCGGCGTCACCAACCTGCAGGTCATGCGTGCCATGGCGTCCCTGCGGGCACGGGGCCTGGTCCGCGAGACCTTTGCCTGGTGCCACTTTTACTGGTACCTCACCAATGAAGGCATCGCCCACCTCCGCCAGTACCTGCACCTGCCGCCAGAGATCGTGCCCGCCTCTCTGCAGCGCGTGCGCCGCCCCGTCGCCATGGTGATGCCCGCACGCCGCACCCCCCACGTGCAGGCTGTGCAGGGTCCCCTGGGCTCCCCACCCAAGCGGGGGCCGCTGCCGACGGAGGAGCAGCGGGTCTACCGTCGGAAGGAGCTTGAGGAGGTGTCACCTGAGACCCCTGTGGTGCCTGCTACCACCCAGCGGACCCTGGCCAGGCCAGGCCCGGAGCCTGCCCCAGCCACAGGTCAGCTGCACCCTGACCCCAAGTCATGATGGGTGGCAAGTAGGGGTCTGGTTGTCGGGTCTTGGGCCTGGGCCTGGGCTGGAGTGGTTGGAGTGGTCAGCCGGCTTGCGGAGCGTCGCTCACACCAGCAGGTGGCCCCCCCTAGCCGAGGGTCAGGGGGTGCGCTTGCTGATGGGCCCATGTGGCCCCTCCTCCTCGGCTGGGGACCTTCGGGTGTGTAGGCCAAGGAGGAGCCTGTGGCCCAGGCACACCCCGGGGCGCCCCCTCCCCTCTCTCCAACACTGCCCTTGCCTTCGCCCCCTCCGCCTCCTCTCCTGTCCCAGTGACCTGTCTTCCCCGAGGCTCCTCGGCACTGAGGTGGGGGTGTGCGAGGAGGTGCTGGCGCCGGTTGGGTGTGGCCCTCCTCCAGCACATGGCCTGGCCGCCTCCCTTGCCGCGGGGGTTGATGGGTGAGGCTTGGTGCCGCACAGAGTGCCCGGTGTGACCGGTAACTGAGTGCTTGGCCAGCTTGGACTTGGTGGTGGCTGGGGACGGCCCTGGGCGGGATGGGGAGAAGCGCAGGGTCCAGGCAGGAGGCTCCTCCGGCAGGGCAGAGGCCAGGCTGTGGTGTGGTGTCTGCTGGCCCAGCCTGGGGCCTAGATAGGCCTTTCCCGGTACGAACCAGGTC
                P2
GCACAGGACAGGACGGGTCCTGTCATCCCAGAGGCTTCCAGAGACCCTTTGCTCTGCCCCTTCTCTGTGGCCTCCCTCTGTCCACAAGCACCTGTACCAGCAGGACCCAGGTGGGCAGATGTGTCCCCTGCAGCCCGAGGCTCACACACACACTGTGGAGCACTCTGCCCTCCGTGCTGGCTCCGGATGGCCCGGTCCCCCAGCACCTGGCCCCACCTCCCTCTTTGGGGCTGGCACCTGGGAGCAGCTTGCTCTCTCAGGCTGAAGAAGGGGGCCTGGGGTGGGGCTCTCAGGGTGGGCAGGATTTGCCAGGGTGTGGGGGAGGGGGCCCAGGGGAGGCCCAAGTTTAGGGCGCAGCTGCCCGTGTGGGATGCTGGGCGGAGCCCTGGTGCTGGCTGGCCTGGGGTGAGTTGGACTTGTTAGGGGCTTGCCTTGGGCCCATCTGGGAGGATCAGTGAGGCTGTGGGGTGGAGGCTGGCCTCCCCTCCTGGCTCTCTCTGCGGCTTTGGACAGGTTTAGTGGCCTCTCCAGCTCCTCTCTAAATGGACGACATGTCTCCCGACAGGAGGCATGAGACAAAGACTGCTTGGCCAGGCATGTAGTCCAGGCCCCGGCCCATGTGGCCTTCCTGCTGTCCTCTCCCACCCGGCACCCCTGCCCACCCACTCCCCGGTCATTTGGCAGCCCAGCTTCCCCATGCTCAACTCCTAGCTGTGGTGACTGGGAGTCCCTCCCCTCCTTTTTCTCCTTCCTTGTCCGTGGCTGGGCTGGGCCTGCAGGGCGGGCAGCTGGCGGCAGGGCCAAGAGTGGGGTGTGCTGGGTGTGGCTGCAGCAGGCAGAGCGGAGGGCACCCCTCCCCGATGGAGGTGACTCAGTCCCCCCAAAGCCACAGCCGCCAGCAGCAGCTCAGAGTTTCCAACAGGAAGCGAGCCACAGCTCCGGGATGAGTCAGGGCCAGCAGGCTCAAACCCCTCTCTGCCCTGGACCTCACGGGCTGAGCTTGGGATGGGAAGCTAGGGGAAGGAGGCGGCTCGGATGTGGGGTGTGGCAGAGGGCCAGGGCTGGGCCTCAGGCAGTGACAGGGGTCCGGATGGCCCTGCATACAGCCCGGGGCAGTTCTGATGATACCTGAGACCAGGCACTTCTTGGAGTTTTTAAGTGACCAAGCAGTGAGCTGCCCTGCCCTTCCCCACTGCACAGCCGCTCCGTGGGTTGGCAGAAGGGCAAGGTCAGAGCTCGTGGGCCAACTGTGCTGGTCCAGGATGGCCCTGTTGGATGGGCTGCCCCTCTCCACCCAGCCCTGGCTAGGGAGCACCAGCTGTACCCCTGCAGTGAGGCCCCTGCCCTCCCTGTGCCCTGGTGGTCTGCTTGTCCCTGGGTGGGGCCCATGGGGGCCTGCCAGCAACACATCTGTGTGGGAAGCCATCCGTCCACCTGCTTGTTTAGCCATGCACAGGCCAGGGCCAGACCCAGATGGAGCCCCTGCCCCAGGGGGCTCCAGATATGTGGGGTGAACTGGGTGAGGAGAGCGTGATGGGAAGGTGGCTTCAGAAGCAATCAGGGAGGGCTCTGAGGAGGTGCCTACCCTGCCAGGAGCAGCCCAGAGAGAGGGACAGAGGGAGGCCGGAAGGCCAGAGGGAGAGGACCCATGGGAAGGCCCTGGGCCTGTGGCAGAGTTTCAGTTGCTTGGTGTGGTGGGAAGCTGTAGGAATGTGTTTTTATATGTGAGTTTCATTTACGTTTCATTTTTATTTTACTTATTGTTTTGAGATGGAGTCCCGCTCTGTTGCCCAGGCTGGAGTGCAATAGCACAGTCTTGGCTCACTGCAACCTCCGCCTCCCGGGTTGAAGCGATTCTCCTGTCTCAGCCTCCTGAGTAGCTGGGATTACAGGCATGAGCCACCATGCCTGGCTAATTTTTGTTTTTTTTTTTTAGTAGAGACGGGGTTTCGCCATGTTGGCCAGGCTGGTCTTGAAGTCCTGACCTCAGGTGATCCGCCTGCCTCAGCCTCCCAAAGTGCTGGGATTATAGCTACCGCCCAGCCCTAATTTACATTGCAAATGGCGATGCTTTGCCTGGCTCAAAATTCTGAAGGAACCGAGGATTGTGTGGAGGAGACGCCAGCTGAGCGCTGTCCATGCACCAGCAACGGGCAGGGGCCAGGTAGCCGCCACCACGGTGAGCGCTGTACACGCACAGCACCTAGAGCTGCTTCCCACAGCAAAGGGCAGGGGTTGGGTAGCCACCATGACTTCCCCTCTACACGGAGGACACGCCTGTGCCTTGACCTCCCCCCTTGACCCTCTGTGGCCCTGCGTCAAGGAGGAAGCCTGGAGGTGGCCTGGCTGCCTTGCCCTTCTTCCCAGCCACAGGGTGCTCTAGGTGGAGGGACTATGGCCCGGCCAGCTTTCCACAGGGATCACTGGCTTGGCCGAGTCACCCGTTTGCGGGATTTCTGTAGATCGCCGGCAGGCTTCTAAGGGGTAGTTACGATGGGATTTGATTTGCACTTCACTGGTCAGCAAGCACCTGGGTGGAGAGGTATGGAGAGGGGAGGCAGGTGTCACCATTGCCCCTGGACAGATGCCTGGGGCTGTGCTGGTTGGGGAGGACTCAGGTGGAGGAAGAGACCACCTTTGGGGCTGAGGAATGTGAGTCTCAAGGAGTGGGGTGTGGTGGGCAGTTTGGGGCTCTGCTCCCGCCTGGCTCCTGAAATACCGCTGGGCACATGGATGCCTGGAATGTGGGTGGGAGGGTGGGAGGGCAGGGCTGGGCTGGTGGGGCCTGCAGCGGGAAGGGGAGGCTACAGGGTGGGGTCTGCTGGGCGGGGGGTCTGCTGGGCGGGGCAGGAACAGTAGGGAAGACCCGTGGGCTGGGGCCGGAGGGCTCTCTGGGATTGCAGGCAGGCCAGTCCCCTGCACCCCCAGGCAGGTCTGCTGGGCTGGTCTGACCCGTCAGTCGGCTGGGCTTCATCTCTGCTTACTGACTAAGGGGTGGCTGCGAGGAGGAGCCGCTGCTACCAGGGCATGAGTGGGCCCTCCCTCTGCCTCCCACCCCTTCCCTCCTTCCTGCCTCGCCTTCCTCCCTGCCGGCTCTCGGAGCAGCCTGGCCTGTGTGGCAGCCATGGACAGGTACAGCATGGAGGAGCTGATTCAGCTGGGCCAAGGTAGGGCAGGCAGAGCTGGGGGCTGTGGGAGGCAGAGGAGAGGGCCGGGGCCGCCTCTGGCAGACCTGTCTCTTCCCGTTGTCCACCGTTCTGTGCCGTCAGACCTGGGATGCAGGGATAGGCACAGCCCCCTCTCGGAGCTGCCCCAGGGGTGTGTGTGATGTGTGTCCCGGAAGCACAGGCCTGTCCGGACCTCCGTGGAGTGGTAGAAGAACTGCCCGCTGCCAGCCTGGGGCACTCCTCCCGCCAGGAGCTGGTTCTCACCCAGGCCATTGAGTCAGGCTCCTCTGAGGCTTCCCCAGCACGTGGCAGGGAGTGGGCTGAAACGTCCGGCCTGTCGGCCCCTGCCCACTGCTGGCCAGATGCTGTCCTGGGCTCAGCTGGGAGTTGGGCCCTTTCTGGCATGCCTGGGTATCCCTGACCCTCAGGGCTGCCTGGGCCCGCCCGACCCCTGGCCGCCCAGAGTGGGCCACTGCAGGCATTTCCCTCGGCCGCACGTTTTTGAGGTGTGGGGCTGGAGAGCAGGAGGCTGGGGTGGGCCCGGCCCTCTTGTTGTCCGCGGGCTTCCCAGCATGGACCTGGGTGGCCTGCCCAGTGGCCCGGGGCCAGGCTGGTTCACTGTGCCTTGCTGTGGGCTGAGGTAACTACACTGGAGGCCACCTGGGCTCAGATGGCAGCGTGCGGGACAGCAGGCGGCGCCTCAGGCTCCTGGTCTGTATGAGGCCTTCATGGTATAGGACTTCATGGGGTTTGCTGAGGGTTCCATGTGGTGCCAGGAGCGACGCTGTGGAATGGGGTATACCGTCAACGTGCATTCTCCTGGGCCTGAGGGGTGCGCCCAGCAGACAGGGGTCCCAGGAAGAGGAGCAGGACTGTGCGTGTCCCACGTCCTGTTTCCCCGGCTGCATTGGGATCTGATCTGCATAGGATATGCTGTGGGCCTGCCTGTGGCCCGGCTCTCTGACCCAGGCCTTCCACAGAGCAGGGCTTCGGGACTCCTGGGTGGAGCCAGGCCAGTCTCCTGCCGTGGCCCAGGAGAGGCGTCTCCATCCCAGATCCCTGGGACCCCAGGAGAGGCTGTGCTGTGGGGCAGGCGCAGGCCTGAGCCCTGGTTTCGGGCTGCCTGGGTCTCTGGCCTGCGCGTGACTTTGGGGTGGCTGTCCCGGCCACCGGTGGGGCCAGGTGCTGTGTTGAGTGACCTGCGCAGGGCCCTCTGGGAGAATGGGCGGCCTCTTCTCTTGGCCCCTGTGGCCTTTCCAAGAGCACCCCCTGGCTTGGCAGGGCAGGTGGGTGGGGACTGCACCGCCTCCTGCAGGGACGTGAGCCCGCACCTCCCGCGCCCTGCTCTATTATATTAGAACCCCCTGTGGGAGCTGGGGGACCGAGGTGTCGGGTGGCACCGTCCAGCCAGACCTGACTGCAGCGGGGCCTGGGTGCCCTGGCGCCGTGCGTCATGTTCCAAGTCTGGGTGGTGACGGTCCTTGCGCGCCCTCCTCGGATATTTATATCCCCTGGGCCCCTGCCCACTGCTCCCCTCCCCCACAAGCTGCTGCTGACAGCAGCACGGCCGTGCCCTCCTCCCACCCGACGCTGCCGCCAGTGGCTTGTGCCTCCTCCGAGGGGCCTGTGACCTCCCACACCCCTGGCCCGCTCCGTCTGCCCCGTGGGCTCCTGCCACCGTCCCCGATGAAGATCGTGCCCGGTAGGTGGGCAGCCCTTGAAAGCCTTGTGAAATCCTGGCCCCTTGCTGGGGACAGGGGCCAGAGGGCTGGGGCTGAGGCTGGAGGCCAGGGGCGCTGTCCCTCGCCCTGCACACAGCCTGCCTCATGTGCCCTCACCCAGACCCCCTCAGCCCTGGTCTGCAGTCTCACTTTTGGGGTGTTGGGGCTGGTGAGGAGGGCGAAGTTGAAGGAGTGCAGTGGGTTCAGCTGCAGGCTTGAGGTGGGGGCTCTGGCATCCCTCCATGCCTGCCAGCTGTCCCCAGAGGGCAGCCTTGGAGGCAGAGTGGGCTGCTCGGGCCCCTCTTCTGGTGGGGACAGCAGGGGGAGGCTGGGAGGGAGGGAGGGTGTGTGCCGAGGCCGCGGGCAGTGCTGGGGGGCTGCGGCTTGGCCAGGCGTGCGGCTGGCCCCTCCTCCCGGCGGGTGTGGCCGCGGGGACGCAGGGGTTAAGGCCAACTAACCTTGAGCGTCAGTAGCCCTGCGCAGGGACAGCTGAGCCGGCCGGGTGGGGAGCGAGGGTGGGGAGGCGCCGAGGGCCGGACCCGCGGAGGGAGGAGGAGAGGAGGCGGGCGGGAGCTGTTGTGGGGCTGCCGCCGCGTATGGGGCGGTCCTGCGGGCAGGGCAGGCTCCGGTCCCGGCGCTCCCCCTCGCTCGCCCTGCACGCAGGGAGGGGGCTGTCTAGCCTGGAGCAGCCGGTTCCCAGCGCAGGAAGGGCAGCCCTGGCGGCTCCGCGTGGCGGCTATGGAGCCCTCGGGCAGCCTGTTTCCCTCCCTGGTGGTTGTGGGTCACGTTGTCACCCTGGCCGCTGTGTGGCACTGGCGCAGGGGACGTCGGTGGGCGCAGGACGAGCAAGGTAAGCCCCGCTGGTGGCTGCCAGGGACTCGAGGCGGGGCAGAGGGGCCGCCTGCCTCCCGCCCCTTCATTCCCTGCGCCCTCCCCTTCCTGTTTCCAAGCTGAATGTCAGTGAAAGCAGTGTTGCCCCTCCACCCCACCCTGCAGTGTAGACCCTGACCCTGCTGCAGTCCCAGGGCGGGGCGGGCTCTGCAGTGGCCCTCAGCCTGCAGCCTGTGCGGGACAGGTGGGTGGAAGCGGAGGCCGGCAGGGCAGGAGCGTCCCCCACACGAAGGGAGGCCTGTGGCAGCCGCCGTGGCAGGGACAAGTGGGCAGGTGCCTGTCCGGCCAGCACTGAGGTGGAGGTGGCTCCCTGCACACACAGCAGGGGGTGGAGCCACACGCAGGTGAGGCAGGTGCCCTCATGCCCTCGGGCCGCACAGGGGGCGGGGGCAGCCAGGCTGTGCCCTGCCCTCCCTGCTCAAAGTCACCAGCTCCTGGGCTGTGGTCCCAGGTGGCAACCGTGCCCAGCACAGGATGGGGCGGGGCCCTGGGGATTGGGCAGCCTCAATTATGGGCTTCTGGCCAGAGGGACTGCCTATCGGGGACCTGGCTGGCCAGACCTCTCCTGCAGTGAGGGCGCTGCCTGGCTCAGCTCCTCTGCTGGATCCCCGGACTTGAGATGAGTCAAGATGGGGCTTCTAGGCGTGGCGCTGGGGACGCCCGGAGCTGCCGGGGACTCAGCTGCCAGGATGGGACTTGGGCTGAGGGCTTCTTCCCCATCCATGTTTTTGGCTGGGGACCAGACACTGTCCCTGCCTCCCCACCTCAGCCCACAATTGCCCCCTTCGGCCACGGTCCTGGCTCACAGGATGCCTCTACTCCCTGGTGGTGCCAGACGCGGCTCAGCTCCTCGGTTCTGCCTCCAGCGTTGTCTGCCCACCACCTGGCCCCATGCCCTGCCTTCCTCCCTGGGCCCTGCAGTCCCCACCGCACATCCTGCTGGGAGGCCCCCTCCCATGCCGCTTCAGTCCCAGGGCAGCCTCCCAGCCCGGCCCGTGGGTTGGGGCGCGGCTTCCTGGCTGCGCTGGGCGGGGCTGCGCGCCAGGGGCAGGTTCTTTGGGTCTGGGCCTCGCTCCCGCAGTGGGCGGATTGTGGGGGGGAGGTGGAAGAGGTGGAGAGCGAAGCCGGCAGCCTCGCAGCGTGGGCTGGAAGCAGAGGCGCCGCCGCGGTCCAGCTCCCCGCACTGTCCGTGGGCCGAACCAGGGCTCCGGCCGTGGCCGGCATGTCGGGGGCGGGGGGCGCCTTTGCCTCGCCGAGGGAGGTCTTGCTGGAGCGGCCGTGCTGGCTGGACGGGGGCTGCGAGCCGGCCCGCAGGGGCTACCTCTACCAGCAGCTGTGCTGCGTAGGTGGGTGCAGTGGCGTGGCCGGTGGCACAGAAGGGGCCACCATCGCTCTCACCTAGTGGGGCTGGGTCTCTGCTGGGAACCGCCGCCTGGGCTGGGATGGGGGTGGCGCCTTGGCCCGGTGCAGCCTTGCCCCCGAGCTCCGGAAACACCTGGGGAGGAAGGCGGGGTGATGTTCCCCTACCCCCATTTGTTTGTCTCTAGAGAAGGGGCTGGAGGGGGCGGGGCTGGAGGGGGCGGGGCTGGCCCACCCCCGGCTGTTCCACGTCAGCCTGGGCTTTGGCCTGGGTTTCCAGGACACGGGGGAGGGAGGAGGTGCTGAAGGCGGGGCTCCAGGCCTTCTGTGGACTTGCTGCAGTCCTGGGCCAGTGGGATTCCCTTCCCAGTCTTGGGGTTTCCATCCTAGGACCGTCTCCACTAGCTGCGGGACCTCAGCCATAGGGTCCACACCTGTAAAACGGGGGCGATACTCCTCAGAGGGCCACGGCAGTGCCTGTCGTGAGGGACCCTGGGCTGGCGGCCGAGGGTCAGCAGCCCTGGACAGCACAGGTGTCAGGCAGGTGTGCAGAGGCACCTGAGCTGGGGGTGTTCTGGGTGCGTCCCAGGTGGGAGAGCCAGCTGAGCTGCAGCTGTAGAGGGTGGGTGTAGCCTGGCTGGGTGGGGCGGGTGTGTCTGTTGGTTTTAGGGTCAGAATAGGATTGGATGGGGCGGGGCAGGCTGGGCTGGGAGGGAGGGGAGGTGATGAGGAGGGAAGAGCTCAGAGCAAAGAGGCACCCAGAGGCAGGAAGTAGGGAAGGGGAGCTTGAGCCAGGCTTGGGAGGTGGAGGCCCCTGACCGAGCCCCCAGCCCACCTGGTCCCAGCCCTGCCCCGCCCCAGCCCTTCTCCTCACCCGGTCCCAGCCTTGCCCGAGTCCGTGTCTGGAGTCCCCACCGTGCCCCTTCCCTTCCCTGAGCCCCTGCCCCATTCCTGACCCTGGCCCGGCCCAGGGCGGCATCTTTGGAGGCCTGTTAGTGGCAGGGAGAAGGCTGGAACAGGGGCCTGGCTGGGGTGGGCTCCACGTGGGTCCTGGACCTTGGCCCCGGCTCCCAGTTTCCAGCTGTCGTGGACTCCCTGTGCCCACCTCCCTGGCTGTTCCGGGATCTCACACTGTGCCTCAGCAGAGTGGCAGCCCCGTTCCTGCAGGCAGGAGCCACCTGGTCACGGCCGGGCCCCCAGAGCCCATACCCAGCAGGCGCGGGGCTTGGCCTCAGGGTAGCTGCTGGCCCAGGGCTTCTGAAGAGGCGACAGTGTTTCCAGAGGCTTTGGACCCAGGGAGGGGCGGCAGAGGTGTGGGGTCCGGGGTGCCCTTGGGCACAAGACTGGAGGTGCTGACCAGTGCGGGGTCAGATTTTGATTTGGGGGCTTCTGGTGACTCCTGAGGTTCTGGAGGCTGCTGGGCCGGACAGGCAGAGCAAGAGGGTCACCCTGAATCTCATGCCAGGCTCAGAGACCATGCATGGAGACCAAGGGGTGTCTTCGTTGGGCGGCGTGTACGATGTGGCAGAGCCCTAGGGGCTGGCTGTGCCCCTTGGCGTGGAGTGGGGGCTTCCCCACCTCTGAGGGGTTTGGGCACCCCCCATTGTGAGGGTTCTGGTGCCCCCTCACGGCCTCAGGGGCAGTAGGAATGAGGTGGGGTGAGCTCTTCCCAGAACTGAGGCCGAGTGCAGCTCCCAGCTGGACCGAATCTGCCTGATGTGCGGCTGGAACATTCTTGTGGTTGGGGCAGCGCATTCTTTCCTATGGTGGTCGGGTGGGCTGGGCAGGCCCAGAAGCTGGCCAGAGGCAGCTCCGCCCCGGACCCCAGGGAGCCGGAGGCCCCAGCCAGCGCAGGCCCTTTGCCCTTTGCCCTTTACAGCATGAGACTGCTTCCCTTGAGCCCCACCCAGCTGCAAACAGCACCCCCCACCCCTGGGGTCGCCCAGGTCCTGCCTCAGGTGTGGCTTCCCCTCTGTGAGTCACAGGCTTGGGCCCCCTCCCCATCCCTGACTCACTGGCGAGGCCCCTCCTGGGGCTTCCCGATGCTCCATTCCCCCATGAGGCCCGGCCTCCGTGAGTCAGCCCTCAGCTACGCGCCTCCGACGTGAGGTGTCTGCCTGGGGCAGGGTGGGCTCCGCAGGTGGGCACTGCTCCAGGATGTGCCAGGTGGGCAGCTTGGGAGGGGCCAGGCCTGGGGTGGGACCCCACTGGCCCCAGGGCCAGGGCAGGCACCATGGGGCAGGGTGTGCTCCCCGCTTCATGCAGGTCTGTGAAGAGGTGTGATTGGGGGGCTGCAAGAGCTGACAGGCAGCAGGTCCCCTCCCCTCGCCAGGTATCAGCGGCTTCTTGAGTCCGCCCTGGCAGGGTGGGTGGGCAGAGGCTGAAGCAGGGCCTGGCCCAGGCCTGGAGGGAGCTCCAAAGAGAGGCGTGGCCTCAGAGCCCCTCCCGTCGGGGGAGCCCTTCAGTCTGTTGGGGGGTCCTTCTGGGTGGCTGTGGGGACGGCCTGTGTTCCTCTGCGGTCCTGGCACCATTTGGTGTGCAGTGAAGTGCCAGCTCCCCGTGTCCCCAGGTCCCCCTAGGGGGCTGCAGTGGTCGGGGTGGGTGAGGAAACCCCGGTTTCCTGTTTCTGGCGGGCCGAGGGCGTGTCTGTGGGGGAGGGGGCTGCTTGCTCCTTCCCGCCGGGACACTCCCCTCCCTGGCGGCGGCCTGGGCAGGGTGGGGCCTCGGGGGCGGGCCGGCCTCGCCGGGGAGTACAGCAGGCAGGGAGTGGCCGCACCGACTGGGGCTCTGAGCTGAGCCTGTGCCGAGTGAGCGTCTCGTGGGGCCGCCAGCCTTCAGCTGCCTGTGTGCCACACGGGGCTGCCCAGAGCACCGTGCCTGAGGCCGAGGGTCCGGGCGACGAAGGTGTGGGGGCAGCATGTCTCAGCACCAGCTCCGCGTGCCGCAGCCCGAGGGCCTGGGCCGAAAGAGAACCAGCTCGGAGGACAACCTGTACCTGGCTGTGCTCAGGGCCTCTGAGGGCAAGAAAGGTAGCAGGGCTCCCCTCGGGCACCCCCGCCAGGCAGGGCGGGCCCCTGCGGGAGGAAGGCCCACTTCCTGTGGCAGGAAAGCCTTGGCACCGCTGGGGGGCTGGTCTTGGAGATGGGGGTCGGGGGCCAGCCATCCCCACCCAACACGGAGTGGGCCGACAGGCGCCCGCGAGGCTGACGGGGGCCTCCGAGCGTCAGATGTCGGGCCTGGAAGGGCGAGGAACTCTGCGCTCTGGGGAAGGTTCGCTGCCCTGTCAGCAGCACACGCGGCTTCCCTTCCAACACCCCAGCGGGCCCGGGCCCTGGCTCTGCCACACCCCTGCTTGGGTCTCAGTCTGTAGAGGCCGGGGGGGACTGCAGGACGGGACTTGTGTGCCCTGGGCTCAGGGTGCGCCCAGGACCCTCCCATTTCCTCGGGGGAAGAGTCCGGGGTGGGGTGGCTGCACCTGGTGGCGCCTGGGCCCCTGGCACCTGGGTCTCTGCTGTGTGGCTGGGCTGCGGGTGAGGGCGCCTGGGTCTCAGTGTGTGATCTGAGGGAGGCTTTGGGGCTCCCCAGGCAGGACCTGGCCAGCCAGGCTGCTGTGTCTGCACGGTGGTCACCTGAGGGCCCCCGGGGCTTCTCCCAGGCCCCAGGTAAGCTGGTCTTGTCTCCCCCAGATGAGCGGGATCGTGTGCAGAAGAAAACCTTCACCAAGTGGGTCAACAAGCACCTCATCAAGGTTGGTGGCGCATGCTGGTGCGTGTCACAGGGGCTGAGGCTGTGGGAGGCGCTGAGGGAGGGTGGCCCCTGCCGCCCAAGGCTGTCCCCATGCTGCCTGTGTGCTCACCTTTCATCTCTGAAACCTGTTATTTTCTGTCCTCTTTCTTCCCTACGCCTGCTCCTGCCTCTCTCTTCCTCCTCTTCCTTCTGGTCTTTCCTGGTCTCTCCTTGCAGCACTGGCGGGCAGAGGTAGGTGCCTCCGTGGGGCAGGGTGTGTTCCGTGGGCTCCCGGGAGACTCAGCCCCCGCCTCCTTCCCTCTGGGACCAGCACCCACTCTGTAGATCAGCCACTCAGGGCCTATTGGTGGCTCTGGGGGACTGGCCACCTCAGCCGCTGCTGTCCCCACAGGCCCAGAGGCACATCAGTGACCTGTATGAAGACCTCCGCGATGGCCACAACCTCATCTCCCTGCTGGAGGTCCTCTCGGGGGACAGCCTGGTACGTGTGCCCCTGCCCCCTCCGGGCCCCGCCTGCCCCACCTGGAGACCTCTGCCTGCCTTCCCTCCTTGGGGCCTCTCCGGCCGCCCACTCTACTTGGAATCCAGCTCAACCCCTCCTGCCTTCTCCCTGGGGGCAGGCTGGCCTCGTCCCTGGCAGCACCTCCCGCCCAGGCTGCTGGGCATCACAGCCGAGGGCCTGGCCTGGGAGTGGCCGCAACCCTCGGACCTCGGCTGCTCTGGGCCTTGCCGCCCTCCGCGTCGGGACACAGTAACCTCATGCTCCGCTTTGCTTTGGTTTCTCTGCTGCTTGGACTCTGAACCTTGACCTCTGCCCTTTACCCTTTGCCCTGCTCTGGCCGGGCAGCCGAGGGAGCGGGACGTAAGCAGGAGCTCACGCCTGGTGAGTGGCTGTGCCTGCCGGCTGCAGCGGCACGGGAGCTCCCCAAGACCGTGGAGCCCGGCTTTCTGGGGGCTGGTGGCCCGTCGGCCAACCCCTGCCTGGCTCTTGGGCGGGTAGGTGGTTGGGTGTCAAGCTGCCCGTCCACCGTGCTGCCCAGAGCTGGCCTGGGACCCACCGGTGGCTGCTGTGGCTGCCACCGCCAGGCAGGAGGACCCCCCCTTTAGTGCCACTGCCCTCCACACGAGGCTTCCTGGCTATTGTCTCCTCCCACCCCTTCCTTCCTTCCCTCAGCCTGGGTCGCTGGACTTCCCGAGCCCTGATGGGCCTGGCCTGGCCCCTGGGCCGGTGGCCTCCCTTTCCCTGCGGGGGAGGGTGAACCTGGGATGAGGGGCTGCTGCCCCCTGGGGAAGACCCTTGGCTCGGTTGCTGCGGCGCTTTGGGGCATGCTGAGGGTGGGGCCCGGGAGCTGCAGCTGCACTGTGGGTGCTGACTGTGCCTCCCCACAGCCCCGGGAGAAGGGGAGGATGCGTTTCCACAAGCTGCAGAATGTCCAGATTGCCCTGGACTACCTCCGGCACCGCCAGGTAAGGCTGCCCGGCAGGCCCTGGGCCCCACCCAGACCCCTCACCAAGCCAGCCCCGCCCTGTGGGCCGTGACCGAGAGCCCCTCGCTCACAGGTGAAGCTGGTGAACATCAGGAATGATGACATCGCTGACGGCAACCCCAAGCTGACCCTTGGCCTCATCTGGACAATCATTCTGCACTTCCAGGTAGAACGGCTGCCCCCAGGACCCCCACCCCCTGCAGGAGTTTCCAGGGTAGCCTGGGCTCCTTGGCTGGCGGGTTGATCCGCTTTCTGGCTAGGGCGTGGCCTGGGAGGTGACTGGCGCCGTAGGTATGGTGGCACCTGGGTGCCTTCCTGCCAGCCGCAACCCTGGACTGGATCTGTGGCCTGAGGGTACAGGCCTCTCTCTGAGGCCACCTATCCGTGCCCTCTGGCCCTGGCTGGGCAGGGCCCAGAGCCTCCCTGTGGAGGAATGCAGGCCCCGGTGCCTGCCCTGGGGAGGCTTCTGCGCAGCACAGGCTGACTCACCCGCTGCTTGTCCTGGCTGCACAGGAAACCACAAATCTGAGAGCTGAGCAGGCAGAGCCTGGTGCTGGCACGGCAGCTGAGTCACACAGGCTGGCAGGACGACAGGCAGCTAGGGGTGGGTGGGCAGAACCTGGCCTGAGCATGCCACTGAGTGTAAAGCCAGGCTGCCAGAGGCCCCACCTGCCAGCCTGCAACCATCGATGGAGCTCGACCCCCAGCCTTTTCCAGGCCTGGGGGTTCTGGGCCAGGTTTAGTGGTACACTTGTGGGTTCCTCTTATGCCCACTGCCCTTCCACTCTGCTCCATCCCAGCTCCGGGAGGTGCAGGGTGCAGCCTAGCCCTCCCCAGCTAGGGGAGTGGGCACCTGCTGTGTGGTGGCAGTGAGGGTTGGGGGTTCTGAGCTGCGTGGTGGCAGCTGCCACCGGCTGGAGTGGCTGGACTTGTGGCCCCGAGGCATGGCCAGTCCCAGCAGCTTCCCAACAGGGAAGGGCCCTGGAGCCCGGTGCTGCCCTGGCTGGTTTGGTGGCCCCATGCACTCCCCCTGGGTGCTGAGCTACCCCTGCCCCTGTGCATGTGGCTGTGGGCACAGAGCAGGCCTGGTGGCTGTGGCTGAGCTGTGGCCTCCTCTCTGCTGTGAGCAGATCTCAGATATCCAGGTGAGTGGGCAGTCGGAGGACATGACGGCCAAGGAGAAGCTGCTGCTGTGGTCGCAGCGAATGGTGGAGGGGTACCAGGGCCTGCGATGCGACAACTTCACCTCCAGCTGGAGAGACGGCCGCCTCTTCAATGCCATCATCCACCGGCACAAGTACATGGCCCCGGGGGCAGGGGGCTGTGGGCTGGGGGCACCCCCACAGCGCCTCCACTAGGCAACTTCAGACACGTTGGGGCAGGGAGGAGGGCAGGAGGAACAGGGATGAGTGCCAGCTGGCCGCCTCGGGGTGGTGGAGTTCAGGGCCCAGCCCCACCCTGGTGTCCTTCAGAAGCACCACCACTTGATCTCAAGACTGGAGGGCCCGCGGGCTGCTGGTCCTCACAGCCAGGTGCATGTGCCCTCTGTGGACGCCTCCGGAGGGGCCTGGTGGGGAGGGTGGCTGCAGGGAACCCCAGCTGCCTGGCACCCTCAGCTCTGCGCAGTCCTCTGTGCTGTGCCCCTGGGCTTCTGGGTGTGGGGGTGGCAGGGTGCTCTCAGCTCTGCCCATGTGACAGTGTATTTTTCAGGGTGAGTGTTGGGGGGGTCCATGGTTGCTGGGATGTGTGAGGAGCCGGCCTGTGCACCCGCCTGTGTGGTGTGGCCGCTGGTCTTGTCCCACCCACCTGACCAGCCACCTGCTTGTCCCAGGCCCCTGCTCATCGACATGAACAAGGTGTACCGGCAGACCAACCTGGAGAACCTGGACCAGGCCTTCTCTGTGGCGGAGCGGGACCTGGGAGTGACGCGGCTCCTGGACCCTGAGGGTACGTCTGCGTGGCCTTCCTCCCTGTCCCTTCCCCTGCTGCCTGCCCCAGAGCCACCGCTGAGCTGCCCTTCCCTGCAGACGTGGATGTCCCTCAGCCCGACGAGAAGTCCATCATCACCTACGTCTCGTCGCTGTATGACGCCATGCCCCGCGTGCCGGACGTGCAGGATGGGGTGAGGGCCAACGTGAGTGGGGGGCCCGGAGGGCAGGGGGCTTGGGCCTGGACGCCCCGACGACCCCTGACAGCCGCCCGTGCCTGCCCGCAGGAGCTGCAGCTGCGCTGGCAGGAGTACCGGGAGCTGGTGCTGCTGCTGCTTCAGTGGATGCGACACCACACGGCCGCCTTTGAGGAACGCAGGTTCCCCTCCAGCTTCGAGGAGATTGAGGTGGGCCTGCCGTGGGGGGCGGGGTCCTGGGCCTGCCCTGAGAGCCCGGCGTGGTTGCCGACAGCCTCGTCCCTGGCAGATCCTGTGGTCTCAGTTCCTGAAGTTTAAGGAGATGGAGCTACCAGCCAAGGAGGCCGACAAGAACAGGTCCAAGGGCATCTACCAATCCCTGGAGGTGAGTGGGACCGCTGGAGGGGTGGGTGGTGTCCTGGAACGCCCCAGGCCTGAAAGGTTCTTCCGGAGCCGACCTGGCCCTGCCTTTGGGACCAGGGCTGTCCCAGGTCTGGTGGGCTGGGGTCTGAAGGCCCAGCGCCCCACCCCCTGCCCCCTGCCTATAGGGCCTGAGTGTGGCCCCTGTCTACAGGGAGCGGTGCAAGCAGGCCAGCTCAAGGTGCCCCCTGGCTACCACCCGCTGGATGTGGAGAAGGAGTGGGGCAAGCTGCACGTGGCCATCCTGGAGCGGGAGAAGCAGCTCCGCAGCGAGTTTGAGAGGTGGGTGGGGCCCTGTGGTGGCAGGGGAACCACCCGAGGGTGTGTCACTGCCTGGGAAGGAAACCCCCCTCCCCGCCCCGCCCCCGGCGGGGGAACCCAAGCTCCCACTCGCCTTAGGACAGTGGGCCACAGCCAGGGAGACCGGAGCGGGAGGCGAGCTGCAGTCTGTGGCCAGGGCTGTGCCGGACCCGGCCAACGCGGCCCCTTCCCTGTGCTGCTGCTGCAGGCTGGAGTGTCTTCAGCGCATCGTGACCAAGCTGCAGATGGAGGCGGGGCTGTGTGAGGAGCAGCTGAACCAGGCCGACGCCCTGCTGCAGTCGGTGAGGGGGTGTGGGGCAGGCAGTGGGCGGGAGGGAGGCCGGAGGAGCCTGCCCCGCCCTGCAGCCCTAAAGCCAGGAGGGGGCTGTCCCCCTGGGCTCCTGGGGCAGGGGCAGGGGCAGGGGCGGGGCATGTGGTGGGGAATCCAGGCCTGAGCTCCTCCCTCCTCGTGTGCAGCTAACTCTCACTGCCTGCTTTGGGCAGAGTTGGTTTCTGGCCACGGGGCAGAACCCACGCTGGGCTGCCTTGCCCCCGCCAGGCCCCCTCCCCGACAGCGGCTTACTGTTAGGGGAGGGGTTGGGGGAGGGCCCAACCCACTGCTGATTGCCCAGGAGGACAGGGCCCACGTGGCATTACAGGAGGAGTTACCCAGGGTGGGTGATGGAGGATGAGGCCCAGGACCCCACAGACATGGTCATGGGGGTCTCGGTTCTTGGACTGGGCTAAGTACTCTGGACTTGGTCCCAATCCCCCAGAAGGCCAGTAGGAAGATGTGTGAGGTGGGCAGGGAGGGAAGGGGAAGGACAGGGATGGAGGCAGTGGCCCACTGAGGCTGAGGAAGCGGCTGAGGGTGGCCGTCTTGGCCGGGGCCCCTGTGAGGTCAGAGGGGGATCAGCTGTGCTCCGAGTCATGGCAACGACCTTGCAGGATGTCCGGCTGCTGGCTGCAGGCAAAGTGCCACAGCGGGCGGGGGAGGTGGAACGGGACTTGGACAAGGCGGATAGCATGATCCGGCTGCTCTTCAACGACGTGCAGACCCTCAAGGATGGACGGCACCCGCAGGGCGAGCAGATGTACCGCAGGTGGGCCCCGCCCTGCCCTCCCTGAAGCCCAGGTACACACGGCCCCAGGCCCGCCAACACCTACCTGAGTCCTCTGCTGCCCCAGGGTGTACCGTCTGCACGAGCGCCTGGTAGCCATCCGCACCGAGTACAACCTACGGCTGAAGGCAGGCGTGGCGGCCCCTGCAACCCAGGTGGCCCAGGTGACTCTGCAGAGTGTGCAGAGGCGCCCCGAGCTGGAGGACTCCACTCTGCGCTACCTGCAGGACCTGCTGGCCTGGGTGGAGGAGAACCAGCACCGTGTGGATGGCGCTGAGTGGGGTGTGGACCTGCCCAGCGTGGAGGCGCAGCTGGGCAGCCACCGAGGCCTGCACCAGTCCATCGAAGAATTCCGGGCCAAGATCGAGCGGGCACGGAGTGACGAGGTGGGTGGCGCTGAGCGATGGGCAGTGCGGGGGTGGGCCGGGCCCAGCCACTGCAGACCTCACCGTCTCATCTGTTGCAGGGCCAGCTCTCCCCCGCCACCCGGGGTGCCTACCGTGACTGCCTGGGTCGGCTGGACCTGCAGTACGCCAAGCTGCTGGTGAGTGGGGGCAGGGCCAGCCGGGGGAGGTGGGTAGCCCGCGGCCTGCTGACACGCACCCTCCTGCCCACAGAACTCCTCCAAGGCCCGCCTCAGGTCCCTGGAGAGCTTGCACAGCTTTGTGGCAGCCGCCACTAAGGAGCTAATGTGGCTGAATGAGAAGGAGGAGGAGGAGGTGGGCTTCGACTGGAGCGACCGCAACACCAACATGACCGCCAAGAAGGAGAGCTACTCGGTGAGCGGTGGCCCAGCTGCCCTGAACCCCACAGCCCCCTCCCCAGCTGTGCCCTGGCCGTCCCCTATGGTCAGAGACCAGTGGGGGCCCTGGGTCGAGTTTGCTGGGAGCGTGGCAGAGCCGGGTGTGGCCGGCCGTCCCTGAGACCCTTTGCTCTGGTGCCAGGCGCTGATGCGGGAGCTGGAGCTGAAGGAGAAGAAGATCAAGGAGCTCCAAAATGCTGGGGACCGGCTGCTGCGGGAGGACCACCCGGCCCGGCCCACGGTGGAGGTGGGGCTCCCTGGGTAGGGGCGGGGCTGCGGGGGGGGCCGTGCCGGGTCCCCAGGCGGGGCGGGGCGGGGCCTGACCGGGACCTGCTGTCCCGCAGTCCTTCCAGGCGGCCCTGCAGACGCAGTGGAGCTGGATGCTACAGCTGTGCTGCTGTATCGAGGCACACCTGAAGGAGAACGCTGCCTACTTTCAGGTGAGAACCGGAGCCCCCTCCGCACTGTGGCTCAGCGGCAGCCTTGCAGGAGCCCCTGGACCCCCTCGGCAGCTCCAATCAGGAGCCCTCCTGTCTGCAGACGTTGCCAGCTGCTGCCTGCAGCCTGACCGGGGCCTTGGACAGGGGTGCTCCCCAGGCCTCCTGGGGGCTTTGCCACTCCTTCCTCAGTGCCCCCGTCTTGGGCCTGCCCTGGGACAACTGTGGGCTGGGGCTCTGGTCCCAGGTAGCCTGGCGTGACCCCCTCCCGCTGCCCCAGTTCTTCTCAGATGTGCGGGAGGCCGAGGGGCAGTTGCAGAAGCTGCAGGAGGCACTGCGTAGGAAATACAGTTGTGATCGCTCCGCCACCGTCACCCGGCTGGAGGACCTGCTGCAGGATGCCCAGGTGAGGGAGGGGGTGTGCAGGGGCGTGTCAGGAGGGGCTGGCCTGGAGTCTGGGCTGCACGGGTACTCTGGAGACTAGGGGGCAACTGGAGGGGCCAGCAGGAGTTGGGAGGCACCATGGGAGGTGTAGAGGTCAGCCGAAGCGGGAATGAGGCAGGATGTTCTGGGCTGCATGGGTACTCTGGAGACTAGGGGGCAACTGGAGGGGCCAGCAGGAGTTGGGAGGCACCATGGGAGGTGTAGAGGTCAGCCAAGGGGGGAATGAGGCAGGATGTGGGGAGCCGAGAGGCAGCCGGTCCAAGCCGGGCCGCCCCCATCTCCATAGCCACAGAGTGAGTGGAAGCCTGAAGTCAGCAGCTGCCGCCAGCCCTTGTGCTGTAGAAGGGGCACCGCCAGCCCTTGTGCTATAGAAGGAGCACTGCCAGCCCTTGTACTATAGAAGGGGCACCAGCTGTTTGGCCTGAGATCTGGAGCGGGCAGAGTGGAACTGGGCTTTGTGCTCTGGGAGCAGGGGTGGGATCACCCAGGAGGGCGGGTGCACAGTGGGGAGTGACAGGGAAGAGGGACTGGAGACTGAAATGGCCTCTGAGGTCGGCAGGGCTGGGCCGGGGGGAGAGCCGGCAGCTCCCAGGTGTGTGCCGTCCTGCGGCCTGGCCCCCGCAGAGGGGTGGGATGGGAGGAGGCAGTACCTTCTGCAGCCCGCAGGGGAGGGGCAAGGGGCGAGGGCATTGTAGGGACCAGAGCGTAGCTGGGAGGACATGGTGGGTGAGCTGGGGTGAGAGGAGGGTGCCCTGTCTGGGTTGGGAAGGAGCGAGGGTGCATGGGTCTGCTTGGGGCCAACCCCCGGGGCCTGTCCCAGCTATACCTCCTGCTTCCAAGTGCTGCCACCCCGGGAGCATCAGGAGGGCCCCACAGGCCTGGTCCCGGGGTCTGGGGGTGCCTGGGGCAGGCCTGTGGGTCTCCATGGCCCTCGTCTGGATGCTGCCCCTCCCCCACAGGACGAGAAGGAACAGCTGAACGAGTACAAGGGCCACCTCTCAGGCCTGGCCAAGCGGGCCAAGGCCGTCGTGCAGCTGAAGCCCCGCCACCCAGCCCACCCCATGCGGGGCCGCCTGCCCCTGCTGGCCGTGTGCGACTATAAGCAGGTGGAGGTGAGCGCAGGCCGGGGGTCCACTGGGGGGCGTGGCCAGGCAGGGCAGGGTGTGGCCATGTCCTCACCGACTCTGCCCGTCCCAGGTGACTGTGCACAAGGGTGACGAGTGCCAGCTGGTGGGCCCTGCACAGCCGTCCCACTGGAAGGTGCTCAGCAGCTCCGGCAGCGAGGCCGCCGTGCCCTCCGTGTGCTTCCTGGTGCCCCCGCCCAACCAGGAGGCCCAGGAGGCCGTCACCAGGTGGGTGGCGGGGGCTCAGCAGGTGGGGGCTGGAAGGCGGGTGGGGCGCTGGTGACGCTGACTGTAGCCCTGACCTGCCAGGCTGGAGGCCCAGCACCAGGCCCTGGTCACGCTGTGGCACCAGTTGCACGTGGACATGAAGAGCCTTCTGGCCTGGCAGAGCCTTCGCCGCGACGTGCAGCTCATCCGCTCCTGGTCCCTGGCCACGGTACGCCTGCCCCCGAGCCGGGGGCTCTCTGGGTGGGAGGAGGGGAGAGGCGTTGTGCAGGGCACGGCCGCCTCGCCCGGCACTCAGCCCCACGTGCTTCCCCCAGTTCCGCACCCTGAAGCCAGAGGAGCAGCGCCAAGCCCTGCACAGCCTGGAGCTGCACTACCAGGCCTTCCTGCGGGACAGCCAGGACGCGGGCGGCTTCGGACCCGAGGACCGGCTGATGGCTGAGCGCGAGTACGGCTCCTGCAGCCACCACTACCAGCAGCTGCTGCAGAGCCTGGAACAGGGTAGGGCACGGCAGGGCTGGGGCGGTTGGGCTGGTGCGAGGTGGGCGGTGGAGTGACCCACATGTGTTCCCCAGGTGCACAGGAAGAGTCTCGCTGCCAGCGCTGCATCTCCGAGCTCAAAGACATCCGGCTGCAGCTGGAGGCCTGTGAGACGCGCACCGTGCACCGCCTGCGGCTGCCGCTGGACAAAGAGCCGGCACGGGAGTGTGCCCAGCGCATCGCCGAGCAGCAGGCAGGGGCCGTCCCCCCCCATCCAGTCCCATCCCTCTCCCCATCCCTCCTCCCACCCTCCCTCCTTTGGCCTTCAAGGGGCGCTGTGTGCTGCGCTCGGTGATGAGTGCGTTCCCACTCCCTGTCTTTGCAGAAGGCACAGGCAGAGGTGGAGGGGCTGGGCAAGGGGGTCGCCCGGCTCTCTGCCGAGGCCGAGAAGGTCTTGGCCCTACCAGAGCCATCGCCTGCGGCCCCCACGCTGCGCTCGGAGCTGGAGCTGACGCTGGGCAAGCTGGAGCAGGTCCGCAGCCTGTCTGCCATCTACCTGGAGAAGTGAGTGCACCTGGCCACAGGCGAGGGGCTGGGGTCGGCTGGGGGGTGCTGGGGTGAGGGCTGTGACCTTGCAGGAGGGTGCAGGAGGGAACGAGTTGGGGGCTGTTCAGAGATCTGGCCAGGAGGCGGGAGGCCCAGCTGGGGTGGTGGCCGCAGAGGTGGTGAGAAGAGTTCAGGGCTGGATACGCTTCGACAGTGGCGGCCGGGGGACTTCCTGGTGATGAGATTCGGGTGTGGGGAAGAGACAGTGGAGCTGGGCAGCGGCTGCTGAGTGAGGTGGGGAAGCTGTGGATCCACTTTCAGTGGCACAGTAAGGAATCCTGCACCCTGCCTCTCGTCTGTCCTGTTCCTGGCCTGGACTTAAGGGCCTCTGGCATTTATTTCCAATGTGTTATGTCATAGTCTCTTCCTGTGTTGCAAACCACAGGTCCACCCGCTGTCTACTCCTCACAGAACCAGCACAGTGATAGGGCCGCCGCCTCTTCCTGTGTTGCAAACCACAGGTCCACCCGCTGTCTACTCCTCACAGAACCAGCACAGTGATAGGGCCGCCACCTCTTCCTGTGTTGCAAACCACAGGTCCACCCGCTGTCTACTCCTCACAGAACCAGCACAGTGATAGGGCCGCCGCCTCTTCCTGTGTTGCAAACCACAGGTCCACCCGCTGTCTACTCCTCACAGAACCAGCACAGTGATAGGGCCGCCGCTGGCTACCACAGGGTGCCATTTCCACCCCGGTGTCCCCTTCGCCCAGTCTTGGGCTGCAAGGGCTGAGAACTACGCTTCCAGGCCCTGTGGGCACCAGGATGCCGCTGTCATCCAGCCAGTGGGCTCGTCCTGTCTCGGGGCCTTGGGGTCTGCCGTTCCTGGCTGGCGCACCCCCTCTCCAGGCTGGGTCCTGAGTGTCCTTGTGTTACAGAGGCTCCCAACCTCCTTCTCCCCAAGGCTGTTCTTCTCCTGAAGCAAGCCTCTGACCACAGGGTCAGCTGACCCCCAGGGTTGGGTCTGGGCTTGGCAGGCAGCACCCTGGGTCGCTGTGGCTTGGGTTCCATTCCCTTGGGCTATTGCTCGAGCCCCATGTCATGGCCCTGAGCCCCGCTTTCCCGCCAGGCTCAAGACCATCAGCCTGGTGATCCGCGGCACGCAGGGGGCCGAGGAGGTGCTCAGGGCCCACGAGGAGCAGCTCAAGGAGGCCCAGGCCGTGCCGGCCACCCTCCCGGAGCTCGAGGCCACCAAGGCCTCTCTGAAGGTATCGTTTCGAGGCTTGGGCTTCATGGCGGGGTGGGGGTACAGCGGGCCCAGCTGAAGCCGCACATGTCCCTCCTGTCCCCAACAGAAGCTGCGGGCCCAGGCCGAGGCACAGCAGCCCACGTTCGACGCCCTGCGGGATGAGCTGCGGGGGGCACAGGAGGTGGGGGAGCGACTGCAGCAGCGGCACGGGGAGCGGGACGTGGAGGTGGAGCGCTGGCGGGAGCGGGTCGCCCAGTTGCTTGAGCGCTGGCAGGCTGTGCTGGCCCAGACCGACGTGCGGCAGCGCGAGCTCGAGCAACTGGGCCGCCAGCTGCGTTACTACCGCGAGAGTGCAGACCCCTTGGGCGCCTGGCTGCAGGACGCCAGGCGGCGGCAGGAGCAGATCCAGGCCATGCCGCTGGCCGACAGCCAGGCTGTGCGGGAGCAGCTGCGGCAGGAGCAGGTGGGCTTGGGTCGGTGGGGACGGGGCGGCTGGGCGTGCCCTGCGGCCACGGCTCTGACCACACCATCACCCAGGCCCTGCTGGAGGAGATCGAGCGCCACGGCGAGAAGGTCGAGGAGTGCCAGAGGTTTGCGAAACAGTACATCAACGCCATCAAGGTGAGGCCCAGCCGCCTGCACCAGGCCCCAAGTCCGGGACCGTTGCCAGGATGATGGCGGGCTTTCCCTGAGCCTGGGCCATGCCAGGGAAAGCCCATGTGAGGGTTTAGGGGCTGGGCAGGGGCCATCGGAGGGCAGAGCTGGTGGCTGACCGCAGTGCATTGCCACAGGACTATGAACTCCAGCTGGTGACGTACAAGGCGCAGCTTGAGCCGGTGGCCTCCCCGGCCAAGAAGCCCAAGGTCCAGTCGGGATCAGAGAGTGTCATCCAGGAGGTAGGGTGGGGCCGAACCTAACTGGGTGAGGGGCTGGCAGGCCTCTGGCTGGAGCTGCAGCTCTCGCAGGGCTAACCCTGGCTCTACTCCACAGTACGTGGACCTGCGTACGCACTACAGCGAGCTGACCACACTGACGAGCCAGTACATCAAGTTCATCAGCGAGACTCTGCGGCGCATGGAGGAGGAGGAGGTACAGCCCGTTGGGCGGAGGCTGGGCAGAGGGTTCCATCTCACCACCTGTTGTGTCCCACACACAGCCACAGGAGCTGCCTGAGGCCACCTCTCGTGGCCCTCCCCACTCTCCCCACTGGCGCTGCCAGCACCCCCTGCCCCCAGCTCAGCCTGCCCCAGGCTGCCCCACCTGTGGCCTGCCCCCCTGGCCTGGTGTGGCTACACTGCTCCCCCACAGTGGCTGGTGCCGCCTCTCCTCCCTCGTGAGCGTCCCCCCTGCAGCCTAGCGGCCGTCCTGTGGGCTCTTCCTTGGCGTTCTGCGTTGGCCTTTCCGCTCAACCCTTTGCTGCCAGAAGTCGGGGGTGCTGAGTTGTGGGGCTAGGGAGGGTCTTCCAAGGCACCCATCTGTTGGCTTCTCCCTACCTTCTGCGTGGCCCTGAATCCTCTTCCGAGTTGGCTCGGGTCTGCACTGCTGTCTCCACGCTGGCCGCCTCGCCTCACCCCGCCCCACCCAGGCTTCCCACCCTGGTCGGGGAGAGGGGTCCAGTGGTGGGAGCCAGGCCCACTGGTGTCCAGCAGAGCTCAGGCAGGGCTGTGTGCTGGGTGTGGCTCGCAGGTTCCGAGCTCCTGCTTTGCTCTCTCTCTCCCCGTCCGTCTGTCTCCGTCGCCTGTCTGCGGGAAGCAGGGAGGACGCCTGGGCCTCCTTCCCCAGGCTGGTGAGCACAGTGGGGCCGGCTGCGATCGCTGCCCGAGCGCCCTGGGCGGGTGCACGCTGTCTCCTCCGCCGGGCTGGGGCCTCTGTCTTCCCCGACCCCTGCTCCGCGCTGTCTGAGTGAACTGTGCCGGTGCGTGCCCCGTCAGCGCCTGCCCGTGTTCACACTCTCTCTGCTTCTCTTCTTCTCTCTGCTGTGGCCACAGAGGCTGGCTGAGCAGCAGCGGGCAGAGGAGCGCGAGCGGCTGGCCGAGGTGGAGGCCGCGCTGGAGAAGCAGCGGCAGCTGGCCGAGGCGCACGCCCAGGCAAAGGCACAGGCGGAGCGGGAGGCGAAGGAGCTGCAGCAGCGCATGCAGGAGGAGGTGGTGCGGCGGGAGGAGGCGGCGGTGGACGCGCAGCAGCAGAAGCGCAGCATTCAGGAGGAGCTGCAGCAGCTGCGGCAGAGCTCGGAGGCGGAGATCCAGGCCAAGGCCCGGCAGGCAGAGGCGGCTGAGCGCAGCCGGCTGCGCATCGAGGAGGAGATCCGCGTGGTGCGCCTGCAGTTGGAGGCCACCGAGCGCCAGCGTGGCGGGGCTGAGGGGGAGCTGCAGGCACTGCGTGCACGGGCGGAGGAGGCTGAGGCACAAAAGCGACAGGCGCAGGAGGAGGCCGAGCGCTTGCGGAGGCAGGTGCAGGACGAGAGCCAGCGTAAGCGGCAGGCGGAGGTGGAGCTGGCCTCGCGCGTGAAGGCCGAGGCCGAGGCGGCGCGCGAGAAGCAGCGGGCCCTGCAGGCCCTGGAGGAGCTGCGGCTGCAGGCGGAGGAGGCGGAGCGGCGCCTGCGGCAGGCCGAGGTGGAGCGAGCGCGGCAGGTACAGGTGGCCCTGGAGACGGCGCAGCGCAGTGCAGAGGCGGAGCTGCAGAGCAAACGCGCCTCCTTCGCCGAGAAGACGGCACAGCTGGAGCGCTCCCTGCAGGAGGAACACGTGGCTGTGGCACAGCTGCGGGAGGAGGCTGAGCGGCGGGCACAGCAGCAGGCCGAGGCCGAGCGGGCGCGCGAGGAGGCAGAGCGGGAGCTGGAGCGCTGGCAGCTCAAGGCCAACGAGGCGCTACGGCTGCGGCTGCAGGCGGAGGAGGTGGCGCAGCAGAAGAGCCTGGCGCAGGCCGAGGCTGAGAAGCAGAAGGAGGAGGCGGAGCGCGAGGCGCGGCGGCGCGGCAAGGCGGAGGAGCAGGCCGTCCGGCAGCGGGAGCTGGCTGAACAAGAGCTGGAGAAGCAGCGGCAGCTGGCGGAAGGCACCGCGCAGCAGCGCCTGGCCGCGGAGCAGGAGTTGATCCGGCTGCGGGCCGAGACGGAGCAGGGGGAGCAGCAGCGGCAGCTGCTGGAGGAGGAGCTGGCCCGGCTGCAGCGTGAGGCGGCTGCAGCCACGCAGAAACGGCAGGAGCTGGAAGCCGAGCTGGCCAAGGTGCGGGCCGAGATGGAGGTGCTGCTGGCCAGCAAGGCGAGGGCTGAGGAGGAGTCGCGCTCCACCAGCGAGAAGTCCAAGCAGAGGCTGGAGGCCGAGGCCGGCCGGTTCCGCGAGCTGGCCGAGGAGGCCGCCCGCCTGCGTGCCCTGGCGGAAGAGGCCAAGCGGCAGCGGCAGCTGGCCGAGGAAGACGCGGCGCGGCAGCGGGCCGAGGCGGAGCGGGTGCTTGCGGAGAAGCTGGCCGCCATCGGCGAGGCCACGCGGCTCAAGACGGAGGCGGAGATCGCGCTCAAGGAGAAGGAGGCGGAGAACGAGCGCCTGCGGCGGCTGGCGGAGGACGAGGCCTTCCAGCGGCGGCGGCTGGAGGAGCAGGCCGCGCAACACAAGGCTGACATCGAGGAGCGCCTGGCCCAGCTGCGCAAGGCATCGGACAGCGAGCTGGAGCGGCAGAAGGGGCTGGTGGAGGACACGCTGAGGCAGCGGCGGCAGGTGGAGGAGGAGATCCTGGCGCTGAAGGCGAGCTTCGAGAAGGCGGCCGCTGGCAAGGCGGAGCTGGAGCTGGAGCTGGGACGCATCCGCAGCAACGCGGAGGACACGCTGCGCAGCAAGGAGCAGGCCGAGCTGGAGGCTGCGAGGCAGCGGCAGCTGGCGGCGGAGGAGGAGCGGCGGCGCCGTGAGGCTGAGGAGCGCGTGCAGAAGAGCCTGGCGGCCGAGGAGGAGGCCGCACGGCAGCGGAAGGCGGCGCTGGAGGAAGTCGAGCGGCTGAAAGCCAAGGTGGAGGAGGCGCGGCGCCTGCGGGAGCGAGCGGAGCAGGAGTCGGCGCGGCAGCTGCAGCTGGCCCAGGAGGCCGCCCAGAAGCGGCTGCAGGCGGAAGAGAAGGCACACGCCTTCGCGGTGCAGCAGAAGGAGCAGGAGCTACAGCAGACGCTGCAGCAGGAGCAGAGCGTGCTGGACCAGCTGCGCGGCGAGGCGGAGGCGGCCCGGCGGGCGGCTGAGGAGGCGGAGGAGGCCCGGGTGCAGGCGGAGCGTGAGGCGGCGCAGTCCCGGCGGCAGGTGGAAGAGGCCGAGCGGCTGAAGCAGTCGGCAGAGGAGCAGGCACAGGCCCGGGCTCAGGCACAGGCGGCTGCAGAGAAGCTGCGCAAGGAGGCCGAGCAAGAGGCGGCGCGGCGGGCACAGGCGGAGCAGGCGGCCCTGCGGCAGAAGCAGGCAGCTGACGCGGAGATGGAGAAGCATAAGAAATTCGCCGAGCAGACGCTGCGGCAGAAGGCGCAGGTGGAGCAGGAGCTGACAACACTGCGGCTGCAGCTGGAGGAGACCGACCACCAGAAGAACCTGCTGGACGAGGAGCTGCAGCGGCTGAAGGCGGAGGCCACGGAGGCCGCACGCCAGCGCAGCCAGGTGGAGGAGGAGCTCTTCTCGGTGCGCGTGCAGATGGAGGAGCTGAGCAAGCTCAAGGCACGCATCGAGGCTGAGAACCGCGCACTCATCTTGCGTGACAAGGACAATACGCAGCGCTTCCTGCAGGAGGAGGCTGAGAAGATGAAGCAGGTGGCGGAGGAGGCCGCGCGGCTGAGTGTGGCGGCCCAAGAGGCTGCGCGACTGCGGCAGCTGGCAGAGGAGGACCTGGCACAGCAGCGGGCCTTGGCAGAGAAGATGCTCAAGGAGAAGATGCAGGCGGTGCAGGAGGCCACGCGACTCAAGGCTGAGGCGGAACTGCTGCAGCAGCAGAAGGAGCTTGCGCAGGAGCAGGCGCGGCGGCTGCAGGAGGACAAGGAGCAGATGGCGCAGCAGCTGGCGGAGGAGACGCAGGGCTTCCAGCGGACGCTGGAGGCCGAGCGGCAGCGGCAGCTGGAGATGAGCGCTGAGGCTGAGCGCCTCAAGCTGCGTGTGGCCGAGATGAGCCGAGCCCAGGCCCGCGCTGAGGAGGACGCCCAGCGCTTCCGGAAGCAGGCGGAGGAGATCGGTGAGAAGCTGCACCGCACGGAGCTCGCCACCCAGGAGAAGGTGACCCTGGTGCAGACACTGGAGATCCAGCGACAGCAGAGTGACCATGATGCCGAGCGCCTGCGGGAGGCCATCGCTGAGCTGGAGCGTGAGAAGGAGAAGCTCCAACAGGAGGCCAAACTGCTGCAGCTCAAGTCTGAGGAGGTACCGCCCCCTCTACGTGCGACGGGCGGGTGGGCCCGGGGATCTGCTTTGGTGGGTGATGGGTGCTCCTGGGCTGGCGGTCCCTGATCACACCCTCTTCTTCTGCAGATGCAGACGGTGCAGCAGGAGCAGCTGCTGCAGGAGACGCAGGCCCTGCAGCAAAGCTTCCTCTCTGAAAAGGACAGCCTGCTACAGCGGGAGCGCTTCATCGAGCAGGAGAAGGCCAAGCTGGAGCAGCTCTTCCAGGACGAGGTGGCCAAGGCACAGCAGCTGCGTGAGGAGCAGCAGCGGCAGCAGCAGCAGATGGAGCAGGAACGGCAGCGGCTGGTGGCCAGCATGGAGGAGGCGCGGCGGCGGCAGCATGAGGCCGAGGAGGGCGTGCGGCGCAAGCAGGAGGAGCTGCAGCAGCTGGAGCAGCAGCGGCGGCAGCAGGAGGAGCTGCTGGCTGAGGAGAACCAGAGGCTGCGTGAGCAGCTGCAGCTCCTGGAGGAGCAGCACCGGGCCGCGCTGGCGCACTCAGAGGAGGTCACTGCCTCGCAGGTGGCTGCCACAAAGACCCTGCCCAATGGCCGGGATGCACTTGATGGCCCCGCGGCAGAGGCAGAGCCGGAGCACAGCTTCGATGGCCTGCGGCGGAAGGTGTCAGCTCAGAGGCTGCAGGAGGCCGGCATCCTGAGTGCGGAGGAGCTGCAGCGGTTGGCGCAGGGCCACACCACGGTGGACGAGCTCGCACGGCGGGAAGACGTGCGCCACTACCTGCAGGGCCGCAGCAGTATCGCAGGGCTGTTGCTGAAGGCCACCAATGAGAAGCTGAGTGTTTACGCCGCCCTGCAGAGGCAGCTGCTGAGTCCCGGCACGGCCCTCATCCTGCTGGAGGCGCAGGCGGCCTCAGGCTTCCTGCTGGACCCTGTGCGGAACCGGCGGCTGACCGTCAACGAGGCTGTGAAGGAGGGTGTGGTGGGCCCCGAGCTGCACCACAAGCTGCTGTCGGCCGAGCGCGCCGTCACTGGCTACAAGGACCCCTACACTGGCCAGCAGATCTCTCTCTTCCAAGCCATGCAGAAGGGCCTCATCGTCCGGGAGCACGGCATCCGCCTGCTGGAGGCCCAGATCGCCACGGGCGGCGTTATCGACCCCGTGCACAGCCACCGCGTGCCCGTGGACGTGGCCTACCGGCGCGGCTACTTCGACGAGGAGATGAACCGCGTCCTGGCGGACCCCAGCGACGACACCAAGGGCTTCTTTGACCCCAACACGCACGAGAACCTCACGTACCTGCAGCTACTGGAGCGCTGCGTGGAGGACCCCGAGACGGGCCTGTGCCTTCTGCCACTCACGGATAAGGCTGCCAAGGGCGGGGAGCTGGTCTACACTGACTCCGAGGCCCGGGACGTCTTTGAGAAGGCCACCGTGTCTGCGCCGTTCGGCAAGTTCCAGGGCAAGACGGTGACCATTTGGGAGATCATCAACTCGGAATACTTCACGGCAGAGCAGCGGCGGGACCTGCTGCGGCAGTTCCGCACGGGCCGGATCACAGTGGAGAAGATCATCAAGATCATCATCACGGTGGTGGAGGAGCAGGAGCAGAAGGGCCGGCTTTGCTTTGAGGGCCTGCGCAGCCTGGTGCCAGCCGCCGAGCTGCTGGAGAGCAGGGTCATCGACCGCGAGCTCTACCAGCAGCTGCAGCGAGGTGAGCGCTCTGTGCGAGACGTAGCCGAGGTGGACACTGTGCGGCGGGCTCTCCGGGGTGCCAACGTCATCGCGGGTGTATGGCTGGAGGAGGCGGGGCAGAAGCTGAGTATCTACAATGCCCTGAAGAAAGACCTGCTGCCATCCGACATGGCCGTGGCCCTGTTGGAAGCCCAGGCCGGCACCGGGCACATCATCGACCCCGCCACCAGCGCCCGGCTGACCGTGGACGAGGCAGTGCGTGCTGGCCTGGTGGGCCCCGAGTTTCATGAGAAGCTGCTATCAGCCGAGAAGGCTGTGACAGGGTACAGGGACCCCTACACAGGGCAGAGCGTCTCCCTGTTCCAGGCCCTGAAGAAGGGCCTCATTCCCCGGGAGCAGGGCCTGCGCCTGTTGGACGCCCAGCTGTCCACGGGCGGCATCGTGGACCCCAGCAAGAGCCACCGCGTGCCCCTGGATGTCGCCTGCGCCCGAGGCTGCCTGGATGAGGAGACCAGCAGGGCCCTGTCGGCACCAAGGGCCGACGCCAAGGCCTACAGTGACCCCAGCACAGGGGAGCCGGCCACCTACGGCGAGCTCCAGCAGCGGTGCCGGCCCGACCAGCTGACCGGGCTGAGCCTGCTGCCGCTCTCAGAAAAGGCTGCTCGGGCCCGGCAGGAGGAGCTCTACTCAGAGCTGCAGGCCCGTGAGACCTTTGAAAAGACCCCGGTTGAGGTCCCCGTGGGTGGCTTCAAGGGCAGGACGGTGACGGTGTGGGAGCTCATCAGCTCTGAGTACTTCACTGCGGAGCAGCGGCAGGAGCTGTTGCGTCAGTTCCGCACGGGCAAGGTCACCGTGGAGAAGGTCATCAAGATTCTCATTACCATCGTGGAGGAGGTGGAGACCCTGCGGCAGGAGAGGCTGTCCTTCAGCGGCCTCCGTGCCCCTGTGCCAGCCAGCGAGCTCCTGGCTTCCGGGGTCCTCAGCAGAGCCCAGTTTGAGCAGCTCAAGGACGGCAAGACGACGGTCAAGGACCTTTCGGAGCTGGGCTCCGTGCGGACGCTGCTGCAGGGCAGTGGCTGCCTCGCCGGCATCTACCTGGAGGACACCAAGGAGAAGGTGTCCATCTACGAGGCCATGCGCCGGGGCCTGCTGAGAGCCACAACGGCTGCGCTCCTGCTGGAGGCGCAGGCGGCCACTGGCTTCCTGGTGGACCCCGTGCGGAACCAGCGCCTGTATGTCCACGAGGCCGTGAAGGCGGGCGTGGTGGGCCCCGAGCTTCACGAGCAGCTGCTGTCTGCCGAGAAGGCCGTCACCGGCTACAGAGACCCCTACTCGGGCAGCACCATCTCCCTCTTCCAGGCCATGCAGAAGGGCCTGGTTCTCCGGCAGCACGGCATCCGCCTGCTGGAGGCCCAGATCGCCACGGGCGGCATCATCGACCCCGTGCACAGCCACCGCGTGCCTGTGGACGTGGCCTACCAGCGCGGCTACTTCAGTGAGGAGATGAACCGCGTCCTGGCGGACCCCAGCGACGACACCAAGGGCTTCTTTGACCCCAACACGCATGAGAACCTCACGTACAGGCAGCTGCTGGAGCGGTGCGTGGAGGACCCCGAGACGGGCTTGCGCCTTCTGCCACTGAAAGGGGCGGAGAAGGCTGAGGTGGTGGAGACCACGCAGGTGTACACTGAGGAGGAGACAAGAAGGGCATTTGAAGAGACACAGATCGACATTCCCGGCGGCGGCAGCCACGGCGGCTCCACCATGTCCCTGTGGGAGGTGATGCAGTCGGACCTGATCCCCGAGGAGCAGCGGGCCCAGCTGATGGCTGACTTCCAGGCCGGCCGGGTGACCAAGGAACGCATGATCATCATCATCATCGAGATCATTGAGAAGACAGAGATCATCCGCCAGCAGGGTCTGGCCTCCTACGACTACGTGCGCCGCCGCCTCACGGCTGAGGACCTGTTCGAGGCTCGGATCATCTCTCTCGAGACCTACAACCTGCTCCGGGAGGGCACCAGGAGCCTCCGTGAGGCTCTCGAGGCGGAGTCCGCCTGGTGCTACCTCTATGGCACGGGCTCCGTGGCTGGTGTCTACCTGCCCGGTTCCAGGCAGACACTGAGCATCTACCAGGCTCTCAAGAAAGGGCTGCTGAGTGCCGAGGTGGCCCGCCTGCTGCTGGAGGCACAGGCAGCCACAGGCTTCCTGCTGGACCCGGTGAAGGGGGAGCGGCTGACTGTGGATGAGGCTGTGCGGAAGGGCCTCGTGGGGCCCGAGCTGCACGACCGCCTGCTCTCGGCTGAGCGGGCGGTCACCGGCTACCGTGACCCCTACACCGAGCAGACCATCTCGCTCTTCCAGGCCATGAAGAAGGAGCTGATCCCTACTGAGGAGGCCCTGCGGCTGCTGGATGCCCAGCTGGCCACCGGCGGCATCGTGGACCCCCGCCTGGGCTTCCACCTTCCCCTGGAGGTGGCTTACCAGCGTGGCTACCTCAACAAGGACACGCACGACCAGCTGTCAGAGCCCAGCGAGGTGCGCAGCTACGTGGACCCGTCCACCGACGAGCGCCTCAGCTACACGCAGCTGCTCAGGCGGTGCCGTCGTGACGACGGCACCGGCCAGCTGCTCCTGCCACTGTCGGACGCCCGCAAGCTGACCTTCCGTGGCCTGCGGAAGCAGATCACCATGGAGGAGCTGGTGCGCTCGCAGGTCATGGACGAGGCCACGGCGCTGCAGCTGCGGGAGGGCCTGACCTCCATCGAGGAGGTCACCAAGAACTTGCAGAAGTTCCTGGAAGGCACCAGCTGCATCGCTGGTGTCTTCGTGGACGCCACCAAGGAACGGCTCTCGGTGTACCAGGCCATGAAGAAGGGCATCATCCGCCCCGGCACAGCCTTTGAGCTCCTGGAGGCGCAGGCGGCCACCGGTTACGTCATCGACCCCATCAAGGGACTGAAGCTGACGGTGGAGGAGGCTGTGCGTATGGGCATTGTGGGCCCCGAGTTCAAGGACAAGCTGCTGTCGGCCGAGCGCGCCGTCACTGGGTACAAGGACCCCTACTCTGGGAAGCTCATCTCCCTCTTCCAGGCCATGAAGAAGGGCCTGATCCTGAAGGACCATGGCATCCGCCTGCTGGAGGCCCAGATCGCCACGGGCGGCATCATCGACCCTGAGGAGAGCCACCGGCTGCCCGTGGAGGTGGCCTACAAGCGCGGCCTCTTCGATGAGGAGATGAACGAGATCCTGACCGACCCCTCGGACGACACCAAGGGCTTCTTTGACCCTAACACGGAGGAGAACCTCACCTACCTGCAGCTGATGGAGCGTTGTATCACTGACCCCCAGACGGGCCTGTGTCTCTTGCCGCTGAAGGAGAAGAAGCGGGAGCGGAAGACGTCCTCCAAGTCCTCCGTGCGCAAGCGCCGAGTGGTCATCGTGGACCCCGAGACGGGCAAGGAGATGTCAGTGTACGAGGCCTACCGCAAGGGCCTGATTGACCACCAGACGTACCTGGAGCTGTCCGAGCAGGAGTGCGAGTGGGAGGAGATCACCATCTCCTCCTCGGACGGCGTGGTCAAGTCCATGATCATCGACCGCCGCTCCGGGCGCCAGTACGACATCGATGATGCCATCGCCAAGAACCTCATCGACCGCTCGGCACTGGACCAGTACCGCGCCGGCACGCTCTCCATCACCGAGTTCGCCGACATGCTCTCGGGCAACGCCGGTGGTTTCCGCTCCCGTTCCTCCTCGGTGGGATCCTCCTCCTCCTACCCCATCAGCCCCGCCGTCTCCAGGACCCAGCTGGCCTCCTGGTCAGACCCCACTGAGGAGACGGGCCCCGTGGCTGGCATCCTGGACACGGAGACGCTGGAGAAGGTGTCCATCACCGAGGCCATGCACCGGAACCTGGTGGATAACATCACGGGGCAGCGGCTGCTGGAGGCGCAGGCCTGCACCGGGGGCATCATCGACCCCAGCACCGGTGAGCGCTTCCCTGTCACCGACGCCGTCAACAAGGGCCTGGTGGACAAGATCATGGTGGACCGCATCAACCTGGCCCAGAAGGCCTTCTGCGGCTTCGAGGACCCACGCACCAAGACCAAGATGTCGGCCGCCCAGGCCCTGAAGAAGGGCTGGCTCTACTACGAGGCCGGCCAGCGCTTCCTGGAGGTGCAGTACCTGACCGGCGGCTTGATCGAGCCCGACACGCCGGGCCGCGTGCCCCTGGACGAGGCCCTGCAGCGCGGCACGGTGGACGCCCGCACCGCACAGAAGCTGCGTGACGTGGGCGCCTACTCCAAGTACCTCACCTGCCCTAAGACCAAGCTCAAGATCTCCTATAAGGACGCGCTGGACCGCAGCATGGTGGAGGAGGGCACGGGGCTGCGGCTGCTGGAGGCTGCCGCGCAGTCCACCAAGGGCTACTACAGCCCCTACAGCGTCAGCGGCTCCGGCTCTACCGCTGGCTCCCGCACCGGCTCGCGCACCGGCTCCCGGGCCGGCTCCCGCCGCGGCAGCTTTGACGCCACCGGCTCCGGCTTCTCCATGACCTTCTCTTCATCCTCCTACTCCTCCTCGGGCTACGGCCGCCGCTACGCCTCGGGGTCCTCGGCCTCCCTGGGGGGCCCTGAGTCTGCCGTGGCCTGAGGCTGCCTGCGCCCACCCCGCTCTGCATGCGGCCCAGCCCGGCTCCCACCGAGGCGCGGGGGCCGTTTTCAACGCTTAAAGGTGTCTTCCTCCCAAGTGGTGCCTAAAGTTTAACCAAAAAGACCAGACTAATATATTAATATATATCTGCTGTCCAGACAGCCTGTATCTTGGGGGACAGGGCTGGCCCAGCCCTGCTGGCCGCCTCACCCCCTCGGGTCTCCTCACTCCCTTCTACCTGCCACTCACACAGCCAGGTGCCTTGGAGGGTCCCAAGCTGGGCCCCAGCCCACCCTCCTGTCTTCCCAGGGTAGCCCGCCTGCCAGTCCTAGCTGCACAGGGCAGCTGGGCCCAACCCTGTCTGTAGAGGGCCCTGGTGTTTCTAGCACTGGCCTGCACGGTGGGCCTTGCTGGGGACGGGGGGCCCCAGTCAGCCTCTCTCCCAGTCTACCCAGAGAAGCCCCTTCCCCATGGGAAGACGAGGCCCTCGGGCCCAGCCCCCACAGTGCTGTCTGATCTGTGCTTTCCAGCTCACCCCCCACACTCACTCCTGAGACCCCTGGCCTCCGGCGTCAGCCTCCAGCCTCTGTTCCCCTAGTAAGTGCCTTCCATGTCGGCCTCTAACCCCAGGCCCCGAGGACCCAGACCCAGTGGGGAGGCGGACGTTCCAGCCGGCATGGCTGGGAACTGCAGACCTGTCCTCCTGGTGGGTCCAGGGGCCCCTCCAGCTTGTGGAGCCCCACACTGGGGTGCCGCCTGCCCGTCTCTCTCCCATGGAGCCCCAGCCCCCTTTGGGCCCAGGGACACCAGCCAGGCTCTGTGCTGACCCTCCTGTTGCACCCAGCCCTGGTCTCAGCAGCGACCACCCCTGCCTCCACCCTCTGAGCTTTGCATGTTCCACTAACCCCGGGCGGGTGGCAGGTGGAGGTGTCAGGCTGCTGGCGCCTCTGCAAGGGCAGAACACTAACCTGACCGTGGGCGGGGCCTTGCGGTATCCGCCCCCAATAAAAGCAATTCCAACCTTCCCGTGCCTCCGGCCTTGTGTCTTGGGGTCAGGTGTGTGTCAGGGACAGGGTGGACATTGCAGCCCCAGGCTCCAGGCTCTGAGCTCAGCCACTATGGCCAGTCAGGGACGTACGTCTGTGTGCAGCAGAGACCAGGTGCTCGCAGGAGGGCAGCTGAGAGACCCTGGGCAGAGGGATTCAGTGTGGGGCAGGGTGGTGGCCACACTGAGGCCGGGCCCCCTGGGACCTGCTGCTGGTCAGGTGCTGACCAGTCTCCCAGCCCCACCCTTCGCACTATAAGGGGCTCACGTTTCCTTATCTGAGGCGGGGTGTGGTATGCTCCCATATCAGTCACTGGCTGTGGCCCCTTGGCCTCCGGGGAGGGTGCCACATGGCCCCGGGTCAGTTGGTTGCTGCTGGCCGGGCAGCCCTCCAGAGAAGGGGCCACAGGCAGTGGGCCTAGTATGAGGGCCTCCACCACAGTCTGCCATGTCTCGGCCCCATGCCCATCACACTCCGGGGATTCCATCTGGATGGGCTTCTCCAGGCTTCCGACTGGTGGCAGTTTCTGGGAAGACTTAGGGGAGGAGGGAAGCGGGGCCACCCCAGCCTGCAGTAGCCACTGGTCCAGGCTCAGCCGATACTCTTTTTTTTTTTTTTTTTTTTTTCAGGCAGAGTCTTGCTTTGTCACCCAGGCTGGAGTGCAGTGTCATGATCTCGGCTCACTGCAGTCTCCACCTCCCATGTTCAAATGATTCTACTGCCTCAGCCTCCCGAGTAGCTGGGACTACAGGTGTGCACCCAGCTAATTTTCATATTTTTAGTAGAGATGGGGTTTCACCGTGTTGGCCAGGCCGGTCTCGAACTCCTGGCCTCAAGTGATCCTCCCTGCCTTGGCCTCCCAAAGTTCTGGGATTACAGGCGTGAGCCACTGTGCCTGGCCTTCGCCTCTCCTGCTGGTGGGACCCAGGCGTTCACCCTGGGGTCCAAGTCCTAGTCTTCTCGCTCTTGCTGGGCTTTACCCTCCCTGCCCCGGCCCGGTAGAGGGTGCTCTGCCTGCTGGTCCGCTGGAACAGGAGTGGCCCCGGCCGGGTGACCCACAGCCATCGAGCGGGGCAGTGCCTTACTCTGTCCCAGGTGGCTGGGTTTTAGGACCTCTGAGCCCTCACAGCTTAAGAGTTGCAGGGAAAGGAAGCACAGGTTGTCAGTGGGTCACTGGGAGCTGGTGAGTGGGCGGCCACTCCTGCCTCTACCCCTTGGTCCCCAGACCCATGCGGTCCTCTGTGGGGCCACTGATTTAGGCCGAAGACCACATCCTGGAGGACGGGACCACCACAGTCCCAGAGGGTTGGTGTCAGGACGGTGGCACCAACTCTTTCCGAGAAGCTCGCCGACGCTCTAGGCTCTGCGGCGCCCGTGTTCCCGCTCCCTCTGCACCCCCTTGCCGGAAGCAGTCAGAGGCCCTGGGGGCCGTTACACAGGAGCCCGTCTCCCAGGGCAGACACGGAGGCATCGTGGGCTGGAAAGGTGGGTCCGTGCCTGGAATACCAGCTGTTGTCTTTCCAAGGGGAAGGGGCCTCCAGGGCTGAGTTTACCTCCAAGTGACCGGTTGGTCTCTGGCTGGTTTTGCGTCCAGGGCCCCTGGTGGTCTTTATGGCCAGAGGACGGGATGTGCTGCTGCCGACAGGAGCTGGGCTTGTCATGGGGCGCCTGGCTTCGTCTGGTCCTCCATCGCTCCAGGCGGGAGCCGTGTGGGAGCACCGGACGCTGCGTCACTGGCCGCGTCATTGGCTTGTGGGTGGCTGGGTGCCCGCTCGGCAGCCGGTGCCCCTGGTGAACACCCACAAGTCCCCACGCTGGTGTCCACCCGGCAGTTCCATTCCCCAGTCCGTCTTCCAGCGTTGCCCTTCTCAGGCCCTGACCGGCCAGCCGAGCCCTTCCCCAGCCCTGTCCACGTGTGTCCTTACCATGAGCTGCTCTCCTCTCCTCATGAGAGGCCAGGGATCGCCCCGACCCCTGA                  
