# Supplementary material for: ΔNp63α promotes radioresistance in esophageal squamous cell carcinoma through the PLEC-KEAP1-NRF2 feedback loop
Source: Cell Death Dis. 2024 Nov 5;15(11):793. doi: 10.1038/s41419-024-07194-4 (PMC11538512; doi:10.1038/s41419-024-07194-4)
Supplement: Supplementary file 4 — Supplementary Table 3 [file 41419_2024_7194_MOESM4_ESM.rtf]

Supplementary Table 3 The complete hARE sequence alignment in the TP63 promoter.
CATTTTTACCCAGGGGGCAGCGTGACCCCTAATTCAGAGACCGTGGAGCTTTACGAAGTCTGAGGAAATGACTGAGAAAAGGCAAGAGCTCTAAATAGAGTTGTTCTAACTACTGAGGACTTGGGACATTGACGTAAAATCCTTGCATTTCTTTATTTATCTGAAAAATGTAGATAATAATACTAACTTACACCACTAGGTGGTTAGAAAATTAATGCATGTTACTAAACATTTAGTACATTTGAGATCCACATATAAGCATTGTTGTTGAGTGCAGAGAATTGTTACTTCTTTGTAACCTGCTTAAATCAATGCATGATATTTACATCAGCTACTCACTAATTCTGAAACCAAACATTATCAGGTATACTTTAATTAGCTCTTTTATGGAAATGTTTGTGCTGAGTTCAAATCTACATGAACTATTAGCGAATTTTTCATTGTGAGGATACCTTAAAGCAAAAAATATGGATTTTCCACCAATATGTTCCTTTTCCTTTATGTCATTTAAGTATCTTAAGTAGCTGTGCAGCTTGACTACTTCACTTGTACAATATGAAGATGAGCAAACTGAGGGACAAATAAATATCCGAGTAAAATCAGAGGAATATCCTCTAAATCACTTCTCTGATTCTGGACAAAAACAGGCTCTTATCTTTGTAATTGTCTTTAGAAATCATTTAGCTCTGCCCTCATTTCACAAATGAGGAATCTGAATCCAGAGTGGTTAAGTAACTTGCCCCAAGGGCACGGAGCTCCTGTCTCCTAACTCCCAGTCCAGTGCTCTTTTCTTCCAACATACTCAAAGTTCTCAAGTAGCCATAGTGGATATATTGTCTTAGATTTTGTCCAGAATCTTTGTAGATATGTTAGACAGAGACCGTATTTGGATTCATGCTGATAGCTGAGGTGGCACATGGAAGGAATCTGACTTTTTCATCTTTCAAATCTTGCTGGGAATCTTTTGACTTCGTGAAAGGTGAAGTTTGTTTACATTTTTAGTGGATATCAATACTTGGGACCCTGAGCCTTAGATTTAGGTGTTCAAATGGCTACATGATCCCATTAGTGTAAAAAACAATTTAATAATGACTTTGGTAGGCA
P1
GTTGTGCTAACAGCATTTCCTCTAGATCATTGATTCCCAAGCGTGACTCATCCAGAACCGCCTAGCAGGCTGTATAAAATCCAGTTGTTGGGGCTGTAACCCTGCAGAGTCTGATTCAGGAGGTCTGGGGCCACGTCCAGAAATCTGTTTTTAAAAAGCACCCTGGATGATTTTCAGGATCATCCAAGTTTTTGGGCCACAGGAATTATGCATACCTTTAAGGAAGTAACCATCTGCTTTCTACTGTTGACTCTTAAGCTGTCAGTAGGTGTAGAATTTAGACACATTGTACAAAAAATTATACTGTATTTTGTAAGTAGGTTTTTTTTTTTTTGCCTTCTAGGCAGTGCTCATTTGGTTAATTATAGGTAGAAAGAGAGATCATTCTTCCCAATATATGTGTGAAGAAATGAATGTTTTGTCTGATATAAATTTGTAGTGTTACAGAGTACTGTCATATTCTTGCATACTCAAGATCAAATTATATTATGGGAGTTTTTTGGTTTTTGTGTATAAAAGAAGGAAGCATTATTACCAGTAACCTATGGGTCTTATATATCAGCCAATGACCACGTCATCCTTTAAGTGCATAAATTTTATGGATGCAAAAGTAGAAGTAAAGGGGAGGCAAATCCGAAGTCGTGAACGTATTTGCAAACTTTGTTTCTGAAATGTATGTTCTAATCTCTTCTGGCTTCCAATTCTCTTCTAAACTTTGTGTCTTCTAAATTTTAGAAGAAATCACAATTTTCACTTATAGTTATCTTGGCCACTTACAACCTTTCAAGCAAAATAGAGGAAATTGGGTCCCTGAGTGGGGGGTGGGGG
P2
TGTAGGATGATGTCAAGAAATAAATCTAACTGAATTACAAATACTCAGCAGTAAAGAGAGTATTTTAATGAATCAGATGATAACCATCAGGGTGCATTTCATCTCCACTAAATCTTCGTACCAAGGCCAGATTCTACATGAATGTTGGTACGTATTTATGTAAATGTATTTTTAAAACAAAAGCCAATTGATATCTTATGCTTTAATACTTATTCCACTAATCATTTACATAGATGCATCACGTGCAGTAATCATTTTTATTACCTATTCACAAGCAAAAATATTAGTATAAACTGGGTACAATAAAATAGAGAAAAGAAATATTCAAATAGATAAGCGTTTTGTTAAAAAAAAAAAAAAGAAGAAAGAAAGGACACATTTATCAGGATTCCTATTTCCCGTACATAATATGGATGTTTGTTTGTTTTTGTAAGTTAACGGGACCGGTGGTTTAACTTGTTATTGAAACATGCTCGAAAAAATCAGGTAGCTTATTTTGTAATTGCTTGTTATGAAACCACTGGCATTTCTCTGGGGAAAATAAGTTAAAAACTCTTTAGCTATCAGGCAGTGGGTTTTAATTTTTTATATTGGTTAAATGTAACAGTGGATTTGCGTACTCTCTCCTAATTTCTAACTTTGTGTAATCATTCTTGAAACCCCAAATCTAGATTTTAAAAAAGAAGCCTTCTAAAAGTTTTCCTGAAGTTTACTTTTCAGTTACAAAGAGTAAAATAACTTTCTGAAATGCCTTCTGTAAATCGTGGTGGTGGTGCGGTTTGTTTGGGGAGATTTGTTTTGTTTTTAAAAGACAGTGCACTTTCTTATGAAAGAGACAGGGAAAGTTTTACCTGTCTGTCTCCTGGGTTTGTTTTTTTTTTCTTTCTTTCTTTTTCTTTTAAAGATTGGTGATAAGGAATTCTAACTACTTAATGAGATGGGAGAGGCCTCACTCCATTGGAGTGGAGGAGTCCAGGTGGAAGTTGATGGATTGGACAGGTAAAGAGAAGAGTCCCGCCTCCTCATGCCTATAGTTGGGTATATATTAGGAAACCTTAAATTATGTACAGAGAGAGAAAGAGAGAGAGGGACTTGAGTTCTGTTATCTTCTTAAGTAGATTCATATTGTAAGGGTCTCGGGGTGGGGGGGTTGGCAAAATCCTGGAGCCAGAA
                                        -1|+1
GAAAGGACAGCAGCATTGATCAATCTTACAGCTAACATGTTGTACCTGGAAAACAATGCCCAGACTCAATTTAGTGAGGTAAGGATTTTAGATTTTAGCACTCCATTTAGAGATGCTTTTTAATTTTTATTTTTGTAAAAAAACTTACGTATTTGCGGTTCTCGGTCACCCAATGTAATGTTTTGCAAATTGTATATAGGAATCTCCTTTTCTTGGTTAATGTTTTCTGTGGTGGCTGTAAGATTTTTTTTTTTTTATTAAGTAGGAGATGAAACAGTAGGAGAAGATGAAAAAGAAAATCTGGGTGACATTATGTATTTGAAAAAATAATATTCAGGAGTTTATAAAATCACTTTTTAGAAAAACAGACTCAGAGAGCAGATCACACTCTGCTAGATACATTGCAATGAATCCTCTGATGGGTATTCATGTACCAGTAAGAAAAATCAGTTGACTGTGTCTTAACTTCTTAGCATAGTTCTATTTCCAGACTTCATCCTAAAAGCAAATGAAGCTTTTTTCAAAATAATCAGGTGAGATTTTTTAAAGCCATCTTCGTTTTTAAGTCTGTCAATCCAGAGACCCACCTAAATCCAGAAGGGGCTGTGATAACGTTTCTGTCGTCTGGAGGGTGACTTGTACTTTCACGTGTGATGACAAATAAAGTTATTTTGGAGGGATGCATTGTTAAATATTTATACATGCATACATGTAAATGTGTATTATAGCATACAGGATTTTATTTAAGAAACTGATTATTTGCATTTCTGATCTCTTCTCAGCAAGTTTGTCTTAGGTATTTAAACCTGTGCAAAGGGATAAAAAAAAAAATCCTGAAGAGACTGTAAGGCTTTGAGGAAGTTTCCTAAGGTTGAGGGAGGTCTAACAACAATATTAGTTTACATTCCTCAGAATGGGGCAGCTGTGTTGACACTAATCAGATTGGGTTGTGGTGGGGAGTGTTGTGGAGAGAGAGAGTTCGGTGTTTCAGATAAAAATGCTTGCAGGTGAGTGCTTGGAAAAGTGGACTAGAGTACTCAAATTGAGCTCCTTAAAAATCAAAACAAAATGCATAGTATTCCGGTGCAGACCGCAAGAGGAAGAGTAGAGCAGAGGGCTGCGAAGCAGGGCCTGTGCTATCGTATTGACCACGCTATTGTCTAGACTCCTGAACTGTGTAAATACAACAGGGGAAATATCGAGTTATCTAAAGAAGGAAAAGGAAGTGCCTATTTTTCTTTAAAACATTCAAGCAGGCTTGCTTGTCCTCAGTAGAGAAACTTGAGTTTCTTGTTTTGCAGTTATATTGAAAGTTAGCTTAAAATGTTACCAATTTGTTTTTAAATAGGATTAGCTTTTAAGATCTTCTTGATTCTATTGGATCCAATTTTGTCCAGTTTTAATCATTCAGAAAATGATCTGTCCATCATGTCATACCTGAGTTTGTTTGTCTTTATTCATGAACTATTTCACACACAGTATTTATAAGCCGCCTAGGATCTTGTAAGTCAAATGGGAGAAGCAAAAGTCTGTTCATTGATATCTCTAAGTGAAGCACAAGGTTGATGTAAAGTGGCAAGAAATGATTAATTTTAGGTTAAATATAATGTCGTGAAAGAAAGCAATTGCATTAATGTTGTGATTAGAGGATATTTGTAGAAATTATTTCCTTTTATGGAGGCAAAAGAGTCATATTGTATGTGAGAATATGGCATGCTTATGAAAGAAAATAGTTTTATACACAGGTTTTAGTTTTTCACAACTCTAGGTAAATATGTAATCAAGAGCTACAAAAGGCATCCCTTGTCTGTTTTCTATTGGAATGCTGCTAAGGTCGACAAAAAATATCTGTAAAAAACATGGCTGCACTTCCCTACCTTTCTATGTCTTTTTGCATGTTGCCTGCATGACTTTCATATCCTTCCACCAGTGCATGTATTAGGTTATAATTTCATGGCTTGACTTCCATTCTATTACGTACGAACAACTTTCTTTCTGTACAGGATTATGCTTTCATATCTAGTCCTCTGTTAAAATTCAGTGCTACATCTGAGTATATGAAATGCCTGGCACCACATGGCTCTGAGAACCATGAATTTAATATAAAAGTTATAATGTATAGGGATTTATGTTTATTAGTAACTGTCAATACAGAAAAAGCAATCTACTATTATTACACAAGAATTGTTAAACATTGGGTAGATTAAAAATGAAAAAGGCATAAAACTCCCTGTAAGAGTGAAAATTATTTAAAAACTATAATCCATTCTTTGACTTCAAATAAATTTATAGTTTAATTTGCTAGCAATTTCTTTGAGTTGATAACTCAAAAACTATATAAGAACACTGTTCCTTCTCTATTCCTTTTCCCACAACTTTTGAATTCTTCCCCAACCCTTAGCAGATTTGGATTTGGGGGAGCTGCTAATTAACAGGAAATGGTCCGGAAGGGAGGAGAACACAAGCAGGATATAACAGGCAAGGCTGAAGGCTCTACCCTTGGGTACAGCGGAGAGGCCGCAAGCAAGCTCCACTCCCCAGTGCCAGACTAGGAAAGACAGAGCCTCTGGGAATGGTCTCAGACTTGGAGATAGAGAGGGTCAAAATAAAGGAACTTCTTCACAGATCAAGTTTTAACTTCAACTTTCTGGCAAAACAAAACAAAACAAAACTGGGCTGTGTGTGTGGTGTAAGTGTGTATGTGTGTGAGTGGGGAGGATCTCAAACTCCTTCTTAGAAAGAGATATTTACTAAAATTCCTCATCCGGGGTTTTAGATGATGAAGGAAAGACACTTTGGTAAATGGGTTACAGTATTTTCGAACTAAATGGGAAATCAAGGTTCATATATGGAAACACCTCATTTTACAAATCAGGGTTAATGATTTTTCTTTCTGTAGTTGAGAGAGCTATATGTATACGATCGTGTTCCCTTTACTCAGCCACTTTCTTTTTAATACCAAGGAGAGATGGGATGATTCATAGTAGATGCTCCAAAACAGTTTCCATGAAGAACAGTTGTACAAAGCCAGTTGAATGCAAGAAAAAAAGTGTGGAGACAGCAGTGTAAACTCCCTGTAGTCCAGGTCATTCTCACTTAATTGACTTCTTTGTCTGCTTGGTCTAGGGAAAAAAATCTGTCCTGTCCTAGTACTATTTCTACTGACTCAACCATTTAAAAGATATGGGTGTGAGTTTTCTATTGATAGTTAATAAATGTATTAAGCTGTCCCATGTTGTACTTCTGAGCTAGTTAGCATCATAGGAAAAAGCACAGTGGGTGAGAAGCAGAAGCTGCATTTTTAATTAGTGTCAGCCAGGTCGTATTCCTCACTCCATGCTGTTGCCATAGATTGCACTACTTGTTTTACAGTTGGTTGTCCTCGAGCCCAGAGGGAACTAGGAGAAAGACATCTTAGATCACAGATCACAGATCATACCTTCCTTCACTACTACTGGAAAAAAAAAGATTTTGAGACAATAAATCACTTATCCTTTACCTCGATAAGGAAAACCGTGTTTGGTTTGGTTTGCTTTTTAGAAGAGCAGTGATTCTCAACAGCATGTCACACGTACAGCAGAAAGCATCAATGGATGTCTACATTTTCTAGAGCAACCAAAAAAATATTGGTTTCTTTTTCATTATACGTACATTTTCTGAATGCAAAGTTGCTAAAACATTCCTCAATGACTATGGACAGAATTTAGATTCTGCGTTCTGCTATAAAGGGAGTATATATCTAATGTCATGAGATTTTAAAAAGGGATTTGGATCAAACAGTTGAGAAATATGTTACCACAAATACACCCTGGTTTTCTTTGATGTGCATATGATTGCTCTGGGTGGAATCTGAGCTGGTAGAATAGAGGGGCACAGGTAAAAATCCTTCCTTCCTAGTATAACTTTTCTCTCCCCTTGAATATTTTTAAATTTCTAATTGTTTTATGTCAACAAATAGAATGAATACAGGTAACAAAAAGATACTTCTGTGAAAAACAACTTGATGATTTCCATAATTTATAAGTTTTACTATTTTGAAGATAAAAAATTGTTAAACCCAAGTGGGCGCTCAATTTTCTTTAGATGTATGTTGAGGAAAATGTCAGTTGACTAATATAGCATTATTGTCCAGTGCCAATTTAGTGGAACCTACAAATGGTATCCAATAGAGTACTACATTTTGGGTCTGGAAGAGCATGGATTCAAGTGTCAAGGTGAAACATTTGATTCAAAAGTTAAGTTTTAAATCTTATTCCTAATCTTAAGGACACAATTTGTGGGCCCAAGAAAATAGTGAAATGTGGCTTATTAATGGGCCACCACTCTAAACATTTTATAATTTTTACTTATACTTTTTGATTAAGTTCTATGTTGACAATATTCATGAAAAGTAGTATTGAGAAAGAGAAACAGGTTAGAAGAAGAACTGACTGACTCTTAAAAAAATGTTTACTGAGCATCAATTATATGTAAAATGGTATATAGGATCTAAAAATAATTAAGAAATAATTCCTACACTAAGAAGTTTATAGTTCTTTGGGGGAATGAGCTAAGTATGCATGCAATTATAATACAGGAGAGATTTTAAGTGTGTGAGCAAGATAATATAAAGCATCAAAGGGAAAGAGAGTCCATGATGTAGGAAGATCAAGAAAGTCTATATGCATATGTAAGATGAAGCTGCACTTGGAAGGACAGATGGAAATTTCCAAGTTCTAGAAATATAAGAAGGAATGAGGATTTTAGGTGAAGAAATAAGCATCAGCAAAGGGAAGGGGGGTGAGGAGTGAATGCTGGTACACTCAGAAGCATCAAATAGTTCAAATGCAATTGAAATTATGAGACGATCAGGAAATAAAGTTGGAAATGCAAGATAGAGTGGCTCAGAGATGACCATAACACTGAGAGGAGAGTACATTTATCATAGTGACAATGAAAAGCCATGGAAGGATTTCGAACAGAAGAATGGTGTAATTGTGTAATTGGAGCTCTACTAGAGAGCACAGGGAGAATAAATGGCTTTGCTTGAGATTGCCTAGAAGTATGAAAATGCATCTCTCTGCATTGCACATTGGCTGCCTGTTTTGCAGAAATATCTATCAGTTCTCTTGGATCTTTGGAAGGATACGCATGGATTGTTTCTAAAGTCTCATACAACCTGTGTATTTTCTGCCCAGTGCTCTTTTTAATAGCTATGGTCCCTGCCATAGACAAATTGGGAACTACTGGTCTTGGTTTGTCTTTATTTATGCCTGCTATTCTAGCATAATTACTAATAGCACACCTTTTCACTTTCAAAACTGTCCCATTTAGAAGATAAATTATATAGTCACTCTGTAAAGACCCCATTCTTTATTTCATTCTCTTCCACACACTAACACTCCACCCCAAAATGAAAAAGTAGTATAAATTTGGCTGTAAGCCAGACACAATGATTTTGAAGTACCAGCCACAAATCATTTTGATTATTTCCCCCACTAAAGGAAACACTTCCTTCCAACCCAACAGTTACTGAAAACTTATAATAGTCACTGTTATATGCACTGTGAGGAAAGAAGAAATTAGTAATTCACAGCTTCTAGCCTCCGTGAATTCATAATCTGAGGGAGTTATGAGGGGTTCGTAAATGACTCCAGTATAAAACAGAATTGGTAAAAGATATCCCAAGTTTTGAAAGAGTGAAATCGAGTTCAAACTGTTTATGATAAAGGTAATATTTTATATGGTTTGAGAACTGAAATTCAGATGTGGCGTGAGGGGAGGAAAGACATTCTATGTAGAGGGGATAAAATTTATAAAAATACAAGGGAGGACTGTGCCAGACAACGTGAAGGAACATAGTATATTGATTTAAAAAGAAAATGTGTTGGTTTAAACACTGATATGTATTTACTATTTATGTCAAAATGGAACGCTTTTGTAGAAATAGCAACAAAAGTTAATTGGAATATTGATCTCACTAAGAGCGGAAACCTATATGTGTTTTAAAAGTGTGACCAGTGTTCAGAATTCGAGATTCAAAACTTCACATACTCTAGGGATGATGAGTGACATCTTGCAGGAAGGAAAAGAAAATTAGCCCGAATGATTCCTTAGGCTGAAAAGTTGAGGATTACCTTTTCCCGTTCCTTAGACTTCTACAGAGCCAGATATGGTGTCCTATCATTTATAGGGACTTAGTAATGACATTTTAAAACTTATCAAAGCTAGGAACAAAATTCAAACTGACTCTCTGTGTTTATTATGAGTTAATTTTATTTATTAAATAGTCGTCTTAGAAATAGGACCAGAAGTCTTTTCTCTGGTCACTGCTCTAAATGCAAATAGTAATCCTGAAGCTGAATATAAGACATTTATTCAGAATGAGAATCCCAGCTGGGAAATAAATAACAGAGCAGAGGGAAATATGAATATCCCATTTAAAAGCCAACCCCACCAAATAAAAAATTTAAGATATTATCTTTGCCATAAAATTTTTAAATTTGACTTTGGGACAAGGATACATGTGTAAGATGTAATATGTAAAGATAAATATTGGGAGAAGACATGTCTTTTCCCACATCAATATCCTCCCTTTCCACAGCCTCCTTCATCACTTCCATCTCACCCAAGAAGACCACGTGGGCAATTGAAGAGATAATAACAGGGCAGGGGAGGGTAGGCCATGGTGGGAGGAAGGAGTGAACCAGTGACTTGATTTAAAGCCAAAGGGAGTGGATAAGAGAACTGGGCTTCAAGATAAATAAGGAGACATCTAAGCTAGTACTCATTTCTTTCTCTTTTTTTTACCAACCTAATCCTCCACTTTCAAAAAGTAGATTTTGAAAGCATCAAATGTTAACATATGATTGTCCTACAGACATTAGGGGAAACAGTGAGGTTCCACTAAATACAACCTCCTTAAAAGAAATAAGTAGTTCAATGAAGTGTCTGTAAAATCTTTTCAAAAGTCTTACTTTATACTTCCCCACAGTTGATTTCCTTAGTTGGTCTGAGAATATAGCACCTATACTATTCAAAAACTATTCTTTTCCATTTTCTGCAAAAAAAATTATAAATTTCTTATTTCTAAGTTGATTGATTGCATGTTTGATATGGACTAACTTTATTCTGATTTTGAAGTTGTTCAAGCTTCTTTTTCAAAGACAAAGTACAAATGGATCATTGTATGGATTCGAGCTGAGATGGCTTCACCAGATAATGTTCACCTGTCAATTTAAAATTCAGTCACTGGGGTTAAGATAGAGCACATGTTGGTTCTAGGTGAGGGAATAACTGGCAAGAATAAAACATTTTTGGAATGTTAGTGAATGTGTGTGTAACGTAAATTGATATTTCATGCAAAACTTGTAATCTGAATTGCATCTTGTCTGTATTTCTTAAATGGCAAAAAGATTTCCTTATAAAAGCTGAAAGAGGGAATTTTGAGTCAAGCTGTGGTGTGTTGCCAGAGGCGGAAATTTATGAAACACTCTTGTCAGCTATGCTGGAAACACTACAAAAGAAATAGTTCTACTTTCTTATTGGTGCTAAAGCCACTAATAGGTGCAGTTTAGTATAATACCCCTATGAGAATTACCACCCCCGAAGTGAGCTTGCCTTGGAAGGAACTCTGTACAGGCATTTTCTTTTTCCTTTTAAGCTTGCGCAAAACACCCAAAGGGGCCATTTCCCACCCAGCCAGTGATCTTTCTCAAGTTTATTCTTTCTCAGATCACAGAGACAATGGGGAATATGCCCTGTTTACTCAAATCAGTCTGGATTAGTGTCACTCTATTGATGGAGTCAGAATTAAAAGGAAAACCATTACCCTCTCAAAGGGCAACATCAGATGTTTGGGGAATGGCGTGATTACTTTTAAATAGACCGTGTGAGACTCTGTCACCAGACTGAGGTTGAGGACTCCACAAGACACACTTGAAGAGAAACAGTAACACTGGGTGAGGCAGGAAGCTCTTCATAAGCCTTAGGCCTGGGAAATTATTTCAACAGACAATCCCAAAATGCTTGAAAGGCTGAAAATCAGAATGGTGAGACTTCAGAGAAGAGGGCTTTTCTGCCTTCTTTCTTTGCTCCAACACCTCACCCCCATATATTTATCTCCAGGTCAAATCCTTTCCAGTCATTAAGCCCTGCTGGGGAGAAACTTAACCCTGAAAAAGCTCTCAGATTCCCCCATTCAGGATAACACTTCCATCCTCTGAACTCTTTTACTCTGTTTCTTCCTCATTGCTCTCTGTGTACACCAGATCCTTCCTCCCTATCCTTTGACTGAAGACCCTGGGAGGGTAGAGGATGTGTTATGCTTATCTCTGTATCCCCTACAGGGCCACTCAGTAATGTTCCCTGAATGTGATGAGGACATGCATCTGTTGCTGTTGCTTGAAGGGATATCTTCACCTAGGAATATATGGTGTATTGTATATACTTAAAAGGCTGTTTGTAATTGACTTGTGCACAAAAATAAATAAAGCGAGCCGACATATTATACAAGCCTCTTCTATCTATATGTTCTGCAGGTAAACCAAATTGTGTCATTTTCCTTCACATCTTGCAGGTCATTTGTTTTTGCTGTTTGTTTGTTTTAATAAGCCCAATATTATTTCCATTATCTTTTAAATAGCAAGAAAGTTTTTGAAAGACAGGGCAGATGATTTGTTTGGTATCAAGTAATTAGTGTAAAGTATGTGCTGCTTTCACATAATTGATTACTCACAAGGACAGAGAATGTGTTTGATTAAAAAAAAGTCTTAGATTTTTATCTTGCTTTGCAGGCCCTTTTTAAAAGTGTTTACCTGTGTCTGCACTGTTCCCTTGATTCAGTTTATCCCCTGCAGTTCTCTCTGTCCTCACCAGAGATGGGGGAAGTTTGATCTGGGTGAATAAGCTTGATGATTTGCAAACGCTCTCTCCTTTTAATTCTCTATAGTGTTATCACTAAAGGATGTCTGAGAAACCTCAAAGCAATTTTTCTCTTAAACGTGGTATTCCAACTTTTGGTTTTGCTCATATCTTTTATAATAGGACAAATAGAATAAAATGAATAAGACGAGTGTCTTTTCAAAAGTAAGGCATGTCTTCTGCATAGCTCAAAACTTGAGTTTTATTTTGCAGTTAAAGTGACAGCTGAGAGGAACACTCCAATGTTATATAGGTCACCATATCTAATTGCAAAGTAATCACTAAAAACTTTAATTATCTCAAATATAACTTAAAAAGTAGCAATCAGCAAAGAATTTGACAAACAAAAGCAAATTAGAACTCATGAAACAGTGTTCCCCGCTTGAATGCCTATAGTCTAACAAGATTGAGTAGTACACTCCTTGTTTCAAGCCTCAGTTTTCCAGTTGCAAATAAAAACTAGTGTTTCAACCTTTAAGTGTTTGTTATATTGGAGCATAAATTTATATCATTTGCAAGAAAGGATTTATAAGTCTGATAAACTATAATCTCAGTTCCTTAAACATACAATCACAAACTGTTAATTATGGCTTTATTCTAAACTGAATTCTGGCTAACTGGGCATAAAATCATGGAAATTAGGCAGGGAGGGACCAATTTTGTGGCCTATCTACCAGAAACTAAATGTCTTTCTCTAAGCTTGTTTCTGGGTGGTTTCTTTTCAAAGCAACCTGCATTTGGCCATGTGCTCCATCAGTAGGAAGTGAATAATTATATGACCACAAAGATATAGGTTCTTATTGCTGACCTAGGTGCATTCGAAACAAAACCTAGACCATCAGGTTAAAAAACACTGCCTTGGCACAAAAGTGTGTATGTGTTTGTTTTAAGGAAGTAGAAGCTCATTAATAAACTTCCCAGCTAGGTGTGAGCTACCAAGTATTACTCATTGTATTGTGAGTGACAGGACTGGCTATTCTGCTACTAGTTCCCATAAAAGCTGAAGCTAAAAGCACAATTCCCACCTACCAAGACAAAGCTAAACAAAAAGTAAAGATCATAGAGATTATTTAGGGATGCACTATAACATTTGTCTGACAGTAGCATATAAAGAAATGAATCAAATTTGGACACAGACTCAGGATAAAATAAATGATCAGTTGTATTAGAATAAAAGAGATACTTTCTGCAAGTAATTTTCATGTTGAAGTGTTTTGCATTTATTCTTTTAAGAACTGTCTCCTGAGCATCTATTATGTTTCAGCCATTATCCCAGGTATTGGGGATTCAGTACTAAACAGGCCAGATATATTCTCTCATTAACTTATTGCCTAGCAGAGAAGACCAACATTTTTAAAAGTTTATACATATAGTTAATTTCTATTATGATTATATGATACAAATGGAAAGTGCTATGAAAATGTGGAACAAAAGAGAATAATCTGTCTGAACAGTCAAAGAAGACTTCTGGGAGATGACATCTGAGCTAAAGGTTGAACAAGGAATTGGAAAACAGCTGGCATGTGCAAAAGACTTGAAGACTGAAGGAGTTAGCCTTTAAAAAAATGAAGAAAGTTCTATTTGGCCAGAGCAGAGTTTCAAATAGTGCCTCACAGGCCACGTTAAAGACCTGAGGCCTTTATTCTAGGAGAATAGGGAGCTGCTCAAGGAATTTAAGCTTGAGAGTGACAAGATCAGATTTGCAATGCCTTTCAAGTGGTAGTTACAAGGAGTTGGGTCTCTGACCCTTTGCAATTATACCCATTCTAACTAAGAATGGGGAAACTTTTATATCCTGTCTTTAATGAGTAAAAAAAAAAAAAAGAAAAAAAAAGCAAATAAAAGAGGTGCGCTTTTCTTGATTGTAACTTTCAGATAAAAATTCCAGGCAGAAATGTGAGTGATTCCTATTTACATGCAGTCGGAATGAAAGCTACTTAGGAAAGCGGGTTCCGGAGCCAGATTTACTGGTTCGACATCCAGGCTCTTCTCACTACCTTGGGCGAGTTACTTAACCTTGTGGAGCTTCAGTTTCCTCATCTATAAAACATGGATAAAACATGGATATTAATGCCATGTGTCTCATGAGAAATAAGTAAAACCATGCAAAGCACTTGAACACTCTAAGACACATACTTAGAACTCCGTAAGAGAATTAATAATAGTAGAAGTAATTGCTCATTTTCTTTTATAGAGGGGATGGAAGATGGATGATAAGTAGATTTCAGAGTTTTAAAATTTATTTTTATTTTTATTTTTTGCTCCTGATAGTCACCATTACTTTATTGAAACTGATATGTCTTCAGTTTCATGTCATGGTAACGAAAGCTTATTTTCTGTGATAAAATGTTAATCACATTTAATCACCCGTTGTTTTAAGTCACAATTTAGGATATGAACTAGACAAATATACTACCAACATTATTTAAAAAATATTTTTAAACTCACTTTCCAAACCAAGGATGCATTACTGCTGGGCTTCTAAAGGCAGTATGGTATGCTTAACATAAAGATGGCTTAACATTCATTGAAGAATTTTATTACTATGTACTTTCTTCTTTTAATATTACCATATCTGCTATATAGAGCTATGAAAACATATTGATTGCTATAGATTGAAAATCTCTTCAAACATATTTTGATTCACTTGGAATAAACTGAAATTCTGAATATTTCCCCTAATACCAGAATTAAAAATTATTAAATATTAAAATTCTATCCTGCTTTCTGCTTAGTTTGATACTTCTTTGATATAAAATGATAATAATTCCTTCAGAATTGGGATGCCTTCTGATTTTCATTTTGTTGAGTTGTTATGATTATTATAGGAGTATGTACTAAAATAATATAATTTTAAAATTTATTTTTCTTAATTGAGTTCTACACATTTTAATTTAGCTGCATTTGGATCAAGTTTTAATTTTAATACCTTTCCCACTTCCTGTCTTTTCAAATGTTGTGTCAAATGTTTGTTGACATTGTTGTCCACCTGATCAGACAACTTTGTATTTATTTCTCTCACCTGCCGTTGGGGCTTCTAGAGCTCTTTCTCCTAAATTATGGAATCTTGGCACTCAAAGGAATCTTGACACTTTGTGTCAGAACACAGGACATCTAGTGAACTCTCTGCTTACAGTCTTGATAGTTTTGGGTGCGTTTAAATACTTTATTCTTATATTTTGTTCTTTGTATGAGTATATATTAAGTATTCACTTTTTATATAATTTTAGAAAACAAAATGAGTGACATATTTTATACCTAATAAAATCTGGTTTTGAGAAAAATGTGATGCTACATAAATTCTTCGGTTGGAAAGATGGTGTTTTAACTTGACGTTTGAATACCAATATATTTTCTTAACCTGGAGTAGAACACTGTAAGTCAATTGTGTTTAATTCTAGCATAAAAGAAACCATCATTCTCTGAGGTGGGGCAGGTGGCAGAAGACGAAAAAATGACAAGTATGTCTCCCTTAGGCAGTGCTTGTTGAAAAGTCACTATCCTGCTGATATTTGGGGATGGAAATGTAGTACTCTCTGCTAAAGCATAATTGAATGAATGCTTTTAAAGCCAGGGTCATCAAGTAAAGCTGTTTTCTTTACCCATGAGGGACTGTAGAATGTTCATAAAAGCACCTCCGTGAAGCCTCAGGTGAAAGGTGGTTATCAGCTGAGGAAACTGTATGTGCTGCCTAAAATGTTTTAGATTTCTAAGTTTGGTCAGGGACATCTGTATAGAGAGAGAACAGTTACCTTAGAAAACTGTTTTGAATGTGGTCTTATAGCATTGTTTTAACGTAGCTTCACAGATGAGGATGGCCATAAGTCACAGAATGTTAGAACTGGAGATCCCTCTCATTGAATCCTCAAATTCAGTCTCCTCAATTTTACACAGACTTTGGATTATGGTAAACTAAGGTCCACAAGTTGTTCAGCTACCTTGCCCAGAGGTACACAGCACACTAGTGGCAGTGTTGGGACTTAGACTCCATGTAAAGAAGTTTAAGTTCAGAATTCTTTTGCATCACTAGGATCCATACAGGAGTGTAAGGAGTACTGAAGCGTGTTCAATCCCCTAGCCTCATCCACCAGTCACTCATGGATTATAAATACAATTTCTTCCAAGTGAAATTTGGGAATACTGTTGTATCTTGTAACAAAGGTCTGGTTGAATCGAGATAAGTTATGATTATATATCCCAACAGTAATCTCTTCTACCCCTGATTTGGTCCCTATCCCATTTTTTTAATAACCTCTTACTATATATTGTTGACTATTGTTGCTATAGTGTCATAAATTTACTATTATAGATAGTAATATCTGAAAAAGGGGACATTTTAAAAAATTCTCTTGGCCAGGTGTGGTGTCTCACACCTTTAATCCCAACACTTTGGGAGGCTGAGGCAGGTGGATCACCTGAGGTCGGGAGTTTGAGACCAGCCCGACCAACATGGAGAAACCACATCTCTACTAAAAATACAAAATCAGCCGGGCATGGTGGCACCTGCCTGTAATCCTAGCTACTCAGGAGGCTGAGGGAGGAGAATCTCTTGAACCTGGGAGGCAGAGGTTGTGGTGACCCAAGATCGTGCCATTGCACTCCAGCCTGGGCAACAAGAGTGAAACTCCATCTCAAAGAAAAAAAATTATCTTTTACAGATAAATTGGTGGAGAGAGATTTTCAAAAACACAAATTATAGGTAAATACCTATTTCTATTGCATTGGTCAAATAGCTTCATTGGAATCTCTTTTTGATGTTAAATACTTTTATAGGAAAATAATTACGTCATCAACTTTAAGTAAATATAAATCCAATTATTTAATATTGAGTTATAGCAATATATATTGACACAGTTTTGTTTCTTCATGGTGAAAACAGTTTGAGATTCAGCTCCTGGGTATCTTTATACCTACCATGCAAGCTGTATGTTTCAGAATGCCCTCAGGGTGTTCTATGATTGCAGGGAATATGTCGAGTCCATGTGACCAAGGCGTACTTCATCCTTTCTGTGATCAGATCAGCTAGTTTGTGAGGAACCTGAATATATCACAACTACAAATAAATTTTCCAGGGTCCAAACAGTGATTCTTCTATTTCAAAGTTATTTTATAGAAAATGAAGCATCTGTTTGGAATCACAGGAAATTATTTTACTTTATAAGTTTCACAGTAGATATCACAGAAGTTGTGTTCATTTTTGCCTTTATGTTTTGTGGAAAGAATACTGCCTCTTTTTTCAGACATGCTGAGATAGATATATAATGTTTCCATTCACTCATTATTTGGGTGTCTGCAGGGTATGTTAAGGTTATTCCAAAATCAAGATGCCTTGTGTTTAGAAAGGGAGAACCTGCTTCCTATTTCATTTCAGATACGCCAATAACTTTTGTTACCTAAGAAATACATGCATATGGGACATGGGGGCTCTATGGAGTTCTTCCAAAGGCCGTTTTTATGGCTGATCTGAAAGTATAGTGTTCTAATCTCTGAGACTGACTCTTTGACTTGTCATTGTCAGTAAGCATTGCATTAACATCCCAGCATATTACACATTTCTCTCCTCTGTTATGGAATTATTGGCTGTAAATAAGGCAAGAGGTACTGCTCCCTTTACCCCGCCAGATTTGCTGTGATAGAAAATAAGGGATAGATATTATTCACTTGACCTGGGACAGCTTCATGGGCATAAAAACTGCAATTGGTCACACAGGGCCCTATCTTAGAAAAGCACCATTCTTTGTTTAATGCTTTGCTATCATCATTTTGAAATTCTTAATCTATGAACAAGAGGTTCTGCATTTTCATCTTGCACTGAGCACTACAAATTGTACAAATTGTTTTGCTGGACCTGTCTGGGTGACACTTGTTCTTCTTGACTATAATGGCCAGCCCTTGGCTTCCAATTGTACCCTGTTAACACACGCGATCTTTCTTTAAATCAAAGGAATAAGTGAATAAGAATATTTTCTTTAAACTAGATATTTTAAAAATTAACTTCTGAAGAATGAATTTATTGATTTTTAATAAACTAAGAAAATATGTAAATGGATTTCCAGTCATTCAGTTCTGACTTTGGTGGGTTTGTTGTTGTTTCTGTTCATTTTAAGCTAACATTCAAAGTTAGTAGTGTCAGTTTTACGCTTTCCAGGAAAAGACAACGGAGTATACTACTTGGCTTATATTTCTGTAAAGTATCACTTGAGAAGCGTTTCAACTGAAATGTGTTTTCCCACTTTGATTGTGTGTAGTAGGCTGATTTCTCATCAATCCTAAGAAATATACCTTTATGTTATATTAATTGAGGATTTAGCATTGGTAACAAATCTTGTTTTTCTTACGTACAAAATCTCCCTTGGGACTGGATATAATTTAAAACTATGACGCCTAAAAGTAGATCCATCAGTACCCACATTGCTATTCATGACTTTTGAAATACAGTTTCTGCTTCTTTGTTGCTTCTCAATGGTTCAGTCTTTCTATCATTCCAAAGGACTCTTGTGACTCCTCAGTAAATGGAATTTTCCCTTTCTTTTCCTTAATATCCATAAAATCAGTTTACTCAAGAAATTATATAGCAGTATACAGCCTAAACCCTTTCTCAAAATTGTCACTAGCTTTTCTTATTCATTAAAACATTTTCATCTCATTACAAATGATTTCTTCCTTTAAAATTTCCCAGAAAGTATGGCCTCTGTATTTTCTGCTGCTCAGTTTAAACACATCATCAAAGACAGATAATTATTAATGTCATATGAGCATAACAGGAGATTTATAAATCAGAAATGTATTTTCTGGGAACCAACTTTCAAGTTAGTCAGGTAAGTTTTATTAACTTCACTGGTGAAGTCCTAGGATAAAGCAGTGCCTAGATCAGGCTTGTTTACCCTTGGCCTTATATGGCACCAAACACTGTTTCAGGACAGTGGAAGAACAGCCTGGGAGGCCTTCGGCCTTGGCCAGCCTTGCCGAGGCCCGCTCACCAGGAGAAATAGGAAGGAAGGCCAGTGAGTGTGCAAAGGAGCATGGCTTATTTTGAGGTCCTGAAGGCCAGTGAGTGTGCAAAGGAGCATGGCTTATTTTGAGGTCCTGCTGATTGCAGGCCAACAAAACAATGGCAGGATTTGAAGGAAGAAAGTATTGCAACAGAGTCACCGTATACTTTGAACTGCAAAGGGAGACTGAGGAAATGTTGCCTCAAGGCCAAAAACCCTGGCTCAAAACACAGGTTCCTCAGGTGACTCAGAGGCAGACCAAGTTCACCAAGTCAAGGACATTTTCCTGCTACCCCCCTGGGGAGAGGGGCCTTCACCAAACCACCCTGGACATGGATCTCCTGCTGTTCAGAACCCACCCAATAAACAACAGAAGCAAACACTTTTTTTTTTTTTTTTTTTTTTTTTTTGCCCCTTACACGGAATTGTCCCTTTTTGTAAGTCCTTGTTGAATTGAAGAGTGTTTGCTCACTGGCTTTTAAAGTGTGGTCCCAAAACTTTTTTCTTCGTTTATATTGGGTTAAAAAAAAAATCAAGGGGCCCATGCGTCTTTGTTCTCATCCAAGCCCACAATTAGTTAGCTGGGGTTCCTTCTGTGTAGCTAGAAGCAGGAGGGAAGGGGGTGGCCCAGCAGGCATTTTACTGCCTGCGAGAAAGTGCTGTGAGTTTCTTTGTCCTTTTTGTCAGGGACGGCTGCCTCCCTAATTGTACTGCTGTAGGGAGGCACGTGCCTTTGGGAGCATTATCTGATTCCTACAGGACGTTCAAGAGTGCTTTGTATACTGACCCTTAACTTGAACACAAAGAATATCTTTTCAATCCCCTTCCGAGGCTGTTGCCTCAGGCTTGGATACCTAAGAGCTTTAGTTCAGCCGGGGTATGGACCATTTATATGGGCTCAGGTTTTATTTTTTTGTCCCCTAGTTGTCTTACCTGCAGTTTGGCCACACCTGCCGGGAGAGAAAGCTGAAGATGGTGGGTAAGTGGTGGATAGCAGCTATAGAAGTCAGAAGGGAGAGAAAGCGAGACAAACAGCTGCTCTTGTCAGCATGGGGATAAGTGTAAAGAGATTAATAGTTATTTGAAGAGCAAATGGGGACATGGCATTCGGCAGTGGAATGCGAATGCATGAATGTAATGGAATGAGCATGTGACTATTAACATTTAGACAAATGCTTCCTGGACTGAATGGAATGGCAAAGCCCACTTCTCCTCCCCCTGTGGAAAAAAAAAAGTTCAATAAAATAATTAAAAGTTTGAAATTTTTAAAAAGATAAGCCTAGCTCCTATTTCTTTGTAAGTTTGTTCTTCTCTATAGTTTTACTTAATCTGGGACTCTCAGTTACATTTCAACTGAAGAGCTAGGCCTATTTCCTTTATAAAATATGTACGAATAGGACTGCCTTAATCTTGCTTTTGATTTTTTTTTCTCATTCATTAGGTTGATGAAACAAGATTTCTATGTGTCGGAAGGTTTTACAAAGGAAAAGCAACTGTACCATAAGCTGGTTGCCCCTTTGTTCATTTTGGCTCAGGTTTAGAAATGATGATAGGCTGATTTGCCTACTCAGGCTGACAGTTTTGATGGCATGGACAGAGCCCTGAAGTCAGAACCAGAAGATTCAGCACCGAAAGCCCCAGTCATTGTGTTAGCGGATGCTAGGGCAAATGCTCTACCTCTCCGATAGTAATATAGTCAAGTTTTTTGTAGAGTAAATGAGGCAAAATTAATTTATTCTTTCATAAATATGTTCTTGAGTACTTTTACTCTGTGCTTGGGCCCATTCACATAGAAGACATAAAAACGTTTGGCAGAAGGAAATACATGAGCTAATGTATTTTTGAATTATATTGACCATAGAATTTTCTTCATAAGTAGTTGGTATTTCTCTTACAAATGTTCTACTCTAGAAAAGAATAAATTTCTATTAAGAAAATGAAAGAACAAATAAAAAAGAATTAGACCAGTTTGGATGATTTACTAGCATGTTACTTTTTAAAATACATAGTTGGGTGTCCCTAACCCCGCTACCCATGATCCTCCAGTCTTTGTGAAACCAGTCCTTAGGGGGCTGGGTGGTACACTCTTGGTACTCTGGGACAGAATGCAGAATTGCAAGATTAAGAGAAAATTCGAAGGTCATTTGATTTTGCCCCCTATGCAACAATTTAACACTTAAATCCCCTCTGGAACATACCTATCAAGTGGTCTTTCTCATTTTGTGTAACACGCCCCAACCCCCACCAACAACCTCAAAATGGAATAGCAGGCAGTATTTATTTAAAGACTTCCTAAGCAGCTACTGAGCACTGGGAACGTTTTGCCTAGTTTGTGAATAAGCATATACTCTACTTTCAAATGCTCTGTAGGAAGTCAGTCTCCAGAGGTGGCACATAGAGCTGAGTGATCTGGAAAAGCCGTTACAAACACACACATGCTCAGACTTCTCTTGTTTTCTCTGTTTACCAAAAATCAACGGTTTTACTTTGAATGTGAAGTGCTTCCGACGTGAGGTCCATCTCTGTAGAATGCATTCACCCATGGATGCCTTTTTTTGGAGCAATGATCCGTGGCTTCAGCGGCTAATATTGGGGTTTCTGGGTGTCCTTGCAGCCACAGTACACGAACCTGGGGCTCCTGAACAGCATGGACCAGCAGATTCAGAACGGCTCCTCGTCCACCAGTCCCTATAACACAGACCACGCGCAGAACAGCGTCACGGCGCCCTCGCCCTACGCACAGCCCAGCTCCACCTTCGATGCTCTCTCTCCATCACCCGCCATCCCCTCCAACACCGACTACCCAGGCCCGCACAGTTTCGACGTGTCCTTCCAGCAGTCGAGCACCGCCAAGTCGGCCACCTGGACGGTAAGAGCAGCGGGCACGCACATACCTGACCCCCCAAGTCCAAGGATGGGCTTCACCACGTCCCAGGGATTTCTCCCCCTTCCCAGTTTAGCGATTCCATGTTCATGGTGGAAAATTTGTCTTTGAATATTTAATACTGAACCAGAGAGAGAGAAAGTGCCAGTTAGTGGGAAAAGTCCCAATTTAGGAACCAAACGTCTGCGTTCTAGCCAGCTCCTGGCAACTTCAGTCAGTGTGTTCCTGCCTGCTTCACACTGTTGTATGTGAAAGTTCATGGCAAGGTAAAGAGCGCTGTGCCAACAAGAAGTATCATAATCATGGGATATGTGTTTACAGTCTCCAGAACGTGAACGTGTTTACCAGTCTCATTCTTGTAAAGAAGTAAGTGTCACATTAAGTTGTGATTTTAAAAACTGAGTAAAATAATAAATATAGTGTTATAAAAATTAAGAAAATAAAATCCCATTTATGAAGGAAGGATGAATATGTTTATACATGTAGGTATCAATAAATTATAATTTATCAGTAAATTGTATAATGTCTTATAAATTACTATTTATAATTTATCAGTGAATTATAATTTATTGATATGTATATATTTATTTAATTTGGAAACATTCTTTTCGAAAAGAAAGCCTTTAAATAGACATATCAGAGCCCTGAATTAGTCCAAAGAAATTGTTAAAGAAAAATCTTTCTGCATATAAGTTTTCTAAGGAAGCATGGAATTTTCTTTTCATAAATTAGGAAGATACAAGAATTAGTCCAAAGAAATTGTTTTAAAAAATCTTTCTGTATATAACTTTTCTAAGGAAGCATGGAATTTTCTTTTTCATAACTAGATCCAAGAATATTTTATTTTGAAGTGTATTTTGAAATGTATTATAACAAGAATAAAGCTCAAGTATAAAAGAGGACACCTACAAAGTTGGTTTTAACTGGTTGATCAAAGAATGTTCAAGACCAAAGTGGTATGTTTGTCAAACAACTTAATCAAGACCAAAGTGGTATGTTTGTCAAACAACTTAATGTTTACATGGATTATAGAAATGCTTCCAAATATCATCATCTTAATAAAAAGTAGCTTTAGAATTGTTTTGACAGTTTTTTCCCTCTTTGACAAATGGATTTCTTTGGAAAGCATTTCTATGTTTATGTTATATATAGTATCTAGTTCATTCCAGCCTTTAGTTTGTATAGTTTATAAAATGATCGGCATGCCTAAGACCTATTACACTCTAACATCATTTCACATCCATTTCCTCTTTTTCACCTCATTTCTTTGTGAAGTACCCAGGACAAGTTTTATAACTTCCACTGTGCTAGTGAGGAAAGCTGAAGAAGTTGGAGGTTAAGTTAAGAGGCATGCCTAAACTTACAGAAACTTGTGTGTAGCTGGGCCTGTTTTTCATTTCTGCTTCCCTCTCACTGGATTGTATCCTTTCTTTCAACCATTTTATATCAGGATCTCCCAAAATTGTCTAAAAATTAATGTTATTTTAGTTCTTTCCTCTTGTGGTTTGAGAAGTAATTCTCCGGTGGCTCATATTTCAACTGTAGAGCATGAACATCAGCTTAAAACTTGATCTCTCCTCCACGTGTTACTTATCAATGGAGTCATAATCCTTAGTTTCCTTTACTTTTATAATTCTGGAGGCGATGGATACTGCAGGTGAGTAAACTTAGAGTTTATAGGCTTCAAATAGTTGGTTGATTGATCTAAGTAGTATCTTGCTAAAGAGCCACTAGAATAACCATTTTATAAATGGAGCACTGATATCTCTTAGGTCACCAACCGGACATTTACAGTCACCAGTCTTCAAAGCTAACAGTTACTAATTCAGTTTACTCCGCCAGGTCTCCATTATATTAGGAGTCAGCATGTCTGATGAGATGATGGGCATATTAGGGCTGCTAAATGAATAATCGCTTTTTTTCCCTGTAAAATTTTTAGAAAACCATGGGGATGAATCTTGATGTAAGTTCAACATCAAATTAGTTATCCTTCCCTGTCCCTTCCTCACTACCTCCTCATATAGAAATCTTAAAAGCCGGGCGTGGTGGCTCACACCTATAATCCCAGCACTTTGGGAGGCTGAGGCAGGCAGATCACGAGGTCAGGAGTTCAAGACCAGCCTGGCCAACATAGTGAAACCCTGTCTGTACTAAAAATACAAAAATTAGCCAGACGTGGTGGTGGGTGCCTGTAGTCTCAGCTACTGGGGAGACTGAGAAAGGAGAATCACTTGAACCTGGGAGACAGAGGTTGTGGCGAGCTGAGATCGCACCACTACATGTGATCTGCCTGCACAATAGAACAAGAGTCCGTCTCCAAAAAAAAAAAAAAAATTAAAGTTATTTTCAGGTTTAGCATAATCCTAAACCATCACTCAAAGCCCCTCTCTCAGAGAAGTCAATTGTCTTACTCAAAAAGTTGGAGATCTCTACCAAAATGTTAGGTTTAAAAGAAAAGAAAATATTCTGGTACACTGGTAAAATTTCCTTACATGTGACAGCAGTAATATCAGTGCCCTGACAAAAGGGATACACTGTGGTGATGAGAAAAAGGCACTGACTGAGAGACAGAAAACCTGGATTATGGTCTTGATCATTTATGCAAACTTAGCCAAGTCACTTGATCTTTCTAAAATTCAGTCTGTGCCTCAGATGAGAAAGAGGATGAGAATGGAGAAACCTACTCTGTTTTCTTAAGGTGAGATGATAGTATTAATAATTAACGTAGAAAGTTTTTAGAAGGGCTTTGGTTCGCATCGATATATTGTCTGCCATTCATGAGTGTCCTTGGTTAAGTGATTTTGCTTCTCTTGGATTTTTGTTCACTTTTCTGCATTCTACCTATAAATTTTACAATCAGGTTTCTACTCACATATCACATGGTAATTATATTTTTATTAAATTTCTTTCTTGTTCACAACCAGGCTTATTTCAGGCCCAGTTCATTTCTTCTAAATTAGGGCACTTGAACCCTTATCTGAGCAGCTACTGAGATCACCTAAAAAGATGTAAGTTCTTCGTCATGGATACCTGCATCTTAGCTTCTAATTCTTTGAGCCAAATTATATATCCTCATGTTTATACACAGACTTCCCATTTTAAAAAGTATTTTTTCATATATTTATCTTAGTTGAGCCTCATAACCAAGTTTGTAATCCCCAGTTTTATAGATGAGGAAACTTGAAGATGTAAAGGTCACATGGCTAATAACCAGCCAAGGATGGGGAAGAATCCAGGCCTCCCTCACCCTAATTCAAGTCTCTCCACCATCCTAGTTAGACTGTTTCTCCTCTGGTTTGAGACCAGGGAGGGGCATACAGGAGCAGGCAGTGCCTGTGTAGTTTGGAGCTTGCCTCTTATGTCCCAGAGTCCAACTGGTAAAGAAATTTTTTGTGCCTTATTATACAACACCTATTTCTTAGGCATTTGTGCATTTTAGAGCAATTTTTCAAAATAAAATGCTGCATTTTAGAACTGCCCACAAACTGAAAGAAACAGAAGCTGCTGTCATTAGGGGGAGATCATGCTTATCGCATAGTCTTCTGGTAATTCTTTAATAAAAATATTTTTAGTACCATTTTATCACTTGCAATCCTTTTATGTCAAGTTTTCTTTTTTAACTTTTCTGGTTTTACCCTCAGGATTACTGTCAAAATATAGCTAATATATTTAAAGTATATGTGCATTTTTCCTTTGCGTTTCTGTGTTTTAAATAACAAATGTGATTATGTTTCCAGTTCTACAAAAATATGGTATTTATTTTAAACTTCTTTCAATGAAATGAAGTAAAATAAGACAACATTTTTCATCTTCTCTGTTTCACACACACATACATACACGACAAACCAACGTTTACTTTAGTTTCAGATTTTCTTATTTCCACAATTTAATTGATACTAGTTACAGAGGCCTATCATTTAATATAATGTAGTGCTGGTCTAGCATTGTAGTTGGGAATCGTTAATCATAGGACAGTTGAAAGAATGGTAGTGTGGAGATGGCAAGAAACTTAAATTCAGATTTCAACTCGGAAACTTACTAACAGCATATGGGGCTTTCACCAAAAATTCAATCTCTCTAAACCTCACTTTTCTCACCTACAAATGGGAACAAATGGGAACATTAATACTTTCCTTATCATGAGGATTACTTCATGTAAACATACCTATTAGCACCTAGCTACTCATATGCTCCCAAGTGTTTATTGGGTCTGAATTTGAATTTCTTCGGTTCGTGAGTTCATTGTAGTTTTGTTGAGGCTTCGACAAAACTTTAGTACCTTTAGTTGTACTAAAGGTACTCATTTGAGAGAATGAAAAATAAAATTTCAACTAATGTTTTTTCTTAAAGCTTTTGGGCTATTAACATGCATACTTTATAGAGGGACTGTGATACTATCCACTATCCAAATATATGAATAGATGGTATAGGGTGATAACAGCCTCTCCTGCATGCAGACTTCTGCAGGCTGGCTCTGCTGCTTTCCGAAGCCTGCCCAAGCAGATGTAGGCTCTCCTTGCCAGTGCAAGGAGAGGCCCTTTCCAGATTTAAGAACCTGTTGATTTTTGACATTCGTTCCTCATTTTTAATCTGATGCTTCATGATGGGAATGCCTTTTTGTATTCTTTGTGTTAAGGAATAACAGTGGGCCTTACTTATGGCAGACTCAAGATATTAATGTATAATGACAAACTACTGGAAGCAACCTGATCAGTCACCCGTGAAAAACTAGATGGCATGTTATAAGCAATAACCATACGGCCATTAGAAAGAATTCTCTGTACTGATAAGGAAAGCTTTGGGAGTTGCTGGTGAGGGAAAAAGAAAGCTTTCTGTGGCACCTGCTTGAGAATTGAGAGTCCTGGTAAGGAACCCCACTTGTAGGGGCGGTGTTTCTTTGTGCTCCCCTCTAGTGATGAGGCTTCTTGGTGAACATAGTTCAGTCTGTCTTGATTATTGGCCTCACACACCTTTGCCAGTTGGAGAGCTATTTTTCACCGCTCATGACACGAGAGTACTCTCTGTGTGCATACGCCATGAAAATCCTCAGGTTGTTTTTTTTTTTGCATTGTTTTGTTTTTTAAATTGTGTTCAGACTGAAGTAAGGACCCTACCCTTGGGACATAGGATCACTTTTTGCTGTTGTATCTTTCTCCTGTCATTGACCTCAAGGTCTTGTATGGCAGACTCCCTGATCTAGTGCCCAGCACAATGTCCAGCACACAGTAAACCTTTAATAAATGGGAAGGAACTTTTTTTCTCTCATAATTAAAGGAAACAATTGTAGTTCCCAGCAGGAGCCCTCAATTCTGACTCTGATTGTTAACTGCCCGTGAATTTGTTCCCTGTAGCCTTCACCCATGATTTATAGAGAGTGTTCTTCCTTTGCCTTTCTTAACCATGCCAGCTTCGTGTCATGTTCAGAGCTGTGGAGAATTTGACTTGAGCAAGTGGAGAGATTAGGGAGGTGGCGTTTGAAAGGGGAACAGCCAGTTCCCGGGAGAGTTCCATTGATCCCAGCATATGGGTCTTGGTCTCGCAAGCTCACTCCCTTTGGCTCCGCAGGCAGGCTGAGATGCGCGCCTGCGGCCCACCCTCCCTGTCATCAGGTTACCTGTAATGTAAGACTTTTAGAAAAAGGCTGCCAGCTCTGCAGAAATCTAATGCAACCCAGCCAATTGCGTGGCCCTCTGGAATTTAGAGACTCTTAAGAATAGCCCATCAAAGGACAAGATGTACAGACAAACTCTTCGATGGTAAATAGGATTTATCAGTTTTCTTTCACAGTGGAAGCACTTCCCCCATTAAAGTCTGAGTCTCATCTGGGGCTTGATCTAATCTTACAATTCAGTGGCTGTTAGATTTTATTGGTGAGGGGCAGAAGTCAGTAGCTCGTGCCACTGGTACATGTGTTCGTGTTTATCATAACAAGATAATTTTTACTTCTTGCCATAGCTGTTTCATAAATTATGTGAGATGTGTATCAGGTGCCTTTTTGTGCCACTCCCATGATGAAGAAAATAATAGCATTGAGCATATTAATCTGTTATCTAAACTATTTCTTAGTTCACAGAGTATTGAGGAAGAATGTACTAAATAAAGTGCTTAGAGATTTTTGGTGATGGTTCAAGTAACTTACCTCTAGGTTTTGGATTTAAAATGAATATTCATTGTAAGGCATGCTTCCGAAGAGTTGTTCCCTCATCCAGAGCAGAATAACTGAATCAGCGTTCTTGCCTGAGTTTTAGATTGACTCTAGGTTAACGTATTCGTGATGAGCCAGTTTAATCTAATCTAAGGCTTATCAGCCTCCCATACTATTTTGAGTGATTTGTTCACTGCACCGTGGAGTGTCTATTTAACATTAAACTGATCTTGATTTATTGCATGTGAACATTATAAAAACTATTGATTTTAAAAATGAATAATTTAAATAAAGCTTATATTTCTTTTCCTACTACAAAGAGTAACAGCAAAAATCCTCTGCTTTTCCCTTTTCTTCTTTCTCCCTATTCTTCTCCTCTTCCCGCATTTCTTCTCCCCCTTCTTTCTACTCCCTCCTTCTCTTTTTTCCTTCATTTATGTTATTATTGTTAGTTAATTTGGTTTTGTAGGTATCATCAGCTACATGGTATCTTTCTTTATTTTTTTTTACTATAGCTTCACTCTTCTCCATAAATTAATATGGAGAACATGATATATATCAATCACCGTGGAAATTTTTATCAGGATTAAATTATCACAACCTTTTCATGTAACAAATCACTTTGGTGAATAATAATGGGCTGCTTTAGAGGTAGCAAACCCAAATGTCATTGGAGTCAGGGAGGTAGCATAAATGAGCCGAATGATAAGTACAGAAACTGTGGCAAAAGAGCTTATATACTGTATCTACAAGGACATCTGCTTTACAATCCCAGACTGTTGTCACAGGGGAAAGTAGACCTAGTATTAAAATATTTAAAATATTAAATTAATTATCACAATATTAACTCTTAAATATTAATTTTTAAAAGAGTAACTAGAAACCTGGGTTTGCATGTAATATCTTCATATCTTAAAATGATGATACTAATTCAAAAAAAAGTTGTAAAACACTACAGGCCAAACAAAACAGTACACAGGCTGCATGCCATCCTGTGCAAGGTGTTAATGGTAACAAGGGGAGATTACTAAATGTTTAAATAAATTAATTGGAAGTTAGTTATTAAAATAACTAAAATAACTGGAATCAGCATCTAAGAGAAAGGTTACTAGTGGGTATATTTTATTCACAAGTAATAATTCTTTATTCCTTTACAAAGTCATATAGATTAATCTCTTGCTGCATTTAACTAAGCAATACATTATCAGAACATATTTTTCTCAACTTGTTTTCTTACAGCAAATGACCTTTCTGCTTCTCATTTTTATATCATTTTATATTTTAAAAATCTAATGGCATGCAGGATTGGATACTTGGAAGTTTATTGTTTGTTTGTTCCTGTGCTTATCATTAGAGAATTATGGAACTCTGTAACTTAACTCCTTAAAATTGGCCAAAGTTGCACTCAGAAAAGAGCTTTGAGATTTAAAGGGCTTCTAATCAATTTTCTCAGCTTACTAACAGATAGCTTACTTTTAATTGGCACATAGTGTGCCAGAACAAAAATCATCTGATAATTGAAGTATAAAGAGAAAATAATGTCATAAGAACTTTTAATGACTCTGGAAAACTATATCATTAAGCTCAAAAGAGATCCCTTAAAATGAATGCAAACTTAAAATCGGCCCTGAGTGATGTGTACCATTTATTGTGGGATTGTCGCTCTTGGTACATTTCCATACATGAGCTTTGCACATTCTCCTGTTCGCATTCCCCAAATTAGGTTATTTCCATAACGTGACCAAGTCTATGCAATTGTTCCTACCCGCTTTGGTAAAGGGCCGATGTATGTTTCTCATGAAAAGTCCTCAGCAGTTACAGCTGTCATATCGCGAGTAAAACCATATAACTATATATAAAAACCCCCTGGTTTTATATTAGCAGCAATTGGTATGTCTAGTCAGGTCACTGTGACTGAAAGGAGACTTGAGATAATGTAGCTTTCCAACAACTTCCAATTCTTCAAAATTAAAGAAGAGTTTATTGTAGAATAGAAAGATAGAAAGATAATTCTTCAGTTCTTTGAAGCCCTGCAGACATTTTGTAGATATGGAATGGTTTTTAGAATGATGGATCCTGTAATGAGGCACACAGAGGCGTAAAGGTAATGATCTTAGTAGATTTCTCTCTTGTGGTTTTTAGTAATGATCATGACCTTATCATTAAGCCCCCCACGTGAAATTTAAAAGAAAATTTGATAGTAGTCAGAGACCCTAGATCTAATTTATTGTGAGGTAAATTAGGGAGGGATTCTGACTTTACAGATAGAATGAGACTTCTCATTTTAGTCTGAATTTAATAATGAAGATTCATGTGGGGGAAAAAAAAAACCCTACAACTTGCCACCTTGTTATTGTATCAGAAAGGCCTGAAAACAGAGCAGGGACCTTAATTGCTAATTGTGTTAGTACCAACCTTTCAAGTTCATTGATTAAATAGGAGTTCCTTCTTGGATTAATGCCTAAGTTTTTATTTTTGTTTTGCTTCATGTGCTGTTCAGCCAGTATCATCTGCATTGAATCTTATAAAAACTATAATGAATAAGTTGAGGAGACTATTTCTAAAGACTTTTCCTTTGAGTGGTTTCCAAGTTGTATTAAATAAGAAGACTAATGATTACTTAAGGGTTCTCATTTGAATTTGAGAAGACATCTGACAGGCTCCTGAGGAGTGTGACTCATTAAGTAGAATACGGGACTCAGAAGGACAACTTTGGGGTACTAACAAGCTATATGAACTGGGGCAGGTCTTTTGTCTCTGTCTGTATGTTAAATGAGTATTATAATTTTTACTTGGCAAGATTGCTGTGAGGCTTAATTAAAGTTTATAAAGTGCTTTGGGAGCCTGAGAGATGAAGCTCTAAAAACATAATATGTTATTATCTCATCCTGGATTTTTGTTTTTAGATTTACTTGCTTGAATCTGTATCATAACGATGCTTACAATTCAGGCAGTTTTCTGAGGTTTTTTATTTCTACAAAGACATGAAATAAAATAACATAAATATATAAGTCCCTAGACATTCATATGGGATGAAAAAATTACACATAATTTTTTTCTGATGTAAAAGAGTTTCTTCTTAAAAAATGTTTAATTGAATCTTATATCTGTTAACCAGCTTTATTTTTTTAAAAGCATTTCAGTGTTTTATTCGTAACATTTTTGCAGAACCAGTAAAAAGTTTTAAAATGCAGTCATCTTTACTGGGTCATGAGAAGAAAAATTTACTCCATTTAAAAAATGTAAATACTTATTTAAATGTAAATAAATATGCGCATTTAGATATATATAAATATAAATATATTACTCCAATTGAATAACAATGGAATTAATTTACAGTAATTAGAATTTGATAATTTGGGCTTGAAAGATATTCCTTTCTTCTATCTTTCTGCCTTACTTTTTTCTTCTACTGTGTGTTCATTTGAGATTTTCATATTTATAAAATATAAAAATGTATATAGAGCCCTGGAGTTAATATAAATTGCCTTGAAAATTCTTTTTATTCCTACTTTGTATTCTTGAAAGAAAAATCATGTTTTCCCATAAGAACAGTGTCATAATTATGGATAAAATTTCTGACCAAGCTATTGTCATCAAGTAAGTAATAGATATGTTTAACTCTATGTCATAAAAGCAAAGCTATTACCACCATAAGCCAGTATAATTATTGGAAATAACAAAATGATTCTCATTGTCATATAAACTTTGCTTAAATATTTTATATGCCTTGACATATATGTATTAAGTTTACTTATAAAATGTTATTTTTATAAACTTATCTAGAACTGACTGCTATTATAATAAATACTTTTAACATTTTGACATGCCATAATTTTGATCCATTTCATGGGGAATAATTTGAGAAGCCTATGTTTCTCAAAATTTTCGTAGGAAAATTAGAATGGATACTTGTATAATATATTCCTGTTACTAGGAATGATGCTTAAAAAAATTCTATAGTGTAGGTCAGTCTTCATTTTAAGCAATTGAATCCATTTTTAATGATATTACTTAAGAATTTTGCTTGTAAGAAAAAAAACTGTATCTGATTGAATTTGAAAATGTAGTTTTGGAGTGTATTTACTCTTCCTGCCTGATACCCCACTCCCCAAAACAGAGTAGTCTTGAGGAAATAAAAGGTCCTCAAAGGCAGCTAGATATGAGAAGAGTTTGAATTGCTAATGGGGAAACTTCTAGACCTTTGCCTTGATGTTAGAAAAGGAATTTGAGAACATGATTTATGTGTTAGACCCAGAGTTTGTCTTTAAACACAATTCAAGTTTATCACCTTTCTTATAACCTTTCTATACAAAACCATGGATTTAGTGAATAAAGCATTTGGAAGACGGTGGATAGTTGATAGATAAAGCAGATTGACCAACATGTAAAAATAGAAAGTGTTTTCATAGTAGGAATTTGGAATAACTTTTTCTATATTCCCATAGTTAGAATGGATGCCTAGAAAATGTATCCTTATTGTGATGATTAAAAATCTCTTTTTGAACTAAGACACGTAAGATAGTAACCTCTTTCTGCTTTGCTTATTCTCCCACCCAGGCTGTGTTGAAGTTATATAAGTATAAGCAAATGTGCACTTTTTTTTTAAATTTTATTATTATTATACTTTAAGTTCTAGGGTACATGTGCACAATGTGCAGGTTTGTTACATATGTATACGTGTGCCATGTTGGTGTGCTGCACCCATTAACTTGTCATTTAGCATTAGGTATATCTCCTAATGCTATCCCTCCCCCCTTCCCTCACCCCACAACAGTCCCCAGTGTGTGATGTTCCCCTTCCTGTGTCCAGGTGTTCTCATTGTTCAATTCCTACCTATGAGTGAGAACATGCGGTGTTTACACTGTTTTATAAAATGCTTTTGTCTAATTTTGTTACCTTTAAGTTTAGCTACTTGGCAAATAAACATTTTTAATACTGCTTAAGTCAAGTCTTTTACAAATAGAATTGTCAACATCCAAGAGTTACCAAAAGATACTTCATTCTTTAGATTTTTATTGTTCTTTCACAGTACTCACTGGTAATGGATTATATTTTACCTTTTTTTTTTTTTTTTTTTTTTGAGACAGTCTCTCACTCTGTCACTCAGGCTGGAGTGCAGTGGTGCCACCTGAGCTCACTGCAACCTCCCCCTCCCAGGTTCAAGCAACTCTCTGCCTCAGCCTCCTGAACAGCTGGGATTACAGGCGCGTGACTCCATGCCCAGCTAATTTTTGTATTTTTAGTAGAGATGGAGTTTCACCATCTTGGCCAGGCTGGTCTTGAACTCCTGACCTTGTGATCCACCCGCCTCGGCCTCCCAAAGTGCTGGGATTACAGGCATGAGCCACCACGCCCAGCCCTATTTTACTTTTATCTATTGTCAATGACTACATTGAATACTTTTGTATGCAAATGTAACAAAGGCATATTTTGCAGTTATTTATACTTTATGTCTGAGAATTATCACATAATTTAGCAAACATTAACTCATCTGTTTACCTTTTGCTGTAAAAGCTAGAGCAAGTGATGTAGATGGGGACAAGAAAATGACTCACTCAGCCTCAGAACCACAAGGAACTATAGACTTCTTGCAGTCTGGTTTTCTCTTCAAAAAGCAATTAAATATACGACCATTTGTATAAGTTTCCACAATGCATTCATATTTCAGAACTGATTTCTTACCTAGCACGGCATGGATTTCAGTTATAATGTTTCTCCTTGCACAAGCATGGTAGACATGGGGTTAGTAGGTATAAGAATGCCTCTGGGAACAATCTTTTAAGGTTTTATAGACTCTTTTCTCTCACCACTCCCCCAACCTAAGTGTAATTGTCATCTTAGACACTTTGAGTTTAGGGGTGGAGATAGGGAGAGCATCATCAGAAGGGACAAAAGGAGAAAGGGGAAGTGAAATGGTTTGTTGGATACTAGTGAGTGGAGCCTTTTACAGAAGTACAATGCACGCATTTTACATTTGTTAGTTAGATGTTTTCTAAGATGGTTTTGTAGCTCTGATGGTATATTTTTATGGATATAAACTTCTAATATGAGGAAGAGACCCAAACCAATTCAAATAGCATGTTAAAATTCAATCTATCTACAATCTTCACGTCCTTGATTATTACATTCTGTAATCTCATGGGTATTTTTGCTTCCAAATATAAGGACATACCTATTATTTCCTTCAGTTTTCTTTTTAGGAGAGTTGACTGAACTATTTCAGGAAAGAAGATGTCTTACTTTCCTTTGCCTAGTAAGCCTTGCTGCTTTTTGAACACAGTGCATTTTCTTCAAAATATACCCACTACTTTAGGTGGTCTTAATGAATGAATAATATGTAATTGTGTATACTTTTCTAAAATTCTTTGAAACTTCCAGGAAGGAAGGTCTCATTAAATATCTATTTGAATTTTAGTGAAAAGAACAAAAAAAAGCAATAACAAATTAAGACCTTTGATTCATGTGTACTAGCAGTGGTTTTATTTTGGTAGCTAGTGCTGTGATGTAATTGAAGCAGTATTTTCATATAGAAGCATTTTTTACTTTTATGTTCTTTCTACTTGTTAGAAGTATTCTTTTATTATGGTTACTTTTGAATGTATGTTTCCCTTGCACTAACTCAGAATACAGCCTCACTTCACAGCGCTTAGATTTGATTCCTGCATTCATACTGTTTGCATTTGAGGGTCCTGGTGACTGCAGTAATCTCCACCTCCTCCAGAGCCAGAGAGAAAGAAATGGCCACAGCTACTTTGTCATCTTTTGTCACTGGTCTCAATAGAGGGTCAAGCGTGCTTACCACATGGAGGATGCTTCAGAACAACAAGGGAAAGAATTAGAGTGTTGCCTGGGTACTGCCCACGCTGCTATATGAACAGTGAGAGAGGTTATATTTTCCTAAGCCTCGCTTAAAGCATTCCCAACTAACAGATTTTCTGTTTCTAATGGTTTGAACTTTTGTTTTTAAGTTGTTATTTGGGCATGTGGTTGTAGGGCATAGATTATCTCCTAATTTATTGCTTTCTTAATTTGACATTGGAATTTAGACTTCAGAAACAAAAAATGCAATACTTTGAATTATCTTCTTTCCATAAGTTCCTGATTTAAAATGAAAATGTCCCCTTAACTGCTAATTACGAGATATCGTATAAAGCATATGATACTTACAGTATATCTTTTTTGGTTGTTTTATAAGATGAAGATTACAAATATGATATTTAGCACATCCTGGCTCTTCTTGAAAGCAGGGACTATGGCTCTTTTACTAAAGGACAAATAGTGCCTAAGGAAACAGGAGGGTGTAGGAGAAAGAATTCAGCTTTGTGGTCAGATAGACCTGGGGGGTTGAATTTCACTTCTCTCTAGCTGTATGGTGTTGGGCTAAACACTGTCTCTAGAGCGGCTTCCTCACCTTTCAAATAGGGATGATACTCCTTTAGGTAACGATGTTATTGGTAGGATTTGATATAATAAGTATAAAGTGCCTAAAACAATGCCTGTAAATGGTAATTTCTATTAGGATGGATCATAGATTTAGGATTAGAATTTACACTAAACCTACCTTATTCATAGATTTGTCCAGAATCATAGCAGGAAATCTACTCCATAAAGTAGCTCTAGCCTCTGGTGCCTAGTAGAGGTGATGTATTAGGATAGTACTTTAAAACTTTTACCCCAATTAAGAGCAGAGGAAAATGAATGGCTAGATTAGCCATCCTGAGGGTTTAATCTGAAATGGTCCATAGAAGACATATCATTTTCTTTCTGAAAAACTTATTTCAACTTTGCATTCAAATATAAATATATATTTCAATATAGGTATTTCTTCCCCTCATTAAAAAGTCTTTAAAATTGTTGTTCCAGGAAGATGAGTAGTATTTATCTTGTAACATTGAGTTATTATACCCAGGCGCACTACCAGGAACACTTCTGATGTACAAGTGCCTTCAATAATTGTTTCAGAAGCACAATTTTGAGATAAGTTAAAGTGCCCTGGGAAAGAGCATTGTTTATTTTTGGAGCCTTAGCCCAAAATGACTCGGACTCATGCTACAAAATGATAACGCTCTTCATCAGTTGTATAGAGCTCCTTCTTTATGGCCAGCTTTCCTAGAGATATTTTAGACTTCTTTAGATGTCCCCAAAGGCACAATAACCACGTAAGAAGGGAAGCCACAGGAAGGCAGATTGCAGCAGAAAATGAATGGTCAGGGTTCTCTAGGGGTGGCTGCACTGCCTCCAGTGGTGGTGAGTTCTCCACCTCCAGAGAGTGAGACACCCTTGACTCAAGTTTTAGATGAGAGAAACAATCAAGCAGCCTCTGAAGTTCCCCTTGAATTCAAATTCTTTGCATCCAGAAGAGAAGTATTTTGTGACCAGAAAGATACAGATTGACTGCAATTGACCTTTTCTATGCCAGACCATTGGTTAACTGAAGAGAGTAGACTCTCCAGACAGGCAGTGGACTCAGCCTACACCAAAACACACAGCAAGCCTTCTGTGCCTTTCCCCAGGACAGTGACTCTTCTTTTCTCTCTGAAGCTGTGGTTCTATGTAGGAAACTACATAGGGTGAAGCTAAAAGGTAGGATGGGCCAGCCTATGTAGGGTAAGGCTTTTTAACTCTATCGCATGGGCAGTGTGCTGACATTGCATACTTTTTCAGCACCTCTTCAGATCGGTATATAAAGTAGATTAACCTGACAGCAGATGGCGGTTGGTTGGAGGGGCCTGCAGTAAAGGAGAGCAGTTTGGAGAATGCCACGGCATGCAGACGCACACTGTGGAGCTGAGCACTCAGTCGGTGATGTGGCTCACTCACCAGGGGAAGGACAGAGGTGAGATAGGGCAAGAGTCAGTTGCTGAAGTCACTACTCCAAAGGGCTAGATAGAAAGGTAGGGAGGAGGAATGGAGAGCCAGAAAAGGAGCCAAAATGATTAGCTATTTTACTAGAGCAGGTCTGTCCTAAAGGCTTGGCTGCCATTCTCTAGCTGGGACCTTGACGAATCACTTCCTCCCTAGTGCCTCAGTTTTCTTGTCTTCAAAATGAGGATGATGCTACCTATCCTGAAGGGTTATTGTGAGGATTAAATGAGATGATGGGGTGAAAGTGATTTGTAAACTAGGAAGTCTATCATGCAAACCGCATGATAGACTTCGGATAGAGGCAGCAGGTATACCTTTCAGCTTTCTGTCTTGCCAAATGAACATTTGTATCCTCTCACCTGGTGTCCAAGATGAAGGTGCTGGTTCAGTGAGATAACTCTTATCCAGCCTAATCTCAAGACTTCCTTTCATGCTCTCTCTCTCACACCTAGCAATAAATGCCAAAAGCCTAATAGCCCCCCAAACCTTGATCGTTTCAAGAGGATCCTTTCAAAGGAAACCTGCCTTTCCTCTCTCCTTGAGGAGAAACCAAAGCAGTCCAGTGAATAAAATTCATATTTTTCTCCCAGGCTGATTGAAATGGCTGGCCGTGGGAGCCCAGGGCCTTTTTGTAGGACTGTTTTATTTATTTATTTTATTATTATTATTATTTTTTTTTTGAGACAGAGTCTCGCTCCGTCACCCAGGCTGGAGTGCAGTGGCACAATCTTGGCTCACTGCAACCTCTGCCTCCTGGGTTCGAGCCATTCCGGATTCGAGCCATTCTCCTGCCTCAGCCTCCCCAGTAGCTAGGACTACAGGTGCCTGCCACCATGCCTGGCTAATTTTTGTATTTTTAGTAGAGTTGGGGTTTCACCATGTTGGCCAGGCTGGTCTTGAACTCCTGACCTCAGGTGATCCACCTGCCTCAGCCTCCCAAAGTGCTGGGATTACAGGAGTGAGCCACCGTGCCTAGCCTGGACTTTTCCAATTAGGCTGTGCCGGGGCTGCTGTGAAACTGTTTTCCTGTGAAGACAGTGATTTTCTCCTTAGGGTGGCTGCTGTCACACGGCACTGCCCCTTTCCTTATAATACTTGATCACAGACACAGTAACATCTGTGCCTAGATCACAAGCCATACTTACTCAGGACGATTTTCCTCTTAAAGCACCAGGGAGGGAGAGGCCCTTGTCAGAATTCTCACGACTGAATCATTGCCATGAGCTAATGTCTCCAGTTTTATGAGTCAGTGCTGGCCCAGGCATCGGTTTTGTAGGTTTAAAAAAAAAAAAAAAAAGACATGGGTTAGACTCCCCTAATATCAGTAGCTTGTTCAGGGGGAAAGCAAACTGAGCTATCATCAGACAGCAGCATTTTAGAAATCAGATGTTTGGTTCCTCTCCTCTGTAAGGATATAGAATAAGTGGAAGAATTTCAGAATTAAAAATAATAGTAATAAAAGCTCTCTGATTTGTGCGTGTGTTTTGTTTTGCTTCTGGCAAAGAAGGTAAAATGGTGTGTTTGGGAGATGAATGTTCTTGTAATAGGCAAGATGTAGCTTGCAGGAGAACATCTGCAACAAACCCTTATTCTGCATGAGATACAGCCAAGACCTGTTTGTGTGTTAGTGCCTGTTCAAACATGTCTAATTTTTAAAATGATTTATAAAGTTTTGGAGGAATCACCTTAAAATGTTATTTCCTCATATAATAGGCATATACATTCACAGCGTGGTGGCTAAACTATACACCCTGTTGGAGTTTGAGCAGTGCCAATATTGAGCTAAGTGATATAAAAATGGTTATCAATATGAAGTAGATAGAAAAGTATTAGGAATATCTGCAATCATTCGTGAGTCTACCGATTAATCCAGCAGCAGGCAAAAATAAATTATGCTTTCCACGTTTTTAAATGTTCTTGGTAGAGAATTTATGCAAAGAATAAAAGGGAAATACATGGATATTTGCTTCAAAATACCCAGAGGTATTGCTCAAAACTTGATTAAAAATAAAATAACAGAATTGGGGATGGACGTCAATAAGAAAGAATAAGCTTAAATAATATCAGTATCTATTAAACAACATGATTTTTAATTGTGTCTTCTCTATTCTTTCCCTTTATCATATATTTACACATAATAGGAAATGCCTTTTAGGAAAGATAGTGGAAGCCTTCTCTTTTGTTTTTAACAGCCATTGCTTATGTAGTGCTAATGCATCTTCTTAAATCCACGTGCTTTAAAAAAAATAATATTTTATGGTAGTCATGTACTTCTCAACACATCATTTGGAAAGAAAGTTAGGGGTATGCGCTGTAATTTGTACCCAGTATTCAGAGTTTACAAGAAAGATTGCTCTTCCCTGCAGCCTCTCCACCCTTCCCCCTCCTTCTCTCTCTCCCCCACTTGCTTCTTGCTCCTTGTTTCTTTCTCTGAAGAAGGACGTGAGCTACATGGAGGACAGGGCAGAAAAGGGAAGAAAAAAATAGGAAATGGTGAGAAAAATATTAAGGACATTTAAAACTTAAAGTTTTATGGTTCTTTTTAGCTAAATGAATCTTTTGAGCTTCCATGGGGGAAATTAGTGTAATTAAAATTTTCTAGTTCATAAAACCAAATCTTTACATACTGACTGAGCCCACTGTAAGTATTGACTAAGGCAAGGACAAATGTTATCCTCTGACTTGGAAAATTCAAAAGAAGCATTTTGTCTCTTAAATGAAGGTAGGTTGATTATATTTGATGAGCTTCTTGGAAAAGGCATTGATTAGTCCACATCCAGAAATTTAACTTCCCTTTTGCTTATACTACTTGAAAAATGATTTTATGGACACCGTGATGTAAACAATGAGTCTCTCTAGGAAGGGATACTGTGTATATTTTTTGTTTTATTTTTAACTTTCTGGCATTCCAAGACACATACAGTTTTTATTTGAGGGAAATATTCGTAAAATAGAATACGAAGGAACTATTCCAAAGTCAGGAGTTGAGTGAAATATCCCAATTAAAAAACTGAAAGGATTGTTTATCAGTAAGTTGCAGGATATGTGTAGAGCTCCACAACATGAAAATTCAGTATTAATAATTTTGGCTTGAAATATTTTCAGTTCTATCTCCTTATAAGCTTCAGAATTACTAACCACCGAAACTGGTGTAACTAAGAGGACGCATAGCATGAGTCTCATAGGATCTCTCCAACTGGTGCCTTGTAATTTTGTATGGTATGCATGTGCACACATGTCCCAAGATGCGTTTGTTGAGGCTTAGTTGAAGGCCCCATACTATGAGGTCTCAAATGTTAGAGTTCACTGAACAGCACCTTTAGTTTCAACATGCATTTTCTAAGCACTGGAAGGTACACATTGAAGAAAAACAGATATAACTCCACAGTTGACTAGCTGGGTTGACTAGGCAAGTGACCTGATGCTTCTGAGACTCAATTTTCTCTTCTGTGAGGTGAAAATAATCTCCATTGAAGGCTTTTGAAAGTTCAATATATGAAGCCCATAATATAGTTCCTGCCACTGAGCAGGGCCTTGATAAATTGTGCTAGTTATTATTAAGGCTAGACACTATTGGGTAATTATATTAATAGATAAGTTAGCCCTTTCTCAAAGAGCTCACAGTCAAGAAGGGCAAACTGATAAACCTAGTTGTAATTTAGTGTGACAGGTGCTATAATCTGTCTGGAATGTTCCCCTAGCCACCACCACCTCTGTCTGCCATGCAGACTCCTACTTATTCTTTAAAACTCATTGGAAGCATTAATTGTAGATGAACACTTGCCTTGTGATACCCACCCCCCACCAGCTCCCCCTTACAGACACGTACTCAGGAGTTCAGAGGCAGTAGCTTTGACCTTTCATCTCTGGGTCTTAGTGCTGGCACATATAATGGGTCTTAACAAATATTTTTCAGGTAATACCTATTACCTAAACAAAGTGGAATGTAGTACAGAAAACTGATTTTGCTAGTTTCACAGAAAACTTTGCATTTAAAGTTGGGCCTTCAAGGATAAGCAAAGTCTTGGGAAAAATAAGAATAGATATTCCAGGTAGAGGGACCAGGGTAAGTAAAGGCATGGAGACATGAATGTGTTATGTAAGTCTAGGAGTGGGAAGCAGTTTGGTATACCTGGACCATAGAAAGTTTGCAGGAACAGGAGTAGCAGGAAATGAGGCTAGAGAGAAGTCCACAGAGCCAGATTGAGATGGGCTGTGAATACCACCCTAAGGAGTTTGGCTCTAGTCCTGCAAGTGTTAAGAAAACATGGAAGGGCTGTGAGTTGGGAGATGACATCATGTTCTGTTGGAGGAAGCTAACTGGCAGCAGTTTGGAGGTAGGGAGACTAAAAGCAGGGAGGCTAATTGGAAAGGTGTTGCAATATTTTAGATAAGAGATATGGGGGCCAGGTGCAATGGCTCATGCCTGTAATCCCAGCACTTTGGGAGGCCGAGGCTGGTGGATCACCTGAGGTCTGGAGTTCAAGACCAGCCTGGCTGATATGATGAAACCCTTTCTCTACTAAAAATACAAAAATTAGCTGGGCTAGGTGGTGGGCGCCTGTAGTCCCAGCTACTCGGGAAGCCGAGGCAGGATAATTGCTTGAACCCAGGAGGCAGAGGTTGCAGTGAGCTGAGATCGTGCCGCTGTGCTCCAGCCTGGGCAACAGAGCGAGACTCCATCTCAAAACAAGCAAAAAAAAAAAAAAGAGAGAGAGATACGGAGATATGGGCAGGCAAAGGGGAAAGGAGGTGAGTGTGGGGTTTGGGGTGGTTTTGCAGTATATATTTTAGAGATAGAAACCTATTTTCCGTAATTGGGATTATTTTGACTCCTACCATTGTAACATTTTCTAGACATTGGTTTTAGAGGTACTTCTTATAAGGACTGTACCAAGAAACTAGTTGAATGATATATTCCTCAAGGTTTGGGGCCTTTTTGGTGCCATGCTTTGCTTTCTTTAGTTAGGATTAGTCTTGGCTATTTATCCTTCTGCTTTGACTATGAGAAGAACCACATACCTGTAATTGGTCAGACAAAAGAACATTTTATATTCTCTATGTCTCTGCCTTGCACTAGAAATGTTTGGGTAAAAAATTTATTGGTTTAAGAAGGAAGAAATGTGCCCAAATAGCCAATGATTGGCTTGATGGCTTTGGCTAGGCCGTTTTGCTGCTTTGAGCTTCAGTTTTCTTATCTGTAAAAGAAACATATTGAGCTAAATCTTGATATCTTTTTCCTATTTCTTAACGTGGTATGGAAGTTAACACTTTCGTCCTTTTACTGAATGAAAATAATCTAATAGTAGCTTAAAGAAAACGGAAGCATCTTTCACTTCAGTGTTTTGAGCGTAATGAAATGAATGCTATTATTATAAATAGCAGTACCATTTTACAAAATCAGAAGCTAGGAAAATGTATTTGGCCTTTGACTCGTGGTTGGTCCTCAGTGAATGTTCAGTGAATAGAATTATAAAATCTCTTTGTTCTTTTTTGCTGAGTACTAAAGACCATAAATTTATTACAGATGATATTTACTGATAATAAAGTACTTTTATTTTTACTGGTACCCAAAGAAGGATTCAAAAATCTTGGCAGATACCATATTACCAAATCTAACTATAGGAGCTGGACCAAGAGAGAAGACTCTGTAAATCACCTGTCTACAGACCCAAGACCCAACCATCTTAAAAGAGGATTCAGGAAATCATTTAAATGTTTCTTCCTGCTTTCATGCCTTGAACATGCTGTTTCTGAGCAATACACTACAGAGATTGCCCTAATTGACACTAAATATGAGTTTTAAGCATTTATTCTGAAAACTAGACCCATGTAAACAAGTTAGCATCCAACTATCAATTCTTAGAAAATCTACTTTCCCTATATGTCTACTGGATTATTTGTCTCAGATCTTAGGGGAGGAAGTGGAAGAGCTGGAGAACAGCACGTGTTATTTTAGAGTTATAACTGGAAGTATTTGGAATGGCTTCTATGAAAGTTTATTTCACTGAAAATGTGGAAACAGCTTTGGAACTAAGGAATGGGCAGAGACTAGATTTGGAGGAGCAGGGTACTAAAAGCCTAGAGTCTGGCGAGGACTTAGAAGAGAAAAAGATTCTGGAAAGTTTGGAGCTCTTAAAGAAAAATAAAAATTTACTTCACTTCTAACTATTTGGTTGGTGCAAAAATAATTGCAGGTTTTGCCATTACTTTTTCACCAACTAACAAATTCAGTTTCAAAGATTTCTATACTGCTGACAGTTTTTTTCAAATATTAGCCCTTTTAATTATTTCATTACCAGACCAATATAACCTTAATTTTTTTCAACCAAGATAAACAATTTTTCTGCAGTAAATTTGTATTTATGTTCCAGACACATCTCATCTGATATACAATATTTTCTTAAATTGTATAAAGAGAAATTATTGTACATCGGATGAGCTGTGTCTGGGATTAAGTATTAGCTCCACTGTAACCTTCATTTTCTTGAACACTGGAAAGAAAGGCCAACGGTCATTTAGTAGGGTTGGAATAGGATGGGGAATGGGGCTTTGGTGAGAGAGCATTGGACTGTTGGTTATAGAAACACAGTTCAAGTATATCTGGAGTTCAGATTTGAGATCTGACTAGGAGAATCTGTCTAGTGAAAAAAGCTAGACCTTTACAAATTGTAGTCAGGAGCTACCGTTGTAACAGTCTGCCAGAGTTCCTGATACAAAAGATTGATAATTTGTGTGTTTGAAAAACAAAACAAAACAAAACACATCTACCTTCAATTTGACTTACTGCAACTCTGATTTTCATGGCAAGGGATAGAGACAAAATTCATTCTATAAAATTTGAAAAAAAATGTATATCCTGAAACCATTGGAAATTGGATATCAGCATTAATTTTATTGGACTGAAAAACCTCCAGTTTACTAACAGGAGTTGAAAGTCATCACTTTTCCTTTCATCATAGTGAGTTTTCATCACAAACCACAATGATATGCTTATCTCAAATATATTCATTTACTTGTTTTACACAAGTATCATTTCTGTTTACATAACACGCAATTTTGTTGCTGCTGCTGTTGTTTTATTATGCCTGTCACTTGAGTAAAAGATGGATAAGGTAAGGTCTCCATTTAGAAATGTATAGTCTCTTGGCAGGGAAGACAAATAAAGGCCCATAATTATCATAACAGAAAAATTAATATGTGACGCAAGAAAAAATAAAGATACTATTAAATTTCTTTGGGACTTTGAAGGAGGAAGGGAGCATATAGACACTTAGTAAATATTGTTTGAAGAAGGGAATGAATATGTAAAGGGAGAATAAACCTCACCTATAAGGTCTAGGGCAATTTAAACACAAAGGAGTTGTGTAGGATTATTTAAGGTTTGCAAATGTGTAAAATTATCTCACTACAATACTAGGGAAATGGAAAAACACCAAAGTTCCTCTACCAGCTTTTGCTGGATGAGATGGGCCAGAAGTAGTAACTGCGAGGGCAGCCTGTATTTCTGTTGTTGGTGTGGTTTATCTTCCCAGTGCCAATGCTGCCAACTTCCCCTCAAATAAAAACCCATAGGGGTTCAGAGTCTCATGAGGTGAGAACAGACTTGCACTATCTGTGACAACAACAAAAGCACTTATAATTTTGATGTGGAGGATCCTAATTGAAATCTGCTGTTTGAGGTTCCTTGGGTCCTGCATTCTTTCTTAAATATTTCTACTTGAGTTGGTTATTGATCCTTATTTATTTTCCGTCTAATCGATTGACTTTATAAATCTATTGCAGAGGGCAGATTAAATAGGCAGACAGTTTTCTAATTTGCTGTGAGGGTATTTCTTAATTTACTTTTTAAATACTGATTTTATTTATACATTTATATGCACAGGTCATTTTCATTTCCATGATATCATTCGAGCTTCACAACCACACTCCAAGTTCAGTGTTGTTATCTTCATCTTCATGTTGAGGATGAGGACACGGAAGCTCACAGGTAAAGTGACAAACCCCAGTCTTTAGGCTACTAGATGGTGGAGTTCAGACTCTGATTTTCTGGCCAGGTGCTCATTTGATGATAGCAGCATGAAGCTCTTCCCATTCTGCACCGTTTGAGCTCCTACCCTTCCTAATCTTCTTTCTCTAATACGAGAACACTGTAATTAAAGTGGTATTTGGCTGATAAGTAGTAGGTTTTGATTTATAACACATAGAGCTGGCAGTGTAATTTTTGTCCCAGAACTTGATGTGCCATGAAAGCTATTATGCTCTTTTTTTTTTTTTTTTTTTTTTTTTTTTTGAGACGGAGTCTCACTCTGTCACCCAGGCTGGAGGGCGGTGGCGTGATATCTCGGCTCACTGCAACCTCCGCCTCCAGGGTTCAAGTGATTCTCCTGCCCCAACCTCCTGAGTAGCTGGGATTATAGGCATGCACCACCACTCCCGCCTAATTTTTGTATTTGTAGTAGAGACAGGGTTTCACCATGTTGGTCAGGCTGGTCTTGAACGCCTGACCTCATCATCCACCTGCCTTGGCCTCCCAAAGTGCTAGGATTACAGGCATGATCCACCACGCCCAGCACTATAATGCTTTTAATAGAGGGAAATGAATCTTCAGATGATGTCAGCATCCTAATTTTTTTTCTCCTCTTGGGGTTTTTCTAAATGACTGGCAAATGGAAAACCTGAAAGTTACCTGAGACTTCTCTGCATCGGGCTTGTTGGGGTATACTGTTCTTCTTATAATCAACCCCAGGGACTGACAGATGGACAGTTGTTAGAGACAAAGTCCTCAATCCAAGGAGGTCACTCAGGGCCAACTTTCCTTAGTATATATCGTTCATTACATTAGTTGGTTATATTACCTGTAGTACAACTCAAGTACAATAGCTGCAGGAGGAGACAGCTCGCCAGCTAAACCTAAAAGTTACAACAGTGACATAGCAAGCAAGGGTAAAATAAAAGGCTCTTGACTTATTCCTTCATTTTTTAATAAAGCATGAAGTTTGACATAAATTTTAACCTCTGGCCGCATAGTCAGTTATGCTTATTATCTTGTGTGTATTATTATGCTGTCAGGAGTTGGCCACTGATGACCAACTCCAAAATTTAGATGATAGAGATAAAACTGGTAGTTAATATATGTTGAGTACCAGGTCAAAAACTCCATAAGACACTTTACACTGTGGGAACTGGTTCATGTAACAGTTCTCCAGGATGGAGGATTAATATTCCCATTTCACAGATGCAGAGTTATGTGTCTTTCTAGACCTGACAACACCTGTCACCTACCAAATGAATAGAGAGCTATGCTATAAGTCTAGGGATGAGTGATCCTTGGATGCAGAGCACTTCCTACTAGTATTCTGACTTTTACTATTATGCGTCTGTTACTATTTGACAGCTCTCATTTTTGCCTGTCCAAATTTAAGCACGTTTAATGAGATAAAGAGAGCAGAGGCAAAGGTGAGACTGGATCTTCCCTTGTATCTTATTGATAGAGAGTAAATCGAATCCAATTTGCTAAGGCCTTCTCTCTCCTTTCATGTTTCCTCACTTTGCCTCCACCACCATGAGGTTGAAAAATGAGTTTCCATTTAGTTTAAGCCTTTTGTTATTCATTACTTAATTTTGCTTCTATTTCCCTCTTCTATCTATGACAAATTCAGCACAGTGAGGGGAAGAAACAGTGATGATAGTGATGGCCTCAAGAAGAAAACAGGGTGACTGAGTAGGAGCAAATAAGTATCTCAGACCAGCATATAAACAAGTTCATTTGTAGTGTAAAAGAACATTATGCCTTTGGCTTGAAAACTGCCTTTCCAATTTATGAGCATGTTTAGGATTATGTAAGTACAGTGTCTTCATAACAAGCCCAACACCTCTTACCCAAAGAATGCAAAACGCTTTGCAAACAGTAGAACTCACAATTCCCCTGTGACCTGGGGACTTATATGTATTTTCATGCATTATTTTTTATAGATTGAGAAGTGAACTTGAGACCTACCAATCTGTCCACGTTGACAAAATAGATCCAATACTAAACTCTTAATTCCAGCTTCCTGTCTTCTTAGAAGCAGATCACACTGAAAGAGGAATCTTATGTCAAGGGTAGCAATTTTCTCTCTCTCTTTTTTTTTTTTTTAAAGAAAACACAAATTACATAGTAGATATATTTGCATAAAGGTAGATCTTGAAAACATTTAATTAAGACAGCGGTATCTATTTCCAGGAAAAAATAGAAAAGCCAGATCTCTACTCCACTGAATTTAAAAATTAGAGCAGCATTTTGCAGTTAAAAACGATTATTATTTTGCATTCTGCCATTTGTCTTTACATGAAGAATGCCTGAATTCAGAATGGAAAATATTTTCTTTTGAAAAGTGCATAGGAAAGAATTGTTATAAAGCAAATGTGTTCAGAATGTATGTAGATATTAATTATAAGTAAAAGTAAAAGAAGTAGGATGTATGTGAGCGTATGTGCATTTGTGTTGCAGATAAACCGATTTTGAAGGTTGAGAGACATGACGATACTTGTTAGGTATGAGGGAGACAAGACCAGAGGCGCCGGGAAGCTTGACTACCGTGGAGAGCTCAGTTGAGACTTGTCTTTGCCCATGGCCTGCAGCGCTTATGTAGAAATTGGGGATGCTTTTACTCGTTCCAAAGCCTAACCTGATCAGACCTGCCCAGGTTGGAGTTTGGGAAAAAAAGTGTTTGAAGTAAACAGGAAAATTACTCACCTTAATGATGTGTCTTACTGGCACTGACAGTGCTTTGATAGTCTGACAGTCTTTTTTTTTAAGTTTTGGGAGGAAAGACAGGGGAAATTATTAATATGATTTTTGGAAAACATAGATTCTTATGCTTATGCACTTGCTAATAAGGTGGAAAAAGTGGGCTTTGCACCATTTACAGTTATGGACTCGCGTTTCCTCCTAATAAAGTCTATATCAGGAATTTTCCATTAGGAATTTCTACCAAAAAGAAAAGAAAATACGTTACTGTTTATTTTGTGTACTTGTGCCATGTATCTCCACAATTGCGCTGTGGCTGTTCACACAGAACAGATTCTTCCTAGCACTTGGGCTGTGGGTTCCCAAACTTGCAGGTCGCTTTCATCTGTCTGGTGATTAAGACCATAAACCAAGTGGTGTGATGAGAGAGTTTGGTGCTCTAGACAGAGCAAGCACACCCTTTTTTCATAAGAAGCACAATTCTTAAATCTCTGCACCTAAATCTTTCCTTAGGATGGGTCAGTTTTTTGGAAAAAGCAATTATATATGTTTTGATATTTTGTTGAAACATAATAGATGTTGATTTCATATGGTTTTTTTCCTTTTTTTTTTTTTGTCTTTGCTAAATGTCAAACGGTACATTTTTTCTAAGCTTGAAGACAGAGAACTGGGTCATATGTCTCTAAACATTTAAATCTTCACTGAGATAGGGAAGAGTGACCTGATCTTATGGATCGTCCGAGTCCTGTGAGACAAAAATGCTGGGTTTTTCATGCAGTCCCTAATTCACTTTGACCTTGGTGGAGGGAGTCAAATCTCCTAGTTTGAGATCCCACACATATAAAGCTCTAGAGTTTCAGGATTAAAATCTTCTTAACTTTATATTCTCAGATTCTGTATAGCTTATTAAACTAGATGGAGGGTCTAGTTATACTGTCTAGAAGGACATAGATGAATAGTTATATTTTATAAAAATTAAACTCCTAATGATAAAAGGCTAAATGGGGAGAGAATAAATTATAGTTTATGAAGGCATGTAAAACAAAAGATGTCTGCCAATTTCCTCTCTTTGGTTCTCTGACTGGGAATAACTGTATCGTTTTTACTTTTTTTGTCCTAACTGTGTCCTAATTGTAAATGAATCTGGTGCCCAGTAAAATCAAATGAATAGAAATACTTACCATGGTGTGTATATACCAGCCTCCTGGTGTCTCAGATATAATTAACATAATGAAAGCATTGCAATACATTCTTACCGAAGGCTCAGTTCCTGTCCTGTCAGCAACTGTTTGCCTACAACTAAAGCAAGTCTGGGTTCAAAGCATTTTAAAATGTTCCAAAAAAGTGGGGAAGGCGGCTGGGTGCAGTGGCTCACACCTGTAATCCCAGCACTTTGGGAGACCGAGGCAGGTGGTCAGGAGTTCGAGAACAGCCTGGCCAACATAGTGAAACCCCATCTCTACTAAAAATACAAAAAATTAACTGGCCATGGTGGCAGGTGCCTGTAATCCCAGCTACTTAGGAGGCTGAGGTAGGAGAATCACTTGAACTCAGGAGGCAGAGGTTGCAGTGAGCCAAGATCGTGCCATTGCACTCCAGCCTGGGCCACAGTGCGAGACTCCATCTCAAAATAATAATAATAATAATAATAATAATAATAATAATAATAATAATAATAATAATGTAAAGTGGGGAAGGCAAGATTTGGCTCACGCGTCAGACTTCCTATCAATGGGTCTATCTTTGAAGCAAGTGCTCAGAGTGTCTATTCCTTTGATCACACGGGTAGTGGTTTATTTCCATGAGTATTTGCTGTTTTCAGGAGATTTCACTTTGGGTTTTGTCCATATTCCACATTCAGGAAACTGTTAATTTTGGAAGAAGAGATTGTTTTTACTTGGTTCTTCTAAGGAAATTAATCAAATGTACACGTGCAACTTAAAATCAAAGAATCCCTGTTTTAAGAGGAGTTTTTCTTATTTCAACTGGCAAACAATATGAAACTGAATGTACCCAAAGTGTTGGAGGTTTACAAACATGATCAGCACTTGTTAATGACAATTTTCTCCTAAGAAAATGGAATGCTACCTTCTAGCATGGGGACTTGTAGGCAGTATTTCTGTGTTTGCTAGCCTTGGAAAGTATAACTCTTATAGGGTTGGGCAAAGCAAAGCGTTTTTGCTTTGTAACCTGTAGAAATGATTACGTAGTGTAATTTGGTTATTTAATATAATTTGTTAATAATTCTCATTCATCTACCAGAAACTTAAAACTTGGTACTATCCTGCTAATTTGGTTTTGGTAATGTCTCTTTTCCCTAATTTTAATATCCCTGTTCCTTGGCAAACCCATCCATACACCTACCTGTTGTTCAACTGGTGTGATAAGCCCCATCAGTGCTCCTAGACATTTTCTTTTCCTACTCTTCCTCTTCAGTGTCAGGCAGAAGGATCTTTCTAACACTCGCCTCAGTTCATGGATTGCTGATGGTCCATTGTTGGTGACTGCATGATGACCTGTAAGCATGTCACACTCTGACCAAAGTGTTTGTATTTGGCCCAATTCCCCACGGCTAATAATAATCCCTTTCTGTTTGGAAAATGTCTTATATTTTTCAAAACACACACATATATGTATTATCACATTTAATCCTCATAACAAACCTGTCACATTGGCAGGTGTTATTCTCACTTCTCACATGCAAAGTTATGTAACCTTTCTTGAGATTATACCTCAGTTTGGAAAGTAGGGGAATGAGCCTTCTGCCTCTGTGAGTACCGTGTTCTTCACGGTGTACGATGCTTCCAAATATATACTCTTCCCAACAGGCAGAGGTGGTGTTTCGCAGTCAACAAAAGATGATTTTTGTTTTGTTTTCCCTTCTGCATGCCCTGTCTCCTGCTACTTCCTCTGTTCAAAACATTTTTTTTTTCTCTCTCTCTCTCTCCTCTCACTGCTGCCTACCAAAATTATGTCACATAATTTGGCTATTTAATGTAATTTGGTTAATAATCCTCATCTATCTACCAGAAACTTAAGAGTTGTTATCATCCTGGCAATCTACCTTGAAGATTGGCAAAATGTTAGTTAACTATTGAATATAGATGATGAGCATATGGAATTAAATTTGAAATTTCCAATTTCAGGTTCCAAAAATCCAATTTCAAATTAAGAAATTTTAAATTTAAATCTTAAATTAAGAAATTTAAGAAACTTCTTAAAATTTTTACTTATCCTTTCTAGCTAGAGTTGCCAGATTTAGCAAATAAAAACCAAGGATGTCAGTTAAATGTGAATTTCAAATAAACCATGAAAAAATGTTTAGCATAAGGGAATCCCATGCAATAGTTATTACTTTTGTTTTATTTTGTTTTGTTTTCTTGAGACAGAGTCTCCTTCTGTCACCCAGGCTGAAGTGCAGTGGTGCAATCATGGCTCACTGTGGCCACTACCTCTCAGGCTCAATCAGTCCTCCCACTTCAGCTTCCTGATGGCTAATTTATGTATTTTTTTTTTCTTTAGGGACAGGGGTTTTGCCATGTTTCCCAGGCTGGTCTGGAACTCCTGGGCTTAAGCAATCCTCCCGTCTTGGCCTCCCAAAGTGCTGGGACTACATGCATGAGCCATTGTGCCTGGCCAATATTCCTTACTTATGCTGAAAAATTTCATTGCTTTTACAATTAAATTTTAATTAGGCTGGAGATTATCTGGTAACCCCATTCCCAGCTCAATCACCAACTCTCTAAGCAGAAGGAAATTTTTCATCTCTCTTGCCATAGTGAGGTCAGGATTAGTGAGGTGACAAGATTAAATATGAGCTAAGCTATGACACTTGTTTTCCACAGTTTCCTGAGCCCCCCTCCCTCCTTTTCTCTTTCTTCCTTCTTACCTCCCTTCCTCTCTCCTATTTATTCTTCCTACCTTTTTGTTGTTGCTGTGAGTGTCTTCACCTGTTTCCTTCCTTCCTTTATATTCCTGGTAATGTTTCAAATTTATTGGCATGGGCAGCTTCTCATAATAGGTTTGTTTTTATTATTTTTCTTGTTATTACTAAAACATGTTAATAGATAGATTCTAACTCTATGGCACAACTAAGACAGTTGTGTTACATTCTTTTCCGTGTTGGGGCTAATTAGAATATTAAGTTTACAAAGAATGCAGAAAAAGCGAACAAGATGAAGCACGTTTCCGTCTTAAACAAATGAACTCTAAAGAAATAGGAAGTAACATGATGACAAGCTTTTAAGAGATGTTGCAAGTAGAGGAAAGGGTTTGTTTAAATCTCTCCCCAAGCACCCCCTACACCTACCCCCAGCCAACACAGATGCTTATGTATTAGTGGCCCACACGGTACCTAATTATCAACGATGGAAATAGATGTCTCAAAATTGTTTCAATTATCCAGGGGTTCCCTTAAAAAAGGAGAGGAGAGCAAAAGTTTAAAACTTGGCTTGAAGGTATTAAAGAGCAAATCTGATCTAATTGATTAAGTTACACCTTTCCTTTTTGGCAGTATCTTAATACCCCTAGGATCTCAGTATTTCAGCATATTGGTTTCCTGTACTTATCTAGAATAAAGAAACTCAGCAATTTGCCTATAGGATAGTAGAGCCTTGAACTAAATTTATCTCATTATTGTTCACTTGAAACCTTGGGGACAGCTGTAGTTGAAGGGACCTTAAAGTCCCTTAAACTTCTCAGCATTTGCAGGTTCACTTTTAAGGTGCACTAAAAAGCAACTAGTATCCTAAGCTAAAAAAAAAAAAAAAAAAAAAAAAGAAAAAGAAAAAGTAGATTTAAGTATACCAATGAGCATGGACTCTGTTTATGTTTTGAAATCCATTTGCTACTCATTTTCCTCGTTTTTAAATCCTATTATATTTACACCTGGGCATCCATTTCTCTGAAGAGAGAAGAATGAAGGATCTAAAATGTCAGAATAACAGTGATTCTCAACAATTATTGTCGGCACTCTTTCATGAAGAGATTTAAAGCTCTTTGCTTATCTGACTTAATTCCTGCCGGAAGGACAGTACAGAGGGATTACGGTTTAAATATTGCAGTTAGAAGCACAAAGTGGAACCTGGACTTCCCCGACCTTGGAAGAAAAATAGTAAAGTGTGGTTCCTATAAAATATTATAGGAACTTCAGACATAGAATGGAGCATCCATTTATAGTGATTTAAGTTCCAGTATTGAAACAGGCCTTCTATTTTTATTTCAATCTCCCTCCCCTTTATTTTCTTTGTTTTGTGATTCACCCCAAAACGTATCTTCTTATACGGTAATATTAACTAGTACCTTAGTCAAAGATTGATCTCCAGAAGTTTGTTTAGGAAATTCCTTGTGTTACTTGTCTAGCAGTTAAACTGCCTAGTGGAAACAACAGCTCAGCTAAATAGTGTGATGCAATCGTGATAGCTGCTGTTCATCAGGCATTTACCCTGGACCAAGCACTGTACCCTGAATATCATGTGGCTTTTTCTCCACACTCAGGACAAGCTTGCAGTGGGCAGTAGTATTATCTCAGCCTTGAAATGAGTCACACAAGGTTTTGCAGCAGAGAGAACTGTCCTCTTTTTCAGCCCTGTACAGGGGAGGAGCAAGGGTGCTGCAGTGAGGGGATCTGGAGTTGAATCTGGCTTAGTAGCTGTGAAAGATTGGGCAACTTATTTCACGTTTAGGAGTTTCAATTTACTTAACTGGCAAATGGAATAACAATGTCTACTTCACAGCTTACTGCGATGATCATATAAGACATAAAGAACTTAGCGAAGTGCCTAGACTGTAGATCACAGTACGTGTTAGCTTTTTGTGTTTTATACATCAAGCGTAAGTCTTATTTTTTACATCTGTCATATTACCCTTTCCCCAGTATTCAGAGTATTTGAGTGTATACGTACGGGTGGTAAGTGTTGTTTTTCATTGAAAGTGCAGATCCTAACTTTTTTTTTCATGCAGTAATCCTCAAAAAGGTAGACGTCTACCATAATTCAGTCTATTGCTCCAATTGGAGGATGTACAGTTGTTTTAGGAGTTAAGGAATTGAGTTTTTTCTTTTTTGCTTTTCGTCTTTTTTTTTTTTTTTTTTTTTTTTTTTTACAAATTCTTTTATTCTTTTCTTCTCCAAATTCAGGCAAACAGTATTAAACACTTTGTTTTAAAAATAGATTGTGTACGTGTGTGTGTGTGTTTATGAGAGAGACAGAGAGAGAGATGGGAGAGGGTGAGAATACACAGTAAGGACTTGGAACATATTCTTTGTGTGAAATGTTAAAAAATTGTTCACGGTGGCTGGGTGCGGTGGCTCACGCCTATAATTCCAGCACTTTGGGAGGCTGAGGTGGGTGGATCACGAGGTCAGAAGTTCAAGACCAGCCTGGCTAAGATGGTGAAACCCGTGTCTACTAAAAATACAAAAATTAGCCGGGTGTAGTGGCAGGCACCTGTAATCCCAGCTACTCGGGAGCCTGAGGCAGAGAATTGCTTGAACCCGGGAGGCAGAGGTTGCAGTGAGCCGAGATCATGCCACTGCACTCCAGCCTGGGCGACAGAGCAAGACTCAGTCTCCAAAAAAAAAAAAAAAAAAAAACAACTATTCAAGGTTTCCCAATAACCTTCTTGGGCTTTGAAGCTAGAGAGCGCTTTGGAATTCATGCTCCATTATATGTGTGTCTTAATGCTATATACTGTTGTAGGTCAGCAATATTTATATACATAAGTTTATATATATGTATATATAGATCTTTTGTAAATAGAGGGTGGATACTTTGTCCCCACCTTTTCCCATCCTAGCATGCAGTATAATGATTTGAACACGGCAAGGACACCACAAAGGCACAGGAAGACATCCTGCAATGTACATATAGTATGTGCCTTATGTACAGTAAGGGCCCTGAATTTAAAGTTAAAACCTAGATTGGAGAATTCTAACTTTGTGTTATCGTGAGCAAAGTAGTTTACTTAAGTCTCTGATTCTGATTCTTCCTTGACAGAAAGGGAATACAGGTGACTTTCTTATAGAGTTAGCTGAGGATTAAATTAATAGCTGTATGAAAATGCTCTATAAACTGTTAAGGACTATACAGATGGGTGAGTGATGGTGTCATTTATGGAAATCAGTAGATGCCATTACTCTCCATACTGTCGATACATAGTCTCCCATTTCTTCTTATCAGACTGGCCTACCTAGTCTTCCGGTAAAAAGAATATCCCGCAGATACAAATTACCATTACTGGAACAGAGGACTCACTTTTAGAGCAGATGTACAAATGAAACTCCAAAACAAGCCACAAATCTACCAGGAAAGAGAACCAATATGTGTGGTATTATATCGCCAAGGATACCTGAGTCATTTCATAGAAATTGAGCTGTGAGTGCCTCCCAGAATGAATGGTTTCTGAACGTGTTTCTGTTAGGAATGATGCAACCTTCAGTAGAGACCAGAGATTTAAGAATTCTATACTGTGAGTCATTTTAAAAGACAATCAAAGAGTATGTGTGGAAGCATAGACAATAACTTTCACCTCTAGCGTCCAGGTGTTGAAGGAGGTCAGCTACCAGGGAAGGACTCCTACGACTTTGGGTGCCACTGCGTGAGGCTTTGTCTTGGACCAGGGGGTAAAACATATCTCAGGCAGGACGCTGAGCTCTAGGTAGGGCTATCGTTTTTGTAGAAGCGTGACTTCTTGATACAACAAAGTAAGAGCTGAGGAAGGAACAGGGAAAACCAGTTTGGGCCCAGAGGGGAAAGAAATGATAGTGTAGTAGCCCGCGGTTTTGCAGGCAGTTGGGAGTAGCAAGCCACCCTTAGGGGGAGGATAATGTTTTCTCTACCTTAAGGGAAAATGATAGCTGACTTGCAGAGCAGTGTGCTGAGACAGACATGCTGCTACTGCAGTGGAAGGGGGTTGCAACGTATTGTCTTCTGTCTGGAGAGAGAGAGAGAGAGAGCGCGTGTATGTCACGTCTGTGTCCATAAAGCTCTCCGATCTGCCCACCAACCCTGTCCCCCACCAATAACATCTTAAAGAGAAGAGGTACAGGAATGTTTTACAGTGAAAAGATTACAGGGCTGGAAATTAGAAGCCACAAGTTCTAATTATGTCTCATTGCTAACTTCTGGTGTGATATTTTCTCTGGGTCTCTCATTTATCGGGGAGTTAAACTAGGTATGTCCAGTAGGGGTGAGGGGTAAGAGTTCTCTGCCCATCCAGGGTTTCTACTAGAAGAATTTTGACTCTACAGATAATTGTGTCTTCCTGTATTGCTGAGGAATGTCCAGGTTTGTTTTATTGAGGGGCAGGGGAGCACATAGATGTCCAGTAGTCAGGAAGGTGGTTTGCAGGCCTTTAATCAGCACTGAGAAGAGGGAAAACTAACTGCAGTTCAGTTCTCAGTTCCACCATTCACTGGAGTGCTGCTTTCAACTTTTGAGCTCCTGTTTCTACACCTCTAACGTATCAGTGTCATCTCACCCTGGCCTTTTTCTCATGATGGTCGTGACTTTAGGAAACATGTAAGAGTAAGCCGACCTTAACACGAGTACCTGCTGTATGACAGTCACTCTGACAAATGCCTCCCAGTGTCTGCTCTGTGAAGGTAACTGTGAATGATATTACTGGTCAGGTGGGGTTGACAGAGGTGGCCCTGGCAGGAGACACTAAATGAAAGGTCATTTTACCCTCCTGGGGAGATAAAGTTGCAAAATTACCGATCAAGTCTGTCAAATCTTTTTAGACTGGGTTTCCAAATCCATACCCTCAGGCAACTAGAACAAGGCTGGCGAGCTCCACCCCCGCCCCTTCCCAAGGTGGGTGCCAACATGCTGCCCTTCAGCAGGGAGGACACAGCTGCCTCTGGGGCCAGTGCCCAGGGCTAGCCACAAAGCTGGCTATGGCCTCCCCAAGAAGACCCAGGACACATTACTCCCTGCAGAGGTTTTGCTGCTTTGTGGCACGGTCTAAGTGTCGCCCCACTCCCACCACCTGCCAGGCCCAGTGGGCTCCTGGCTGGTCCTCACGGTAAGTGAGTAGCAGACAGCCCACCTGCTTGATGTCAACATTTGTGGGCGCGGCCCGGGAAGAATGTGCCGCTCCCAGCCTCCTTGGCTCAGGTAATTAATCCCCGCAACTGACAGGTGGCAACAGGCGGCCCTTTGGCTGGAGCCATCAGGAAAAGTGCTTGGAGGAAGGATTTTTTTAAAAAAATGATCAAGAGCACAATTCGTACATGTCTGGCAGCTAGACGGCTGTGGCAGGAAGGGAGGCAGGGAGAAGTAAATACCTTGCCAAGACGACTGTGGGGCCAATGTTGGGATCTGCGTCTGCCTGATCATTGTGATAGATCCAAAAGGATATGGTGGCCTTGGACTTGTTTGCAAGCTCACTTTGCAGAATTCCCCTTCCTTTGATGTGGATACAGTCTTAAAATGGAATGCCTCATTTGTGAATCAAAAAATCCGGGGCCAAAGTTGGAGCCCCAGAGGAGAAATAATTTGCCCAAGATGATGCAGTAAGTCAGTGGGCAGGTAGAGGACTAGAAGCCAAACTCAATGCCTCTTGGCTTTGTTTCCATGATTCACTGGGCAATATTTACTGCCCCATTATACACATGTAGTTTAGAAATAGAATGACAAAACTGGGGTCCTAGTCAAAGGAAGGATGATCAGTTGCCTCTTGAAAGATATTAGTGTTTTCAATCTAACCACTTAAAGCTGACTTCTCAAGAGAATGCCCCCACCCCAACCCCAGACCACCTGTACCCATTCACCCCCCCTACTTTTTATTTTTGGTAGTTCGGGACACTGACTATCAGAACACATTGACCTGATGTTTATTAGGATGGATATATTAAAAGCCATTGAAATGAACACTTTTCCTTTTTCTTTTTTTTTTTTCTTTGAGACAGAGTCTCGCCTTGTCTCCCAGGCTGGAGTGCAATGGCATGATCTCAGCTCACTGCAACCTCCACCTCCTGGGTTCAAGTGGTTCTCCTTTCTCAGCCTCCCAAGTAGGTGGGATTACAGGCACGCGCCACCACGCCCAGCTAATTTTTGGCACGACCTCAGCTCACTGCAACCTCCGCCTTCTGGATTCAAGCGATTCTCCTTTCTCAGCCTCCCAAGTAGGTGGGATTACAGACATGTGACACCACACCCAGCTAATTTTTGTATTTTTAGTAGAGATGGGGTTTCACTATGTTGTCCAGGCTGGTCTCAAACTCCTGACCTGAGGTGATCTGACTCGGCCTCCTGAAATGCTGGGATTACGGGGGTGAACCACTGCACCCGGCCAACACTTTTTAAACATTACATTTTTTAAAAAATAATATCTTTCTCGATATGAAATTTAAAATGTTTTTCAAGTGAGTTTTTTTGTAGAGGACAAATCCAAAGTATAGTATATAATTAGCCCATATAACTTCATGTGATAATCAGTGTCTTTGAAGCAGACCAAACTGAAGCTGTGTCTCAGCGCTAAGGGTCTTTTTACATTTTACTGAATTGGATCTTTAGAATTAATTTTTCAAACCCTCCTTATCAAAAGTTATTGTTGGACCCTCAAATTTTTATTTTCATGTATCTCATTTCTCTTCCCTAAATCTTGCCTCCCACCAGATTAGCTGCCAGAACTTTATCTGGTTCTCTGGCAACCCTCCATCTGTTTTGTATGATTTTTCATTCCCAGCAGTTCTAATTATTGATTTCATCACCCTCTTACCCTTATAAAAGGACATTTTTAATTTGGCAGTCCCAAGGAATATAGCTTATAAAAAGAAAAAAAATTAAGTCACTGAATGTTTTAGGAGAGCACTAAGTTAAGACAGACTTTGTACTTGATTAACGGAATTGTTCTGAGAGCTTTAGAGCTTGACTTTTTTTTCTTCCTGTCAAGTATTTTGGGTTATTTGAGATAGTACAGACAACACTTTACCTCTTAAGTTTTTTGAACTAGAAGCCATAAATTAGAAGTGCACCTGGCATTTCCTGTTTGTGACTAGTGGCTATCTTTTAGATAACAGTGAAGTCCTCAATGTCCTTGATAGGTTCTTGGTAAACTGCGACCTTAAACAAAATGATGTGCAGGAGATCTTCGAGTAATTGTTTCATACAATGTTATTTTCTTATAACAGTGATGAGAAAAACAAATTGTTTTGTTATATGTGTTTTGCTTAAAGTTACAGTTCCCAAAAATCTATCAAAAATGTTAAATGAGGATTCATTGTATTTGTAAATCTCATTATTTTTCTCTCTTTTTTAAAGAAACTAAATTCAAATTCATTCAGAAAGCCAGGCATAAAGTGAGACTAATATGTTCGTTGGTGTATTTAAGCCTTTCTTAGAGTTTTCTTTTTTTTAAGACCTCAGTATAAGAACATGCCAGACGTGAATTGATCTGGAATTGCAGACTCGGGAGGGCTTTGATGCTACTCTATCTAGTTGCCTTCCTTGAAGAGGGATTGAGAAGAGAGGTAGGTTATAGAATTAATGATTTTTTTTTGTTCCGTGAGAGTTTATTTCATCCATAGTCTCTAAGATTTAGGGCTCAAGATAGCCATGTGATTTGAGATTTGCATGTAGTATGGCATTTCTTCTGGGAGAGGATGAAAGAGCTAAAGTTCTTGTTTTTTGGTTTTGGTGTAGTAGGCAGGGAGGGTTTTGATTCTACCTTTAGGAAAGGGAGGTGTTGAAAAATTACGCAGGTGTGTGCTGATATTACCTGGCACCTCTATGCATTGTGATACTGAATTACCAGTCTGCCAGCCAAAAGCGTGTAATATAGTATGTCTACTGAGGCAGGTGGTAAAGGTAGGGTCCTAGTCACTGGAATTCTTTTTTTTTCCCCCTTCTCTTTGCAACATATTATTAGTAAAGGAGCTGAATTTACTTGTTTTCCAAAATCCCCTGGAGTTGTTTTATTTGAACATGATATTTGGCTTTAAGCAGCTCACCCCTCGATATTCCAAACTTCCTCCCCTCCCTCCCAAAGCAAAACAAAATTTAGGCAAAGCTTACTTATCCATTTTGACAAGGATATTTTATAAAAAGAAGGAGTTTAGTTCATTTGTTAGTTTTCAGGAACTTCTTTTCCATGTCATTGTTGAAATATTTAGTTCATGTGGATGGCCAAAACTGTCATGTCAGTATACAAGGAAATTTGGTTGAAGATCAGTACAATATTAGGCTCATCATTCTTGCCCATGGGCTGGTGCTTCCTGGGAATGAACAAACTATTGAATATATGTACAGTATGTATACAGAAGAACATATATTAAGTTCTACTTTATATATTTATACATATATGATCAGAAATGGCCAGTAGGGTGTGTGTGTGTGTGTGTGTGTGTGTGTGTGTGTCATCAGAAAGATTACCAACACTTTGTTTTCCACATTCTTTTTTTTTTTTTTTTTTTTTTTTGAGACAGTCTCACTCTATTGCCCAGGCTGTAGTGCAATGGTGTGATCTTGACTCACTGCAACCTCTGCCTCCTGGGTTCAAGCAATTCTCCTGCCTCAGACTTCTGAGTAGCAGAGATTACAGGTGTGCATCACCACACCTAATTTTTATATTTTTAGTAGAGATGGGGTTTCACCATGTTGTCCAGGCTGGTCTCGAACTGCTGACCTTGTGATCCACCCACCTCGGCCTCCCAAAATGCTGGGATTACAGGCATGAGCCACCACGCTTGGCCTTCCACATTCTTTATTAAAGAGTCACTTTGCCAGTGCAAATCTGGTAACTGCGTTCTCTAAGAGATTTGCACCAAATTATAAAATAATCAAATGAGGCCGGACACAGTGTCTCACGCCTATAATCCCAGCACTTCTGGAGATTGAGGCGGGCGGATCACCTGAGGTCAGGAGTTTGAGACCAGCCTGGTCAACATGGTGAAACCCCGTCTTTACCCAAAATACAAAAATTAGCTAGGTGTGGTGGCGGGCGCCTGTAATCCCAGCTACTCAGGAGGCTGAGGCAGGAGAGTCGCTAGAACCTTGGAGGCAGAGGTTGCAGTGAGCTGAGATTGCACCATTGCACTCCAGCCTGGGTGACAGAGCGAAGCTCAGTCTTAGAATAACAAAAATAATCATCATCATCATCATCATTATCATCAAATGGAAGTAACACTATACATTATCTATTTTCCCCTCTATGAATGATTAGCTCAAGATTTGGGGGGATCATGTAAGTTGCCAGTTATTTCTCAGTGAGTGTCAGATATTTATGACTTCTTTGAAGAGGCCAATCGTTTTTGAAAATTATTTCAGATGTGCTTTATTTCCCCCCACCCATCACAGTATTTTATTTCTGATTTCACTTAGATTTGCATAATGCAAAGAGCCACAGTTGCACTGGGGGTCCTTTCTAAATAGGCAACGAGGTTAGTGCAACCCACTGCTGACTTATTGAACCCAGGACCTGATGGAGTAGAGTTGATTCTTAAAGCAATGAGTGGGCATTTTGTGTTGTTTTTATGTTCAAGCTCCCATTGTGAGAATATGGTTACACTTTAGGGAAGAAAACCTCAACTCTTAGACAAACTTCTTTGCTTAACTAAAAATAAGTATATATACTCCTGTAAGAATTTGGTGATCATATTTTTATTTTTGTTAGTAAGATGGCCTCTGAGCTTTACACTTGATTGTCTAATCATATATTTTCTAATCATGTGTTTATTCACTCAAATTCACTCTGGCACGAATACACCAGTTCCACTTTGGCCTCATACTATAACTGTTAAAACTGTGTGGGACGTGGGGAAGAACACTGGTCTGTATGTCAAGATGGTCCCAGTTACTGAGAACTTTTGAGCAAATCTCTTCTCCTTTCTGGGGCTTAGATTCCTCATCGGCGAATAAAGTGGCTGAACCAAATGATCTTTAACTTTGAATTTTCCTGATCTAACTTTTCCTTTTCCTTTTCCTCCCTCTGCCTCCTCCTCCTCCTCCTCCTTCTCTCTCTCTCTCTCTCTCTCTCTCTCTCTGTTGCCTAGGCTGGAGTGCACTGGTCTGAGGATGGCTCACTGTAACCTCAACCTGCTGCACTCAAGTGATCCTCTGCACCTCAGCCTCCTACAGATGCCCCCATACCTGGCTTTTTTTTTTTTAATTTTGTATAGGCAGAGTCTTCTTGTATTGCCCAGACTGGTCTCAAATTCTTGGGTTCAAGCAATACTCCTGCCTAGACCTCCCAAAATGCTGGGATGACAGGCATGAGCCACTGAACTTGGCCCCTGATCTAATGTTCTAAAAAAATCTTAGCAAATGTGTGTTAAACTAAAAATGCTTTGAAATGGGATGACTCAGTAATAGAAACATAGAACCAAGCCCTTGCTTTAAGATGTCAAGTTGGATTTCTACAGAAATTCTGTAATTAGAATATAATATTGTAGTAAAAGCATTATCTTCATAGAACTAAGGAAATCTGAGTTCACTTCTTCATTACCCAAATTTAGTTGTGCAACCTTTAGAAATCATTTTATCAGGGATGCACTCCTGACCCTCTGTGATATCATGAAATACGTATTTGGACTTTGTCCAAGTTTCTTGGCATATAACTCCTAAAATCCTTGGAATTTCCAAAATGCTGCCTTTTTTGTATGCTAATGCTGGCTCATGGGTTCAGAATGGGGGTCTGGTCACTGGAAAGGCAAAGGCACAATTAGAGAGTCAGCCCCACCCCCCAGCCTCCAGGAAGGAGAGAGAGGCTGAAGGTTAAGTTGATCACCACTTGCCAATGGTTTAATCAATCATGGCTAGATAATGAAACCTTCATAAAAACCCAAAAGGACAGGTTTCAGCAAGGTTCAAGATAGTAAAACATGTGGAGAGTTTTAGAGGTTGAAGCCCAGGGAGGGCATGGAAACTCTGCACCTCTCCCCCCATACCACTCCCTAGATGTCTCTTCATCTTCATCCTCTGCAACATCCTTTATAATAAACCAGTAAATGTGTTTCCTTGAATTCTGTAAGCTGCTCCAGCAAATTAATCAAACCCAAATTGGGGTCATGGAAAACCCAATTTGAAGCTGGTTGGTCAGAAGTTCCAGAGGTCTGGATTTGCTCCTGGTTGTGTGTGGAGTCTTGGGAACTGAGTCTTCAACCTGTGCGATATGACAGATCTCCGGGTAGACAGTGTCACAGTTGAATTGGAGGACACCCAGCTTGGTGTCTGCTGCTTGGTGAGTGGTGAAAAACTCCCACACATTTGGCCACAGAAGTCATCTTCTGTGTTGATGATTGTTGCTGTGGTAATGTGAGAGGAGAGGAAAAAACACAGTTTGAGCATTTCCCAAAACATCCTCCAAGCTAAATAGACCCCCATGTTTTACCTTCTCTCCTTTTGCTTTTTTTTCAAGACATGTGTCACAATTTTAGAAATTATTTTTTTTAATGTCAGTACTTCCTTTGGATCAACTCCCTTAGTGTAAGGTTTACCGTTGGTTCCCTAATAGCCAGGACAAGGACTGGCATGAAGCGCGTGCTCAATAAATATTTGCTGAATTAATAAGCTTCACCTCTTTAAGTCTCAGTTTTATATCAGAAAATTAGGAGAAGAAGGTTTGGACAAGAGGTGCTATAATGTTCCTTCTGAGTTCTAACATCTTCTGTATTCTCTGTCTTCTAAAATCATAGGTCTGTAACAAGTTTACAGGTTGGTGCTACTCATAATAATTTATGTATTAAACATTTTAAGTGAGAAATATTTCAAGGTCAGGTGTGGTGGCTCATGCCCTATAATCCGAGTACTTTGACAGGCTGAGGTGGGTGGATCACACTTGAGGTCAGGAGTTTGAGACCAGCCTGACCAACATGGCAGAACGCTGTCTCTACTAAAAATACAAAAATTAGCCAGAAGTGGTGGCACTCGCCTGTAGTCCCAGCTGTTTGGGAGGCTGAGGCAGGTGAATTGCTTGAACCCAGGAGGTGGGTGTTGCAGGGAGCCAAGATCATACCACTTCCCTCCAGCCTGGGCAACAGAGTGAGACTCCATCCCCCCGTACCCCTTCCCCCACCGCCACCACCAAAAAAAGAATTATTTCTAACGTAAAAAAAGTAAGTAAATTAAGACATCTGAGTATAAGACAAAATGTTAACATTTTTCCATATTGCTTCAAATCTTTTCCTTAAATAAAATATAAAGCAAAAAGTACTCCTCCATTCTATCTCATTTCCCTTCTTTCAGCCATTAAAATGAATAAAATATACCTGTATATAAGAACATGGATAAATCTGGGAAGCATGGGAATTGAAAATAGATTACTAAGGCTAAATGTAGGATGATGTAAATTAAATTTTAAGGACACGGGGAGGGATAGCGTTAGGAGATATACCTAATGCTAAATGACGAGTTAATGGGTGCAGCACACCAACATGGCACATGTATACATATGTAACAAACCTGCACGTTGTGCACATGTACCCTAAAACTTAAAGTATAATAATAATAAAAATAAAAAAAATTTTAAGGACACGCAAAATTATATTACATATATTTATGGATGTGTATTAAAAATGTATGGGAGTAAATAAAATTCTTTTTTTATAAATGAATCTAGTAATTTGAAATGGTTAAGAACTTAATGATTATTAATAGATAAATATGAATTGTGTGATTTATTTTAAATGGTTAACATTCAAACTTTAGCAGACTTTTACTATGTCAACAAACACTTTCTCACCAGGTTGAGATACAGTTTTCACAGTGCTGTCTTAAAATTACTTAAGCTATGTTAAACACTTTATGTGGAGAATCTTAGAAATATGAGGCTTCAGTCATAATTGTTATACATGACTTTTATTTTGATCTTTGCAGTTGAGTAAGAATTTTTTAATCTATTTTTTCTTTCACCTTAAGAGATGTGAACTATAATTTTTTCCATTTTATATGTGTAATGGTGAAGTGGCCAAAATGTGGGTTAGATGTTATTGTATTTCAAAGCGAGCTTGGGAAGGGGCGCAAAGCAGTCTGCCTTTAAATGTGTTATTTCCAGCCAGGCGCGGTGGCTCACACTCGTAATCCCAGCACTTTGGGAGGCCGAGGCAGGTAGATCGCTTGAGCCCAAAGAGTGAGAGACCAGCCTGGGCAACATGGGGGAAACCCCGTCTCTGCTGAAGAAAAAGAAAAATAGAAAAAAAAATTAACCGGGCGTAGTGGTGGGCGCCTGTAATCCCAGCTACTCTGGAGGCTGAGGCAGGAGAATCACTTGAACCTGGGAGGCAGAGGTTGCCGTGAGACAGGATCGCGCCACTGCACTCCAGCCTGGGTGACAGAGCGAGACTCTGCCTCAAAAATAAATAAATAAAATAAAATGTGTTAGTTCCCCTTTGGAGCAGAAAGAAGTGGACACTTTTATAAGGTAAGGAGGGAAGTGAACAAGGGCAGGCGGTCCCCCTGCTAGCTTGGTGCCTCATCTACCTGATAGTTGAGTTGGCACCAACCCGGGCAGAAATAAGTTGTGAAAATGGCCAAGCAGGCAGACTTAAGATATGCCCTTCTGATGGGTGAAAGTCCCAAGGCCACCCCGTGAAGGTGAAAGTTCCATGGCAGGTGTTCTCTGGTTTGCAAATGGACTATCAGCTCTCCAGGAGAGATTTGTCTTGGAGCACACAGTTAGAACTTACCCTGCAGGGAGTATCTGGTGAGGAGGACGTGAAGGGTTATATTTGCATTTCTGAAGGGCTAAGTAGGAAACAGGGAACGGGAGGAAAGGGGACAGGATAAGAGAAAATAATAAAAATAATAGTAACTCATTCCTCTTTTCTTAGAAGAAATGGGAGTACTCAGTTAAGACTACATGAAGGCAGAGGGAGGGAATAAAAAGAGGAGGCAGAATTTAAATAGGATTTTTTAACTCCTAGTCCAATAAATAAATGATGCCCACAGTTGTTGGTGTTGGACAAGAGTCTGTTTTCTGTTGCTTTTAGATCTGAGAACTGAGCCCTCTCCTTCACAACAAAATTGGCCTATTAATTCCAGTGTTACTTCATGTAATTTTTTTATTTGTCCAGAACTAAAACTATCCTTTCTCTATTGTCGACAATTCCATTGGCTAAGCCAGGTTTCAATTGTCTGCTGAATATCAGGAGCTCTGTGTTAAAATTAACTCATTCATTCTGCTTTGATTTTAGAAAACAGATAAACAACTGATAGTATTTTTGCAAGCATCTTTTGTTCTAAAGCATAATATGTATTGGTCTGTTTTGGTACTTCCATGTATGTATTTCATGTTAAAACCAAGAAGGCTGAATTTAGTAAATCCAAAGAGTTTCCCCCATCATTTTCCCTTTTCATTCATTTCTACTGCCCTAGGTTGGGCCTTCCTCATTTCTTGTCTGAATAGCTCCTGATGGGTTTCCCCAATTTTACTTTGCTTGTTCCCACCACCTTTGTATTTCTGAATACAAAGAACTGATTAGGTCTTTCCCCTACCTTATAATCCTCAGTGACACCTCTTTGCCTGCTCATAGTAGTTAACGGTATCTTGTGACATCCAAGGCTCCTCAGAATCTGGTAAGTGCCTTTCTAGCTCAGCCTCATTGACTTCTGCTCGGCTACTCCTCCTCCTTCCCCCATCCCATCTATATTTCATCCACAGTTAACATCCCCTTGCTTTTACTGATGATATTCTGTCTTCATGGAAAGCCTACTGTTGTCTCCTGGCTTTTTCTTCCTTTCTTTCTTGGGTGCACAATCCAATTGGTGAATAATAACTTCTTTTTTAAGGCCTAGTTTAAATATCACTACCTCTATGAAAATTGTCTTTTCTATTTTAATTTTCTCTTTCTTATCCCAGCCAGTTCATCAGCTAATCTGGTGACTTCTCATCACCTCTACTGCTGGTCTAAGCCACTCTCATCTTACACTTGGATTATTGGCAGGAGCCCCCTGACTGGTCTTCCTGCTTCCGTCTTTGTTCCACAAAGTCTGTCCTTCACATAACAGCTACAGTAGTCTTATAGAAATAAAAATCAGACCACATCAGTTGCTCAAGCCCCTCTAGTGGCTTCCTATCTCACACGAAAAAAGCTCAAGACCCGTCCTGCTCTACAAGGCCTTGCGTGATCTGGGCTCCCTATTATCTCTGATCTTGGATCTTAACTCCTACCACTGCAGGCTTTGCTTACTTTTCTGCAGGTGCACTGGCCTCCATGGCAAGCATACCCAGCCAGGACTTTAGCACTTGCTGTCTCTAGTACTTGGAACACTATCTCTCCAGGTATATATATGGCCCAGCTCATTATTTCCTCCAGACTTTTTTCTTTAATATCATCTTATTGAAGAATTCTTTCCTAACAATCTTGGAAAAAATAATATCCTCTTCACACTCACTCCCCTGACTCTTGGCTTTTTATACTCTATCCCCTTTGAATTTACCCTGATTTACTTTTTCTTATAGCACTTATCACTATCTGACATAGTAGTTATTTGTTTATTCTAGAATTCTAGCATTTTTAGGGCATAGACTTTGTCTTTGTTTAATGCTGAATTTCCAAAACTAAAACAGAGCCTTGGTAATAGTATATGCTCAATAAAAATCAAATGTATTACCTAGTAGTTAATTGTTCTATCTCTGTAATCCCATAACACTTGCAGATGGTTCCTTTATACCCAGTGTATTTTGGTTGTTTTTGCTGTACTCTGCCTCTAGACTGTGAGCAACTTGAGGATAAAGATCGATTTTTTTCTTTCAATTTATTTTATATTTCAAGTACCATCCTAGGCAGCATGGATACATAAAATTCTGCATTCCAGGATTTTAAAGTCTTACTTCTATCTGTTCCCAGTACAGAATAAGGACCTAGCATTGTGCAATACATGCTCAGTAAGAGTTGGCTAGATTAAGTATTGAATGTTGTACAAATGAGAAGAGCTAGCTCTCATTTGTGGTTTATGATGGCAACTGACTAGCAGCTTTTAGTGTAGTCTCTCCTATGTTGTTGTTTTTATGTTTGTTTGTTTGAGATGGACTTTTGCTTTTGTTGTCCAGGCCGGAGTGCAATGGCGCAATCTCAGCTCACGGCGACCCCCGCCTCCTGAGTTCAAGCAATTCTCCTGCTTCATCCTCCCGAGTAGCTGGGATTACAGACATGTGCCACCACGCCCAGCTAATTTTTTTGTATTTTTAGTAGAGACATGGTTTCTCCATGTTGGTCAGGCTGGTCTCAAACTCCTAACCTCAGGTGGTCCACCCGTCTTGGCCTCCCAAAGTGCTGGGATCACAGATGTGAGCCACTGCGCCCGGCCTCTCCTAAGTTTGTATTAAAATATTAATATTACAAATTCCAGAAAAAATTAAACTAAATTGGACACTCAAGCCATTGGCAGAATTTTATAATAATTTTATGCCCTAAAAGCCTGATAGGATAGAACTGAAAAATGTCAAAATGAAATGAAAGCTGCAAACACTAGCTGTGGCTATATCTTGCTATGTTCATGGAAAAGTAAAAAAGCGAGAAAGGGCTTATATTAAAAACAAACAAAATCCTATCTATAGGGGAACATCGTTTTAAATACTCAAGAAAATCAACTTAGGAACTGGAAGAAATATTTAGAAGATTACTTTGGTGCAAATTAATAAGACAGTAACACTGTAAAGTTGGGAAGACATGGCAAACCTAAAATTGCAGGCTGAGATGTAAAGAGGACTAGTAAAAAGGGATGAAGATTAAATAAGGAAGGACATTTGAAATGCAGCAGAAGAAGTGAAAGTATTATTTGTAGTAGAATAGAGCAGAATTAAGTGTAGAAAACCATACACATACTGAAGACAAATTTAAGAAGCTTTCCCAAAATGCAAACAATAGGAAAAACTGCATAAAATATATGGCGGATAGAAAGTGGAGATCCTAAAGTAAGACTTAACGTGTTTCTGGAAACTGATACGAGCAGAAGAAACAAAAATACAATTGAAGAACAAAATGTATTGAGGTCAAGCATGAAAGTTAAAAGGACCCACCAATTTCCAAGTAACTTCCATTTTAAAAATCACATTTAGAGACAGTGCCACTTTATTTTTTAAAAAAGATAAACAAAATTCTACAAGAAACTATGGAAAAATAAGCAGCTTACTTAATTTAAAGAAATAAAATTCAGATTGACCTTGGACTTTTCTACACTGAAATGTACATTGACTCAATTACTCTGTTTTAAGAGATCTATCTAAAGCCAATTTATATACTAAACTGTGCTATATGGTGTTATTTTTAACTGGAGTAAAATTTATACAGAAGTATACTAAATTATACTATTCTACTAAAAAGTTACAATTAAATAAATAATGGTATATCCCTTTTTAATACTGTGACAGCCACCACTTAAAATCATGTTTTAAAATTAGAACATGCTCATGATATATTAAATTATAAAAAGAATACCGTCTTTGTATTATATGCCCCTAGTTTGTGTGTATATATGTGTGTGTGTGTGTATGTATGTATGTGTGTGTGTATGCACAGAAAACGGTCAACAATGTTAATAATGGTATGTATCTCTTGAGTAAGCAAGAAATCATTTTTTTCCAGAATTCTGTGTGCACATGTTTTGACAATGATGAAATATTAATAAAAGGCAGATTGTAGAAAAAGAGAAGTTGGGGAGACGTTGACAAAAGTTTTAAATTTTCTGTAACGGCTTCTCATAGTGACAACACTACAGGGCAAGGTACTTTATACCTTCACATAGAAGGTTTGAAATATTTGCTATCCACCCAAAGAGCTGCTGGAAATTTAATACAATGGGCTGGCATAATCAAAAAAGTTCTTAGCTATAGAAGATATTTTTTCAGGTATAAAATCAACTGAGTACTTAATTTAATGCCTATAAGTAGGAGAAACCTACATAGAGATATAAGGTAACCCTGGGAATAGCAATTAAAAAATCTGTTTAGAATGGTAAAGAAATGTGTAAAGGAGAAAATAAGTTTGAGCTAGAAATGGAACCCGTGAAACAAGCAGAAGACCTAGAGAATTAATTTTCTTAGGAATGTGAGTAAGTGTTGTATTTGATATACTGGTTATGAGGACTGATAAAAGATTTAAAAGAAATGCTTCACATATGACTCAGTATTAAGAATTAGATATATCAAAACTTTGTGGACATAGCTAAGAAGTACCTAGAAGAAACTTGGTAGAATTAAATGTTTATATTAGAAAAAAACGTGAAAGTCTAAAATGAATGATATATGATTCTAGTTCAAAAAAATAGCAAACTCAACCCTGGTTTGATTATTGCAGTCACATAAAAATAAAAATTTAAAGGTATGCAGATTTGAAAGGAAGAAGTAAAACTGTCCCTGTTCATAGTTGACATGATCATCTATGTAGATCGTAAGGAAGGCATCTACAAAAAGCTACAAGAACTAGTAAGTTTACCAAGGTCATAGGCTACATGGTCATTATACATGAATTAATTAGGTATAAATACATGACATAGTATAAAACGCATTAGAAACAAAAAGAAATTTTCATTTTTAAAAGTACTGTTTATAACAATTATTTTAAAATAAAATACTGAAGGATAAATCTAACAAAATAGGTACAGTATTTATACACTGAAAACTATAAAACATTTCTGAGTAAAATTAAAGATACTCTGTTTAAATGGAGAGATATCATATGCTCATGGTTTAAAAGACACCACATTGCTAAGGTATCAGTTCTCCCCTAAAAGACCTTACATATTTAACACAATCTCATGCAAAAATCTTACCAGGCATTTTTATGGAAATTGTAAACTGCATGTAAATTTTGTATGGAAAAATCAAAGGATATAAAATAGCCAGAACAATTTTTAAAAAGAACAAAATTGCAAGAATCAGACTACCTGATTTCAAGACTTACTATAAAGTAGATTAACCCAAACAATTTGAAATTGATATATGGATAGACAAATAGATCAATAGGAAAGAGGGAAGTATTCAGATACAGACATGAACACATATGACCAATTGATTTTCAATAAAGAGTCCAAGGCAGCTCAACAAGGGAAAATATGCAGGTAATCTTTTCAACAAATGGTGTTAGAACAATTGGATATCTGTTAAAAAGAGAAGAATTATAAACCCTTAACTCACACCATATACAACAATTGACTGAAAATATAGTTCTAAATGGAAGAGCTAAGACAATAAATCTACTTGAAGAAAACATAGAAAATCCTCCTAATGTCAGGCCAAGATTTCTTAGGATATAAAAAGCAAGAGCTTTCAAGGGAAAATATTGATAATTAAGTATTTTGGGAAAGCAGGAAAAAAGCCAAGCTACAGGCTGGGAGAAAGTATATTTTTAAATGTATATCTGATAAAAGACTTGTAGCCAAAATACATAAGGGACTCCTAAAACTCACATTAAAAACAAAAGCAACCTAACTGACAAATTACCCCAAATCGCCAAAATAACCCAATTAAAAAATGGCCAAAAGACCGGAATGGATATTTGTCAAAATAGGATAAGCATATGAAAAATGCCCAATATCACTAATCAGGGAAATGCAAATCAAACTACAATGAAATACTACTTCATTTAGAATGGCTATTAACAACAAGATAACTGTTAGCGAGGATGTGGAGAGAAGAGAACCTTTACATACTTTCAGTGGGAATGTAAATTAGTACAGCCATTATGGAAAATAGTATAGAAGTTCCTTGAAAAATTAAAAATATAACTACCATGTGATCCAGCAATCTCACTACTAGGCATTTATCCAAAGGGAATAAAATCAGTATGTCAGAGGGATATCTTCACTCCTGTTTATTGCAGCACTATTTGCAATAGCCAAGATATGCAATCAACCTAAGTGTCCATCAAAGGATAAATGGATAAGAAAATGTTGTGTATAGGCCGGGCGCGGTGGCTCACGCCTGTAATCCCAGCACTTTGGGAGGCCGAGGCGGGCGGATCACGAGGTCAGGAGATCGAGACCATCCTGGCTAACACGGTGAAACCCCGTCTCTACTAAAAATACAAAAAATTAGCCGGGCGAGGTGGCGGGCGCCTGTAGTCCCAGCTACTCGGGAGGCTGAGGCAGGAGAATGGCGTGAACCCCAGGGGGCGGAGCCTGCAGTGAGCCGAGATTGCGCCACTGCACTCCAGCCTGGGCGACAGCGAGACTCCGTCTCAAAAAAAAAAAAAAAAAAAAAAAAAAGAAAATGTTGTGTATATGCACAGTAGAATACTATTCAGTCAGAAAAACATAATGAAATCCTGAGGTTGGGCATAGTGGCTCATGCCTGTAATCCCAGTACTTTGGGAGGCTGAGACAGGAGGATCACTTAAGCCTAAGAGTTTGAGACAAGCTTGGGCAAAATAGTGAGACCCCATCTCTACAAAAAACATACACCAAAATTAGCTGGGCATAGTAGTGCGCACCTGTAGCCCTAGCTACTCAGGAGGCTGAAATGGGAGAATTGCTTGAGTCTGGGAGGTCGAGGATGCAGTGAGCCGTGATGGTGCCACTGTACTGCAGCCTGAGTGACAGAGCAAGAACCTGTCTCAAAAAATAAAAAAAGAAATCCTGATCCTGTCATTTGCAACAACATGGATAAATCTGAGGACATTATATTAAGTGAAATAAGCCAGGCACAGAAAGATGTAACCACAAGATCTCACTCAAATGGAATCTAAAAATGTTAATCTCATAGAAGTTGAGAGTAGAATAGGGGTTACCAGAGGCTGAGAAGGGGACACGGGGTGAGGGGAAATGGGGAAAAGATGGGTCAATGGGTACAATATTACAGTTAGACAGAAGGAATAAGTTCTGTTCTATTGCACAGTAGGATGACTATAGTTAACAGTAATGTATTGCATATTTCAAAATAGCTAGAAGGGAGGATTTTGAATGTTCTTACTACAAAGAAATGATAAATGTTTAAGGCGATGGATATACTACATATCCTAATTTGATCATTATACAATATAGACATGTATCAAAACATCACATTTTACCTATAAATATGTACAATTCTTTTGTATCAATCATAAAATAAAACCTAAAAAGTGAAATTTCTGTGTTACAAAGGGGGAGAACACAGTCTGTATTACAATTTTTTTAACTCAAAAGGTACAAAATATTTGAACAGATGCTTCAAAAAATATCAAATAGTTGTATAGAATTACATAAGTTTGTTTTTATTTTTATTATATATAAATATGAATGAAATAAGTACATGAAAAGATGTTAAACATTATTAATCATCAGAGGAATGTTAATTTAAACCACAATGAGATCTGCATACATACCAGAATGACATCAATTAAAATAACAGCTGACAATACCAAATGCTGACAAGGATATGAAGTCCTCATATATTGCAAGTGACAATGTAAAATGGCACAACCACTTTGTAAAACTATTTGGGAGTTTGTTAGTAAGCATACACTTACCCAGCAATCATATTCCTAGGTATTCAAGAAAAATGGAAATGTACATCCTCACAAAAATTTGTACATGAACGTTCATAATAGTTTTATACACAATAGAAAAAAATGGAAATAATTGGTGAGTTGACAAATAATGAAGTGCTATTCAAAAATACAATGGAATGCTGATACGTGCAAAAACCACAGATAAATCTCAAAAGCCTTATCACATAAGATTATATACTATATTTTTCCATTTACATAAAATTCTGGAAAGGTAAAACTAGTGGAGGAAACAGGTACATGATTGTTGAGGCCAGGAGCTAGGGAGGGGATGAACTGCAAAGAAGCAAAAGATAATTGGAACAGGAATGGAAATATTCTATATCTTGATTGTAGTAGTAGTTGCACAACGGTGTACATTTGTCAAGCCATCAGCTTTTGCACTTAAAAACTGGTATACTTTGCGGAAAAGTATACCACATAAAACAAAACCCCCATACAAAAAAGAATAAATTAGGCCTTAGAATGAAAAGAATCTCAAGTCAATGTTGAGGAGCAATTTAGACGTGAATTTTTTAAATTCCACAGAGAAAGAAAGATAGTAAAATAAGCCATGTGTGTTCAAATTTACAATTTCTGCTGACTTGAATCATCAGGGTATTTTAAAATCTTGCTGTAAATCAGATTCCCATGTTCCAATCTTGCAGAAGCCGGGAAAATTGGAGATGATGGTGCTATTTTGTAAGGGATACGGAAGTGGGCAAGTCTAAGGACTTGTTTATACACATAGACCAGGGAGACTGAACTTAGTACTGGGTAAAAAAAATAAAGGTCATCATGGAACAGATTTGCAAACAATGAAGTAAACAGAATAGCAGAGATTTGGCTTTGTAAAAAGAAAAAAAAGCGATTGTGCCAGACCACTTCAGCGCCTCTAGTTGCTGAATTGCAGGACTGGTAGTTGAACCAAAAGCAATAAACATAATGCATCTTGGCTGTGGTCAGATATTTTGATTTAGTCAAATATGAAGCCCTCACCACTAAAATTGGAAAATATGGTTTGCAGTACAGATACAGTACTAGCTGAAAAGTGATATCTGAATTGTCCTGGCATGACGTGGAGAGCAGAGGAAAAAGAGAAGTGTACCACACTGGGAATATCACTTTTGTTGTTGTAGTTGTTGTTGCTTGCTTGTTGCTTTTAATTTTTTTAGGTTTCAGATTCAGTAGGTACATGTTCAGTATTGTTACATGAATATATTGTATAATGGTGAGGTTTGGGTTTCTAGTGTACCTGTCACCCAAACAGTGAACATTGTACCCAATAGGTATTTTTTAACCCGCATCCCCTCCTACCTTCCTCATTTTTGGAGTCCTCAGTGTCCATTATTTCCATCTTTATGTCCATGTGTATCCTTTTGGGGTTTTTGTGGTTTTTTTTCGTATGTTTAGTAGAGACTGGATTTCTCCATGTTGGCCAGGCATGTCTTGAGCTCCTGCCTGCCTCGGCCTCCCAAAGTGCTGGGATTACAGGCATGAGCCACTGTGCCCGACCACATGTGTCTTCACTACTTATCTCCCACATATAAGTGAGGACACATGATACTTGATTTTCTTTTTCTGAGTTGTTTCACTTAGGATAATGGCCTTCAGCTCAATCCATGTTGCTGCAAAGGACATGATTTCAGGGAATAGCATTTTGAATTTTGGGTGATGATCCTATGGTGTGTTTGAAGCATTAAAACGTTTGTATGGGTAGAACATGGGGTGAGAAATGTGTGTGGGTGGGGGTGGAGGGGTAGGGGGAATTAGAGATAGATAAGGCCATAATACAGAGGGCCTCATAAAGTCCTCAATAAACCATGTTAAAGAGCAAGACTGTATCCTTGAATTCCCAGAAGCAGCTGGTGTAATCGTCCAGGCTGTCTTCCACCTAGCCCAAAGTTTTTTCAACTCTATCACTTGCTGCCTCTCCCTACCATTAAATGAGTAAGCAATATACTGAGAAATTAATAAAACTATGTTAAATAGACCACTTTTGAGCTCCATTTTAATGCTTGGAGAAGAGTCGGTAAGCATTGTAATTGATTCAGAAAAGAACCACAATGTTGGAAACTAGAAAAAGGTAAACATTTGCATGGCTGAGTTAAGAGAACAGAGACATAAGAAACTGTTATTACAGGACTAGGATAGACTCATATACCAGTTACTAAGCAGGTTCTTGGGCAAGTCCTTTCTGTACATTTATTTTCTTATTTATAAAATAGGGATGATAATTCAAGTCAATTATCTGTTATCTACAATTCTGAAGTTCCAAGAGCTCTGAAAATCAGAAGTTGGTTCAGTTTTATTTTGATTTCTTAAGTTTGTGGCACACTCATTTGATGGTGAAACCCAGGTTTCGTAGTCATTTATCAGTTAGTGTGAATATTTATGAATTCTGCAGAAATAATGTTTGATCTTATGGTGCTACCCTAAATTCTGCTGGATACATTCATATGTTATGCAAACTACTATTTTTCTAAAATCTAAACAATTCTGGATTCTGAAAATTCTGGCCCCTGTGGTTTCAGATAAGAGACAGTATGCTTGTAATACCTTCCTCTTAGGGTTGTATCAGGATAAAATTTCTTAAGGCAGAACTCAGTAAACGATGAAGCTATTATTATTTATCATTAATTAAGCTTAAAAATTAGATTCCCACCCCCATCCCCGAGAATGAACAAGAATTAGCACTTGACGATCCTTAATTTTATTCTTAATGCTTTTATACTGTACTCTCTCGAGCTTGATTTATAGTATATCAGTTATTAAAATGGAATGTTTTCCTTAAGAAACTTCTACGCTGAACACTTAACCTTCATTTCTGCAATCTTTACATACTTGGAAGGATACCTTATTTGCCTTTAGGTATTAGTTGACAGACTTATTTTTCAGTCATCTTTCCTAAGGGTCATCTTACGAAATTCATGGTTAGATTATGCGTTATATACCTCCTAACGAAAACACGAATGGATATGCTTATTTTCTTCAAGTTCTCAATAAAAGGAAGTTCTTAACATGTCATAAAACAAGTTCTGTGTAAGTCTTTAGTTAAAAGCACTAGAGCAAAAATACCCATCTAGATTTCATTCAGTGTTTCCCAAGTAGAATTTTATTTTTGTGGTTGAAGTTTGGCATGTTTCCTATTTCTTCAAGCTATATAAAACCCTTTTGCTGCGTCTAATCCATGGCCTGCGGACATGGAGGACTGATTTTGCAATACTTTTCTAGCATTGGGAAACTTCTAACTAAAAATTTTGTGTGCTGGAATACTTGGAAGAAAAGGAAAGGTCATTTAAACAAATACTATATTTTAAAATGAAGAAATAAAGCAACATTTTAAATGTGTGATTTTTGTTAAGATACTTGCCATTAGTGAGAGTTAGGGTGGGACATATCAGTAACGAATCCGATTGCCACTATGGCTCTGCCCCCCCACCATCTTTCCTCTGGGAGGATGTTGGGGCCAACTTACGATTGCAACCTGTGTGTGTGTTTATCCTTTGTTTGGATACATGTTACTAGGTGCCCCACTCTTGGCCAGCAATTCCAGTGCCTGGGTGGTTTGCCTGAGCAAAGTGCCTGAGTGGCTAGGAATGTAGAGATAGGGCTTTGACCCAGAGCACAGGGATGATAAGGTTTACTATAGTGTAAGGATTAGAATGAAGGTTCTCTTCTGCTTCAGAAGTGGGAAGAGGAAGCAACACAGGATGGGATCTCTATCTGGTTCTAGGTCTCAGGCTTTCTCCAGCCCGTTTTGCCAACTTACAAGCAACTAACCACCACTAAACTTCCTCTCCCTGGAGAAGGAAAAATTTCACTCACTTGTGAGTGAGAAGATCTGCATCCAGATCCTTTCTGTGCCATGATTAGCTGTGCTACATCAGGCCCGGTGTTTTCAGGAGTTTAGGACCAAACAGCCCCTTTCAGCTCTTGCATTCTCCTAGTCTTTGACATCAGAAGGAAAGTGCAGGCGTTCAAGGGAGTTCTTGTTTTACAGTTTCAAGCGATCTGACGTCAGGCGAAATCTCTTCACTCTCTAACAGATATTCCATCTTAAAAAAAATCCCATGAACCCTAATTCCTGTGAGTGTCTCTAAAGTATATTAAAGACTGGATTAATTGTAATCTAGGCAGAAATCTGTCTTTGTGGTTGTTGTTTAATATCACTCAGTACATGCCAATACTCTTCTTTCACTTAGCTTCTGGTAATCTATGAAGCTACTCGTGGTTTCATTCATTTAAACCCCTCTGAGCCTCAGCTTCTTCATGCTAAAGGAAAATAACAGGAAATAATGGCATAAATGTGTTTTGATAAACATAAAACCTTTTGTAAACATGGGGTTTTATTTATGATAGCACAAAATATTAATATAACTTCCTCTAATAACATTGACATACGTCACATCTATAGTAGCCAGTAGTAAACAGGCAGCATGCAGCTCTAAAAAGTGGACAGATTTTCATTGTTCTGATTTGGAATGTAACAATATCTCCTGTTGGTTCTCTCCTTCCTTTCTCCACTGGCCCCAACTCTAAGCAGTATTCCACTGAACTGAAGAAACTCTACTGCCAAATTGCAAAGACATGCCCCATCCAGATCAAGGTGATGACCCCACCTCCTCAGGGAGCTGTTATCCGCGCCATGCCTGTCTACAAAAAAGCTGAGCACGTCACGGAGGTGGTGAAGCGGTGCCCCAACCATGAGCTGAGCCGTGAATTCAACGAGGGTAAGCAGAATTTGAATCTCTAACTGTTCAACCTCCTTGAAGGTCAAGATTCTGTGGGCATTTTTGTTTGAGACCCACCTACCTGATTCAGACTTCTGCACTCCGATGGCAGATCAGTCTGCCTTTTTTTTTTTTTTTTTTTTTTTTGGCTGACAGTATCTAAGAATAATGATAAATAAATTCATGGCTTTCACAGTGGTTGAACTATTTAATATTAGTAATTACTAGATGACCTGAACTACTGGTTATCTTTACTCCTTCCTGCTTCCTTCTTTTTATTTTTCTTGCCTTTAAAATAAAATAAGAGGCTGGGTACGGTGGCTCATGCCCGTAATCCCAGCACTTTGCGAGGCCGAGGTGGGCAGATCATGAGGTCAGGAGATCGAGACCATCCTGGCTAACACAGTGAAACTCCATCTCTACTAAAAATACAAAAAAATTAGCTGGGCATGGTGGCGGGCACCTGTAGTCCCAGCTACTCGGGAGGCTGAGGCAGGAGAATGGTGTGAACCCAGGAGGCCAAGCTTGCAGTGAACCAAGATGGCACCACTGCACTCCAGTCTGGGCGACAGAGCGAGACTCCATCTCAAAAATAAAAAATAAATGAAAAAAAAATAAGAGGGTGCCAGGAGACTACATGGTAGCTATTCTACTTGGCCCAGGCTACAGTTTCTTCAGATTTAGTGTTTTCCCAGAAAGATTCTCAAGTCTAGACCAGTTGAGACCTGGAAGGGTAAGAGAAAGGAGCACCAAATGAAAGAAATAATGAAAAAACCAGAAGAAGAAAAAGGAAAAAAAGATGAGAAGGCAAGTAATGGGTAAGGGGAATAAATAGAAGGCCTAGAGCATACTGTGGCCAATCAAATAAAGGACTGGGAGTGGAGGGATGAGGTATCATGAAGGCCACCTTTAGACGCTATATCCATGAATAAGTCATGCCTCATATTCTACATCTCAGATCCTACAACAATTTAGCAGAAAAGGTCAGCAATGCCAAGAGTTCCCTTAGATCTGATATTATAGTTCCATGAAGGGAGACTAAACAGAGAGTGCTGAAAAGTGGAAGGTAAGAGCAGGATAAAGCATAAGCAATCCTCAAAGAGTATTGAGGGTGCCAAAGGTGTTGGGGGTTAGGATACTTTATAGAAAAAATATTAAAATATAAGGATTGATAAATATAGAGAGTATAATTGTAGAGAAAATCAATGGAAGAAGAAAGAGAATGGATCGGAATTTAAAAGAAGAAAATAAAGTCAAAATTCTCTTTAAAAAACTATTTTTTACACATTAATTTCAAGTCCTTGCCCACATGGCCACATTATCCGTACATTTATCTCTTGCTTTTCACTTAACAATCTGCCATAAAGAATATTGCAATATAGATAGACTTCATTATCAGCCTCCCTAACTTGGTATTTCACCAAGATAACATACTATTTGTAAGCGTTTTCCCATTTTTTAAACATAAAAATTGTTTTTAGTTTTAACCATAATAGATGATGCAGCAGAGAACATTTTGATATGCTTCACTTTTTACTTTTATTGAATGGTTTTTGTAGACTGGGACTATAGGACCTAAGAGCTGTTAAAAACAGCTTTACGGGTCCCAGTTCTTGGAGCATCTTCTTTTTTTTGGAGCTCTGCTAAATATCACAATTTTATGAAACTTTACTCAGTCTCTTTTTTAAAATTGCATCCCTTTTCTTACACCCAACTTGGGACTCCTTCAACACACAAACACCAGAACGTTTCTTATCTTTATAATCAACACAATGTTTAAAAGTTGAGGAGGAGGAAAGAAGAAGAAGTGGAAGTCATATCCTTGCTATCTTCTGCTTCTCAGTGTATTTGCTGGTCAGACTTTTGGGCAGCATTTTCTCAGTTAAACTGATTTTTTTTTAAATTAATTTGCTTATTTCCCTTTCATTTTCCAGGAAGCTGGCAAAATTTCCTCCACGATGAGGTCAGAGATGTAGTTATAGAGTTCCATAAAGCTTGAATCATTAATCTTAAACTCCAAATTATAGGACTGTTCCCTTAACTGTAGATGTGCATTCCAGCCCTTTCTGTTGCTATAAAGGTTATGACACCTTCATGTATTTGATATCTGGCTATGGGATCTGTTCGTTTCTTCAAGGATGCACATTTTCTTTATTATACAGACTATTTCTTTTGCCACCAACATCCTGTTCATGCAATTTCATTTTATTTTCTTTATTTTTAAGGTAAAGAACTAACTCTTTTATTGTTTTCTGCTCTGCAGGACAGATTGCCCCTCCTAGTCATTTGATTCGAGTAGAGGGGAACAGCCATGCCCAGTATGTAGAAGATCCCATCACAGGAAGACAGAGTGTGCTGGTACCTTATGAGCCACCCCAGGTAAAAAGCAAAAAACCAAACCAAAAAACAACACCTCTATGGACTGAGTAGACTTGAGAGAACATCTGTTTCAGCAACAGGGATGTTTCTAGCATCTATCACTGTCTTAGTTCTTGTCATCAAATATATAATATTTTTGTATTTTTTTCTACCTGTTCTCCACATGCTTTCCCTTCAGAATACCTAAATATGCACTGAATCAACTTGGTAACATGGCTCTGTGTCCATATTCCCATCTTAAGGGCAGATAACAATTGAACTGGCAATTGGGTGTATACATGAGATTGTTTTATCCAACTGGCAGTTGGACGGGTAGTGGATAAGCAATAGTAGTGGATGCAAGTGTTACCATAAGTGGGAAAGCCTAAGAGGGATCTCTTAGCTGAAGTTTGAATGAACAGATGATAGATTTGTAAATCTGTTGCATGAAGCAGAGTAGTGTGGTTGAAAGTACACTGGTGTAACAGGACCCAAGTTCTTTGGTTTGCTGGCTTGCTGTGTGGACCTTCGGCAACTCTCTCTCTCTAGGCTTCAGTTGCCTTACCCGTACGTATGTACTTTGGACTAGATTATGTTTAATTCTGAGAACATTTTGAGCTCTAAGATGTTATTAATGGAAGTATATATTCAAATCTATATTGTACTGTTTTATCCACACAGGGAAAATATTCTTGGAGCATTGCTACTGTTTTTGGCAATTATCCCCAGTTTTCCCTTTTCAGTGATATTCATGAAGGTGGTATAGCCTATTTGAATTACATGATGTGGATCAGCTTACAAACGAACAGGATCAAAGATCACAAAATGTTAAAGCCTGTCTCACCTAAGGTAGTGTTCAGTGTTGCAAACATTAGCTTTAAGCTTCCCCGCAGGCAAGATGAAGAAAAATCTACAACAGGGTTCAGAGTTTGCCCTTTTAGGAGGAAGCGTATCACTTCATCAGAAGTGGAATTCCTTAAATAGAGGGAAGAACTGAGAAGGAACAACGTCAGTTTAAACCCTTGTTAACACAGATTATTTACCCCTTGTTTTCAGGTTGGCACTGAATTCACGACAGTCTTGTACAATTTCATGTGTAACAGCAGTTGTGTTGGAGGGATGAACCGCCGTCCAATTTTAATCATTGTTACTCTGGAAACCAGAGAGTAAGTGGCGTATGTAAAATTGTCATTCTACACAAAAAATCACGAGCAGAGGGCAAAGTGAAATCGTGGCTGCTTTATCATTAATTTTGCATGTGCAGCGGAGAGCTTGTCCTTTGTGCTCTAAATCCTTGCTACAAACGGTTACATAAAAGATCTAAGAAAGTGGAGACAAAGGAAGGTGGGTAAAGTTAGAAGGAAAAAAAGAGCTAGAAAAGTGTGCAAGTCACTTCATACCTGAATTCTTGACATTTGACTGGAATTGTTCTGATTAGACCATGGTCCTCAAGGCATTTCACAGTTTTTTTTAAGTCTGCGCTGCCTTAGGGGATTTTATCCTTGAGACATCCACTGGCTTAACTCAAGTTTCCTTCAAAATATGTAGCTAAATACAGCTGTTCAGCTAATAGCTCAGAGGTTCTTTGGAGAACAAATGGAATGTTATTTACTAATATTACTTGTGGCATGTTAGCACTTTTGTGTTCTGCCAAGTGCTTTTGGGTCCATTCTCAAAGCCGCCATGGCTAAGCTGGTAGTACGTTGGCGATGGCCCATATGGGAAGTGGAAGTGGTAGATCTTCAGGGGACTTTCAAAATGCTTTGAATTTAACTCTTTCTTCCCCTTTATTCTAATTCCTAGTGGGCAAGTCCTGGGCCGACGCTGCTTTGAGGCCCGGATCTGTGCTTGCCCAGGAAGAGACAGGAAGGCGGATGAAGATAGCATCAGAAAGCAGCAAGTTTCGGACAGTACAAAGAACGGTGATGGTACGAAGCGCCGTAAGTAGATGTAGTGGCCAAATGGGGTAGGGTTGAATCTTCTCCAGATGTTGGAGAATGGGGTGATATTGGAGAAGCTGCATGATAAGACCTGTGACCTTCAGCAGCAAGTGGGACGTCAGCCCTCAGAGCCAGTGAGAATAGGTATAGCATTGAAGTGGACTCCAGGCATGTACTACAGCTTTACAAAAACAAGTAGTCATTGTGACAATTTCTCAGTCCAGGGATTCTCAAAATGTGACTTCCACACCAGTAGCATAAACATCATCTATTCACAGACACACACCAGCAAGATACGGGCTTTCCTGCAACATTACTTAAGCCAAAGGCTATTTCCCAGCATACTGAATTTCTTTTATGTATTCTTCCCTACCATCCTTCCACCTCCTTATTGCTAGGATCACAATCAATGTAAAAACAATTGTCCCCAGTTAAATGGACGCTTAGGGCTAGAGCCTCTAATCTTACATGTGTTGCTGGTACTACTGTCTTTTTAATATGTATATTAAATCTGATTAACATTAATATTTAATTATTAAGTATATTTAATATGCATTAGTGCTTTAGAAGTGTTCCCAGGATGAAACTTGCATTTTTCCTCCACCAGCGTTTCGTCAGAACACACATGGTATCCAGATGACATCCATCAAGAAACGAAGATCCCCAGATGATGAACTGTTATACTTACCAGTAGGTCTTCCTTGGGTGTTCATGGTTGCTTCATTTTAACCTTCTTTGAATGGGCTTTTACAGTATGATCATCATCTCATTATCTGTGACAATGGGAAAGGAGGTGTCTTAGTCAGTTGAAGCTGCTACAACAAACTACCGTAGACTAGATGGCTTATAAACAACAGAAGTTTATTTTCTAGAGTCTGGAAGTCTGAGATCAAGGTGCCCCTATGGTCGGGTTCTGGTGAGAGTTCTCTTCTGAGTTGCAAACTGCCATTTTCTCCTTGTATTCTCATATAGTGGAGAGGGCTAGAGAGCTCTCTAGGGTGTTTTTTTTTTTTTTATAAGGGCAAAATCCCATTTATGAAGGCTCCACTCTCATTACCTAAATTACTTCCCAAAGGACTCACCTTCTAATGTCATCACAATGGGGTTAAAATTTCCATCTGTGAACTTTGGAAGGACAGAAACCTTCAGTCCATAATGGGGAAGAGGGGAGACGGTTGAGAGCTGGTGATACAAAGGAGAGAAAATATGTGTTTTTTAAAATTAGAATATTATTCTTTCTCTCCACAGTTTTGGACTGTATTACTAGCATGAGCTTCTCTACTTACCTACCCACCCAACTAAAGCTAAACTCCTAACAGAATTTTTCTTACCCTAATATTTAAAATTAATTTGTCCTTCATTATTTGAGAATGATAAGATTATCCTAGGCAGCATGGCCATAGAAATTTAGGCCGAAAATGTTGTCAGAAAAAGTTCACAAAATTGATAATGCAGCAATGATAGCCTTGACTTTGATGTCTTTTAGGCAGCACTATAAAGAAAGCCAAGCAAGGCAGAAGCCTGAATTACAGTCAGCCCTCCCTATCTGCCTGTTCCATATCTGCAGATTCAACCAATCACAGATCAAAAATAGTTGGAAAAAAACAATAAAAAAATACACCAATCAAAATAATACAAATATAACAACAATATAGTATAACAACTAATTGCATGGCATTTACATTATATTAGATATTATAAGTAATCTAGAGGTAATTTTAAACTATACAGAGGATATGCATAGGTTACGTGCAAATACTACACCATTTTATATAAGGGACTTGAGCAACCTAGGATTTTAGTATCCATGGGGGCCAGATGGGGGTGGGTAGTCCTGGAATCAATGCTCTGCTGATATCAAAAGACAACTGTGTATGTAAATGGCCAGAAGTCTTAGCCCAATTATTATGTAAGAACCTCATTAATTCCTACAAGGGACATTACTTCTCTTGCAATCACTAATGAGATCACTTTATTAGATTATGGTTGCAGTTAGTTAGTTGAATTCTTATTGCATTAGGTCTTTGCCATGTAGGGTGGCAATGACGTTTTGGTGGCTGGCATAGCCCTTGATGCCATCTAAAGCGATGACATGAGACACAGCCAAGCAGGGCCAGGAGATTTGACCATGAACTACTACCCTGGGATTGAACTAACGAAAGTCAACGGATTTAGGTGTGCTGCACTTTCTTCCTCATCTTCTTTGGGTCCTGACTCTCAGGGGAATAAATTGCCAGTAGACCAATCAGACGGTGAGTTAGCTGAGATTCCCAGTGTCTTGTTTCTCAAGGTATATATGGAAACTGACTTGTGATGTTAAAGTAAAACATTACCCTGATACACTAAATATTCCAAAAGTCTTAATATGTTTTCAGCTGATAAAACAGGAACTGCTTGAAAAATTATTTTAAAATTTCAGTTTGTACTAAGAAGGAGGTATCCTTCTTAGTGTCATGTCTCAAGTTGAGAGAAGACTTAGCCTAAATAATTTATGGGTGAGGAAAAACTCCATTAATTTAATATATATTTTGTTGGTGTTTGGTGAGTGGCAAATATGCTAGACCTTGAGCATTTAGAGTTCAGTAGGATACAATTCTTACTTTCAACTACCTTGCCTTCTATTGAGGACACAAACAGTTATAATGTAATAAATAAATGTAATGTAATAAAATAAATATTCCTAGATCAAGGCCTAGTGCTGTGTGAGTACCCAAGAAGCTCCAAAGATGCAAAAACTATAAATAAGAGTAAGATCAAGAAAAGCTTCTCAGAGAAGGGGTCCCACAGCTGGGATGAGTAAGAGTTAATTCAACAAAAGGTGGAGAACATTTTTCATTTCTGAATTCTGACTAATTCTGCCTTTCCTACTGGGTGCAGCAACTGGGGTAAGGCAGTTAAAGTGCCTAGGGTGCAAAATTTAAATAGTCACTCACACCCAAGGCAATGCAGTTGTGCTAAGTGCCTCTCTTGCCTCACTCTAGTCCTGGCCCGGCTCATCCGTTTTTTTGGTTTTTGTTTGTTTTGAAACAGGATCTCACTTTGTTGCCCAGGTTGGAGTGTAGTGGCAGGATCATGGTTCACTGCAGCCTCAGACTCCCAGACTCCAGCAATCCTCCCACCTCACCTCCCCAGGTAGCTGGGACCACAGGTGTGGGCCACCACGCCCACTAATTTTTGCACATTTTGTAGAGAAGGAGTTTTTTTCCCAGGGTGGTCTTGAACTACTGAGCTCAAGCAATCTGTTCCCTCCCAAATTGCCGGGATTGTAGGTGTGAGCCACCATGTCTGGCCCCTGCTCATAATTGTAAAAGCCTTTAAGAGCTTTTATTTAAAGATTTTAAGGGACTCTCTTAAAGAGAGAAACATATTTTATCCCACATGGAAACTTTTATCCTATCAAACATAGTTACAATCTATTCTTATTTTTTGTAGTAGTTTAGTTTTATGAAGGTGTTGCAAAAATGGTATTGGTGGATATGAATCATCTCATCGCTCCTAAGAGAAATATAGGATTGGGTTCCTATGCACCTCTGGTCACATACATTTCACAACAGAATAATATATCACTTCGTTTGTTGTGTGTTTCTGTTAAGAGACATTTTATTTAATATATACCGTTGACTCATTAACATCAGACTCGTGGCCAATTAGCCTTATAACTCATGCCTGAATAAAGTTTCTCTAACACACACACACACACACACACACACACACACATATTTTCTCCATAAGGCATGGAACGACCTTCTCTCACTTAGGGACACTAGATAGCACTTCCGCACTGCCCTTGAGGGTCTTTTAAATAACAAAATCACCAATGAAAAGCACAAAAATGTAAAACACGTGGCGCTAAATAGACCATGAAAAGAACGTGGGTTTACGGTATAAGAGCTACAAGATGACAGAGCATCTCCTTGTTCGAACCAGCTGGGAATGCACACAGGTGACTCAAATTTTTCATCACTCTGCCCGGGTCTGAAAGACCACAAAAGGGCCAAGAGTGTTGATTTTCATGTTACAAATAAACGTTAGCAAATAAACAAATTGCAATTACGGAATCCTCAAATAATGAGGATTGACCACACTTCTAACAGTTCTACAGCTTTTCATGTTTCCTTCTTTCCTTCTGCTCACTTCCATAGGTGAGGGGCCGTGAGACTTATGAAATGCTGTTGAAGATCAAAGAGTCCCTGGAACTCATGCAGTACCTTCCTCAGCACACAATTGAAACGTACAGGCAACAGCAACAGCAGCAGCACCAGCACTTACTTCAGAAACAGTGAGTGTATCAACGTGTCATTTTAGGAGGCATGAGTGAGGGTGACTTTATTTGGATCAGCAATAGGGTGATTGATGAGCAATGTGGAACATAATGGGAGATAGCAGATTGTCATAGATTCAGATGACCTGGTATGGCAACCCTCTTTCAGTTGCAACCTTTTTTACGTGTCTTATTATAACCTTCCCTTCAGAATTCCACTTATGTTCTGAAATTAAATACAAACCATTTCTGGTGAATTACAAAGAAACTCACACTAACAGTTCTCGTCTCTATATGCCTGGTCCATACACACTAACAGTAAGTACACACTCTATTTGGTAGTGATGTGTATATTTGAAAACATGAAATCTTTTCTCATCCCAATGGATTGTCTTATAAATCTCCTGGGATGCACACTATCCACTTTTGGGAATAACACTGTAGACCAGGGATAGCAAATAGGCTTTACTATAATATAAAGTGACTTGTTTGAATGCTGTAATGAGAAGAATTCTGAGACCTAGTGCATGATAATTGGGGAAATATCTGGGTGCAGAAGGATAAGGTAGCATCATGTTGCCGTATTTTAGAATCTCTGCTCTAGATTGTCTTCCAGCAGTACATTTTTGGCATAAATCAAGCCAGATTTTTCAAGTATATTACCATATACATATAACCTATTTATATGTTAAGCTTTGCCTTGAATCTTGGATCTTTCCTAAAGTGATTTTATGACTGACTAGAATAGAATAGCTCTTATGAAAGGATGAGAGGTTGACCAATCAGGACCTTGTTTATATTTACCTTTCTGTGGTTAAAAATAATCACTTGTTAGTTTAATTAAAATATGATATGTGTCCTGGAAGTATTGTCAGAGACTCTGTAGATAAGTGTAAAATATATACATTATGAGTCCACTTGAGTAATGTAGACTTTCAACTTCATACTTTAGGCAAAAAAGAAGTCAAAAACTATCTTCCAGAGGCAAAGATTATATTTTACAATAGAAATGCAAATGAACAATAAAAATGGTTATTTTCTATGTTATTAGCTGTTGTAGGGAGTCAGCTGTGGAAAGTGTAATAAAGAGGGCTTATGATTATTATTATTATTATTGAGTCTCGCTCTGTCACCCAGGTAGGAGTGCAGTGGCACTTTCTTGGCTCACTGCAACCTCTGCCTAGCGGGTTCAAGTGATTCTCCTGCCTCAGCCTCCCGAGTAGCTGGGATTAGAGGCACCTGCCACCATGCCTGGCAGATTTTTTGTATTTTTTAGTAGAGATGGGGTTTCACCATGTTAGCCAGGCTGGTCGCGAACTCCTGAACTCAGGTGATCCACCCACCTCGGCTTCCCAGAGTGCTGGGATTACAGGTGTGGGCCACCGTGCCGGCCTTGTTATTATGTTTTAATCAATATGAATGTATCTCTGATAGTAAAGAAACACTTTCTGAGCACCTGTTATCTACAGAGTGGAGTAGACGGCAGGGAGAAAAGAGGCACATTCTCTGTTCAAAGGACTCTAAGCTATTTGAGCATCCAAGATCCTTTTTGGCCCGAAGGGATCTCACCATTTACTTTTAGCATAAAAGTTTTGCAAATTTTAATGGTATGGTTTTATTTGTAAGCCTGAGCAGAAAGTTTAATCACACAGTGTATTCTGAAGGAGAATTAGAACTCAGAGTTGCAGACACAATTTCCATTGCTAATCATATGGATGTTTTGGGATATCAGATGGAAATATATACTAACATATTTAAGTGGAAAATGATTTAGTTCTTATAAAACCATACAACCAGGATCAAAGAAACAGAGACCTGTAACATCATTTATAGACCCAGGAGGGATATCTACCTTTCCTAAGATATGTGTGTCATGGTATTAAGAAATACCCTCAAATTTCCTCCTGTTCACTCTGAAAGTTTGACTATACCAAAATGACCTCCAAAACATCACTAAGTAATATATCATGCTGAACTAGTTGCTCTTCAGTACTCTTTCCTGTCTTTTTTTTTTTTTTTTTTTCAGTTAATAAGAAAAGGTCTCATATGTAGTACAGGAATTGGCAAGCTGCTTTTTGTAAAGTCCTGGTAGTAAATATTTAGATTTTCTGGGTCTATATTGTCTTTTGCAACTACTCAGCTCTGTGGTTGTAGCACAGAAACAGCCGAAGATAATGCATAAAGGAAAGAGAGTGGCTGTTCCATAAATGGAACTTTATGGACCCTAAAATTTGAATTTCATATAATTTTCACATGCCGTGAAATATTATTCTCCTTTTGATTGTTCCTCCCCCTATTAAAAACTGTAGGCCGGGCACGGTGGCTCACGCCTGTAATCCCAGCACTTTGGTAGGCCAAGGTGGGTGGATCACGAGGTCAGGAGCTCGAGACGAGCCTGGTCAATATGGTGAAACCCCGTCGCTACTAAAAATACAAAAATTAGCTGGGCAAGGTGGCACGCACCTGTAGTCCCAGCTACTTGGGAGGCTGAGGCAGAAGAATCGCTTGAACCTGGGAGGCAGAGGTTGCAGTGAGCCAAGATTGTGCCACTGCACTCCAGCCTGGGCAACAGAGAGAGACTTCATCTCAAAAAGAAAAAGAAAAAAAAATACATACATATATATATATATATATATATATATATATATATATATATATATAAACTATTCTTAGCTTGCTGTAGTTTGATAGTCTGTCTTATAGATAAATGCGTGGATCTCCTTTCATTGCTTCTTGAGGCTCCAATTTTGTTTGATAGGTGTACTTGGAAGTTAATATGTATATTCCAAGCTAGAAAGTTATTAAAAGATTTTAGGAAATCCTAAAGTGGTTTTTCAGGTGGCATTTCAGTATCTAATAAAAAGCAACACCAGCCTTGAACAGATATCTTTTTTGTCTGAGAAAGTGAGGCATGCCCATCCAGATATAAAATGTAGTTAACGACACAACCTCATTGTCAGATGAGGCTGGAATAGTTGACTTGATTCACGTCACATATTCTTATTGAAGATGTGCTATGTGAAAGCATTAAGGATAGAGGTGAGAAAGATGTAGCTCCTGCCTTCCCATGGTCTACAATTTAATGGTGAATTTCTAACCTTAACACTCTGAGTTTGAAGAAAGCTCTGGAAATTTATCCTCAGCATTGTGCAAGTTAATAGGACAATTTTAAACAGTTTCCATGGATCATATGAGAAAAAGAATAGTTTCATGTCATTGTTTTAGAAACTTGACAAGCCAGGAGGATTGACATCATTCAGTTCAGGGGCATATGCTAAATGTATTTGTTTTTATGGCATACATGAAAATACATAACCCTCTTTTTCTATACGATGAGTATCCAGTGCTTATTGTCTGACCGGCTCACATTGCTCTAAAGGGAATTTTTTATTCATATTTCATAAAATAATCTGTGTCCCCAGAGAAGAAAACACATGTTAATATTGTGTTATTTTTAAAAGCACACAAGATTTTAGGGACATATTTACACTTTTTTGCATGTGTGGCCACAGATAGATAGTAAAGAACTAGTAGCTTCATAGTTCCCTGGTGTGTTCATTTAACTGTATTATATGCATGCTTTTGAGTTGGTATCTCACAGCTGTGATAACTTGTGCATGGAAATCTAACAGACTGCAAAATTAAATCTTTGCAAAATATAGACATTTGGTCTAATTATGTCTTTGTAACATTATATATACATGGGTCTCCAGTGATTGATATGCATCTATATAAATCCTTCAGTTGAATTCTTCAGCTGAGTTTTTTTAGCTCTTATTTTCATGCAAATCTCCAAGAAAGAGCATGAATGACTAAACAAAGAAAAACAAAAATCTAATTTCCTGGCAATCATTTGAAAAGAATAAATCAGCTTGGAAAATACCTATAATAGCCTGTGATTTTCTTTCCAAAAAGATAGTACCAATTTTATGCTGGTAAGGAATCAATTTTGTATTTTTCTTTATAGTAGAGGGAAAATTAGTGGGAAGAGAACGTACTTTCTGTAAGGTGGCACTACAATATTTTTACTTTAATTACTTCTATTAGGTTCCCACTATGTGTTTTCTTCTGCCTGCTTTTCATTTGTGCATGGTTTTTCTTTTTAAAAAAATTTCTTCGCGTTTACCTTCATGATCATAGGCTTTATCGAAGATAGACTTTACAGAGCTGCTGTGAATCAAATTGTTATAGATACAAGTAGGCTTTATATTACGAAGAAATGTGATTTTAGTTGATTTGCTTACATTGGAATAAATATAGAGAGTTATGTTTCTAGATGCCAGAATCTAATAGAAAGACAAATCGGACCCTATCTTTAGACTTCTTGCCATCCTATTATCAAGGGACATCAACGCTTAAGACAGCTTTGCAGAAGCCTCTTGAGACCTATTTAGCAACGTTTTGACTTCTGTACTTCAGATCAGTACTATTTCTTTTTTCATTTGTTAGTTGTTTTCCCTTATTCTTGGCTTCCAACATAAAAGCGAGGCTGACATCACTTTGTTACAGTCTCGCTTAGGCTAGCTCTTACTTTCCTCTGGAAATTTATTTTTGAGCTTCTACAGGATGAATTTTATTTAAATGATAAAACAGATCTGTGACATCCTGATGAGCCCTTAGTGTTTCCTTCTCTTTTATGATTACTTGACAATTGTGGCTCATTAGTAACACAAGTCAGAGATTATTAGAACAAAGCAGGATCTCCAAGTCCATGGTTCCAGCCCCTCACACCATAGCCGGATAAACTGAGGCTCTGTGAATTTATAGCCAGTTTATAATAAAGCTAAGACTAGAATCAGATTTAGAACATAGAGCTCTTTGTAATGTTGTGTTCTGCTACATTGGCAGTTAAGTAAGTAGTTAATTATAAAATTTAAACATCTATTTCCATTTTTCTGTCTCTTTTTTGTAGGATTGTTTTAGAGATGGGGTCTAGCTATTTTGCCCAGGCTGGACTTGGACTTCTGGGCTCAAGCGATCCCCTGCCTCAGCCTCCCGAGTAGCTGTGGACCACAGGCACACCCCACTGTGCCTGGCACGTTTTTCTGACTCTGTTGTTAGTTACTTGGTATTTATTCTTGTAGGTTCACTTTAAAGTCCTGTATCTTTAAACTTGTTTGGCGATGGTTATCATCAAGAATATTGATATTTTTTTCTCAGATTCATGTATCTGAGGGAGAAGCAGGCAGTATTTTCAGTATTATCAATGTAGAAAAGAGAAAGGTACACGGTCTCCAAACTGATAGAAAATGTCAACACCGTATTTCTAGGTTAGACAGAGAAATCCTCCACGTTTCCAGAATATCTGAAATTGCCAGCTTTATAGGTTAAAAAGAGATGAATATTGATAATTTCATATGATTCACCCTTATAGTTATTATGGAACTACACAAGCTTTTACAGTTTCCTTTTTTTTTTTTTTTTTTTTTGAGACAGAGTCTTGCTCTGTCACCCAGGCTAGAGTGCAGTGGCATGATCTCGGCTCACTGAAACATTTGCCTCCTGGGTTCGAACAACTCTCCTGCCTCAGCCTCCTGCGTAGCTGGGATTACAGATGCCTGCCACCATGCCCGGCTAATTTTTGTATTTTGATATATATATATAATTTTTTTTTTCTTTTTTTTTTTTTTTAGTAGAGACGGGATTTCACCATGTTGGCCAGGCTGGTCTCAAACCCCTGACGTTGTGATCCCCCCGACCTTGGCCTCCCACAGTGCTGGGATTACAGGTGTGAGCCACCGCGCCCAGCCTACAGTTTACTTTTGAATTTCATAGTTATGAAGTTTTCAGGGCAGAGATTTATTATCTCTGAATTGGTAATGAAGAAAAACCATAGAGACGGGGTTTCACCATGTTGGCCAGGCTGGTCTCAAACCCCTGACCTTGTGATCCCCCTGACCTTGGCCTCCCACAGTGCTGGGATTACAGGTGTGAGCCACCGCGCCCAGCCTACAGTTTACTTTTGAATTTCATAGTTATGAAGTTTTCAGGGCAGAGATTTATTATCTCTGAATTGGTAATGAAGAAAAAACTCAATGATTAATTAAACTCAGATAGATTAAATTACTTTTGTAACTTCTCTCACCTCATTGCAGAATCAGGCTCAGTCCTAGGTCTTAGAGGTCTTTAGTTAGCTGTACATTGCTTCCCCATCCATTTTTATTCGGGCATATTCTGTTAAGATGATATCAATTCTTGCCGGTGACATGGTTTACTGAATCCTAGCATTAAGCAAATAAAAATGACTTTGTTCCTTTGAAAGGCATAGCCAAATGTGATTCAAGAAGCCAATCTGGGAAACAATTTCCAAACAGTTCATGAGATTGCCCAACTTTCAGCAAAATAAAAGGGTTTATTTCTGGCATCCTCGTGGTGTAAATAATTTCTTAATTATGCAAAAAATTGCTTTCTTGGGTTTGATATATGCATTTATAGGCCGAGTTTTTTACTCAAAGGATTAAGGTATTTATCCATCTCAATCTGGGAATATCCAAAATACTCATTAAACAGTTCTTTGTAAATGCCTGATGCCACAGGCCCCATTCTGATAATATCATATTTCTCTATACCTCACACTTAGGCTAGACAAGAACTGTAATGAGGGATAATAATTGACCAGATGACATTTTACAAAAGATTCAAGTTTCTTCTTCTTCATTCCTTTTTTTTAATTGAGTCCAGTAAACCGTATGCGAAGCACTGTGTTAAATACCTTGATCTACCTTATCTCAGTGGTCCTCGACTGAATCGCCTTGTGTAACTTATTAAAAATATGTTTTCCTGGATGCCAGCTTGTGTTGGAAGGGAGCCAGGAAAATGCATTTTTATAAGTTTCACAAGTGATTCTGATGTTTATTTCTGGTTAACTACTATAGCCTTATCCCATTTCTTCCTCATAAAAATCTTGAGAAGTCTCAGTAATTGTAAAAGAAGATAAATTGGAAGAACAAAAATAGCTTTTCAGGCACTCTATTCTGTCTATACTATGATCATGAAGGTATAAGGAGTGTGTTTCTGAATTCAATTGATTTGAATAGATGAAGTCCTAGGCCTTCATTTTTTCTTTTCTCTGGTTCCTCTCTGCAGTCTCCTTTCAGCCTGCTTCAGGAATGAGCTTGTGGAGCCCCGGAGAGAAACTCCAAAACAATCTGACGTCTTCTTTAGACATTCCAAGCCCCCAAACCGATCAGTGTACCCATAGAGCCCTATCTCTATATTTTAAGTGTGTGTGTTGTATTTCCATGTGTATATGTGAGTGTGTGTGTGTGTATGTGTGTGCGTGTGTATCTAGCCCTCATAAACAGGACTTGAAGACACTTTGGCTCAGAGACCCAACTGCTCAAAGGCACAAAGCCACTAGTGAGAGAATCTTTTGAAGGGACTCAAACCTTTACAAGAAAGGATGTTTTCTGCAGATTTTGTATCCTTAGACCGGCCATTGGTGGGTGAGGAACCACTGTGTTTGTCTGTGAGCTTTCTGTTGTTTCCTGGGAGGGAGGGGTCAGGTGGGGAAAGGGGCATTAAGATGTTTATTGGAACCCTTTTCTGTCTTCTTCTGTTGTTTTTCTAAAATTCACAGGGAAGCTTTTGAGCAGGTCTCAAACTTAAGATGTCTTTTTAAGAAAAGGAGAAAAAAGTTGTTATTGTCTGTGCATAAGTAAGTTGTAGGTGACTGAGAGACTCAGTCAGACCCTTTTAATGCTGGTCATGTAATAATATTGCAAGTAGTAAGAAACGAAGGTGTCAAGTGTACTGCTGGGCAGCGAGGTGATCATTACCAAAAGTAATCAACTTTGTGGGTGGAGAGTTCTTTGTGAGAACTTGCATTATTTGTGTCCTCCCCTCATGTGTAGGTAGAACATTTCTTAATGCTGTGTACCTGCCTCTGCCACTGTATGTTGGCATCTGTTATGCTAAAGTTTTTCTTGTACATGAAACCCTGGAAGACCTACTACAAAAAAACTGTTGTTTGGCCCCCATAGCAGGTGAACTCATTTTGTGCTTTTAATAGAAAGACAAATCCACCCCAGTAATATTGCCCTTACGTAGTTGTTTACCATTATTCAAAGCTCAAAATAGAATTTGAAGCCCTCTCACAAAATCTGTGATTAATTTGCTTAATTAGAGCTTCTATCCCTCAAGCCTACCTACCATAAAACCAGCCATATTACTGATACTGTTCAGTGCATTTAGCCAGGAGACTTACGTTTTGAGTAAGTGAGATCCAAGCAGACGTGTTAAAATCAGCACTCCTGGACTGGAAATTAAAGATTGAAAGGGTAGACTACTTTTCTTTTTTTTACTCAAAAGTTTAGAGAATCTCTGTTTCTTTCCATTTTAAAAACATATTTTAAGATAATAGCATAAAGACTTTAAAAATGTTCCTCCCCTCCATCTTCCCACACCCAGTCACCAGCACTGTATTTTCTGTCACCAAGACAATGATTTCTTGTTATTGAGGCTGTTGCTTTTGTGGATGTGTGATTTTAATTTTCAATAAACTTTTGCATCTTGGTTTATCTTGCAGTTTTTTTGTTTCTGTCTCTCTCACTTTTTTTCCACTAAATACTAGAATATTCTCATGTAAGTGCTTGAAGTAGCATGTGTAGGTCTAAGGAACAAGAATAATAATAGAATCATCACTTTGTTCCTACGTACATTTCTTCTGTCAGTATTTGGTATTTTTATTTAAGATACTCTGTATCAAGGTCATCAATCGTAACAAGGAACTAAAATGCCTAAACTAACTTAAAAAAATACTTTAGCTCTATGAGTTTTAAAGTTTGTCCTAGGAAGCCAGACACCATAGAATCATTTATTGACATTCCTGACAAACCTCTAGTTGTATGTTAATTCGCCAAATTTACTAAAGTGAATTTCAATCAAATCCAAATGTTTTAAATAACATGGCTTCTGTGGCATAGACATTAGCAAATATTTGGCCAGAAATCAACAGTGGAACCTATTGTCCAATTTTTTTTTTTACTTATGTTTTGAGCCCTGAATAATTGCTTTTAAAAGCTTCATAAGAAGTTCTAACCAAAAAAAAAAAATTAGTATTTTAGATAAATATTTCAGAAAAAAAAATCTTTATATTTGAGATAAAAGTATATTAGTAAAACCAGATGCTCCTGTTATATTTGCAAGGTGTTTTTCAATTGGTCTAATAACTTTGATTCACAAAGCCCTTAAAGGATTGGTGAGTATGTTTCGTTTTTTGATATTTGCTTTTCTTTCAGAAGGGTTTTTCTTTTCTTTCAGCCTTTTTTAAGTAAGTTTTTAATGTGTTTATAGTGACTTGTATATAAATGCACATATTTGAAATGATGGAAAATATTAGAAAATTACAGTTTTCCCATCTAGAGAATAAATGCTGCACTGTCTTTAGTAGCCAGATAACCCGGCTAGAGAGAAAGGTCACAGGTAGGCAGTACTGGGTAAAATTGAGAGAAGATATGATTCTTTTTCTGATGGTCCATGTTTTCCCTTTTTTTTTTTTTTTTCCTGACCTTTCTCATAACAAGGATGCTCTGCTCCGCCCTCAGTATCCCTTTCTGCTCCCAAATTCTTTACTGCAGAAATGGTTTGTTGAAATAGATTTGGCCTAAGCTATCACCAAATCCTACTAGAGGGTTGCTTTTTAATGAGCAGTGGAAGATTTTAGCCAAAAGAAGGAATCTAGTGGAATTCTAGACATCTAGGAGGTAGATAGTGTAACTTGTGTCTGCGCAGGAATTTTGATATTCTACCACATAGATAGTCACTAGGGTTGGAACCAGAAAACCTGATTTGAAGTCCTATATGTCCTAAAACCACTTCTCGCTTTTCAATTTTGAATTTTATTCTTCGGAAACTGTGCAGAAGCTTATATTGTTAAAGTTAAGAACTGGGACTTGGCTCCACCAGGATTTTTCTTCAAAGCTGGAAATACGTGAAATATGATTTTCTACATCTCTGTTACACTGAATTCTTAGAAAAATCTAGTGAGGCTCTAGAGAAAACAACCTGAGGTATACACCTTACGATTTTTTTCTGCAGACAGCTGGATTCTTACTTTTTTGGGTTTTGGATAGGAGAGGAGAGTGTAGGCAAGAAAACCAGAATGGTACAGGTCCTGTTATTCTTCTGAACACATTTTCCTTCTCTTTAGTTTCTCCAGAAATTTTTGGAATATTTCACTTAAGAACTATTGTATTAAATTTGTAATTTTTTTTAAAAAAGGTTGTTCTTCAAGGCTTTCAAATTGGCTGAGTTTATTCAGCCTTGCTTCCTTTTTCACCGTATGTAGTTTTCAGAATGCTTCTTCTAGTTCAGTCATTTTGAAATTAATAATAGACAGACTACCTTGCTTCCCTTCCGTGTGGCTTTAAAACTCACTTTCATATTAAAGGGAAGGCATTCCGAATTTCTGACCTTTCCTGACTGTTAACCCCATTCAACTGTTCACCCTCATTTCTGGGTATGTTCAATTAAGAATCCAACTCACCTCTGGCCACTTCCTCCTATTAATTCTTACATAGTTATCTTCTATGCCATGGGCCACATATCAGAGTGTTAAATATTTGAGGCTGGAGGGAAACAGTGAAAGAAGCTAAGAAGCTGATGTTTCCTGAGCACCTACAAATCACTGGACCCTGTCCAGGGCTGTTACATGATCTCATTCTTAATAGAAATCTTCTAGAGTAGGTACAATATCTAGGCTACAGGTTAAGAAAATGAGACTCAAGAAACTTGTACAACATCCTGCGTCACTAAGTTTGTGTTAGATGTTTTGATTGACTTAGAATCCATGTTCCTTCTTTCTTCATCTGTGGTTTCTGACCTACCCTCCTCAAATTAGTGGCTTATGATGAAAAATGGTAAATGAACTAATAAAATAGTCAAAAATAATAACCATAAGCTAAAGAAAGCAGGAGGTTGTAACAGGAATTGTAAGAAGGAATTTACTCTGAGCGTCCAGAAAAAAAAAAAAGCAAAACTTTTAAATGTATTTTTATTTTTCTAATTGTGGCTATTAATGATTCAAGCACTGCTTTCTATTAGCTCTTGCTGTACATTTGACTGGTTTGAGGAGGGACAGATAGAGAGGTCTCCTCAGGTTACAGAGGACCAACTGCATCAGGAATATGGGCTACCTGGAATCTTAGGTACTTTTCAGTGAGCAAAGAGAATTGAAGAAACATCAGCTGGCAGAGAATCTCCTTACTGCCTTGCCCCAAGCTTTCCTATACTCTGATGTGGACTAATGGATGAGCCTAAGTGCAGCCATATGTGCTGACTCTTTGAAAAGGTTCCCCCACAGGGACGGAGTGGATTCAGAAGTGCTTGGTAGTAGCTGGAGTTACCTCCTTTACCCCTCTTCCATCTTGGTCTCCTACATTTCTCTTACTACTTCATCTATTTCTTGTCCCCATAACCTAGAAAAAACAATAGGTGCCAGGAGACCTAGTTCTGCCCTCAGTGCATCCATTTAGCAACATGTGATCTTGGGCATATGTTTTTTCTTAGAACCTTATATTTACCCATATGTATATTTGGAATAGTAATTTCTGTCCATCTCTGCCCTGCTATACCATGACCTGCTCCACAAGGATGGTGTGACAATCATTCAGAAAATGTACAAAGAAGAGTTCGGTAACCTGAAATGTTTTATAGTTGGGAGGATTTATTGTATCAACACCAGTATATATATACAATGTGGATCTATAATTGTGAGTATTTGATAAGGGCTAAAGAGAAAAGTCAATGCCCTCCTACCTTAAAGGCAGTGATTGTAAAATACACCTGAATTGTTAATTTACATTAATTGACATGATAATTTATACACATTAAAATACAGCATATTGTTTGCATTCATCTAGCTGTGGCTCTCCATTGACATGGAATGGAGCCTTCCTTATTTTCATGTAATTATGGAGAGAATGCAGAGTGGCAGCTTCGAAGACCATCCCTACGATTGCAGCACCTAAGTGCTAGGGCCATTTTTAAAATGATTTTTTTCAAATATTATGCCATATAGGCTTGGAACCTGGGTCCATTTACAGAGCCTCCAAAATTTGAATTATACATATATATGCAGATAAAACTCAAAGAAAAAATTTGTCTCCCCTCTAATGTAAATATGCCTGTGACAGGGAAAAGGTAAACAGGAACAACATAATTCGCTTTGTTTGAGAATTGTTAAAGGGGAGTTTTCCCATAACAGACTAGACACTTGCTTAAAGTCCAGTCTTCATTCTGTGGTCACAGTAATTCAGTGTAGATGCTGCATAGTCTGTACATTTCTTCCTCATCTTCTCTTTTTCAGTTCTCATCATAGCCGTATAAGGTAGGATGGATTATTATATCCATTGCCTCCACTACTCTATGAGTGTCTCAAAGATGGGGACCATGCCTTAAGTTTTATTAACTTGAACATCTGACTCATGCAGCAAGTGTAAATGTATGTAGTTTATATAGTTTTCTTGTGAGAAAACAAAGGCCAGGATGTTAAGTAGCTCGCATGGGCATCTATAGCATCCTAACAGATGACGGAGGAAGGCTTCATCTCTGACTTTGTGACTCTCATTGTAGTACACATCACTAGTTTTTATATTAAGATAAAACATCAGGAATGACAGTTGAGTTGGGAATATTATCTGTTAGATATAATATGACAAATGAGTTCTGGTAACTTTCCAAATTGAAGATCATGCTGAATGGAAAACTATTGGGTTTTTCTGTTGCTTTGTTTTTTTCATTTCTCCCAGGAGACTTTTGGAAAGTCAATAGAATAGTTATTTGGGCTTCAGCGTTCTCCTGTGTAGAAATGCTTTTGACATTCCAGTAGGTTCTCTCATACATCATGAACATCTCTATTGAAAATGCATGTTTCCAAGTAGGGGCCTCTGCATGACAGCAAGATGAGCTGCCAAATGCCCAGTTTGATTGGGTTTGTAGTCATTTTCTGCATATCCCAAATGGAATATACAGAAAGCTACAAATGGAATGCCTTAGCTGCAGTTTGAGGGTGGGATATCCATGTTTTTCGTCAGCCTTTAAGTCTTTGCTCAATTCTTTTCCCTAACTGCTTTTCAAACTCATTTGAGTTGAAGGGAAAAGAGGAGAAGGGAATGGAGAAAACAACTGAAGGGAAAAGAGGAGGCAAGAATTTTCTCTTTCTCAAAGGAAATACTAAACCTTGCACTCTGCACATTCAGGAAAAATATGATTTGATGATAGCCTCGCAGTCAGTTTCTCCCTATTCAGGAAATATTCTCAGATGGAAACAAAATTAACTTCTTACCATTAATCCTAGAAGAATGTTTTCAAATGTTTGGTTTGAGGCCATGTTTTAAACAGAGACCTGTTGAAAATCAATAGTCCCCACTCTTCAGTGTCCCTTGCTCACCATTATTTCCATGTTTGTCTTCCTAGGACCTCAATACAGTCTCCATCTTCATATGGTAACAGCTCCCCACCTCTGAACAAAATGAACAGCATGAACAAGCTGCCTTCTGTGAGCCAGCTTATCAACCCTCAGCAGCGCAACGCCCTCACTCCTACAACCATTCCTGATGGCATGGGAGCCAACAGTAAGAGCATCTCCTTTTAGCTGTGGCTGAAGGATGAACAGGCTAGCTTAGGACAAGACTCTGTGATGGGGAAGGCATGTTCTTAAGCTAAATGAGAGACACAGTGGGACAGATCAGATCAAAGTGGAGTGGCTTGGGTTTCTGACTGAACCTTTCACTTTTGAGGTTTATCATTCATTGCAAAATATACTACTGGTAGAGATAGGGACGGATGTTTCTTTAGTGTAGGGATAAGGATTTCTGTGAAAAGAACAGTCTACTTGCTGCTATCCTGGATGGAGACTCACCACCTGCAGCCTTTGAAATAGTCAAAAGGAAACAAATGCAACATGCATAAAGTAGCATGTTGCAGTGAACTTAAAATCTAGAGAAATAGGCCGGGCATGGTGGCTCACGACTGCAATCCTAGCACTTTGGGAGGCCGAGGCGGGAGGATCACTTGAGGTCAGGAGTTCGAGACCAACCTGGCCAACATGGTGAAACCCCGTCTCTATTAAAAATACAAAAGTTAGCTGAGCCTGGTGGTGCATGCCTGTAATCCCAGCTACTCAGGAGGCTGAGGCATGAAAATCACTTGAACCCGGGAGGCAGAGGTTGCAGTAAGGTGAGATCACACTGCTGCACTCCAGCCTGGGTGACAGAGTGAGACTCAGTCTCAAAAAAAAAAAAAAAAATCTAGAAAAGTAGATTATCGTCTGGACTCTATGAAAAAAATACCTCTAATTACAAAGAAGTTACTTCTCGAATACTTAATTTTCTATCCATAAAATTGTCACTATATATACTTAACATTTTATGTCTGTTACTCTATAATCCTGGCCTTTTAAACTCACCTATTCTATCCCAGACACAGTCAGTAAGGAACTGATCTACGAAGGAACCTGCCTTTTATTTCTTCTCACACACATTCCTCATATCAGCTAAGTACAAATGAAAAAGGCACTGTAAGAATTGGGCCTCCAAGTGGATGATGACTGGCATATAGCTTTACTATAAATATAATTATCTTCTCCACTAGGACTAAATCTAGCAAAGTGGATACACAAACTTGAAGGAAAAAAAAGTCGTGGTAAAAATGCAAAAGATTGTATAACACCCTCGGGGATACAGAAGACTGAACATTGCATGGGTATCATATAATCCTTACTACCTAAAAAACAGTACCCTCAAGCTTGTCTTTTGAAATCCTAATTATTTTGATCAAACCTCTAAACGAGCATTTCAGTGTTGTCTCTTCTGAAAGAAATCAGGGGAGGCTAGGATTATGACTTTCTGTGGTCTAATGGCACACATTCATTGTTTTGGTTTTTTTTTTTTTTTTTTAGACAGAGTTTCGCTCTTGTTGCCCAGGCTGGAGTGCAATGGCGCAATCTCGGCTCACTGTAATCTCTGCCTCCCAGGTTCAAGTGATTCTCCTGCCTCAGCCTCCCGAATAGCTGGGATTACAGGCGTGCACCACCACGCCCGGCTAATTTTTTGTATTTTTAGTAGAGGTGGGTTTTCACCATGTTGGCCAGACTGGTCGCGAACTCCTGACCTCAGGTGATCCACCCACCTCAGCCTCCCAAAGTGCTGGGATTACAGGCGTGAGCCACCGCGCCTGGCCACACATTCATATTTTAATGAGATAGAGGTGTGTGTGTTTGCAATGGTTGCCTGTTATGGCAAACGTAGACTTTAGAGTGAGATCACTCTCTCACTAATGATAAACTCATCATAAGGCTTAAAACCTGACCCCTTGACAGGCCTACTTTGACATAAAAAGAGTAGAAACCATTTATTTTCAACTTTATTTTATTTCTTGTGACATCTTAGAGTCCTCAAGTTCCTTCTTGGTATGACAAAATTGTGATAATAGTGATGATGGTTCACATTTGTATAACTTACAGTTTGCCTACTTTTTTTCTTATTTGTACTTTACAATTATTTCAGAAGGGAAACAGATTGAGATGTGATAGGGTATGCTCTTTAGTTAGCTAGTTAGTGGCAAAGACCTTTTCTAGGCATACTGCTACTCACTAAAGTAGAAACAAATTACATTAATCTTTTAGTAAATTATTTTGTTCTTTTTGACATGAAGGCAAAATTCATTTTATAAATATTGATATTAAACTTTTTTTCTCTATCAGTGAGTGTTTACACTTAATTGTTTAGTAAAATATCAGTAAGTATTGGTGGTGTTCCTGTATACAACCCTGAAAATGGACCATAAGTAATAGAATATTTAATTGTATCATCATTTATTAGGCACGTGAGTCCTTCTAAATATTGAAGTCAGTCTCCCTATTCCCAAATAAATGTTCCAAGGGTCACTCTTTCATTCACAGATTTGTGCATTTATCCACTTACCCTAAGTTTACTGAGCACCTACTCTGTGCCAGGCACTGTGCTAGGGACTGTAGGGATCTAGACACCTATAAGATCTAATCTCTGTGCTTGCACAGGTTAGTGAGGGAGACAGAAATATCACCCTCGAAGGCAAATACAAGGAAGAATATGTTAACATTAAGAGCAATTAGCAATTAGAGAATTAGAAGCAGAGAAAGGGGAGGAAAGAGATTGTTCTCGGACAAGGTACAAAAGTCAAGAGGTGCCTAATTAGTTTGGAGTCTGACCGTTCTGTTTGACTAGATGTAGGCTGTTTGAAGGGGTGTAGTGAGAGCGAAGATTAAAAAGGAAGGCTGGTAGTTTAGGCCCTTGATAAAATTTAACCAGACAAGATGGACCACTGGGATGCTGGTACATGATGATGGCAGTAACCCTTTTTGTTCCTCCTGCTTCTGTTCAGTTCCCATGATGGGCACCCACATGCCAATGGCTGGAGACATGAATGGACTCAGCCCCACCCAGGCACTCCCTCCCCCACTCTCCATGCCATCCACCTCCCACTGCACACCCCCACCTCCGTATCCCACAGATTGCAGCATTGTCAGGTGAGTCCACAGCATGTGCCCCTGGGGGCCTGCCCTAAGCATCCCGGGATGGTGGAGGGCGGATACTGTTATAGTCCATAAAACAGTTGGAAGGGAAGACAGCAGTCCTGTGGTTGGAGTTCAGTCACTATTATCTCTGATCTGTGGAGTGGTCAACAATGTTTTATCAAGGAAAACTACTATAACCAGCATGGAAACAAGGGAAAGTGTCTGTTGATAGGCATATTGTTTTCATGTCTGTTTCCCTCGCCTTCACTTACTCCCTTCCTCCTCTCTCACATTTTTACATTGTCAATTGCGGCCCTCAAGTTGGTGCTCAGGGCTGGGTTTCCAAAAGGGCAATAAACTTGCTATCTCGCTCCTTTTCCTCTCTCTCTAATCCTTATAGCTCTCAGCCACCATGGGACCATTATTTTGGAGTTGGATGTCCATAAAAAGTTTTAATGTTATCCAAAATGACAGGGACTTTCAAAAACTGATGGCCTTTACTACTCTGCCTATGGGAAATGGGCACACTCCAGAAAAGGGGAGTTGAGATAAGCAGAAGAAAGAAATGCATGAAGCCTGAAAGATTTGCTTCCCACAGAGGCAAGAGTAGGTTCCCAGATTTCTTGCCTAGTGTATATCTTCTTCTTCTGCTTCATGTGCAGAATGATTGTTTCATTTGGCTACTGGAACGATTTGTGTTGATTTTCCTGCTACGTCAATCACAGGGATGGCAAAGTGCGTGGCCTTTTCCACCCAGGGTCTGTTCTGTAATGATGGTGGCATCATCATCAGTCTCAGACCAGCACCAAAAGGAAGGGGATCTGTATCCTGAGAAGGACATAAATCAAAAGGCATGAATGACTTAATGGAGTAGAGGCCTACAATTTTTTTCTTAACCACATGAGCCATTCTCACAAAGCCCAGATACAAAACAAATAAAGTAGAGATGTTCTTTTTGAAGTGTAGAGCCAAGCAGCAAGAAAGCTAGGAACCTGAACACTGGACTTCCCTTTCACCCTTTGCAATGGTTCCTAGGACACTTACACAGAACCCTAGAATTCCACAGATCACCATATGATAATCGCTGATCTGCAGGAATCCTTATAGTTGATTTTAATTTCTGTAGACCTAAACTTCATGTCACCAGTAATCTCCAGACCTCAGACTTAAGGCCCACATATATATTACCCAATCCTCATCTCTGATGTGGGGCATCCAAGGGCAAAATATATTGGGTTTTCCCTTATCTCGCCAATGCAGTTGGGGTGAACTTTCTTTTTCTGTTTCCTCCTTCCTCTTCCCTCCTCCCTCTGCAGTTTCTTAGCGAGGTTGGGCTGTTCATCATGTCTGGACTATTTCACGACCCAGGGGCTGACCACCATCTATCAGATTGAGCATTACTCCATGGATGTAAGTAACTGTTAGACTTTTCTCAAATTTTATTTCTTCATTTCTTTCCTCTGATGACAACCGCCTTGTAGTTCAATCCCTGATAGTTTAAAAATTTGTTTTTGTCATGCCCCCAATTATCCATTTTGATAGAACCCATAACTATGTTAGAAAGATTCCCAGGCCTCAACCTATACATATTAGAAGCTTAGCAATCAATCTTCCTTAGCTTGTCTTATCAGTCAACAATTGTCTATTGCACTTACACTATTCTCAAGGTGGGAAAGAGGAGCTATTCCCGCAATCAAAACATTCATAATTTCTAAAGAATTAATACAACTTTTTGAGTGTGCTCATTAATCTGTATCAGTTCCCAGAAAGGATAGCAGGGCACCGGTGGTGCTATTCTCTTAAAAACATGGAGAGATGACACAGTAGTCATTTGCCATGACTATTTAAAACAAAACTAGGAATAGAACACAAACGTGTAAGCTTACAGTCTTTAACTTTATTAACTGGCCTTTTCTGCCTTTCTTTAAATTAGTACCTTAAGGGAGATTCAGCCGTTCCTCTCAATTAAAACTATATGTTCCTTTATTAATTTTCAGTTTCATCCTAATAGAGCCCAGCCATTTCTAATCATTGGGCATTATCAATGGCTATGGGGTCAAATCCCAATCACCCTTTTTAGTAGCAATGAGAACTTGGGGTTATCACTTTGCCTCTCTGGGCCTCAGTTAATAAAATATTGGGTTCAAATTACTCCTTTCTGGCTTTCATATCCTTTGATTTGTCTTTGAAACAAGCTGGGCGAGAGGGGACTTTTTCTTGCTTCCTACTCCACTTTTTGAACTCCTGCTCACACGGGAGCTAGTGTTGCTCTGGTTTATGGCAATCTGATTTGTCTCTTAGGCCGCGCTCTTCATGCTGCTCTCTTTGGGGTTCACTAGCCTCTCTTCTAGCTTCCAGAGGGTACTTTGGACTGCGGGAAGAAGACAGTCTCTGCCTCTGCTGCTACTTCTATTGTTGGGGACTCTTTTTTCCCCCTCCTTGAGCACCGATGCTAGAGATGCCCCTGCAGCATAAGAATGCCCCCACCATGAGGTGACGTGTACATAGTGTACTGCCCCTATGCCCACCCCTCTTCACCCCATAAATCACTCCTGGCTGCTGCTCCCTGATAGAATAAGTTATAAGCAAAAACTGCAGGGAAAGCTTTAAGAGTAAGTTCCAGTATTATTCTGATAACTTTACAGTCACTTCCGTCCACTGAAGAGAACAGAGTTGCCCTATATTTGAGAGAGTCTCCTGTAGTAGATTCCTACATTTTGGAACAGAGAATCTTCCTCTTCCCTATATTTTTAAAGTTATACTTTCCACTTGCTCACTTATTTCCACTGGGGAATGTGTCAGGATGTTCCCTTGATCATTAGGATCTGCAGATACCCTTTATAAGTTCATTTCTGCTGATGTTGACTAAGTCCTATGCCAATCTGTGGGCCACAGCTTTCTCACCTGTCAGTCATTAGTTAAAATCCATCCAGGGCATCATGACAATGAACAGAGTCTAATTTACAAAGAGCTTTGCATTTACTGAGAGCCTCAAAAGTATAAAGAATCCAAGAAACATAAAAAGCTATGCTTATTTAGGCCGGGCATGGTGGCTCACACCTGTAATCCCAGCACTTTGGGAGGCCGAGGCAGGCGGATCACGAGGTCAGGAGACCGAGACCATCCTGGCTAACACGGTGAAACCCTGTCTCTACTAAAAATACAAAAAATTAGCCAGGTGTGGTGGTGCACCTCTGTAGTCCCAGCTACTCAGGAGGCCGAGGCAGGAAAATGGCGTGAACTTGGGAGGCGGAGTTTGCAGTGAGCCGAGATCACGCCACTGCACTCCAGCCTGGGTGAAAGTGCAAGACTCCGTCTCAAAAAATAATAAAAAAAGCTATGCTTATTTTTTTGTGGATTACCTTCTTTATAAGGTATTCTTCTGCTACAGTAAGAGATTAGGAATACCTCTAATGGCCTGAAAATTCACACCATAGGAAAATATGACACCAGACTACAAACTTCCAAATTGAGAAATGTTGGGAAGCTGTGCAATTTTCTCCTCCTCACCTCAGCTTCTTCCTGCGTATTTCTAGAAAGAGACACCCTTTTCTCAGCTTTTATCTTAGAGCCAGTCTAAGTTATTGGGCATCATCACTTCCTGGAAAAGGAATGTGACCTAAATTTGGGGAACTTCATTAGTATCCCCATAACATGCAAAAATGAGGTAACAATACAACATAATAATAATACAAATGGTTAATATTTATAGAGTGTGTTGTAATTTATAAAATACTTTCATAGCTGTATTTACCTATGAGTCTATAGCATCTTTTGCCCTATAAAGCAATTGTATCTCATTTTACCAAAGAAGAAACTGAGGCTGGTAGAGAATAAGTGACTTGCTGAAGTTCTATAGCCAGTCAGTGACCATCAAAACCTCTGCCACAGTTTTCTCACTCCAAGCACAATGTGCTGTTTACCTACCATTCTGCCCTGTGAAAATATGAGCTATCACTGAAACAATCTAAAGAAGAGCTAACGCAGATAAATGTAAGTGATATCACCACAGTCAGTTAAGGTTGACCCTATTTGCAATGCCTTCTGCACCCATTCACAGAACATAGACACAGTTGCTCTGCATTTTGATACCTTGCTTTATTCAAACACCAAGAGACCTTTTAGATAAAAGTGCCGTTTCAGGTTCTGAGGATGCCCTAAGTCCCTAATTTTTTCACTTAGTTATGTGACTTTAAACTTGTCAACCTCTCTGGCCTTCAGTTGTTCTATCTATAAAACAGGTCTAATAATGTTTACCACTAATCTGGGGATTAAATGATATCATGAATATAAAATAATTCAGGGCCTGGCACATAGAAAGTACTCAATCTTTTCTTCTCATCTCCTCAACCAAGTGGTGGTGACATTCCATTAATACCTTTCTTCTAGTGACTAGCCAGGTAAATTCAAGCATAAGTAGGTACCTCATGTTTCTATTTGGGATTTTTGCCCTCTCATCTAGCTATTATCCCAATTTTACCAATGAAGAAACTGAGGCCAGTAGAGACTAAGTGAAGTGTTCTACACAGGCAGGAAAGACACCTCAAGGCTGTGCCTTTGTGCTTAGTTCCATAGAGTTGAAGACTCAGAGAACTAATTTTATTTTCTAATTTGTGGATCAATAGATTCAGATCAATTAAACCAGAGCATCAGGGAATGATAGGATGCTGTGGACTAAATGTCCGTTTTTCTCCCTGTTTTCATTCTCCATGACACCTTCCCCTGTTGCACAGGATCTGGCAAGTCTGAAAATCCCTGAGCAATTTCGACATGCGATCTGGAAGGGCATCCTGGACCACCGGCAGCTCCACGAATTCTCCTCCCCTTCTCATCTCCTGCGGACCCCAAGCAGTGCCTCTACAGTCAGTGTGGGCTCCAGTGAGACCCGGGGTGAGCGTGTTATTGATGCTGTGCGATTCACCCTCCGCCAGACCATCTCTTTCCCACCCCGAGATGAGTGGAATGACTTCAACTTTGACATGGATGCTCGCCGCAATAAGCAACAGCGCATCAAAGAGGAGGGGGAGTGAGCCTCACCATGTGAGCTCTTCCTATCCCTCTCCTAACTGCCAGCCCCCTAAAAGCACTCCTGCTTAATCTTCAAAGCCTTCTCCCTAGCTCCTCCCCTTCCTCTTGTCTGATTTCTTAGGGGAAGGAGAAGTAAGAGGCTACCTCTTACCTAACATCTGACCTGGCATCTAATTCTGATTCTGGCTTTAAGCCTTCAAAACTATAGCTTGCAGAACTGTAGCTGCCATGGCTAGGTAGAAGTGAGCAAAAAAGAGTTGGGTGTCTCCTTAAGCTGCAGAGATTTCTCATTGACTTTTATAAAGCATGTTCACCCTTATAGTCTAAGACTATATATATAAATGTATAAATATACAGTATAGATTTTTGGGTGGGGGGCATTGAGTATTGTTTAAAATGTAATTTAAATGAAAGAAAATTGAGTTGCACTTATTGACCATTTTTTAATTTACTTGTTTTGGATGGCTTGTCTATACTCCTTCCCTTAAGGGGTATCATGTATGGTGATAGGTATCTAGAGCTTAATGCTACATGTGAGTGACGATGATGTACAGATTCTTTCAGTTCTTTGGATTCTAAATACATGCCACATCAAACCTTTGAGTAGATCCATTTCCATTGCTTATTATGTAGGTAAGACTGTAGATATGTATTCTTTTCTCAGTGTTGGTATATTTTATATTACTGACATTTCTTCTAGTGATGATGGTTCACGTTGGGGTGATTTAATCCAGTTATAAGAAGAAGTTCATGTCCAAACGTCCTCTTTAGTTTTTGGTTGGGAATGAGGAAAATTCTTAAAAGGCCCATAGCAGCCAGTTCAAAAACACCCGACGTCATGTATTTGAGCATATCAGTAACCCCCTTAAATTTAATACCAGATACCTTATCTTACAATATTGATTGGGAAAACATTTGCTGCCATTACAGAGGTATTAAAACTAAATTTCACTACTAGATTGACTAACTCAAATACACATTTGCTACTGTTGTAAGAATTCTGATTGATTTGATTGGGATGAATGCCATCTATCTAGTTCTAACAGTGAAGTTTTACTGTCTATTAATATTCAGGGTAAATAGGAATCATTCAGAAATGTTGAGTCTGTACTAAACAGTAAGATATCTCAATGAACCATAAATTCAACTTTGTAAAAATCTTTTGAAGCATAGATAATATTGTTTGGTAAATGTTTCTTTTGTTTGGTAAATGTTTCTTTTAAAGACCCTCCTATTCTATAAAACTCTGCATGTAGAGGCTTGTTTACCTTTCTCTCTCTAAGGTTTACAATAGGAGTGGTGATTTGAAAAATATAAAATTATGAGATTGGTTTTCCTGTGGCATAAATTGCATCACTGTATCATTTTCTTTTTTAACCGGTAAGAGTTTCAGTTTGTTGGAAAGTAACTGTGAGAACCCAGTTTCCCGTCCATCTCCCTTAGGGACTACCCATAGACATGAAAGGTCCCCACAGAGCAAGAGATAAGTCTTTCATGGCTGCTGTTGCTTAAACCACTTAAACGAAGAGTTCCCTTGAAACTTTGGGAAAACATGTTAATGACAATATTCCAGATCTTTCAGAAATATAACACATTTTTTTGCATGCATGCAAATGAGCTCTGAAATCTTCCCATGCATTCTGGTCAAGGGCTGTCATTGCACATAAGCTTCCATTTTAATTTTAAAGTGCAAAAGGGCCAGCGTGGCTCTAAAAGGTAATGTGTGGATTGCCTCTGAAAAGTGTGTATATATTTTGTGTGAAATTGCATACTTTGTATTTTGATTATTTTTTTTTTCTTCTTGGGATAGTGGGATTTCCAGAACCACACTTGAAACCTTTTTTTATCGTTTTTGTATTTTCATGAAAATACCATTTAGTAAGAATACCACATCAAATAAGAAATAATGCTACAATTTTAAGAGGGGAGGGAAGGGAAAGTTTTTTTTTATTATTTTTTTAAAATTTTGTATGTTAAAGAGAATGAGTCCTTGATTTCAAAGTTTTGTTGTACTTAAATGGTAATAAGCACTGTAAACTTCTGCAACAAGCATGCAGCTTTGCAAACCCATTAAGGGGAAGAATGAAAGCTGTTCCTTGGTCCTAGTAAGAAGACAAACTGCTTCCCTTACTTTGCTGAGGGTTTGAATAAACCTAGGACTTCCGAGCTATGTCAGTACTATTCAGGTAACACTAGGGCCTTGGAAATTCCTGTACTGTGTCTCATGGATTTGGCACTAGCCAAAGCGAGGCACCCTTACTGGCTTACCTCCTCATGGCAGCCTACTCTCCTTGAGTGTATGAGTAGCCAGGGTAAGGGGTAAAAGGATAGTAAGCATAGAAACCACTAGAAAGTGGGCTTAATGGAGTTCTTGTGGCCTCAGCTCAATGCAGTTAGCTGAAGAATTGAAAAGTTTTTGTTTGGAGACGTTTATAAACAGAAATGGAAAGCAGAGTTTTCATTAAATCCTTTTACCTTTTTTTTTTCTTGGTAATCCCCTAAAATAACAGTATGTGGGATATTGAATGTTAAAGGGATATTTTTTTCTATTATTTTTATAATTGTACAAAATTAAGCAAATGTTAAAAGTTTTATATGCTTTATTAATGTTTTCAAAAGGTATTATACATGTGATACATTTTTTAAGCTTCAGTTGCTTGTCTTCTGGTACTTTCTGTTATGGGCTTTTGGGGAGCCAGAAGCCAATCTACAATCTCTTTTTGTTTGCCAGGACATGCAATAAAATTTAAAAAATAAATAAAAACTAATTAAGAAATTGTGTGTGTGTCTATCTGTGTCTGTTTGCCTATTTGTCCATTCTTCCATCCTTTGGCCTTGTTGATATTATCGAGGAAGAAGAGATACCGAGCAAAACACTGGAGAGTTTCAGATGTTGACGTTTCTCATCTGAGAACAAATAATCATTCTTTTCTCATTTTAGCCAACCTACAAAATAGGAAAGACCAGAATGGCATTTTCATTTGGTGAATGAGACATTCAAAGTTTTAATACCTTATTTGATTGAGGTCAGAAAGAGTTTGCTTTAGAACAGGGACTAGACTTCAAATATTCCTAATGTCTAACTTAGTGACCTTCCGATGATACTCAGCAGTGACCTCATCAGTACTGTGGGAGTGGAAAGCAAATAAGCTGGTGGTATGTGGGAGCACGCTGCAGATGATGGGCCCTGACTGGGCCATGTTCCCTTTTAAATTCATTGTTAAATATCACCCTTCAGTTATGTACATTGATTTAGCCAATACTATCTCTATCAAAATGGGTGAATTATTTTTATTAAATAAGAATGTATATTACTTTATGGGATAACATTGGATGGCATGAATACAATATAATGCCTTAGACTTATAAAATAAACTTT                                                        
