# Supplementary material for: ΔNp63α promotes radioresistance in esophageal squamous cell carcinoma through the PLEC-KEAP1-NRF2 feedback loop
Source: Cell Death Dis. 2024 Nov 5;15(11):793. doi: 10.1038/s41419-024-07194-4 (PMC11538512; doi:10.1038/s41419-024-07194-4)
Supplement: Supplementary file 5 — Supplementary Table 4 [file 41419_2024_7194_MOESM5_ESM.docx]

**Supplementary Table 4 Primer sequences for ChIP-PCR**

ΔNp63-P1-F: GGTGTTCAAATGGCTACATG

ΔNp63-P1-R: GGACGTGGCCCCAGACCTCCTG

ΔNp63-P2-F: GTTATCTTGGCCACTTACAACC

ΔNp63-P2-R: CAACATTCATGTAGAATCTGG

ΔNp63-NC-F：CACAAATGAGGAATCTGAATCC

ΔNp63-NC-R：GCATGAATCCAAATACGGTCTC

K14-F：GTCTGTGCCCAAGGTGACCT

K14-R：GCAGGGCCTCTGCACCTTAA

PLEC-P1-F: GAATCAGGCCAGGAAGATGC

PLEC-P1-R: CAGGAAGATGTAGGGACCAC

PLEC-P2-F: CAGAGGCCAGGCTGTGGTGT

PLEC-P2-R: GTGTGTGTGAGCCTCGGGCTG

PLEC-NC-F: CTTCCATGTTCTCGTGTGTC

PLEC-NC-R: GTCTCTCATTACCGACACCA

NQO1-F: GCTCTAGTTCTTTTTCCTTCACC

NQO1-R: CTGAAAAATTAGCTGGGCGTGATG
