# Supplementary figures and images for: ΔNp63α promotes radioresistance in esophageal squamous cell carcinoma through the PLEC-KEAP1-NRF2 feedback loop
Source: Cell Death Dis. 2024 Nov 5;15(11):793. doi: 10.1038/s41419-024-07194-4 (PMC11538512; doi:10.1038/s41419-024-07194-4)

Figure 1

A

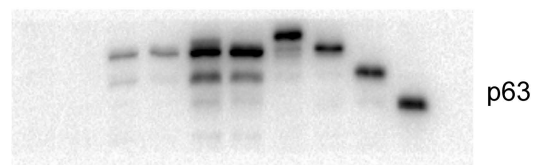

D

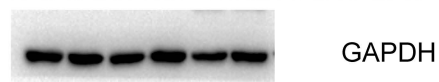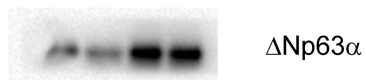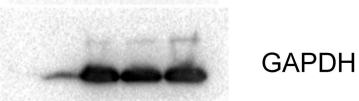

I

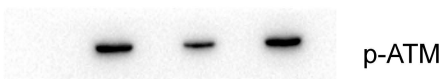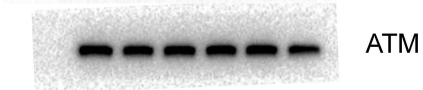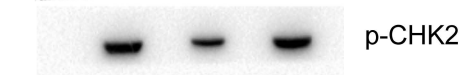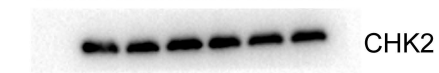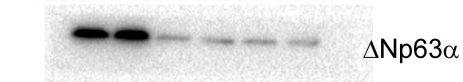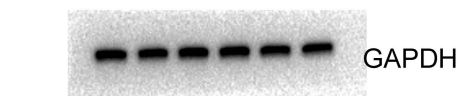

B

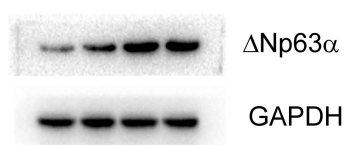

C

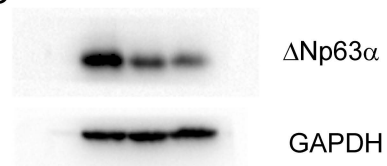

E

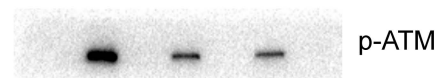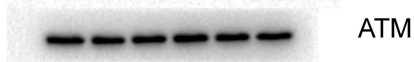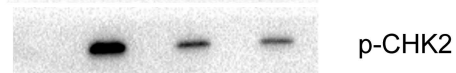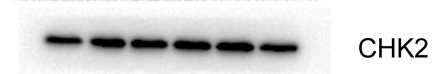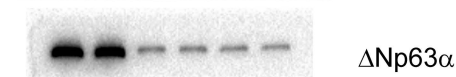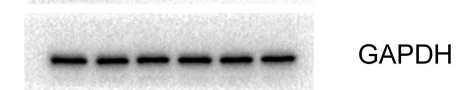

J

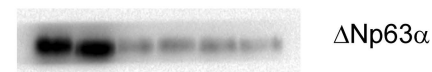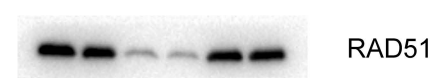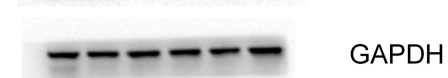

F

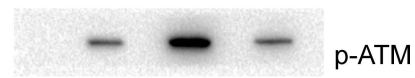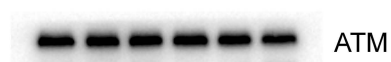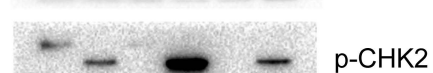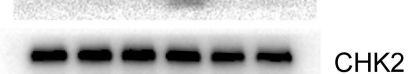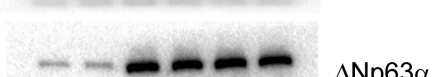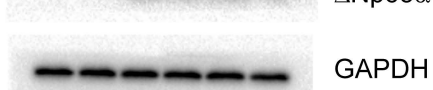

Figure 2

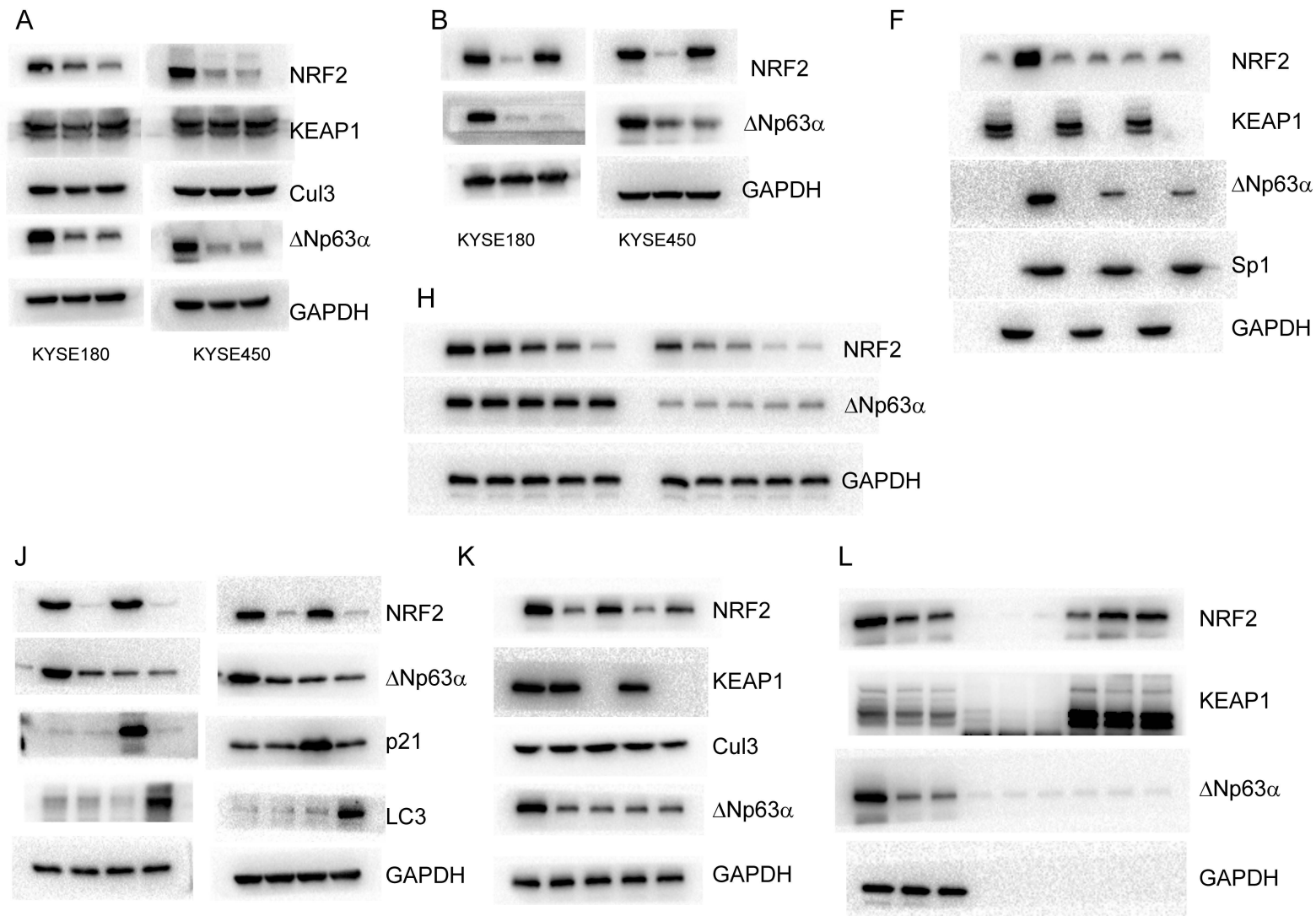

Figure 3

A

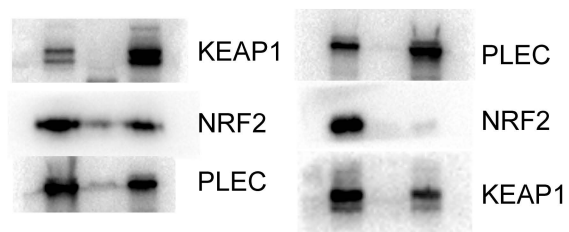

C

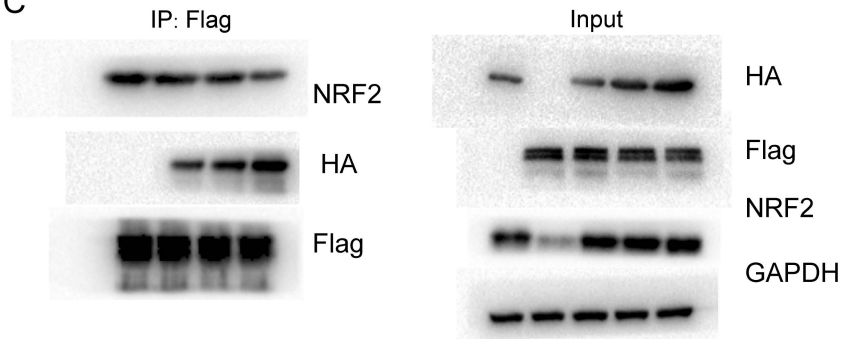

F

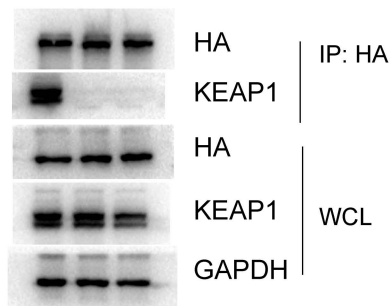

G

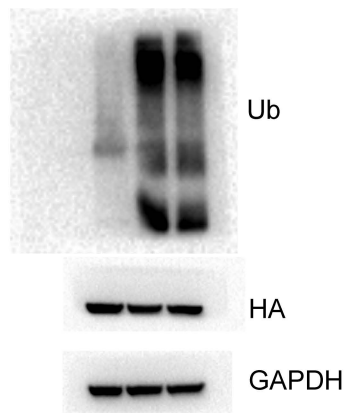

H

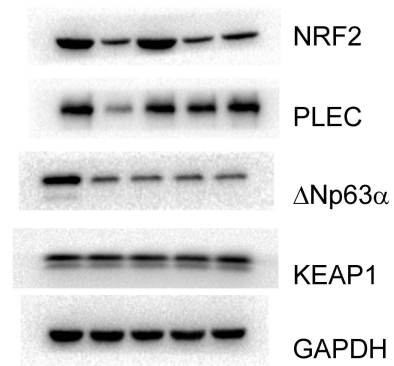

Figure 4

B

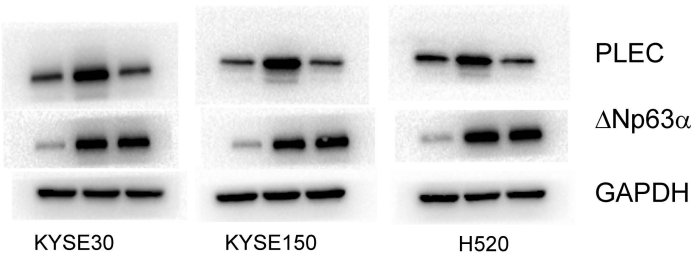

D

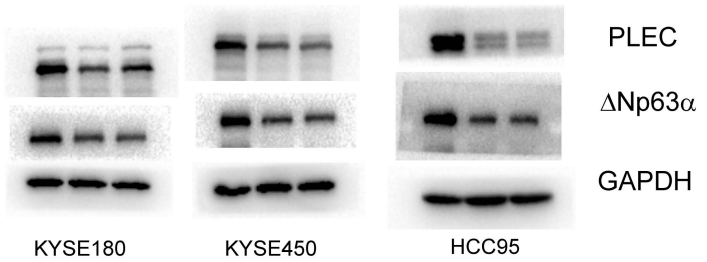

Figure 5

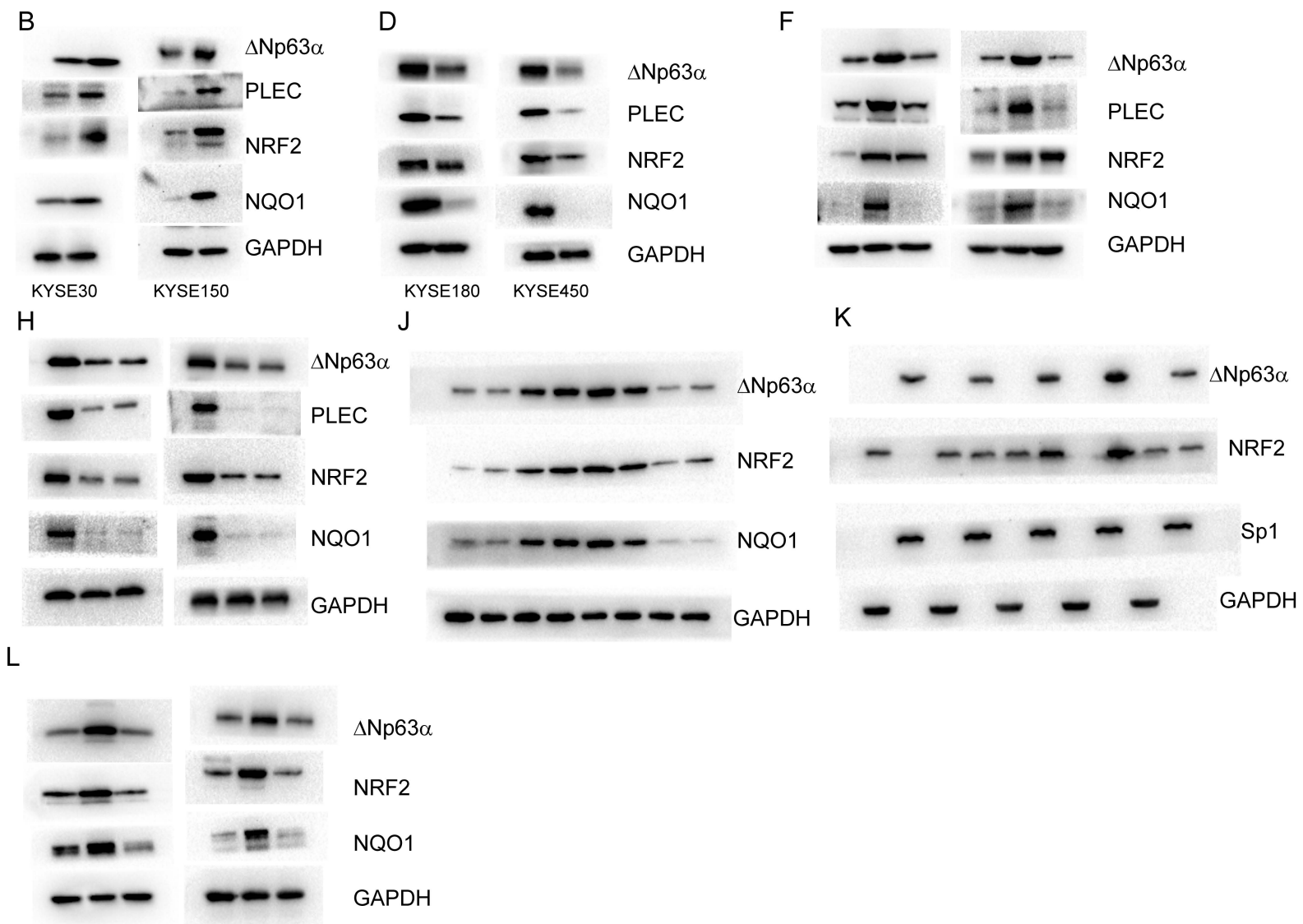

Supplement: Supplementary file 6 — Original Western blot [file 41419_2024_7194_MOESM6_ESM.pdf]
